# Linker Engineering of Hybrid Triazole-Thiazolidine Antifungals Identifies a Promising Lead Against Drug-Resistant *Candida* Species

**DOI:** 10.3390/ph19081260

**Published:** 2026-08-10

**Authors:** Alexander Yu. Rudenko, Olga A. Komarova, Alexander Yu. Simonov, Ratislav M. Ozhiganov, Dmitrii A. Averianov, Sofiia R. Kuklich, Ekaterina A. Guseva, Sofya Y. Sokolskaya, Lyudmila G. Kuz’mina, Natalia E. Grammatikova, Alexander B. Kulko, Victoria A. Bidiuk, Sofia S. Mariasina, Vasiliy A. Ivlev, Peter V. Sergiev, Vladimir I. Polshakov, Alexey B. Mantsyzov, Igor B. Levshin

**Affiliations:** 1 Belozersky Institute of Physico-Chemical Biology, M.V. Lomonosov Moscow State University, 119991 Moscow, Russiaeguseva98@mail.ru (E.A.G.);; 2 Chemical Department, M.V. Lomonosov Moscow State University, 119991 Moscow, Russia; dimitryaweryanov@yandex.ru (D.A.A.); sofia.mariasina@yandex.ru (S.S.M.); vpolsha@mail.ru (V.I.P.);; 3 Shemyakin-Ovchinnikov Institute of Bioorganic Chemistry, Russian Academy of Sciences, Miklukho-Maklaya 16/10, 117997 Moscow, Russia; 4 Gause Institute of New Antibiotics, 11 B. Pirogovskaya Street, 119021 Moscow, Russia; 5 Faculty of Fundamental Medicine, M.V. Lomonosov Moscow State University, 119991 Moscow, Russia; sofya.sokolskaya@yandex.ru; 6 N.S. Kurnakov Institute of General and Inorganic Chemistry, Russian Academy of Sciences, 119991 Moscow, Russia; 7 M.V. Lomonosov Institute of Fine Chemical Technologies, MIREA-Russian Technological University, 119571 Moscow, Russia; 8 Clinical Antituberculosis Center, Moscow Government Health Department Scientific, St. Stromynka, 10, 107014 Moscow, Russia; 9 Federal Research Center of Biotechnology of the RAS, 33-2 Leninsky Prospect, 119071 Moscow, Russia; 10 Institute of Pharmacy and Biotechnology, RUDN University, 117198 Moscow, Russia

**Keywords:** triazole-thiazolidine hybrids, antifungal activity, *Candida* spp., azole resistance, CYP51, ergosterol biosynthesis, *Candida auris*

## Abstract

**Background:** The emergence of antifungal resistance and the limited number of clinically available antifungal drug classes necessitate the development of new agents with improved efficacy and safety. We investigated how linker architecture influences the antifungal activity and lead properties of hybrid triazole-thiazolidine derivatives. **Methods:** A focused library of triazole-thiazolidine hybrids incorporating alkylamine, amide, cyclic amine, 2-hydroxypropyl, and thiazepane linkers was synthesized and characterized. Antifungal activity was evaluated against reference strains and clinical isolates of *Candida* spp., Aspergillus fumigatus, dermatophytes, and Cryptococcus neoformans. Structure–activity relationships were analyzed by molecular docking. Selected compounds were further assessed by SCRAPPY profiling, fluorescence microscopy, mammalian-cell cytotoxicity assays, acute oral toxicity studies, and evaluation of microsomal stability and interactions with human CYP450 isoforms. **Results:** Linker architecture strongly influenced antifungal potency. Amide- and cyclic amine-containing hybrids were generally the most active, whereas simple alkylamide derivatives showed narrower activity profiles. Compound **28** emerged as the most promising lead, exhibiting sub-microgram MIC values against several *Candida* isolates, particularly *C. parapsilosis*, and retaining measurable activity against an azole-resistant *C. albicans* strain. Docking generated putative CYP51-binding models, while SCRAPPY profiling and fluorescence microscopy revealed an azole-like cellular response consistent with perturbation of sterol-associated homeostasis. Compound **28** was tolerated at 300 mg/kg in an acute oral study but showed concentration- and time-dependent cytotoxicity and rapid CYP3A4-mediated microsomal metabolism. **Conclusions:** Systematic variation of linker architecture identified compound **28** as a promising exploratory antifungal lead. Further optimization should focus on improving metabolic stability and cytotoxicity, together with direct target validation, pharmacokinetic characterization, and in vivo efficacy studies.

## 1. Introduction

Invasive fungal infections (IFIs) represent a severe and steadily growing clinical challenge, particularly affecting immunocompromised patients, including patients with HIV infection, recipients of solid organ or hematopoietic stem cell transplants, patients with diabetes mellitus, and critically ill individuals receiving intensive care or cytotoxic chemotherapy. Despite advances in antifungal therapy, IFIs remain associated with high morbidity and mortality, underscoring the urgent need for new therapeutic strategies [1,2,3].

The development of selective antifungal agents is complicated by the shared eukaryotic biology of fungal and human cells, which limits the number of fungus-specific molecular targets and can narrow the therapeutic window. The major classes of clinically used antifungal drugs, including polyenes, echinocandins, and azoles, can cause clinically relevant adverse effects. These include hepatotoxicity, which may range from asymptomatic elevations in liver enzyme levels to severe hepatic injury [4,5]. The identification of antifungal agents that combine potent activity with improved safety therefore remains an important unmet medical need [6].

Among the currently approved antifungal drugs, azole derivatives, particularly triazoles, have played a central role in the treatment of IFIs over the past several decades [7,8,9]. Their principal molecular target is lanosterol 14α-demethylase (CYP51), a key enzyme in the ergosterol-biosynthesis pathway that catalyzes oxidative removal of the 14α-methyl group from sterol precursors. Inhibition of CYP51 disrupts ergosterol production and alters fungal membrane structure and function. Although CYP51 is a clinically validated antifungal target, the number of approved inhibitors remains limited, and resistance to azole antifungals continues to increase [10].

Azole resistance can arise through several mechanisms, including mutations or overexpression of CYP51, increased expression of efflux transporters, alterations in sterol metabolism, and biofilm formation. These mechanisms can substantially reduce therapeutic efficacy. The problem is particularly important for clinically relevant *Candida* species, including *Candida auris*, an emerging multidrug-resistant pathogen associated with healthcare-associated outbreaks and treatment failure [11,12]. The development of new azole-like molecules that retain activity against clinically relevant and azole-resistant *Candida* isolates therefore remains an important objective in antifungal drug discovery.

In addition to acquired resistance, the clinical utility of existing CYP51 inhibitors may be limited by off-target interactions with human cytochrome P450 isoforms, which can contribute to drug–drug interactions and toxicity. Current medicinal chemistry efforts have therefore focused on improving fungal CYP51 selectivity while maintaining antifungal potency. A notable example is oteseconazole (VT-1161), a tetrazole-containing CYP51 inhibitor developed by Viamet Pharmaceuticals and approved by the US Food and Drug Administration for recurrent vulvovaginal candidiasis. Although oteseconazole shows improved selectivity and a favorable safety profile, its clinical use is restricted because of the risk of embryo-fetal toxicity, illustrating the continuing challenges associated with this target class [13].

As part of our ongoing efforts to develop hybrid antifungal agents containing a triazole pharmacophore, we previously reported a series of compounds incorporating sulfur-containing heterocycles, including thiazolo[4,5-d]pyrimidines [14] and thiazolidine-2,4-diones [15,16]. In these studies, alkylpiperazine-linked derivatives showed greater activity against *Candida parapsilosis* compared with fluconazole, whereas acetamide-linked analogues were less potent. Replacement of the thiazolo[4,5-d]pyrimidine moiety with a thiazolidine-2,4-dione fragment connected through a piperazinyl acetamide linker substantially enhanced antifungal activity. The resulting compound L173 exhibited MIC values comparable to or lower than those of voriconazole against selected strains.

Hybrid molecule design has attracted considerable interest as a strategy for improving biological activity by combining complementary pharmacophoric elements within a single chemical scaffold. In antifungal drug discovery, thiazolidine-2,4-dione derivatives are of interest because of their broad pharmacological potential and their reported effects on fungal cell-wall biosynthesis, possibly involving inhibition of mannosyltransferases [17,18,19]. Amide-linked triazole derivatives have also demonstrated potent antifungal activity in several studies [20,21,22,23,24,25,26,27,28,29].

Building on these observations, the present study systematically examined the influence of linker architecture on antifungal activity within a triazole-thiazolidine hybrid scaffold. We synthesized a series of hybrids containing alkylamine, amide, hydroxyalkyl, and cyclic amine linker motifs. Particular attention was given to oxygen-containing groups positioned near a basic nitrogen atom, based on the hypothesis that these structural features might modulate antifungal activity, physicochemical properties, and the predicted orientation of the compounds within a putative CYP51-binding site.

The synthesized compounds were evaluated for in vitro antifungal activity against selected reference strains and clinical isolates of *Candida* spp., as well as *Aspergillus fumigatus*. Molecular docking was used to generate putative CYP51-binding models and to provide a possible structural rationale for the observed structure–activity relationships. Because direct biochemical target validation was not performed, the docking results were interpreted as mechanistic hypotheses rather than evidence of CYP51 engagement.

The aim of this work was to design, synthesize, and biologically evaluate a new series of triazole-thiazolidine hybrid antifungal agents, define the principal structure–activity relationships governing their potency and spectrum of activity, and identify a lead scaffold suitable for subsequent medicinal chemistry optimization, mechanistic validation, and pharmacological characterization.

## 2. Results and Discussion

### 2.1. Chemical Synthesis of Hybrid Compounds

**Figure 1:**
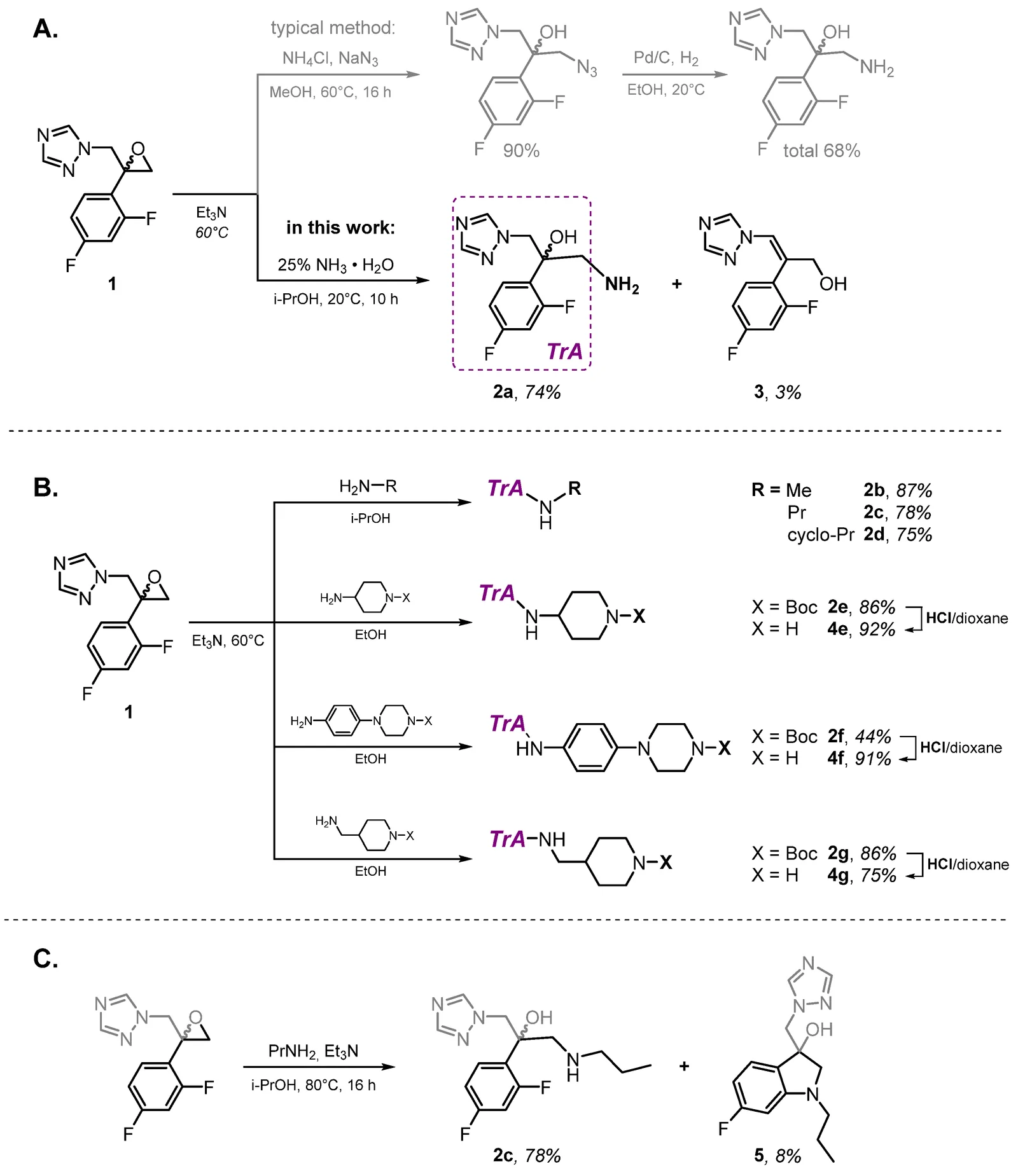
Synthetic routes to triazole intermediates used for the preparation of hybrid compounds. (**A**) Synthesis of aminotriazole using the previously reported and improved methods. (**B**) General synthetic scheme for the preparation of amino-substituted triazoles **2**–**4**. (**C**) Synthetic scheme for the preparation of propylaminotriazole **2c** and propylindoline **5**.

Within this study, several series of hybrid compounds incorporating a triazole moiety and sulfur-containing heterocycles were synthesized. These series differed in the nature of the molecular linker. Their preparation involved both previously reported compounds, obtained using the unified methodology shown in Figure 1B, and newly prepared triazole intermediates (**2f**, **4f**). Intermediates **2e** and **2g** were previously obtained by reductive amination of 2-aminotriazole **2a** with 1-Boc-piperidin-4-one [30] and 1-Boc-4-formylpiperidine [31,32], respectively, although only in a low yield of 25%.

In all epoxide ring-opening reactions, commercially available triazole-containing epoxide **1** (Figure 1) was used as the key starting material and reacted with either commercially available or specially synthesized amines. This approach ensured the versatility of the synthetic strategy and enabled modulation of the linker structure without substantial changes in the reaction conditions.

Aminotriazole **2a** (Figure 1A) was one of the key intermediates used for the synthesis of the hybrid molecules. This important intermediate is frequently described in the literature as a precursor for various triazole derivatives [20]. Traditionally, its synthesis is accomplished via a two-step procedure starting from epoxide **1** and involving sodium azide (Figure 1A). Significant drawbacks of this route include the use of potentially hazardous reagents such as sodium azide and hydrogen, as well as the requirement for an expensive 10% Pd/C catalyst.

In the present work, an alternative and milder method for the synthesis of compound **2a** was employed, based on the reaction of epoxide **1** with aqueous ammonia in an alcoholic medium at room temperature (Figure 1A). Under these conditions, the target product **2a** was obtained in a good yield of 74%. The formation of amino alcohols was evidenced by the disappearance of epoxide proton signals and the appearance of CHOH and CH_2_N resonances at δH 3.2–4.8 ppm. The expected [M + H]^+^ ions were observed in the ESI-MS spectra. Notably, allylic alcohol **3** (Figure 1A) was isolated as a byproduct of this reaction.

Previously, the synthesis of this compound had been reported only under significantly harsher conditions, namely by reaction of epoxide **1** with sodium tert-butoxide in a tert-butanol/DMSO system at room temperature. This approach was considered superior to numerous alternative methodologies involving heating with strong bases [31,32]. Moreover, the literature indicated that the resulting allylic alcohol **3** was an isomeric compound with undefined configuration. In this context, a detailed structural investigation of compound **3** was performed using NMR spectroscopy, which allowed the structure shown in Figure 1 to be unambiguously confirmed.

Reaction of epoxide **1** with various amines (Figure 1B) afforded the corresponding amino-substituted triazole derivatives in good yields that were largely independent of the nature of the amine. In the case of diamines bearing a Boc-protected amino group, deprotection was achieved by treatment with hydrogen chloride in dioxane (Figure 1B). Boc removal was confirmed by the disappearance of the tert-butyl singlet at 1.4 ppm.

Upon reaction of epoxide 1 with propylamine in isopropanol, increasing the reaction temperature and time up to 16 h led, in addition to the formation of the major product **2c**, to the isolation of 1-propylindoline **5** as a side product in 8% yield (Figure 1C). The structure of compound **5** was established by NMR spectroscopy and mass spectrometry. Structurally related indoline derivatives have previously been reported in reactions of methyl-substituted epoxides with aliphatic amines. However, their formation required considerably harsher conditions, namely heating in an autoclave at 70–100 °C for 24–46 h [20]. In the present case, the formation of compound **5** under substantially milder conditions points to a distinctive reactivity of this system.

**Figure 2:**
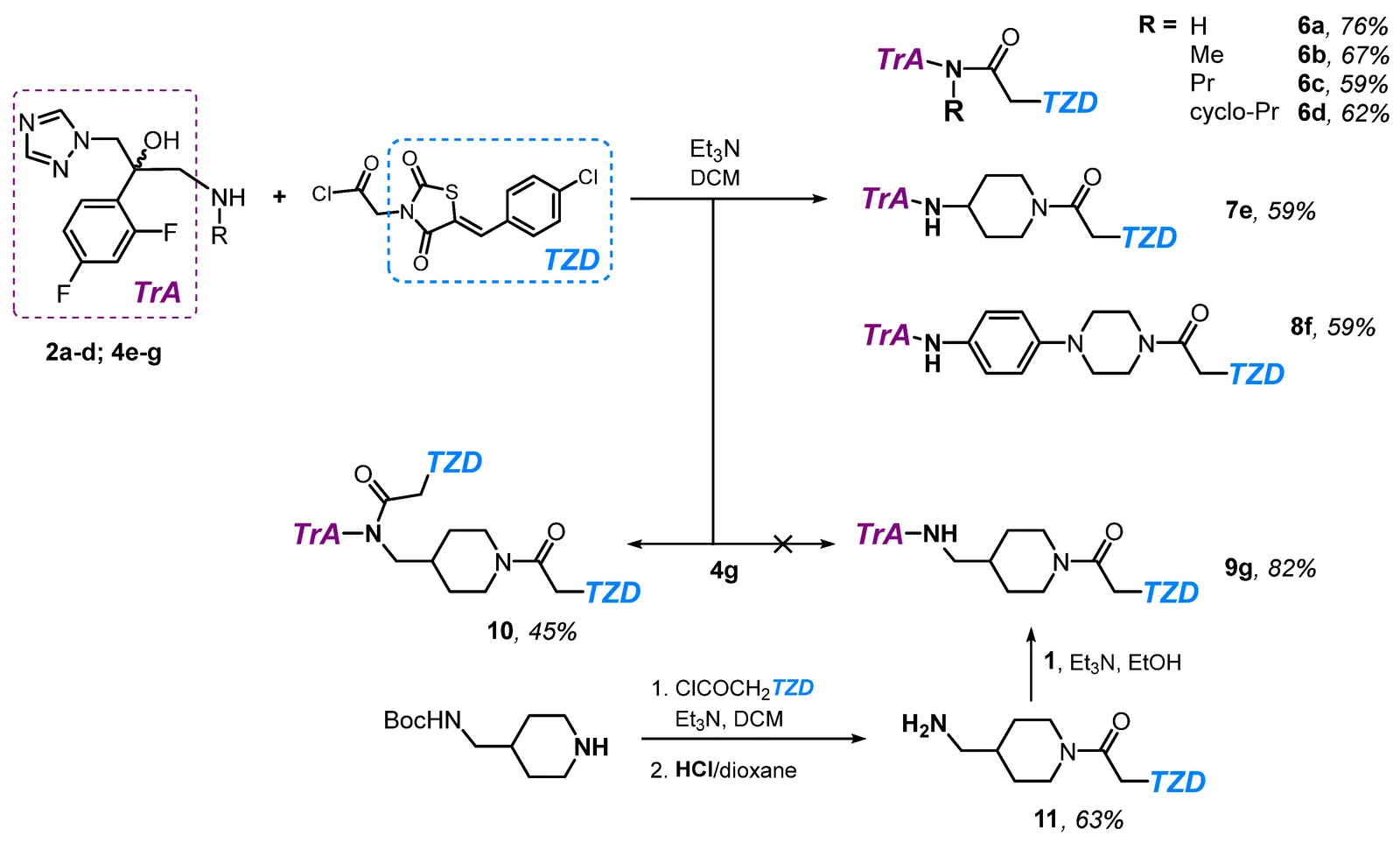
Synthetic schemes for the preparation of hybrid compounds **6**–**10**. Triazole intermediates bearing alkylamine or cyclic amine linkers were acylated with the thiazolidine-2,4-dione-containing acyl chloride. An alternative stepwise route involving intermediate **11** was used to obtain compound **9g** and avoid formation of the bis-acylated product **10**.

For the synthesis of hybrid derivatives **6** to **10** (Figure 2), triazole intermediates **2a**–**d** and **4e**–**g** were subjected to acylation with a thiazolidine-2,4-dione-containing acyl chloride (**TZD**, Figure 2) in dichloromethane in the presence of triethylamine. However, this approach failed to afford the target compound **9g**, as the reaction was accompanied by simultaneous acylation of both amino groups, resulting in the formation of the bis-acylated product **10**. Therefore, the synthetic route to hybrid **9g** was modified. Commercially available Boc-protected aminomethylpiperidine and the acyl chloride of the thiazolidine-2,4-dione derivative were used as starting materials. The reaction was performed under conditions analogous to those used for the synthesis of aminotriazoles **6** to **8** and, after deprotection, afforded the TZD amino derivative **11**. Subsequent reaction of compound **11** with epoxide **1** in ethanol in the presence of triethylamine gave the target hybrid **9g** (Figure 2).

**Figure 3:**
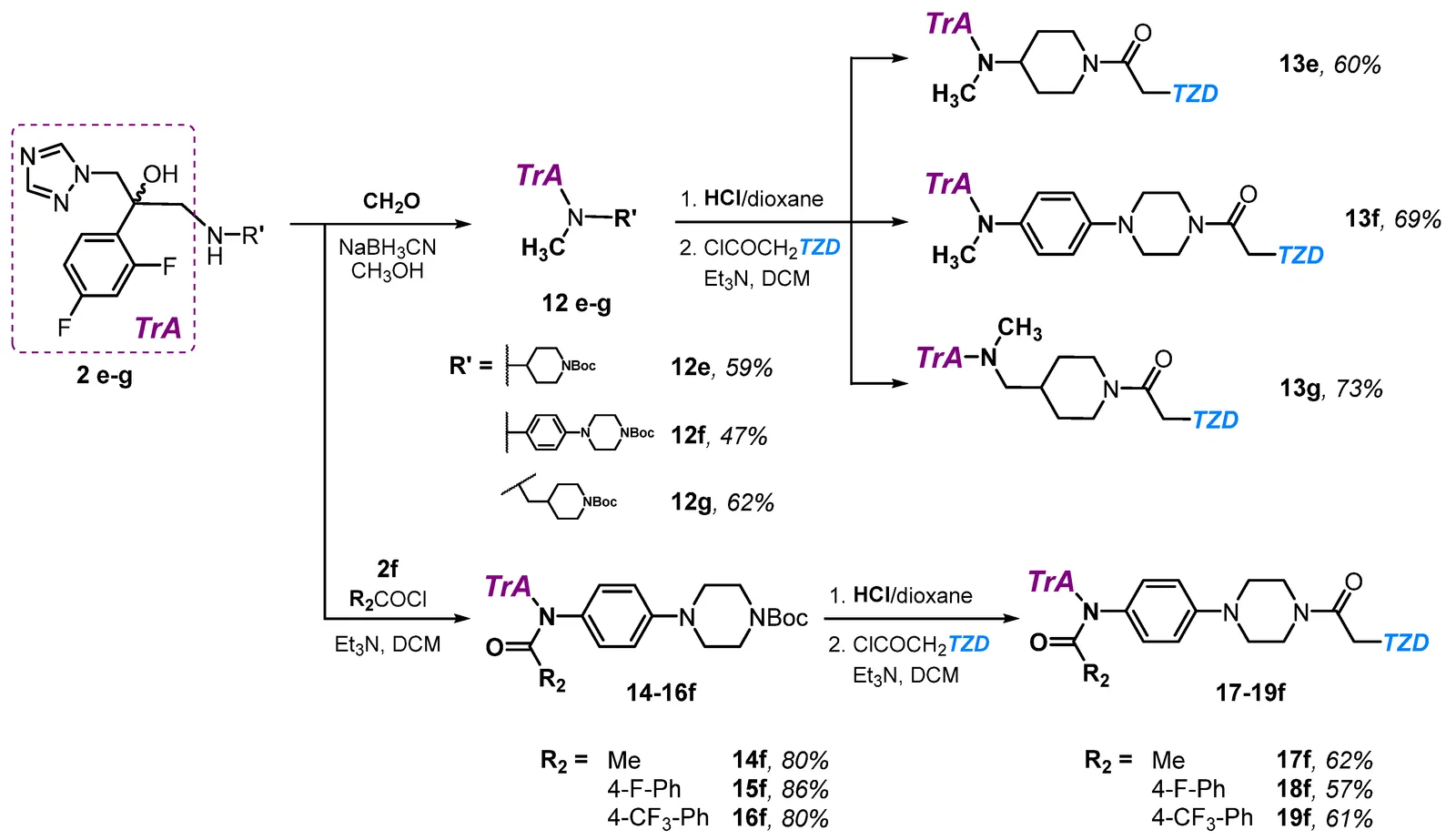
Synthesis of amide amino-substituted hybrid derivatives. Reductive N-methylation of protected amino-substituted triazoles afforded intermediates **12e**–**g**, which were subsequently deprotected and acylated to produce compounds **13e**–**g**. Acetylation or aroylation of phenylpiperazine-containing intermediates, followed by deprotection and coupling with the thiazolidine-2,4-dione fragment, afforded bis-amide derivatives **14**–**19f**.

For comparative microbiological evaluation, a systematic analysis of the effect of substitution at the amino group of the triazole fragment in compounds **7e**, **8f**, and **9g** was undertaken. To this end, a series of chemical modifications was performed, involving the introduction of methyl, acetyl, and aroyl substituents at the proximal nitrogen atom (Figure 3).

Introduction of a methyl substituent into the hybrid compounds was achieved using a unified reductive amination protocol applicable to all series. Specifically, the amino derivatives **2e** to **2g** obtained at the epoxide ring-opening stage were reacted with formaldehyde in the presence of sodium cyanoborohydride in methanol, affording the corresponding N-methylated intermediates **12e** to **12g** (Figure 3). The reaction proceeded selectively and reproducibly, with no appreciable formation of side products.

Subsequent Boc deprotection by treatment with hydrogen chloride, followed by acylation, afforded the final amide derivatives **13e**–**g** (Figure 3). N-Methylation was indicated by the appearance of an N-CH_3_ singlet at 2.2–3.2 ppm and the corresponding methyl carbon signal in the ^13^C NMR spectrum. This stepwise approach avoided uncontrolled polyacylation and ensured high purity of the target compounds.

For the preparation of bis-amide derivatives based on diamines **2e**–**g**, acid chlorides of acetic and aromatic acids were employed. However, most of the resulting bis-acylated compounds exhibited limited stability and partially decomposed during column chromatography. An exception was represented by derivatives containing a phenylpiperazine fragment (**2f**), for which compounds **14**–**16f** could be successfully isolated and characterized. These intermediate bis-amides, after Boc deprotection and subsequent acylation with the thiazolidine-2,4-dione-containing acyl chloride under the previously described conditions, afforded the target hybrid diamides **17**–**19f** (Figure 3). These results indicate a pronounced influence of the substituent within the linker moiety on the chemical stability of the amide derivatives, which was taken into account in the subsequent compound design.

**Figure 4:**
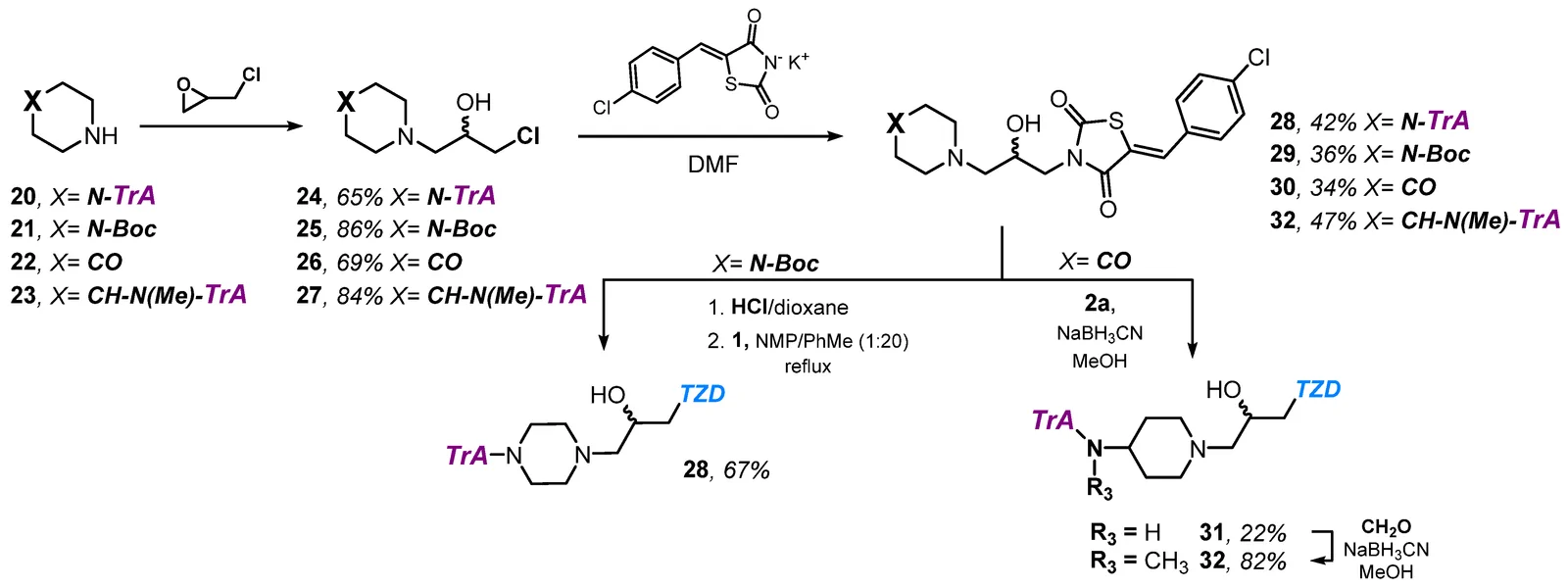
Synthetic scheme for the preparation of 2-hydroxypropyl analogues of the amide hybrid compounds. Compounds **28**, **31**, and **32** were prepared using complementary convergent and stepwise synthetic approaches. Compound **28** was obtained through coupling of the triazole-containing chlorohydrin with a thiazolidine-2,4-dione derivative or by epoxide ring opening with the corresponding piperazine intermediate. Reductive amination and subsequent N-methylation afforded aminopiperidine derivatives **31** and **32**, respectively.

The third group of hybrid compounds was designed based on a bioisosteric approach involving replacement of the acetamide group at position **3** of the thiazolidine-2,4-dione fragment with a 2-hydroxypropyl substituent (Figure 4). This modification was intended to assess the effect of a polar and conformationally more flexible fragment on the biological activity and physicochemical properties of the compounds.

For the synthesis of the corresponding hybrids bearing a triazole fragment linked through a hydroxypropyl spacer (**28**, **31**, **32**), TZD derivatives **29** (X = N-Boc), **30** (X = CO), and **32** were employed. These compounds were prepared according to reported procedures starting from piperidin-4-one **22** [33] and Boc-protected piperazine **21** [34]. Reaction of compounds **21** and **23** with epichlorohydrin in an alcoholic medium afforded the corresponding chlorohydrin derivatives **25** and **27**, which were subsequently reacted with the potassium salt of a thiazolidine-2,4-dione derivative in DMF according to our previously described methodology [35]. An analogous triazole-containing intermediate **24** was obtained from precursor **20** using a modified procedure developed in our group, involving the addition of epichlorohydrin and tetrabutylammonium chloride as a catalyst in N-methylpyrrolidone (Figure 4). The subsequent condensation with the potassium salt of the TZD derivative was carried out in DMF under heating to afford compound **28** [36]. To confirm the structures of compounds **28** and **32**, a convergent synthetic approach was employed starting from thiazolidinedione intermediates **29** and **30**, respectively, and the corresponding triazole fragments.

Hybrid **28** was also obtained by an alternative route. After Boc deprotection of the 3-(2-hydroxypropyl)piperazinyl thiazolidine-2,4-dione derivative **29**, the resulting amine was used in the next step without additional purification and reacted with epoxide **1** in a toluene/N-methylpyrrolidone (20:1) mixture in the presence of triethylamine. These conditions enabled efficient formation of hybrid compound **28**, which had previously been obtained by a convergent synthesis from the 3-chloro-2-hydroxypropyl derivative **24** and the TZD potassium salt in DMF under heating. Reductive amination of piperidone derivative **30** with aminotriazole **2a** afforded aminopiperidine hybrid **31**. Subsequent N-methylation of **31** with formaldehyde and sodium cyanoborohydride in dichloromethane furnished compound **32** (Figure 4).

### 2.2. Unexpected Transformation of the Thiazolidine Fragment: Formation of Thiazepane Derivatives

**Figure 5:**
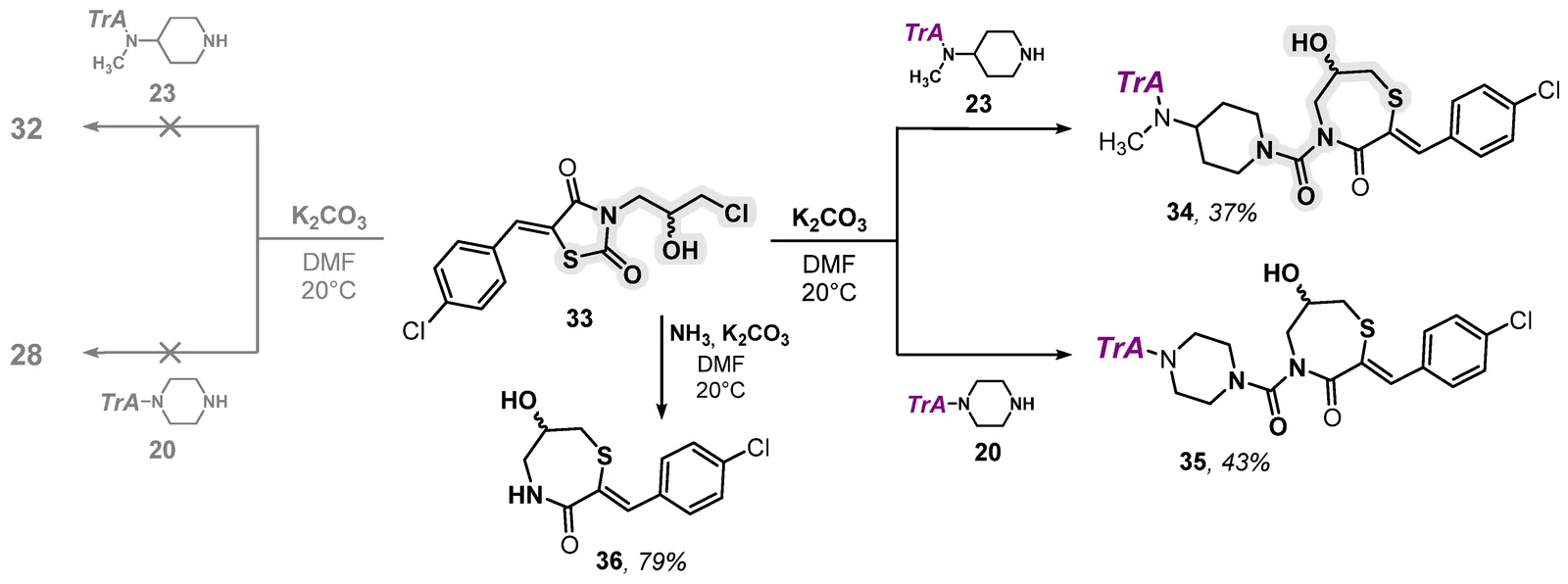
Synthetic scheme for the preparation of thiazepane hybrid compounds **34** and **35**. Base-induced transformation of the N-(3-chloro-2-hydroxypropyl) thiazolidine-2,4-dione derivative **33** in the presence of secondary amines afforded the rearranged thiazepane products **34** and **35**.

During attempts to synthesize compounds **28** and **30** from the previously reported N-(3-chloro-2-hydroxypropyl) thiazolidine-2,4-dione derivative **33** in DMF at room temperature in the presence of potassium carbonate, thiazepane hybrid compounds **34** and **35** were obtained instead (Figure 5) [37]. We have previously demonstrated that compound **33** can undergo base-induced transformation in alcoholic media, leading to the formation of oxazolidine derivatives. Under the conditions employed in the present study, the observed results suggest an alternative reaction pathway involving cleavage of the thiazolidine ring at the R–S–C(2)=O bond, with formation of an isocyanate intermediate, R–N(3)CO. Subsequent reaction of this intermediate with a secondary amine, followed by intramolecular cyclization involving the sulfur atom, results in formation of a seven-membered 1,3-thiazepane ring containing a carbamate fragment. The structures of compounds **34** and **35** were established by analysis of 2D NMR spectra, in which formation of the seven-membered ring was confirmed by characteristic correlations in the ^13^C-^1^H HMBC and ^1^H-^1^H ROESY spectra. Additional support for this structural assignment was provided by formation of the model compound **36** via a similar rearrangement; its structure was confirmed not only by NMR spectroscopy but also by X-ray crystallographic analysis (Supplementary Figure S1 and S2, Supplementary Table S1).

### 2.3. Antifungal Activity of the Synthesized Hybrid Compounds

Previously, we demonstrated that attachment of 5-arylidene thiazolidine-2,4-dione derivatives to the triazole core through a piperazinyl amide linker results in pronounced antifungal activity against both *Candida* spp. yeasts and dermatophytes, including *Microsporum canis* and *Trichophyton rubrum*. In that study, the highest activity was observed for compound **L173**, bearing an acetamide piperazinyl linker connected to a thiazolidine-2,4-dione fragment substituted with a p-chlorobenzylidene group at the 5-position of the heterocyclic ring [15,16].

In the present work, this structural motif was used as a starting point for further optimization aimed at elucidating the influence of linker architecture on antifungal activity while preserving the triazole and thiazolidine pharmacophoric fragments. As a result of the synthetic effort, four distinct groups of hybrid compounds were generated.

The first group comprised compounds containing either an amide linker (**6a**), alkylamide linkers (**6b**–**d**), cyclic amino linkers (**7e**, **8f**, **9g**), or their N-methylated analogues (**13e**–**g**). The second group included bis-amide derivatives bearing amide linkers (**14**–**19f**). The third group consisted of bioisosteric analogues incorporating a 2-hydroxypropyl linker (**28**, **31**, **32**). The fourth group was represented by triazole-thiazepane hybrid compounds (**34**, **35**). This compound library design enabled a systematic comparative analysis of the effects of linker polarity, flexibility, and degree of substitution on biological activity within a unified pharmacophore framework.

The antifungal activity of all synthesized compounds was evaluated in vitro against a panel of reference fungal strains, including *Candida parapsilosis* ATCC 22019, *Candida albicans* 604M, *Candida krusei* 432M, *Candida auris* VGNKI, and *Aspergillus fumigatus* ATCC 46645 (Table 1). Commercial antifungal agents fluconazole, voriconazole, and posaconazole were used as reference compounds.

All biological experiments were performed in three independent replicates. Minimum inhibitory concentration (MIC) values were determined after 24 and 48 h of incubation, and the data presented in Table 1, Table 2 and Table 3 represent mean values calculated from three experiments. In addition, it was shown that intermediates used in the synthesis of the target hybrid compounds did not exhibit detectable antifungal activity, confirming the critical role of the hybrid molecular architecture in the observed biological effect.

Hybrid compound **6a**, containing only an amide-linked thiazolidine fragment, exhibited high to moderate antifungal activity against all tested fungal strains. Notably, its activity against the multidrug-resistant *C. auris* strain was markedly superior to that of the reference antifungal agents fluconazole, voriconazole, and posaconazole (MIC 0.25 µg/mL versus 4–32 µg/mL). In contrast, compound **6a** displayed only moderate activity against *A. fumigatus*.

Hybrids bearing alkylamino-triazole fragments **6b**–**d** and incorporating a thiazolidine-2,4-dione core showed moderate activity against *C. parapsilosis* and *C. albicans*, but were essentially inactive against *C. krusei*, *C. auris*, and *A. fumigatus*. These findings indicate that simple alkylamide linkers make only a limited contribution to broadening the antifungal activity spectrum.

Comparison of structurally related hybrid derivatives **7**–**9g**, containing cyclic amine moieties, revealed that compound **9g**, bearing a free amino group, displayed the most favorable overall activity profile. For *C. parapsilosis* and *C. albicans*, the MIC values of this compound were lower than or comparable to those of fluconazole, with the exception of activity against *C. krusei* and *A. fumigatus*. Remarkably, compounds **7e** and **9g** demonstrated higher activity against the multidrug-resistant *C. auris* strain than voriconazole and posaconazole (MIC 0.5–1.0 vs. 2–8 µg/mL).

N-Methylation of the nitrogen atom in compounds **13e**–**g** exerted an ambiguous effect on antifungal activity. While enhanced inhibitory potency was observed against *C. parapsilosis* (MIC 0.0125–0.25 µg/mL), activity against *C. krusei* remained moderate (MIC 4–8 µg/mL), and no overall expansion of the activity spectrum was achieved.

Introduction of acetyl or aroyl substituents at the nitrogen atom in compounds **14**–**19f** resulted in a complete loss of antifungal activity, highlighting the critical importance of a free or only weakly substituted amino group for productive interaction with the biological target. Against *A. fumigatus*, only compounds **7e** and **9g** exhibited moderate activity (MIC ≈ 8 µg/mL), whereas all other derivatives were inactive.

Compounds from the third and fourth groups were screened against an extended panel of clinical *Candida* spp. isolates, including the triazole-resistant *C. albicans* 8R strain (Table 2). Fluconazole and itraconazole were used as reference drugs. All tested compounds outperformed fluconazole against the clinical isolates and, in several cases, demonstrated activity comparable to or exceeding that of itraconazole. The most pronounced activity against *C. parapsilosis* was observed for aminopiperidine analogues **31** and **32**, incorporating a 2-hydroxypropyl linker attached to the thiazolidine-2,4-dione fragment (MIC 0.0125–0.05 µg/mL).

The piperazine derivative **28**, featuring the same linker type, showed slightly reduced activity against most strains but exhibited more pronounced inhibitory effects against the resistant *C. albicans* 8R strain (MIC 0.5–8 µg/mL), which is resistant to all investigated triazole agents. This observation suggests a potential role of increased linker flexibility in overcoming antifungal resistance.

Replacement of the thiazolidine ring with a seven-membered thiazepane core (compounds **34** and **35**), while preserving the key functional motif (–S–C=CH–(4-Cl-Ph)–CO–NH–), resulted in retention of high activity against *C. parapsilosis* (MIC 0.125–1.0 µg/mL) and moderate activity against other yeast species (*C. krusei*, *C. tropicalis*, *C. glabrata*, *C. utilis*). However, activity against resistant *C. albicans* strains 8R and 604M was lost, underscoring the sensitivity of the biological effect to the topology of the heterocyclic fragment.

In the microbiological evaluation of antifungal activity against dermatophytes, moderate activity against *M. canis* B-200 and *T. rubrum* U.2002 was observed only for two representatives containing an amide linker, compounds **7e** and **13e**. In contrast, replacement of the linker with diamine-derived fragments, namely piperazine or aminopiperidine moieties bearing a 2-hydroxypropyl group, resulted in a marked increase in antifungal activity for compounds **28**, **31**, and **32**. The highest activity was observed for the N-unsubstituted aminopiperidine derivative **31**, which inhibited both dermatophyte strains with MIC values of 0.03 µg/mL. Commercial antifungal agents, including fluconazole, itraconazole, and amphotericin B, were used as reference compounds. The results of the microbiological evaluation, performed in triplicate, are summarized in Table 3.

Against *Cryptococcus neoformans*, appreciable antifungal activity was observed only for compounds **13e** and **28**, with MIC values of 2 µg/mL and 0.25 µg/mL, respectively. The remaining derivatives showed only weak activity against this strain.

### 2.4. Molecular Modelling

**Figure 6:**
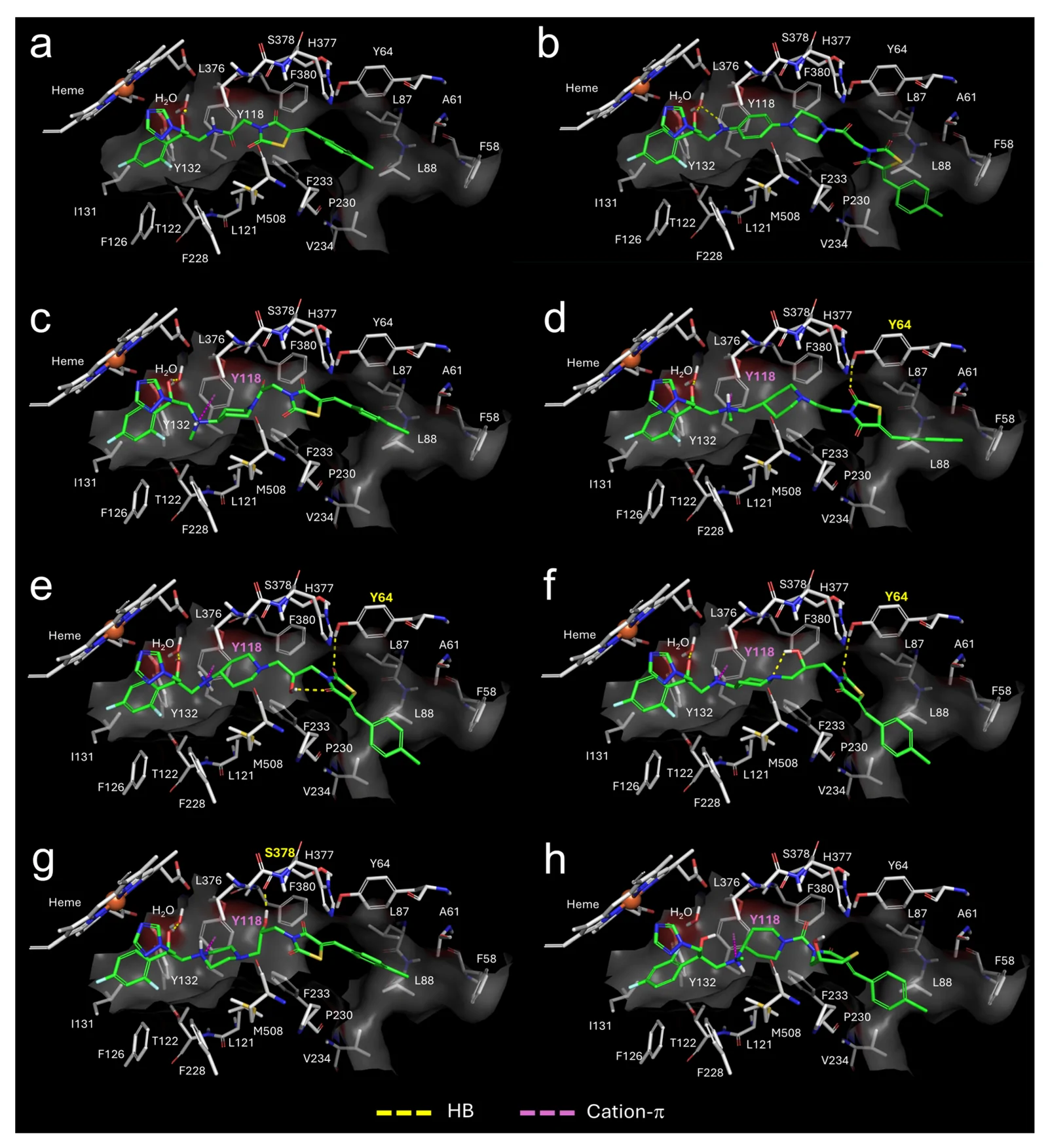
Predicted binding modes of synthesized ligands in the active site of CYP51: (**a**) **6a**, (**b**) **8f**, (**c**) **13e**, (**d**) **13g**, (**e**) **31**, (**f**) **32**, (**g**) **28**, (**h**) **34**. Hydrogen bonds are shown by yellow dashed lines; cation–π interactions are in purple. The labels for the residues engaged in polar contacts are colored and enlarged. Key residues are depicted by sticks; backbone is not shown in some cases for clarity. A portion of the binding site surface is shown.

Molecular docking was used to probe potential binding modes of synthesized molecules in the active site of CYP51 and interpret experimentally observed SAR (Table 1 and Table 2; below data for *C. parapsilosis* ATCC 22019 is interpreted). The screened compounds can be sorted into two groups: (1) amides without basic nitrogen in the linker (**6a**–**d**, **8f**, **13f**–**19f**) and (2) amines featuring basic nitrogen (**7e**, **9g**, **13e**, **13g**, **28**, **31**, **32**, **34**, **35**). Compounds **6a**, **6b**, **6d**, **7e**, **8f**, **9g**, **13e**–**f**, **28**, **31**, **32**, and **34** fitted within the binding pocket. The fluconazole core reproduced the expected orientation, with the hydroxyl group making a hydrogen bond with the structural water molecule residing within the binding site (Figure 6). The sidechain arms of group 1 ligands did not form polar contacts with the receptor (Figure 6a,b). Analogously to the earlier described TZD series [15], ligands with short linker between the fluconazole and TZD cores (**6a**–**d**) were buried within the binding site where the position of the side chain arm was stabilized by the hydrophobic contacts with the lipophilic interior of the substrate access channel (residues Y64, L87, Y118, L121, T122, Y132, P230, F233, L376, H377, F380, M508; Figure 6a). Unsubstituted **6a** demonstrated more than 30 times higher activity as compared to the N-alkylated derivatives **6b**–**d**. This clearly indicated that branching of the sidechain arm bound in the narrow substrate access channel is unfavorable due to the steric factor. Indeed, **6b** and **6d** showed perturbed binding modes as compared to **6a** (Supplementary Figure S3a), whereas **6c** did not fit within the binding site. Compound **8f** featured the sidechain arm elongated by the phenylpiperazine insert. This resulted in higher cLogP (3.4 for **8f** as compared to 1.9 for **6a**) and allowed additional lipophilic contacts between the p-chlorophenyl moiety and the solvent-exposed residues at the gate to the binding site (F58, A61, L88, V234; Figure 6b). Despite the higher capacity to form hydrophobic interactions, the activity of **8f** was not improved (24 h MIC 0.25 and 0.06 µg/mL for **8f** and **6a**, respectively). Similarly to **6a**, branching of the sidechain arm of **8f** was poorly tolerated: although the N-methylation did not alter activity in this case (**13f**), introduction of the bulkier substituents via acylation resulted in completely inactive **17f**–**19f**. Corresponding precursors **14f**–**16f** were inactive as well.

The characteristic feature of the group 2 compounds was the formation of the cation–π interaction between the protonated basic nitrogen of the linker and the phenyl ring of Y118 (Figure 6c–h). The linker of **7e** was extended by the 4-aminopiperidinyl fragment as compared to amide **6a**. The p-chlorophenyl moiety was positioned favorably to engage Y64 in the stacking interaction and form additional hydrophobic contact with L87. The lower potency of **7e** (24 h MIC 4 µg/mL) as compared to **6a** can be related to the increased flexibility of the longer linker, resulting in an additional entropic loss upon binding. N-methylation of **7e** (compound **13e**) resulted in significant MIC improvement (from 4 to 0.125 µg/mL), likely attributed to the optimal positioning of the methyl group in the hydrophobic cavity formed by the sidechains of Y118, Y132, T122, and L121, as well as to a higher conformational rigidity of the alkylated linker (Figure 6c). The linker of **9g** was further elongated by one methylene group, allowing hydrogen bond formation between the TZD carbonyl and Y64 OH. The p-chlorophenyl moiety was exposed to the solvent and positioned on the lipophilic surface formed by F58, A61, and L88. N-methylation of **9g** resulted in the more active **13g** (24 h MIC 0.125 and 0.06 µg/mL for **9g** and **13g,** respectively), allowing the fit of the methyl group in the hydrophobic cavity of the binding site similar to that for **13e** (Figure 6d). Formation of an additional hydrogen bond was likely the key factor that provided the gain in potency for N-methylated **13g** over **13e** and for desmethyl **9g** over **7e**.

Replacing the oxoethyl fragment connecting the piperidyl ring with TZD in **7e** with the longer 2-hydroxypropyl insert resulted in **31**. As in the case of **9g**, linker elongation by one methylene group allowed formation of the hydrogen bond between the TZD carbonyl and Y64 OH, accompanied by the potency improvement. (*R*)-configuration of the stereocenter in the linker was favorable for binding (Figure 6e). The bound conformation was stabilized by the intramolecular hydrogen bond between the linker’s OH and TZD carbonyl. N-methylation of **31** (compound **32**) did not result in potency improvement, presumably due to the different positioning of the N-methyl group in the binding pocket as compared to **13g**/**13e** (Figure 6f). (*S*)-configuration of the stereocenter in the linker was preferred for binding in the case of **32**, whereas an intramolecular polar bond in the linker was formed between the hydroxyl group and the unprotonated piperidyl N. The higher potency of less lipophilic 2-hydroxypropyls **31** and **32** (24 h MIC 0.0125 µg/mL for both **31** and **32**) as compared to **9g**/**13g** with a similar length of the linker can in part be attributed to stronger cation–π interactions manifested in shorter distances between the basic nitrogen of the ligand and the Y118 phenyl ring (Supplementary Table S2).

Piperazine **28** featured the linker being roughly one methylene group shorter as compared to **31**/**32**. The TZD fragment was shifted deeper inside the substrate access channel, preventing interaction between TZD carbonyl and Y64 OH (Figure 6g). Instead, a new hydrogen bond was formed between the linker’s OH and S378 backbone carbonyl, stabilizing the bent conformation of the propyl chain in the ligand-bound state. TZD position matched that of **13e** (Figure 6c,g), engaging Y64 in the stacking interaction. (*R*)-configuration of the stereocenter in the linker was preferable for binding. The population of the protonation state of **28** capable of forming cation–π interaction with Y118 is significantly attenuated at the physiological pH as compared to **31**/**32**. This decreases the impact of the cation–π interaction on the potency of **28**.

Both **34** and **35** with the bulky 1,4-thiazepan-3-on ring likely require additional induced-fit of the receptor for optimal binding. The sidechain arm of **34** did not form a hydrogen bond with the residues of the substrate access channel, whereas the position of the p-chlorophenyl moiety resembled that in **31**/**32**. Compound **35** did not fit within the binding site in the docking experiment with the rigid receptor.

### 2.5. SCRAPPY-Based Cytometric Proteome Profiling of the Cellular Response

**Figure 7:**
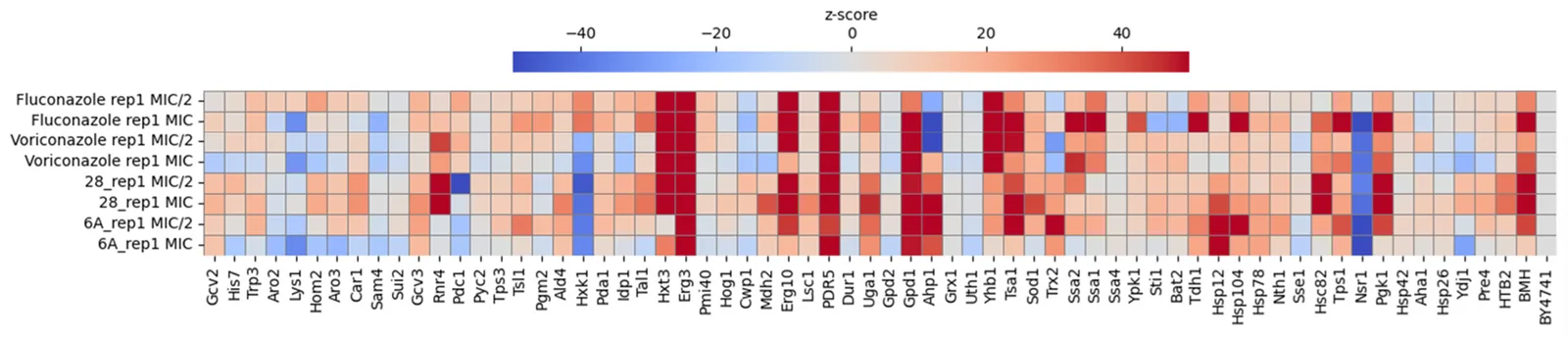
Effects of compounds **6a**, **28** and reference azoles (fluconazole, voriconazole) on the abundance of GFP-fusion proteins in *S. cerevisiae* (SCRAPPY analysis). Heatmap showing z-scores of GFP fluorescence for individual proteins. Red indicates increased abundance; blue indicates decreased abundance. Concentrations: compound **6a** MIC—50 μg/mL (0.5 × MIC = 25), compound **28** MIC—50 μg/mL (0.5 × MIC = 25), fluconazole—15 μg/mL (0.5 × MIC = 7.5), voriconazole—0.04 μg/mL (0.5 × MIC = 0.02). Each concentration was tested in duplicate. Azole data were obtained under the same conditions [39].

To further characterize the mechanism of action, we performed SCRAPPY analysis (GFP-fusion protein abundance screening) [38] for compounds **6a** and **28** in comparison with reference azoles (fluconazole and voriconazole) [39]. As shown in Figure 7, the heatmap profiles of **6a** and **28** are highly similar to those of the azoles. Notably, both compounds induced strong upregulation of Erg3 and Erg10–key enzymes in the ergosterol biosynthesis pathway, as well as reference azoles. This transcriptional signature is characteristic of ergosterol biosynthesis inhibitors. In addition, we observed a comparable increase in the abundance of Pdr5, a well-known multidrug efflux pump associated with azole resistance. The upregulation of Pdr5 by **6a** and **28** was similar in magnitude to that caused by the reference azoles, suggesting that these compounds, like azoles, are recognized by the cellular efflux machinery. Overexpression of ABC transporters such as Pdr5 (and its homolog Cdr1 in *Candida albicans*) is one of the primary mechanisms by which fungal pathogens acquire resistance to azole antifungals [40]. Furthermore, both **6a** and **28** strongly increased the abundance of Gpd1 (glycerol-3-phosphate dehydrogenase), a key enzyme in glycerol biosynthesis that is typically upregulated in response to osmotic and oxidative stress [41,42]. For azoles, which inhibit ergosterol biosynthesis, this response is triggered by the resulting membrane damage and subsequent osmotic imbalance.

Taken together, the SCRAPPY profiles reveal that compounds **6a** and **28** induce a response highly similar to that of reference azoles: upregulation of ergosterol biosynthesis genes (Erg3, Erg10), increased abundance of the efflux pump Pdr5, and elevation of the stress marker Gpd1. This coordinated response strongly suggests that **6a** and **28**, like azoles, perturb sterol homeostasis and trigger a comparable cellular stress program.

### 2.6. Phenotypic Microscopy of Yeast Cells with Compound ***6a*** and ***28***

Compound **28** was selected for additional cell-based phenotypic evaluation as a representative active compound from the 2-hydroxypropyl-linked triazole-thiazolidine series. This compound showed low MIC values against *C. parapsilosis* isolates and retained measurable activity against the tested azole-resistant *C. albicans* strain. Compound **6a**, an amide-linked analogue that also displayed pronounced antifungal activity in the primary screening panel, was included as a comparator to determine whether structurally distinct active hybrids produce similar yeast-cell phenotypes. Therefore, compounds **6a** and **28** were examined by fluorescence microscopy to assess whether their antifungal activity is accompanied by cellular changes consistent with perturbation of sterol-associated yeast physiology.

**Figure 8:**
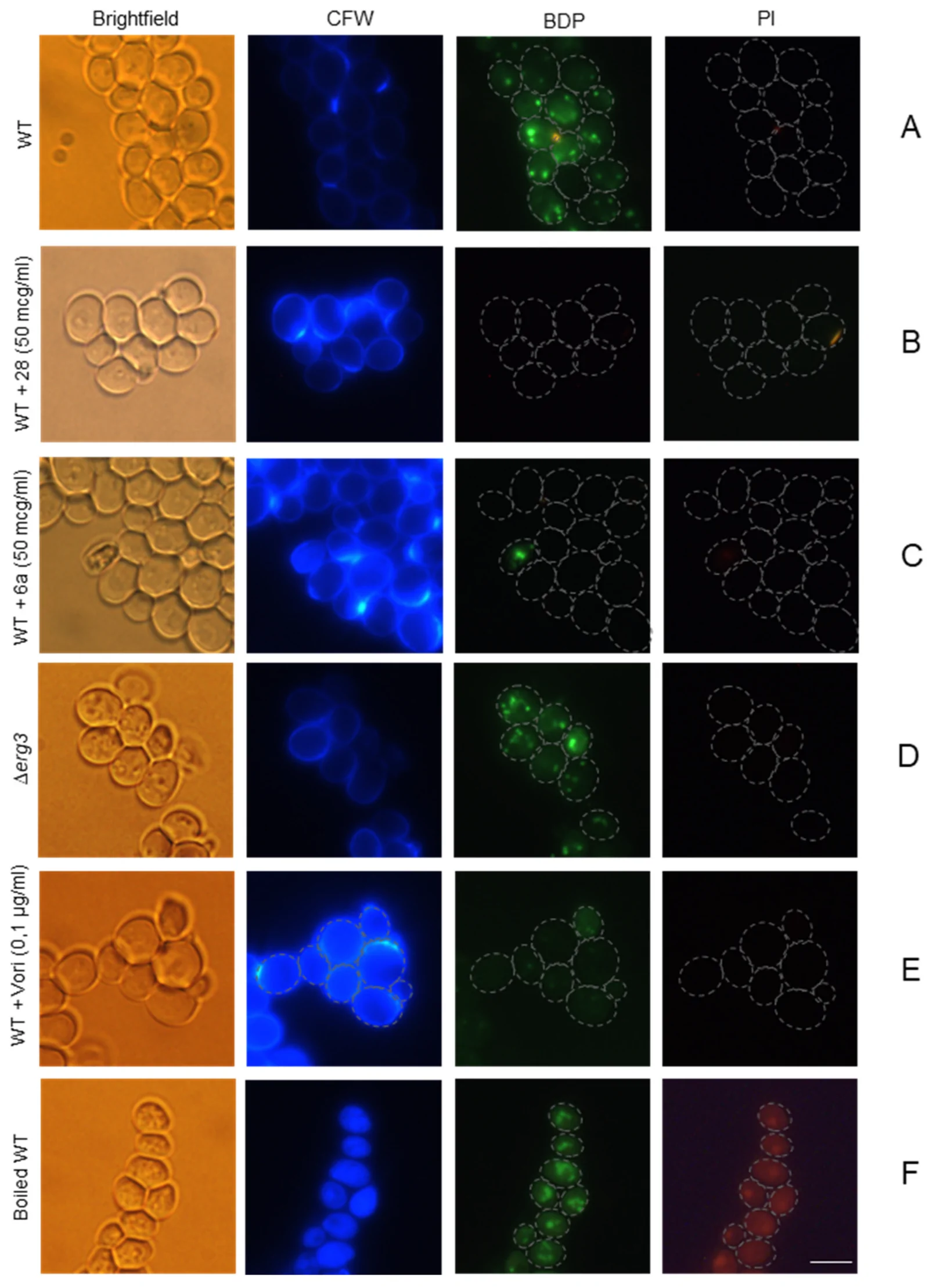
Compounds **28** and **6a** induce cell wall stress and lipid droplet exhaustion in *S. cerevisiae*. Representative bright-field and fluorescence microscopy images of: (**A**) untreated wild-type (WT) cells (**B**) WT cells treated with compound **28** (50 μg/mL) (**C**) WT cells treated with compound **6a** (50 μg/mL) (**D**) untreated *Δerg3* cells (**F**) WT cells treated with voriconazole (0.1 μg/mL) and (**E**) heat-killed WT cells used as a positive control for membrane permeabilization. Scale bar, 5 μm.

To assess cellular phenotypes, log-phase *S. cerevisiae* BY4742 cells were treated with compounds **6a** or **28** (50 µg/mL, corresponding to the MIC determined for both compounds, Figure S4) or with voriconazole (0.1 µg/mL) for 4 h, stained with Calcofluor White (CFW), BDP, and propidium iodide (PI), and examined by fluorescence microscopy (Figure 8). Untreated WT, *Δerg3*, and heat-killed cells served as controls.

Cells were treated for 4 h with 50 µg/mL of each compound, or with voriconazole (0.1 µg/mL), and compared to untreated WT, *Δerg3*, and heat-killed controls. Staining: BDP (lipids), PI (membrane integrity), and Calcofluor White (cell wall chitin). Both compounds caused strong CFW staining and loss of BDP-positive lipid droplets, a phenotype identical to that induced by voriconazole and distinct from that of the *Δerg3* mutant. PI uptake was observed only in heat-killed cells, indicating that the plasma membrane remained intact under all other tested conditions.

After 4 h of exposure, both compounds and voriconazole induced markedly stronger CFW staining in WT cells compared with untreated controls (Figure 8A–C,F). In contrast, PI uptake was not detected in compound-treated cells, voriconazole-treated cells, or untreated mutants, and was confined to the heat-killed control, indicating that the plasma membrane remained intact during the 4 h treatment period. BDP staining revealed abundant neutral lipid droplets in untreated WT cells, whereas cells treated with either compound showed a pronounced reduction of BDP-positive signals (Figure 8A–C,F). Voriconazole-treated cells exhibited a similar but less pronounced decrease of BDP staining. Heat-killed cells retained BDP staining despite complete loss of cellular morphology, ruling out artefactual lipid degradation.

To validate target specificity, we employed the *Δerg3* mutant as an epistatic control. This strain lacks the C5-sterol desaturase, operates downstream of the CYP51-dependent step, and is well-characterized for its resistance to azole antifungals. If compounds **6a** and **28** acted as non-specific membrane disruptors or inhibited a step downstream of Erg3, *Δerg3* would phenocopy treated WT cells. However, *Δerg3* retained BDP staining and showed only weak CFW fluorescence, closely resembling untreated WT (Figure 8D,E). This differential response, together with the phenotypic match to voriconazole-treated WT cells, confirms that the observed effects are specifically associated with perturbation of sterol biosynthesis rather than with general pathway disruption.

When incubation was extended to 16 h at 50 µg/mL (1 × MIC) and 100 µg/mL (2 × MIC), compound **28**-treated cells showed increased PI uptake, stronger CFW signal, reduced cell size, loss of rounded morphology, and granular cytoplasm (Supplementary Figure S5). These late changes suggest secondary loss of membrane integrity and cell wall damage upon prolonged exposure.

### 2.7. Cytotoxicity Evaluation of Compound ***28***

**Figure 9:**
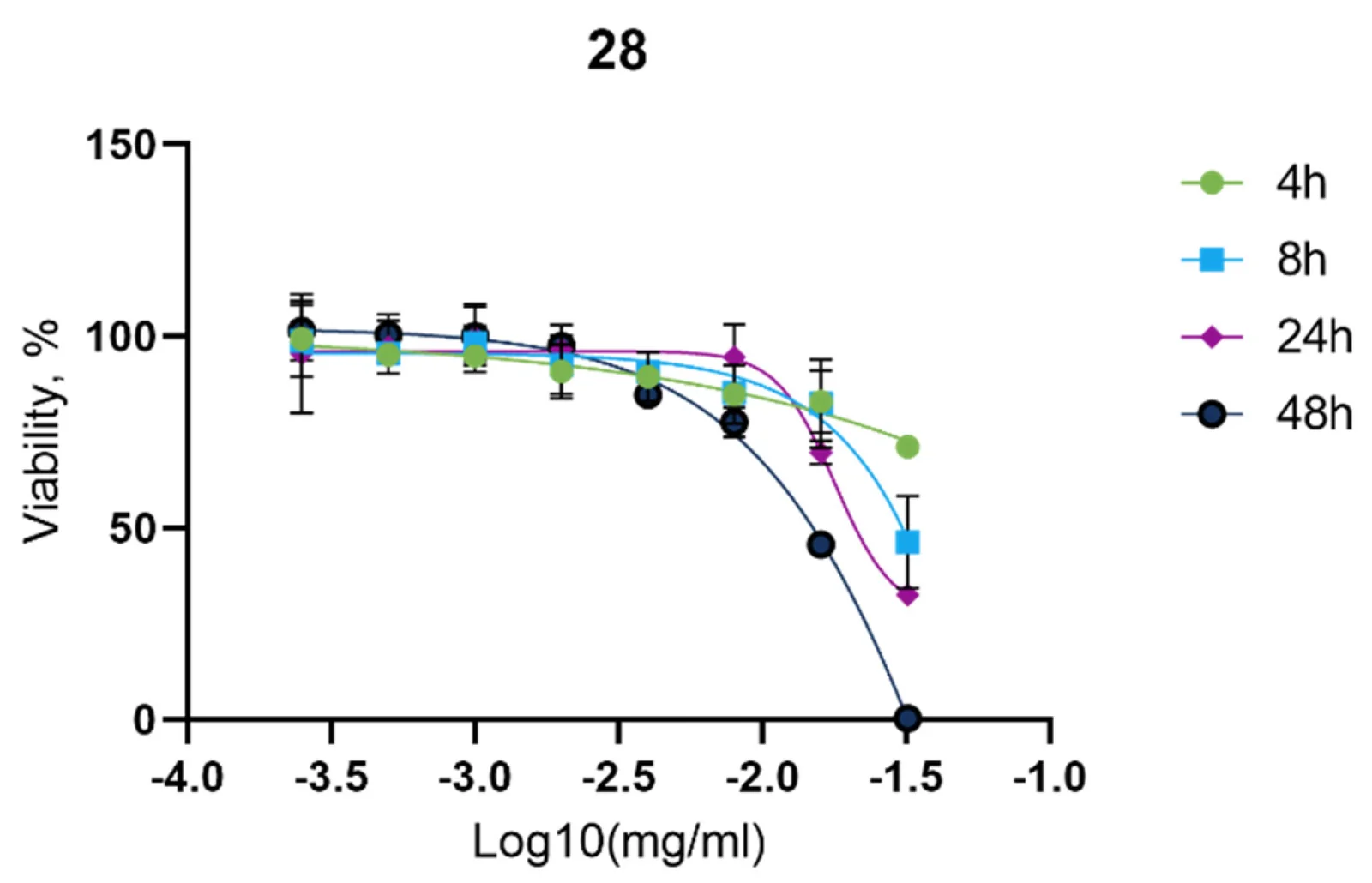
Time-dependent cytotoxicity of Hep-G2 cells treated with compound **28**. Cell viability after 4, 8, 24, and 48 h of exposure to compound **28**, determined by the MTT assay.

To further characterize the cytotoxicity profile of compound **28**, human hepatocellular carcinoma cell line Hep-G2 viability was evaluated by the MTT assay after 4, 8, 24, and 48 h of exposure (Figure 9). No CC_50_ value could be determined after 4 or 8 h within the tested concentration range, indicating that compound **28** did not produce measurable acute cytotoxicity at early time points. Detectable cytotoxicity appeared only after prolonged incubation, with CC_50_ values of 18.2 ± 3.4 μg/mL after 24 h and 14.9 ± 1.2 μg/mL after 48 h.

In addition to the MTT assay, the cytotoxicity of compound **28** was independently assessed using an ATP-dependent luminescent CellTiter-Glo viability assay in Hep-G2 cells and normal human peripheral blood mononuclear cells (PBMC). Compound **28** was tested over a concentration range of 1 nM to 100 μM. The obtained CC_50_ values were 13.4 μM (8.28 μg/mL) for Hep-G2 cells and 14.1 μM (8.71 μg/mL) for PBMC after 24 h. These values were in the same concentration range as those determined by the MTT assay, supporting the consistency of the cytotoxicity assessment across two independent viability readouts.

These values are substantially higher than the MICs observed for the most susceptible *Candida* isolates, including *C. parapsilosis*, for which compound **28** was active in the sub-μg/mL range. Thus, the antifungal activity of compound **28** is achieved at concentrations below those associated with cytotoxicity in the MTT assay. However, the decrease in CC_50_ after 48 h indicates time-dependent mammalian-cell toxicity, which should be taken into account during further structural optimization.

Phase-contrast microscopy was used to complement the MTT data and assess cell morphology after treatment with compound **28** (Supplementary Figure S6). DMSO-treated control cells retained typical adherent morphology throughout the observation period. At 8 μg/mL, compound **28** caused no pronounced morphological alterations, and the cell layer remained largely comparable to the control. At 16 μg/mL, moderate changes in cell morphology and reduced cell density became visible, particularly after 24–48 h. At 32 μg/mL, compound **28** induced marked cell rounding, loss of spreading, and detachment, with the effect becoming more pronounced over time. Overall, the microscopy data were consistent with the MTT assay and confirmed that cytotoxic effects of compound **28** are concentration- and time-dependent.

### 2.8. In Vivo Acute Toxicity of Compound ***28***

Compound **28** was selected for a preliminary acute oral toxicity assessment based on its in vitro antifungal activity and cytotoxicity profile. The study was performed in male and female mice after a single oral administration of compound **28** at doses of 300 and 2000 mg/kg.

At the dose of 2000 mg/kg, compound **28** caused mortality in animals of both sexes, with death observed in 2 of 3 males and 3 of 3 females. This result indicates pronounced toxicity at high acute exposure. In contrast, no mortality was observed after administration of compound **28** at 300 mg/kg during the 14-day observation period.

At 300 mg/kg, treated animals did not show visible clinical signs of intoxication during the early post-administration period or throughout the subsequent observation period. Body-weight dynamics were not markedly affected in either males or females. A moderate decrease in food and water consumption was observed, more pronounced in females, suggesting a transient effect on general physiological status. Gross macroscopic examination did not reveal visible morphological alterations of major internal organs in animals treated with 300 mg/kg compound **28**. These data indicate that compound **28** was tolerated at a single oral dose of 300 mg/kg under the conditions of this preliminary screening. These results should be interpreted only as an initial acute toxicity assessment and do not establish a complete in vivo safety profile.

### 2.9. Metabolic Stability and CYP450 Interaction

The metabolic stability of compounds **28** and **35** was evaluated in human liver microsomes in the presence of NADPH. The contributions of the major cytochrome P450 isoforms CYP1A2, CYP2C9, CYP2C19, CYP2D6, and CYP3A4 to the metabolism of the compounds were also assessed, together with their ability to inhibit key human CYP isoforms.

Racemic compound **28** was rapidly metabolized in human liver microsomes, with a half-life of 3.92 min. CYP phenotyping indicated that its metabolism was mediated predominantly by CYP3A4, whereas no appreciable contribution from CYP1A2, CYP2C9, CYP2C19, or CYP2D6 was detected. These findings identify rapid CYP3A4-dependent metabolism as a major liability of compound **28** and an important objective for further lead optimization.

Because the metabolites and exact metabolic soft spots of compound **28** have not yet been identified, specific structural modifications remain provisional. Potential optimization strategies include steric or electronic shielding of oxidation-prone positions, replacement of potentially labile groups within the hydroxypropyl–piperazine linker with metabolically more stable bioisosteres, and modulation of linker flexibility and lipophilicity while preserving the structural features associated with antifungal activity in this series. Because compound **28** was evaluated as a racemate, resolution and separate assessment of its stereoisomers may also be informative, as stereochemistry can influence both CYP-mediated metabolism and antifungal activity. Metabolite identification and experimental mapping of the metabolic soft spots will therefore be required to guide the rational design of subsequent analogues.

In contrast, compound **35** was highly stable in human liver microsomes, with no substantial metabolism detected over at least 60 min. However, it strongly inhibited CYP3A4 (IC_50_ = 0.11–0.18 μM) and CYP2C9 (IC_50_ = 0.41 μM) and moderately inhibited CYP2C19 (IC_50_ = 2.84 μM). Partial inhibition of CYP2C8 and CYP2D6 was observed, with enzyme activity reduced by 55–65% at 10 μM, whereas the effect on CYP1A2 was minimal, with an activity decrease of no more than 20%.

Thus, despite its high microsomal stability, compound **35** shows a pronounced propensity to inhibit several clinically relevant CYP isoforms, indicating a substantial potential for drug–drug interactions and reducing its attractiveness for further development in its current form. Compound **28**, by contrast, combines potent antifungal activity with preliminary acute oral tolerability at 300 mg/kg and therefore represents a promising exploratory lead for further medicinal chemistry optimization. Its rapid CYP3A4-mediated metabolism remains a major limitation but may be addressed through targeted structural modification guided by metabolite identification and metabolic soft-spot analysis.

## 3. Materials and Methods

### 3.1. Molecular Modelling

Molecular modelling of the protein-ligand interactions was performed as described previously [15]. In brief, the crystallographic structure of CYP51 from *C. albicans* (PDB ID 5TZ1) was used as a receptor model. The crystallographic water molecule bound in the active site was retained. Docking was done using DOCK 6.9 [43] followed by analysis of obtained binding modes in ChimeraX 1.10.1 [44], PyMol 1.8 (Schrodinger LLC, New York, NY, USA), and AuPosSOM 2.1 [45]. The cLogP descriptor was calculated using DataWarrior 6.5.1 [46].

### 3.2. In Vitro Antifungal Susceptibility Testing and MIC Determination

The antifungal activity of the synthesized hybrid compounds was evaluated in vitro by the broth microdilution method using RPMI 1640 medium (with L-glutamine and 0.2% glucose) buffered to pH 7.0 with MOPS, in accordance with ISO 16256:2012 for yeasts and the CLSI reference procedures for susceptibility testing of yeasts (M27) and filamentous fungi (M38). Fluconazole, voriconazole, and posaconazole were used as reference antifungal agents (itraconazole was additionally included for the extended *Candida* isolate panel). Test compounds were dissolved in DMSO to obtain stock solutions (10 mg/mL). Working solutions were prepared in RPMI 1640 and subjected to twofold serial dilutions in 96-well plates to yield final test concentrations in the range 32.0–0.015 μg/mL (growth control and sterility control included). Standardized fungal inoculum was prepared according to the corresponding guidelines and added to each well. Plates were incubated at 35 °C and visually evaluated for fungal growth.

MIC values were defined as the lowest compound concentration causing complete growth inhibition relative to the growth control. MICs were recorded after 24 and 48 h for *Candida* spp. and after 48 h of incubation for *Aspergillus fumigatus*. MIC values were determined in three independent biological experiments. Because MICs are discrete endpoints obtained from twofold serial dilution series, arithmetic means and standard deviations were not calculated. When all three experiments gave the same endpoint, that MIC is reported. When the endpoints differed by one dilution, the observed range is shown.

The primary reference strain panel included: *Candida parapsilosis* ATCC 22019, *Candida albicans* 604M, *Candida krusei* 432M, *Candida auris* VGNKI, and *Aspergillus fumigatus* ATCC 46645. For selected compounds (groups 3–4), testing was expanded to additional *Candida* isolates: *C. parapsilosis* 58L, *C. albicans* 8(R), *C. tropicalis* 3019, *C. glabrata* 61L, and *C. utilis* 84, *M. canis* B-200, *T. rubrum* U.2002, and *Cryptococcus neoformans.*

### 3.3. SCRAPPY Analysis

SCRAPPY analysis was performed as described previously [38]. Briefly, a panel of 64 *S. cerevisiae* BY4741 strains expressing GFP-tagged proteins was treated with compounds at 0.5 × MIC and MIC for 6 h. Propidium iodide (1 μg/mL) was added 1 h prior to the end of incubation to stain dead cells. GFP fluorescence and propidium iodide staining were measured using a Cytoflex S flow cytometer (Beckman Coulter, Brea, CA, USA) equipped with a 96-well sampler. GFP was assayed with a 488 nm laser and 525/40 FITC filter; propidium iodide was assayed with a 532 nm laser and 585/42 PE filter. At least 10,000 events were acquired per sample. Dead cells (PE-A > 5000) were excluded from the analysis. Protein abundance was calculated as median GFP fluorescence normalized to cell size (FITC-A/FSC-A) with autofluorescence subtraction. The data were then normalized to untreated controls, and z-scores were calculated for each protein across all conditions. Data analysis was performed using custom Python scripts (https://github.com/fedorrik/scrapper, accessed on 1 August 2026); heatmaps were generated using the same scripts.

### 3.4. Fluorescence Microscopy

Log-phase *S. cerevisiae* BY4742 cells were obtained by diluting an overnight culture in fresh YPD medium and incubating for 4 h at 30 °C with shaking until the culture reached mid-log phase. Cells were then either left untreated or incubated with compounds 28 or 6a (50 µg/mL) or with voriconazole (0.1 µg/mL) for an additional 4 h at 30 °C. Untreated wild-type (WT) and *Δerg3* strains served as phenotypic controls; heat-killed WT cells (boiled for 5 min) were included as a membrane-damage control. The *Δerg3* deletion mutant used in this study was obtained from the Yeast Gene Deletion Collection [47].

After treatment, cells were stained with the following fluorescent probes: BDP^®^493/503 (Lumiprobe, Hunt Valley, MD, USA; 2 µM) for neutral lipid droplets, propidium iodide (Sigma, St. Louis, MO, USA, 2 µg/mL) for plasma membrane integrity, and Calcofluor White (Sigma, 1 mM) for chitin in the cell wall. All staining was performed for 15 min at room temperature in the dark, followed by two washes with YNB medium to remove excess dye while maintaining osmotic balance. Cells were then resuspended in YNB and mounted on an agarose pad prepared with YNB medium [48].

Fluorescence microscopy was performed using an Axioskop 40 fluorescence microscope (Zeiss, Oberkochen, Germany) equipped with a 100× oil-immersion objective (numerical aperture 1.2) and a Q-Imaging camera (Micropublisher, Surrey, BC, Canada). A mercury short-arc lamp HBO 50 W/AC (Osram, Munich, Germany) served as the light source. The following excitation/emission filter sets were used: 360/425 nm for Calcofluor White, 470/520 nm for BDP^®^ 493/503, and 546/590 nm for propidium iodide. Images were acquired in both brightfield and fluorescence channels.

### 3.5. Cytotoxicity Assay

Cytotoxicity was assessed in human hepatocellular carcinoma cells Hep-G2 (ATCC^®^ HB-8065™) and normal human peripheral blood mononuclear cells (PBMC) using an ATP-dependent luminescent viability readout (CellTiter-Glo^®^). Cells were maintained at 37 °C in a humidified atmosphere (5% CO_2_) in DMEM supplemented with 10% fetal bovine serum and 1% antibiotic/antimycotic mixture. For the assay, Hep-G2 or PBMC were prepared in complete DMEM at 2.5 × 10^4^ cells/mL and dispensed into white 384-well plates (40 µL per well; 1000 cells/well). Plates were centrifuged for 1 min at 50 *g* and incubated for 16–24 h (37 °C, 5% CO_2_). Test compounds were prepared as 10 mM stocks in DMSO, serially diluted, and added to cells to obtain ten final concentrations from 1 nM to 100 µM (two technical replicates per concentration). Plates were centrifuged for 1 min at 100 *g* and incubated for 24 h. Negative control wells contained medium without cells (0% viability), and positive control wells contained cells with vehicle (DMSO) without compounds (100% viability); staurosporine was used as a cytotoxic control. After incubation, CellTiter-Glo^®^ reagent (15 µL/well; equilibrated to room temperature) was added, plates were gently mixed, and luminescence was recorded 5 min later using a microplate reader (Varioskan Flash, Thermo Scientific, Waltham, MA, USA). Percent viability was calculated relative to the vehicle and no-cell controls, and CC_50_ values were obtained by fitting concentration–response curves in GraphPad Prism 5.

Cytotoxicity MTT assay was carried out on the Janus (Perkin) automatic station. Hep-G2 cell suspension in 140 μL of DMEM/F12 media was seeded in a 96-well plate. Cells were incubated at 37°C in 5% CO_2_ for 24 h. The compounds were dissolved in an DMSO aliquot to get a concentration of 32 mg/mL and then were diluted with the media solution immediately before the experiment. Maximum compound concentration was 32 μg/mL, and DMSO volume concentration did not exceed 1%. Cell viability was determined by MTT assay after 4, 8, 24, and 48 h of incubation. The cells were treated with the MTT reagent (Paneco LLC, Moscow, Russia) solution (0.5 g/L) and were then incubated for 2 h at 37°C in a CO_2_ incubator. Then, the MTT solution media was removed, and 140 μL of DMSO was added; the cells were additionally incubated for at least 15 min at room temperature with the use of an orbital shaker in order to dissolve formazan produced from the MTT reagent reduction. Formazan absorption was detected at λ = 545 nm with a tablet-reader VICTOR X5. A nonlinear regression model, in which four parameters were measured (lower limit, upper limit, slope, and CC_50_), was found for each compound with the software GraphPad Prism 9.

### 3.6. In Vivo Acute Oral Toxicity (Mice)

Acute oral toxicity was evaluated in CD-1 mice (male and female, >6 weeks old) in accordance with GOST 32644-2014 (OECD Test Guideline 423 [49]) under GLP-compliant conditions. Animals were housed under controlled environmental conditions (20–25 °C; 30–70% humidity; 10–15 air changes/hour; light/dark cycle) with food and water available ad libitum. The test compound was formulated on the day of dosing in 0.5% (*w*/*v*) low-viscosity carboxymethylcellulose (CMC) in drinking water and administered by oral gavage at 10 mL/kg. A limit dose of 2000 mg/kg was first administered to 3 males and 3 females; based on the outcome, an additional group received 300 mg/kg (3 males and 3 females), with a vehicle control group included. Animals were monitored for 14 days for mortality and clinical signs; body weight as well as food and water consumption were recorded. At the end of the observation period, surviving animals were euthanized by CO_2_ inhalation followed by necropsy and macroscopic examination of internal organs.

### 3.7. Human Liver Microsomal Stability and CYP450 Interaction

#### 3.7.1. Microsomal Metabolic Stability

Metabolic stability was assessed in pooled human liver microsomes (XenoTech, catalog N0610, Kansas City, KS, USA) in the presence of NADPH. Test compounds (final 1 µM) were incubated with human liver microsomes (final protein 0.25 mg/mL) in 50 mM phosphate buffer containing MgCl_2_ (3.3 mM) at 37 °C with shaking (400 rpm). Reactions were initiated by adding NADPH (final 1 mM), and aliquots were sampled at 0, 5, 10, 20, 30, and 60 min. Samples were quenched with acetonitrile containing an internal standard (tolbutamide, 200 ng/mL), proteins were precipitated (30 min at +4 °C), and samples were centrifuged (10 min, 2500 *g*). Supernatants were analyzed by LC–MS/MS. Half-life (t_1/2_), in vitro intrinsic clearance (Clint), and percent remaining were calculated from depletion kinetics; verapamil was used as a reference control compound.

#### 3.7.2. CYP Phenotyping (Major Isoform Contribution)

To estimate the contribution of major CYP isoforms to compound metabolism, incubations in human liver microsomes were performed in the presence of selective chemical inhibitors (furafylline for CYP1A2, sulfaphenazole for CYP2C9, omeprazole for CYP2C19, quinidine for CYP2D6, ketoconazole for CYP3A4). Depletion of the parent compound over time (0–60 min) was compared between inhibitor conditions and no-inhibitor controls; reactions were initiated with NADPH and quenched/processed as described above.

#### 3.7.3. CYP Inhibition Assay (IC_50_ Determination)

Inhibition of CYP isoforms was studied in human liver microsomes using a cocktail of probe substrates: phenacetin (CYP1A2), tolbutamide (CYP2C9), S-mephenytoin (CYP2C19), dextromethorphan (CYP2D6), amodiaquine (CYP2C8), and two CYP3A4 substrates (midazolam and testosterone). Test compounds were prepared as 10 mM stocks in DMSO and tested in a 6-point serial dilution (final concentration range 10 to 0.0098 µM). After pre-incubation (10 min, 37 °C, 400 rpm), reactions were started by addition of an NADPH-containing cofactor mix and incubated for 20 min (37 °C, 400 rpm). Reactions were stopped by adding cold acetonitrile containing propranolol (50 ng/mL), followed by cooling (−20 °C, 10 min) and centrifugation (2500 *g*, 10 min). Metabolites were quantified by LC-MS/MS using calibration curves (Analyst 1.5.2), percent inhibition was calculated from “min/max” controls, and IC_50_ values were obtained by sigmoidal regression in GraphPad Prism 5.

#### 3.7.4. Chemistry General Information

All reagents, salts and solvents were of highest purity and used as supplied by Macklin (Shanghai, China), AmBeed (Buffalo Grove, IL, USA), Aldrich (St. Louis, MO, USA) and Acros (Geel, Belgium). Purity of the compounds was checked by thin layer chromatography using silica-gel 60 F254-coated Al plates (Merck, Darmstadt, Germany), and spots were observed under UV light and ninhydrin test. ^1^H NMR spectra were recorded on a Bruker AVANCE 600 spectrometer (600.13 MHz), a Bruker Avance 700 MHz NMR spectrometer equipped with a Zaxis-gradient 5 mm HCN Prodigy cryoprobe, and a Varian VXR-400 spectrometer (400 MHz, Varian, Berkeley, CA, USA) at 25°C temperature in CDCl_3_ or DMSO-d_6_. Chemical shifts for ^1^H NMR were reported as δ values, and coupling constants were in Hertz (Hz). The following abbreviations were used for spin multiplicity: s = singlet, d = doublet, t = triplet, dd = double doublet, m = multiplet, bs = broad singlet. The chemical shifts are reported in parts per million (ppm) using DMSO-d_6_ or CDCl_3_ as an internal standard; the coupling constants are expressed in Hz. All spectra were processed with MestReNova 14.2.2. The mass-spectral measurements were carried out by the ESI method on micrOTOF-QII (Brucker Daltonics GmbH, Bremen, Germany). Analytical HPLC was performed on a Shimadzu LC-20AD system using a Kromasil-100-5-C18 (Akzo-Nobel, Amsterdam, The Netherlands) column, 4.6 × 250 mm, temperature 20 °C, UV detection, mobile phase A—0.2% HCOONH_4_, mobile phase B-MeCN, (pH 7.4), fl-1 mL/min. LC/MS was performed on Agilent 1100 LC/MSD (Agilent Technologies, Santa Clara, CA, USA), MSD Ionization Source: APCI or ESI, detectors: ELSD, SEDEX 85, DAD (UV) 200–400 nm, TWC (Total Wave Chromatogram). The ^1^H and ^13^C NMR spectra, ESI-MS spectra, and HPLC chromatograms for the synthesized compounds are pro-vided in the Supplementary Materials (Figures S7–S131, Table S3).

##### General Procedure **A** for the Synthesis of Compounds **2b**–**2g**, **9g**

To a solution of compound **1** (1.0 mmol) in alcohol (10 mL) was added the corresponding amine (1.2 mmol), followed by triethylamine (3.0 mmol). The reaction mixture was refluxed at 80 °C for 6 h. Reaction progress was monitored by thin-layer chromatography (TLC) using dichloromethane/methanol (20:1, *v*/*v*) as the eluent. Upon completion of the reaction, the solvent was removed under reduced pressure. The residue was purified by silica gel column chromatography (SiO_2_, dichloromethane/methanol 25:1, *v*/*v*) to afford compounds **1a**–**1c** as purified products.

##### General Procedure **B** for the Synthesis of Compounds **4e**–**4g**, **11**, **13e**–**g**, **17**–**19f**, **29**

To a solution of Boc-protected amine (1.0 mmol) in dichloromethane (10 mL) was added a 4 M solution of hydrogen chloride in dioxane (3 mL). The reaction mixture was stirred at 25 °C for 3 h. Completion of the reaction was confirmed by TLC. The reaction mixture was diluted with toluene (5 mL) and concentrated under reduced pressure. The resulting residue was suspended in diethyl ether (10 mL) and sonicated for 5 min. The ether layer was decanted, and the solid residue was dried under reduced pressure for 1 h to afford compounds **4e**–**4g**, **11**, as their corresponding hydrochloride salts in quantitative yield.

##### General Procedure **C** for the Synthesis of Compounds **6a**–**6d**, **7e**, **8f**, **10**, **11**, **13e**–**g**, **17**–**19f**

To a solution of the corresponding amine (1.0 mmol, 1.0 equiv) in dry dichloromethane (10 mL) was added triethylamine (2.0 mmol, 2.0 equiv) under stirring at room temperature. After 10 min, the appropriate TZD-containing acyl chloride (1.0 mmol, 1.0 equiv) was added, and the reaction mixture was stirred at room temperature overnight. Reaction progress was monitored by TLC. Upon completion, the solvent was removed under reduced pressure, and the residue was dissolved in dichloromethane. The organic solution was washed with an aqueous 1 M HCl solution, followed by washing with water and saturated aqueous sodium chloride solution. The organic phase was dried over anhydrous sodium sulfate, filtered, and concentrated under reduced pressure. The crude product was purified by flash column chromatography on silica gel using hexane/ethyl acetate (1:4, *v*/*v*) or dichloromethane/methanol (25:1, *v*/*v*) as the eluent to afford compounds **6a**–**6d**, **7e**, **8f**, **10**.

##### (Z)-2-(5-(4-chlorobenzylidene)-2,4-dioxothiazolidin-3-yl)-N-(2-(2,4-difluorophenyl)-2-hydroxy-3-(1H-1,2,4-triazol-1-yl)propyl)acetamide (**6a**)

Compound **6a** was prepared according to General Procedure **C**. White solid; Yield 76%. ^1^H NMR (600 MHz, DMSO) δ = 8.4 (t, *J* = 6.1, 1H), 8.3 (s, 1H), 7.9 (s, 1H), 7.7 (s, 1H), 7.7–7.6 (m, 2H), 7.6–7.6 (m, 2H), 7.4–7.3 (m, 1H), 7.1 (ddd, *J* = 11.9, 9.2, 2.6, 1H), 7.0 (td, *J* = 8.5, 2.6, 1H), 6.0 (s, 1H), 4.6 (d, *J* = 14.4, 1H), 4.5 (d, *J* = 14.4, 1H), 4.3 (d, *J* = 16.5, 1H), 4.2 (d, *J* = 16.5, 1H), 3.7 (dd, *J* = 13.9, 6.2, 1H), 3.6 (dd, *J* = 13.9, 6.0, 1H). ^13^C NMR (151 MHz, DMSO) δ = 166.7, 165.9, 165.1, 161.9 (dd, *J* = 245.9, 12.9), 159.0 (dd, *J* = 247.5, 12.3), 150.6, 144.8, 135.4, 132.0, 131.8, 130.2 (m), 129.5, 124.6–124.2 (m), 121.8, 110.8 (d, *J* = 19.8), 103.9 (t, *J* = 26.9), 74.5 (d, *J* = 5.0), 54.8 (d, *J* = 5.2), 45.8 (d, *J* = 4.4), 43.3. ESI-MS (*m*/*z*): Found mass for C_23_H_18_ClF_2_N_5_O_4_S = 534.1 [M + 1]^+^.

##### (Z)-2-(5-(4-chlorobenzylidene)-2,4-dioxothiazolidin-3-yl)-N-(2-(2,4-difluorophenyl)-2-hydroxy-3-(1H-1,2,4-triazol-1-yl)propyl)-N-methylacetamide (**6b**)

Compound **6b** was prepared according to General Procedure **C**. White solid; Yield 67%. ^1^H NMR (600 MHz, CDCl_3_) δ = 8.0 (s, 1H), 7.9 (s, 1H), 7.8 (s, 1H), 7.6 (td, *J* = 9.0, 6.4, 1H), 7.5–7.4 (m, 4H), 6.9 (td, *J* = 8.3, 2.5, 1H), 6.8 (ddd, *J* = 11.3, 8.4, 2.5, 1H), 5.7 (s, 1H), 4.7 (d, *J* = 14.3, 1H), 4.5 (d, *J* = 15.8, 2H), 4.4 (d, *J* = 16.2, 1H), 4.3 (d, *J* = 14.6, 1H), 3.4 (d, *J* = 14.6, 1H), 3.1 (s, 3H). ^13^C NMR (151 MHz, CDCl_3_) δ = 167.5, 167.4, 165.7, 163.1 (dd, *J* = 250.6, 12.0), 158.7 (dd, *J* = 246.2, 12.1), 151.7, 144.8, 137.0, 133.3, 131.7, 131.5, 131.0–130.3 (m), 129.8, 123.6 (d, *J* = 13.3), 121.9, 112.1 (d, *J* = 20.6), 104.2 (t, *J* = 26.5), 57.0, 55.7, 55.6, 42.6, 37.9. ESI-MS (*m*/*z*): Found mass for C_24_H_20_ClF_2_N_5_O_4_S = 548.0 [M + 1]^+^.

##### (Z)-2-(5-(4-chlorobenzylidene)-2,4-dioxothiazolidin-3-yl)-N-(2-(2,4-difluorophenyl)-2-hydroxy-3-(1H-1,2,4-triazol-1-yl)propyl)-N-propylacetamide (**6c**)

Compound **6c** was prepared according to General Procedure **C**. White solid; Yield 59%. ^1^H NMR (600 MHz, CDCl_3_) δ = 8.0 (s, 1H), 7.8 (s, 1H), 7.8 (s, 1H), 7.6–7.5 (m, 1H), 7.5–7.4 (m, 5H), 6.8 (tt, *J* = 8.2, 4.3, 1H), 6.8 (ddd, *J* = 11.2, 8.4, 2.5, 1H), 5.9 (s, 1H), 4.7 (d, *J* = 14.3, 1H), 4.5 (d, *J* = 14.3, 1H), 4.5 (s, 2H), 4.1–4.1 (m, 1H), 3.5 (d, *J* = 14.6, 1H), 3.3–3.2 (m, 2H), 1.7–1.5 (m, 2H), 0.9 (t, *J* = 7.4, 3H). ^13^C NMR (151 MHz, CDCl_3_) δ = 167.7, 167.3, 165.7, 163.0 (dd, *J* = 250.4, 12.2), 158.7 (dd, *J* = 245.9, 11.8), 151.6, 144.8, 136.9, 133.2, 131.6, 131.4, 131.0–130.3 (m), 129.7, 123.7 (d, *J* = 14.9), 121.9, 112.0 (d, *J* = 20.0), 104.1 (t, *J* = 26.6), 77.0, 55.8 (d, *J* = 6.2), 54.3, 51.5, 42.2, 21.7, 11.2. ESI-MS (*m*/*z*): Found mass for C_26_H_24_ClF_2_N_5_O_4_S = 576.0 [M + 1]^+^.

##### (Z)-2-(5-(4-chlorobenzylidene)-2,4-dioxothiazolidin-3-yl)-N-cyclopropyl-N-(2-(2,4-difluorophenyl)-2-hydroxy-3-(1H-1,2,4-triazol-1-yl)propyl)acetamide (**6d**)

Compound **6d** was prepared according to General Procedure **C**. White solid; Yield 62%. ^1^H NMR (400 MHz, DMSO) δ = 8.4 (s, 1H), 7.9 (s, 1H), 7.8 (s, 1H), 7.7–7.5 (m, 4H), 7.5–7.2 (m, 1H), 7.1 (t, *J* = 10.6, 1H), 7.0–6.8 (m, 1H), 5.8 (s, 1H), 4.8–4.6 (m, 3H), 4.6–4.3 (m, 1H), 4.1 (d, *J* = 14.2, 1H), 3.6 (d, *J* = 14.3, 1H), 2.6 (s, 1H), 1.0–0.9 (m, 1H), 0.9–0.7 (m, 2H), 0.8–0.6 (m, 1H). ^13^C NMR (101 MHz, DMSO) δ = 168.5, 166.4, 164.8, 161.6 (dd, *J* = 289.5, 12.8), 159.1 (dd, *J* = 288.6, 11.9), 135.2, 132.0, 131.6, 129.3, 124.5 (d, *J* = 11.7), 121.5, 110.7 (d, *J* = 20.1), 103.6 (t, *J* = 26.6), 75.2, 55.2, 52.2, 43.2, 30.6, 10.7, 8.7. ESI-MS (*m*/*z*): Found mass for C_26_H_22_ClF_2_N_5_O_4_S = 574.1 [M + 1]^+^.

##### (Z)-5-(4-chlorobenzylidene)-3-(2-(4-((2-(2,4-difluorophenyl)-2-hydroxy-3-(1H-1,2,4-triazol-1-yl)propyl)amino)piperidin-1-yl)-2-oxoethyl)thiazolidine-2,4-dione (**7e**)

Compound **7e** was prepared according to General Procedure **C**. White solid; Yield 59%. ^1^H NMR (600 MHz, CDCl_3_) δ = 7.9 (d, *J* = 5.1, 1H), 7.7 (s, 2H), 7.4 (d, *J* = 8.5, 1H), 7.3–7.2 (m, 4H), 6.7–6.6 (m, 2H), 4.5 (dd, *J* = 14.3, 3.3, 1H), 4.4–4.3 (m, 3H), 4.2–4.1 (m, 1H), 3.6–3.5 (m, 1H), 3.0 (dd, *J* = 15.8, 12.4, 1H), 3.0–2.9 (m, 1H), 2.7 (dd, *J* = 24.9, 12.4, 1H), 2.6–2.5 (m, 1H), 2.5–2.3 (m, 1H), 1.7–1.6 (m, 2H), 1.2–0.9 (m, 2H). ^13^C NMR (151 MHz, CDCl_3_) δ = 167.5, 165.9, 162.9 (dd, *J* = 249.6, 12.4), 162.6, 159.2 (dd, *J* = 248.1, 12.9), 151.5, 144.8, 136.8, 132.9, 131.7, 131.4, 130.0, 129.6, 124.8 (d, *J* = 13.6), 122.1, 111.7 (d, *J* = 20.5), 104.4 (t, *J* = 26.3), 73.5, 55.9, 54.7 (d, *J* = 14.4), 51.7 (d, *J* = 15.5), 43.5, 42.5, 41.2 (d, *J* = 9.2), 33.1, 32.7, 32.3, 32.0.ESI-MS (*m*/*z*): Found mass for C_28_H_27_ClF_2_N_6_O_4_S = 617.0 [M + H]^+^.

##### (Z)-5-(4-chlorobenzylidene)-3-(2-(4-(4-((2-(2,4-difluorophenyl)-2-hydroxy-3-(1H-1,2,4-triazol-1-yl)propyl)amino)phenyl)piperazin-1-yl)-2-oxoethyl)thiazolidine-2,4-dione (**8f**)

Compound **8f** was prepared according to General Procedure **C**. White solid; Yield 59%. ^1^H NMR (600 MHz, DMSO) δ = 8.3 (s, 1H), 8.0 (s, 1H), 7.7 (s, 1H), 7.7 (d, *J* = 8.7, 2H), 7.6 (d, *J* = 8.7, 2H), 7.4 (td, *J* = 9.1, 6.8, 1H), 7.1 (ddd, *J* = 12.0, 9.1, 2.6, 1H), 7.0 (td, *J* = 8.5, 2.6, 1H), 6.8 (d, *J* = 8.4, 2H), 6.6 (d, *J* = 8.4, 2H), 6.0 (s, 1H), 4.9 (t, *J* = 6.7, 1H), 4.7–4.6 (m, 1H), 4.7 (s, 2H), 4.6 (d, *J* = 14.3, 1H), 3.6 (t, *J* = 5.1, 2H), 3.6 (t, *J* = 5.1, 2H), 3.5 (dd, *J* = 13.2, 6.7, 1H), 3.4 (dd, *J* = 13.2, 5.5, 1H), 3.0–3.0 (m, 2H), 2.9–2.8 (m, 2H). ^13^C NMR (151 MHz, DMSO) δ = 166.8, 165.2, 163.0, 161.7 (dd, *J* = 245.7, 12.5), 159.1 (dd, *J* = 246.2, 11.5), 150.5, 144.9, 143.4, 142.4, 135.4, 132.2, 131.8, 131.8, 130.4–130.1 (m), 129.5, 125.5–125.2 (m), 121.8, 118.6, 113.4, 110.8 (d, *J* = 21.2), 103.9 (t, *J* = 27.3), 74.8, 55.2, 51.2, 50.8, 50.3, 44.3, 42.7, 41.9, 40.1. ESI-MS (*m*/*z*): Found mass for C_33_H_30_ClF_2_N_7_O_4_S = 693.3 [M]^+^, 694.3 [M + H]^+^.

##### (Z)-5-(4-chlorobenzylidene)-3-(2-(4-(((2-(2,4-difluorophenyl)-2-hydroxy-3-(1H-1,2,4-triazol-1-yl)propyl)amino)methyl)piperidin-1-yl)-2-oxoethyl)thiazolidine-2,4-dione (**9g**)

Compound **9g** was prepared according to General Procedure **C**. White solid; Yield 82%. ^1^H NMR (600 MHz, MeOD) δ = 8.3 (s, 1H), 7.8 (s, 1H), 7.8 (s, 1H), 7.5 (d, *J* = 8.7, 2H), 7.5–7.4 (m, 3H), 6.9 (dd, *J* = 11.7, 8.8, 1H), 6.8 (t, *J* = 8.4, 1H), 4.7 (q, *J* = 14.4, 2H), 4.6–4.5 (m, 2H), 4.4–4.3 (m, 1H), 3.9–3.8 (m, 1H), 3.1–3.1 (m, 2H), 3.0 (d, *J* = 12.2, 1H), 2.7–2.6 (m, 1H), 2.5–2.3 (m, 2H), 1.8–1.7 (m, 1H), 1.7–1.6 (m, 2H), 1.2–1.1 (m, 1H), 1.0–1.0 (m, 1H). ^13^C NMR (151 MHz, MeOD) δ = 168.5, 167.0, 165.0, 164.1 (dd, *J* = 248.3, 12.2), 160.7 (dd, *J* = 246.9, 12.1), 151.2, 146.0, 137.6, 133.4, 133.2, 132.8, 131.1 (d, *J* = 15.9), 130.6, 126.6 (d, *J* = 9.3), 123.3, 112.0 (d, *J* = 21.0), 105.2–104.6 (m), 75.3, 75.3, 57.2 (d, *J* = 4.9), 56.7, 56.4 (d, *J* = 4.5), 46.1, 43.7, 43.6, 37.2, 30.8. ESI-MS (*m*/*z*): Found mass for C_29_H_29_ClF_2_N_6_O_4_S = 631.0 [M + 1]^+^.

##### (Z)-5-(4-chlorobenzylidene)-3-(2-(4-((2-(2,4-difluorophenyl)-2-hydroxy-3-(1H-1,2,4-triazol-1-yl)propyl)(methyl)amino)piperidin-1-yl)-2-oxoethyl)thiazolidine-2,4-dione (**13e**)

Compound **13e** was prepared according to General Procedure **C**. White solid; Yield 60%. ^1^H NMR (700 MHz, DMSO) δ = 8.31 (d, *J* = 3.1 Hz, 1H), 7.99 (s, OH), 7.97 (d, *J* = 1.8 Hz, 1H), 7.75 (s, 1H), 7.72 (s, 1H), 7.71–7.67 (m, 3H), 7.65–7.61 (m, 3H), 7.40 (tt, *J* = 8.9, 6.6 Hz, 1H), 7.17 (ddt, *J* = 12.0, 9.1, 2.9 Hz, 1H), 6.96 (tt, *J* = 8.5, 2.3 Hz, 1H), 4.63 (dd, *J* = 16.7, 9.2 Hz, 1H), 4.59 (d, *J* = 14.4 Hz, 1H), 4.54 (ddd, *J* = 14.4, 7.5, 3.9 Hz, 2H), 4.31–4.25 (m, 2H), 3.94 (d, *J* = 13.4 Hz, 1H), 3.02–2.94 (m, 1H), 2.87 (d, *J* = 4.6 Hz, 2H), 2.55 (ddt, *J* = 16.3, 12.0, 3.3 Hz, 1H), 2.12 (d, *J* = 2.6 Hz, 3H). ^13^C NMR (176 MHz, DMSO) δ = 167.85, 166.74, 166.62, 165.10, 164.97, 162.54 (d, *J* = 3.6 Hz), 162.38–160.71 (m), 158.75 (dd, *J* = 247.2, 12.3 Hz), 150.28, 144.82 (d, *J* = 6.1 Hz), 135.29, 132.09, 131.73, 131.67, 129.67 (d, *J* = 8.8 Hz), 129.39, 126.33 (d, *J* = 13.7 Hz), 121.67, 110.64 (d, *J* = 20.7 Hz), 103.66 (t, *J* = 26.8 Hz), 74.05 (dd, *J* = 12.4, 5.5 Hz), 61.59, 59.50 (d, *J* = 30.4 Hz), 55.49, 43.66, 42.63, 41.40, 39.41 (dp, *J* = 42.0, 21.0 Hz), 27.87, 27.57, 27.28, 26.94. ESI-MS (*m*/*z*): Found mass for C_29_H_29_ClF_2_N_6_O_4_S = 631.2 [M + H]^+^.

##### (Z)-5-(4-chlorobenzylidene)-3-(2-(4-(4-((2-(2,4-difluorophenyl)-2-hydroxy-3-(1H-1,2,4-triazol-1-yl)propyl)(methyl)amino)phenyl)piperazin-1-yl)-2-oxoethyl)thiazolidine-2,4-dione (**13f**)

Compound **13f** was prepared according to General Procedure **C**. White solid; Yield 69%. ^1^H NMR (600 MHz, DMSO) δ = 8.0 (s, 1H), 7.7 (s, 1H), 7.4 (s, 1H), 7.4 (d, *J* = 8.2, 2H), 7.3 (d, *J* = 8.2, 2H), 7.1 (q, *J* = 8.3, 1H), 6.9 (d, *J* = 11.9, 1H), 6.7 (d, *J* = 8.6, 1H), 6.6 (d, *J* = 8.5, 2H), 6.4 (d, *J* = 8.5, 2H), 5.6 (s, 1H), 4.5 (d, *J* = 14.3, 1H), 4.4 (s, 2H), 4.3 (d, *J* = 14.3, 1H), 3.5–3.4 (m, 1H), 3.4 (t, *J* = 5.1, 2H), 3.3–3.3 (m, 3H), 2.8–2.7 (m, 2H), 2.7–2.6 (m, 2H), 2.2 (s, 3H). ^13^C NMR (151 MHz, DMSO) δ = 166.8, 165.2, 163.1, 161.8 (dd, *J* = 245.5, 13.0), 159.0 (dd, *J* = 246.8, 11.6), 150.5, 145.1, 145.0, 142.4, 135.4, 132.2, 131.8, 131.8, 130.1, 129.5, 125.4 (d, *J* = 10.3), 121.8, 118.1, 113.6, 110.9 (d, *J* = 20.4), 103.8, 76.2 (d, *J* = 6.0), 60.4, 54.9, 50.5, 50.1, 44.2, 42.8, 41.8, 40.2. ESI-MS (*m*/*z*): Found mass for C_34_H_32_ClF_2_N_7_O_4_S = 707.8 [M + H]^+^.

##### (Z)-5-(4-chlorobenzylidene)-3-(2-(4-(((2-(2,4-difluorophenyl)-2-hydroxy-3-(1H-1,2,4-triazol-1-yl)propyl)(methyl)amino)methyl)piperidin-1-yl)-2-oxoethyl)thiazolidine-2,4-dione (**13g**)

Compound **13g** was prepared according to General Procedure **C**. White solid; Yield 73%.^1^H NMR (600 MHz, DMSO) δ = 8.3 (s, 1H), 8.0 (s, 1H), 7.8 (d, *J* = 2.1, 1H), 7.7 (d, *J* = 8.6, 2H), 7.6 (d, *J* = 8.6, 2H), 7.4 (q, *J* = 6.8, 1H), 7.2 (ddd, *J* = 11.8, 9.1, 2.6, 1H), 7.0 (td, *J* = 8.5, 2.6, 1H), 5.6 (s, 1H), 4.6–4.5 (m, 2H), 4.5 (s, 2H), 4.2 (t, *J* = 14.0, 1H), 3.8 (t, *J* = 13.0, 1H), 3.0–3.0 (m, 1H), 2.9 (d, *J* = 13.8, 1H), 2.6 (d, *J* = 13.6, 1H), 2.6–2.5 (m, 1H), 2.3–2.2 (m, 1H), 2.2–2.1 (m, 1H), 2.1 (s, 3H), 1.6–1.5 (m, 2H), 1.5 (dd, *J* = 97.3, 13.1, 1H), 1.0–0.8 (m, 1H), 0.8–0.6 (m, 1H). ^13^C NMR (151 MHz, DMSO) δ = 166.8, 165.2, 162.6, 161.6 (dd, *J* = 245.9, 13.4), 158.9 (dd, *J* = 248.2, 12.1), 150.5, 144.9, 135.4, 132.2, 131.8, 131.8, 130.1–129.8 (m), 129.5, 126.7–126.2 (m), 121.8, 110.6 (d, *J* = 20.6), 103.7 (t, *J* = 26.5), 75.1–74.3 (m), 64.8 (d, *J* = 19.2), 64.1, 55.9, 44.1, 44.0, 42.8, 41.8, 40.1, 33.5, 29.5. ESI-MS (*m*/*z*): Found mass for C_30_H_31_ClF_2_N_6_O_4_S = 645.2 [M + H]^+^.

##### (Z)-N-(4-(4-(2-(5-(4-chlorobenzylidene)-2,4-dioxothiazolidin-3-yl)acetyl)piperazin-1-yl)phenyl)-N-(2-(2,4-difluorophenyl)-2-hydroxy-3-(1H-1,2,4-triazol-1-yl)propyl)acetamide (**17f**)

Compound **17f** was prepared according to General Procedure **B** and **C.** Total yield 62%. ^1^H NMR (600 MHz, DMSO) δ = 8.4 (s, 1H), 8.0 (s, 1H), 7.7 (s, 1H), 7.7 (d, *J* = 8.7, 2H), 7.6 (d, *J* = 8.6, 2H), 7.4 (td, *J* = 8.9, 6.7, 1H), 7.0–6.9 (m, 2H), 6.9–6.5 (m, 4H), 6.4 (s, 1H), 4.7 (s, 2H), 4.6–4.5 (m, 2H), 4.4 (d, *J* = 14.5, 1H), 3.9 (d, *J* = 14.5, 1H), 3.7 (t, *J* = 5.2, 2H), 3.6–3.5 (m, 2H), 3.3–3.2 (m, 2H), 3.1 (d, *J* = 5.1, 2H), 1.7 (s, 3H). ^13^C NMR (151 MHz, DMSO) δ = 173.2, 166.9, 165.2, 163.2, 162.0 (dd, *J* = 249.4, 12.3), 158.8 (dd, *J* = 248.2, 12.0), 150.5, 149.3, 145.1, 135.3 (d, *J* = 34.8), 132.3, 131.9, 131.8, 130.1, 129.5, 128.0, 121.8, 115.7, 110.8 (d, *J* = 22.8), 103.9–103.1 (m), 75.3, 56.4, 55.5, 48.1, 47.8, 43.8, 42.8, 41.5, 22.4. ESI-MS (*m*/*z*): Found mass for C_35_H_32_ClF_2_N_7_O_5_S = 736.2 [M + H]^+^.

##### (Z)-N-(4-(4-(2-(5-(4-chlorobenzylidene)-2,4-dioxothiazolidin-3-yl)acetyl)piperazin-1-yl)phenyl)-N-(2-(2,4-difluorophenyl)-2-hydroxy-3-(1H-1,2,4-triazol-1-yl)propyl)-4-fluorobenzamide (**18f**)

Compound **18f** was prepared according to General Procedure **B** and **C.** Total yield 57%. ^1^H NMR (600 MHz, DMSO) δ = 8.4 (s, 1H), 8.0 (s, 1H), 7.8 (s, 1H), 7.7 (d, *J* = 8.7, 2H), 7.6 (d, *J* = 8.6, 2H), 7.4 (td, *J* = 9.0, 6.8, 1H), 7.3–7.2 (m, 2H), 7.0 (t, *J* = 8.9, 2H), 6.9 (td, *J* = 8.4, 2.6, 1H), 6.9–6.8 (m, 1H), 6.8–6.5 (m, 4H), 6.4 (s, 1H), 4.7 (s, 2H), 4.6 (s, 2H), 4.5 (d, *J* = 14.5, 1H), 4.3 (d, *J* = 14.6, 1H), 3.6 (t, *J* = 5.3, 2H), 3.6–3.5 (m, 2H), 3.2–3.1 (m, 2H), 3.1–3.0 (m, 2H). ^13^C NMR (151 MHz, DMSO) δ = 171.1, 166.8, 165.2, 163.2, 161.8 (dd, *J* = 246.1, 12.9), 158.8 (dd, *J* = 246.7, 12.6), 150.6, 148.5, 145.2, 135.4, 132.2, 132.2, 131.9, 131.8, 131.8, 130.8 (d, *J* = 8.6), 130.0, 129.5, 128.3, 124.5 (d, *J* = 14.6), 121.8, 115.2, 114.7 (d, *J* = 21.8), 110.7 (d, *J* = 20.4), 103.6 (t, *J* = 27.1), 75.3, 56.9, 55.5, 48.1, 47.7, 43.7, 42.7, 41.3. ESI-MS (*m*/*z*): Found mass for C_40_H_33_ClF_3_N_7_O_5_S = 816.3 [M + H]^+^.

##### (Z)-N-(4-(4-(2-(5-(4-chlorobenzylidene)-2,4-dioxothiazolidin-3-yl)acetyl)piperazin-1-yl)phenyl)-N-(2-(2,4-difluorophenyl)-2-hydroxy-3-(1H-1,2,4-triazol-1-yl)propyl)-4-(trifluoromethyl)benzamide (**19f**)

Compound **19f** was prepared according to General Procedure **B** and **C.** Total yield 61%.^1^H NMR (600 MHz, DMSO) δ = 8.4 (s, 1H), 8.0 (s, 1H), 7.8 (s, 1H), 7.7 (d, *J* = 8.7, 2H), 7.6 (d, *J* = 8.7, 2H), 7.6 (d, *J* = 8.0, 2H), 7.5–7.4 (m, 1H), 7.3 (d, *J* = 8.0, 2H), 6.9–6.8 (m, 2H), 6.8–6.6 (m, 4H), 6.2 (s, 1H), 4.7–4.6 (m, 4H), 4.5 (d, *J* = 14.4, 1H), 4.3 (d, *J* = 14.5, 1H), 3.7–3.6 (m, 2H), 3.6–3.5 (m, 2H), 3.1–3.1 (m, 3H), 3.1–3.0 (m, 1H). ^13^C NMR (151 MHz, DMSO) δ = 170.5, 166.8, 165.2, 163.2, 161.9 (dd, *J* = 246.1, 12.7), 158.9 (dd, *J* = 246.5, 12.7), 150.6, 148.7, 145.2, 140.3, 135.4, 134.3, 132.2, 131.8, 131.8, 130.1–129.9 (m), 129.7–128.0 (m), 128.7, 124.7, 124.5 (d, *J* = 13.2), 122.8, 121.8, 115.1, 110.6 (d, *J* = 20.4), 103.6 (t, *J* = 26.9), 75.7–74.9 (m), 56.4, 55.4, 48.0, 47.6, 43.7, 42.7, 41.3. ESI-MS (*m*/*z*): Found mass for C_41_H_33_ClF_5_N_7_O_5_S = 866.1 [M + H]^+^.

##### General Procedure **D** for the Synthesis of Compounds **12e**–**12g**, **32**

To a solution of compounds **4e**–**g** (1.0 mmol) in methanol (5 mL) and acetic acid (0.2 mL) was added an aqueous solution of formaldehyde (30 wt%, 1.5 mmol) at room temperature. After 10 min, sodium cyanoborohydride (2.0 mmol) was added portionwise, and the reaction mixture was stirred at room temperature for 24 h. Reaction progress was monitored by TLC. Upon completion, the reaction mixture was diluted with water (20 mL) and extracted with dichloromethane (3 × 15 mL). The combined organic layers were washed with saturated aqueous sodium chloride solution, dried over anhydrous sodium sulfate, filtered, and concentrated under reduced pressure. The crude product was purified by silica gel column chromatography using dichloromethane/methanol (25:1, *v*/*v*) as the eluent to afford compounds **12e**–**12g**, **32**.

##### General Procedure **E** for Acetylation of Compounds **14**–**16f**

To a solution of the appropriate amine **1b** (1.0 mmol) in dry dichloromethane (3 mL) was added triethylamine (2.0 mmol), and the reaction mixture was stirred for 10 min. Acyl chloride (1.2 mmol) was then added dropwise at 0 °C. The reaction mixture was allowed to warm to room temperature and stirred overnight. Reaction progress was monitored by TLC. The reaction was quenched by the addition of saturated sodium bicarbonate solution. The aqueous layer was extracted with ethyl acetate (3 × 15 mL). The combined organic layers were washed with brine, dried over anhydrous sodium sulfate, filtered, and concentrated under reduced pressure. The crude product was purified by silica gel column chromatography using dichloromethane/methanol (25:1, *v*/*v*) as the eluent to afford the corresponding acylated derivatives.

##### Synthesis of Compound **24**

Epichlorohydrin (2.7 g, 30 mmol) and tetrabutylammonium chloride (350 mg, 3 mmol) were added to a solution of 2-(2,4-difluorophenyl)-1-(piperazin-1-yl)-3-(1H-1,2,4-triazol-1-yl)propan-2-ol (3.5 g, 10 mmol) in N-methylpyrrolidone (NMP, 25 mL). The reaction mixture was stirred at 75 °C for 3 h and then at 45 °C for an additional 12 h. The mixture was cooled to room temperature, and the excess epichlorohydrin was removed under reduced pressure. The residue was diluted with water (100 mL) and extracted twice with EtOAc (2 × 50 mL). The combined organic extracts were washed with water (25 mL) and brine (10 mL), dried over Na_2_SO_4_, and concentrated under reduced pressure. The residue was purified by column chromatography using CH_2_Cl_2_/MeOH (50:1 to 10:1) as the eluent to afford compound **24** (2.69 g, 65%) as a colorless oil.

^1^H NMR (600 MHz, CDCl_3_) δ = δ 8.36 (d, *J* = 25.4 Hz, 1H), 7.73 (d, *J* = 4.5 Hz, 1H), 7.37 (q, *J* = 8.5 Hz, 1H), 7.15 (td, *J* = 9.2, 4.6 Hz, 1H), 6.94 (td, *J* = 8.5, 2.6 Hz, 1H), 6.48 (d, *J* = 6.2 Hz, 1H), 6.12 (s, 1H), 4.61 (d, *J* = 14.2 Hz, 1H), 4.55 (d, *J* = 8.6 Hz, 2H), 4.40 (dd, *J* = 11.3, 7.1 Hz, 1H), 4.03 (dd, *J* = 11.5, 5.3 Hz, 1H), 3.69–3.54 (m, 2H), 3.38 (s, 3H), 3.31 (s, 1H), 2.89 (t, *J* = 15.6 Hz, 1H), 2.69 (d, *J* = 13.7 Hz, 2H), 2.60 (s, 2H), 2.37 (s, 2H), 1.13 (s, 1H). ^13^C NMR (151 MHz, CDCl_3_) δ 174.08, 162.77, 162.69, 161.15, 161.07, 160.11, 160.03, 158.47, 158.39, 150.70, 145.18, 130.66, 130.07, 130.02, 129.97, 126.60, 126.52, 111.02, 110.88, 104.15, 103.97, 103.79, 74.76, 74.72, 74.69, 66.20, 63.83, 60.68, 60.56, 56.01, 55.97, 54.55, 54.40, 53.79, 53.46, 49.93, 49.92, 48.79, 44.51, 44.49, 37.73, 34.99, 34.77, 30.40, 29.28, 21.67, 18.94, 17.52, 14.13. ESI-MS (*m*/*z*): Found mass for C_18_H_24_ClF_2_N_5_O_2_ = 416.2 [M + H]^+^.

##### General Procedure **F** for the Synthesis of Compounds **25**, **26**, **27**

Racemic epichlorohydrin (10.0 mmol, 1.0 equiv) was added to a solution of the corresponding cyclic amine (10.0 mmol, 1.0 equiv) in ethanol (50 mL). The reaction mixture was stirred at room temperature overnight. Upon completion, the solvent was removed under reduced pressure to afford the crude chlorohydrin products **25**, **26** as a colorless oil. The obtained material was used in subsequent transformations without further purification.

##### (Z)-5-(4-chlorobenzylidene)-3-(3-(4-(2-(2,4-difluorophenyl)-2-hydroxy-3-(1H-1,2,4-triazol-1-yl)propyl)piperazin-1-yl)-2-hydroxypropyl)thiazolidine-2,4-dione (**28**)

*Method A:* A suspension of the potassium salt of (Z)-5-(4-chlorobenzylidene)thiazolidine-2,4-dione (4.0 g, 14 mmol) in a solution of 1-(4-(3-chloro-2-hydroxypropyl)piperazin-1-yl)-2-(2,4-difluorophenyl)-3-(1H-1,2,4-triazol-1-yl)propan-2-ol (4.0 g, 14 mmol) in anhydrous DMF (50 mL) was stirred vigorously at 90 °C for 12 h. The reaction mixture was cooled to room temperature and concentrated in vacuo. The resulting solid residue was triturated with water (50 mL) and EtOAc (100 mL). The organic layer was separated and washed with water (20 mL). The organic phase was then partially concentrated (to approximately half of the initial volume) and cooled to +5 °C. The precipitate was collected by filtration, washed with i-PrOH (5 mL), and dried at room temperature to afford compound **28**. Yield 42%.

*Method B:* To a solution of (Z)-5-(4-chlorobenzylidene)-3-(2-hydroxy-3-(piperazin-1-yl)propyl)thiazolidine-2,4-dione (3.0 g, 7.8 mmol) in a PhMe/NMP mixture (20:1, 100 mL) were added 1-((2-(2,4-difluorophenyl)oxiran-2-yl)methyl)-1H-1,2,4-triazole (1.9 g, 8.0 mmol) and triethylamine (5 mL, 38 mmol). The reaction mixture was stirred at 90 °C for 12 h, then cooled to room temperature and concentrated in vacuo. The residue was purified by flash chromatography using EtOAc/MeOH/Et_3_N (25:1:0.1, *v*/*v*/*v*) as the eluent to afford the product as an amorphous powder (3.2 g, 67%).

^1^H NMR (600 MHz, DMSO) δ = 8.26 (s, 1H), 7.89 (s, 1H), 7.71 (s, 1H), 7.67–7.56 (m, 4H), 7.36 (dq, *J* = 16.6, 8.4 Hz, 1H), 7.15–7.06 (m, 1H), 6.92 (t, *J* = 8.2 Hz, 1H), 5.03 (s, 1H), 4.51 (s, 2H), 3.90 (t, *J* = 6.6 Hz, 1H), 3.68–3.58 (m, 2H), 3.55–3.32 (m, 1H), 3.28 (t, *J* = 7.0 Hz, 3H), 2.68 (s, 4H), 2.53–2.46 (m, 2H), 2.32 (d, *J* = 19.1 Hz, 6H), 2.16 (t, *J* = 8.1 Hz, 3H), 1.89 (q, *J* = 7.4 Hz, 4H). ^13^C NMR (151 MHz, DMSO) δ = 167.37, 165.73, 163.24, 163.15, 161.60, 161.51, 159.85, 159.78, 158.22, 149.26, 144.83, 135.21, 131.94, 131.71, 131.23, 130.06, 129.53, 123.29, 122.45, 111.52, 111.39, 104.65, 104.48, 104.31, 72.90, 62.58, 62.57, 62.03, 55.52, 48.59, 48.53, 45.27, 29.04. ESI-MS (*m*/*z*): Found mass for C_28_H_29_ClF_2_N_6_O_4_S = 618.9 [M + H]^+^ HPLC–MS: Phenomenex C18 column (150 mm, 3.3 μm), column temperature 30 °C, mobile phase: acetonitrile; *m*/*z* 619.1810; *t*R = 10.4–10.5 min. The spectral data were consistent with those previously reported [36].

##### (Z)-5-(4-chlorobenzylidene)-3-(3-(4-((2-(2,4-difluorophenyl)-2-hydroxy-3-(1H-1,2,4-triazol-1-yl)propyl)amino)piperidin-1-yl)-2-hydroxypropyl)thiazolidine-2,4-dione (**31**)

Compound **31** was prepared according to General Procedure **D** from compound **2a** and compound **28**. The product was obtained as a colorless amorphous powder. Yield 22%.

^1^H NMR (400 MHz, DMSO) δ = 8.24 (s, 1H), 7.92 (s, 1H), 7.87 (s, 1H), 7.69 (s, 1H), 7.60 (d, *J* = 8.7 Hz, 2H), 7.55 (d, *J* = 1.4 Hz, 2H), 7.30 (qd, *J* = 14.4, 7.7 Hz, 2H), 7.07 (ddd, *J* = 11.7, 9.1, 2.3 Hz, 1H), 6.88 (td, *J* = 8.4, 2.2 Hz, 1H), 4.48 (s, 2H), 3.65 (s, 2H), 3.46 (s, 5H), 3.33–3.20 (m, 1H), 3.33–2.83 (m, 16H), 2.71 (s, 2H), 2.38–2.09 (m, 4H), 2.00–1.85 (m, 1H), 1.80 (t, *J* = 10.6 Hz, 1H), 1.62 (d, *J* = 10.9 Hz, 3H), 1.40–1.29 (m, 1H), 1.29–1.19 (m, 2H), 1.19–1.06 (m, 2H), 0.91–0.63 (m, *J* = 7.3 Hz, 2H). ^13^C NMR (101 MHz, DMSO) δ = 167.56, 166.18, 162.74, 150.75, 145.15, 135.58, 132.33, 132.15, 132.01, 131.93, 131.50, 129.81, 129.05, 122.77, 74.27, 74.22, 63.80, 63.21, 52.59, 52.41, 49.01, 47.31, 38.51, 36.19, 32.61, 31.15, 30.23, 28.80, 23.66, 22.84, 14.27, 11.17. ESI-MS (*m*/*z*): C_29_H_31_ClF_2_N_6_O_4_S = 633 [M]^+^.

##### (Z)-5-(4-chlorobenzylidene)-3-(3-(4-((2-(2,4-difluorophenyl)-2-hydroxy-3-(1H-1,2,4-triazol-1-yl)propyl)(methyl)amino)piperidin-1-yl)-2-hydroxypropyl)thiazolidine-2,4-dione (**32**)

*Method A*. Compound **32** was prepared according to General Procedure **G** from potassium (Z)-5-(4-chlorobenzylidene)thiazolidine-2,4-dione and 1-[3-chloro-2-hydroxypropyl-(4-((2-(2,4-difluorophenyl)-2-hydroxy-3-(1H-1,2,4-triazol-1-yl)propyl)methylamino)piperidin-1-yl)-(27). The product was obtained as a colorless amorphous powder. Yield 34%. *Method B.* Compound **32** was prepared according to General Procedure D from compound 31 and formaldehyde. The product was obtained as a yellow amorphous powder. Yield 32%.

^1^H NMR (700 MHz, DMSO) δ 8.33 (s, 1H), 7.95 (s, 1H), 7.95 (s, 2H), 7.77 (s, 1H), 7.66 (d, *J* = 8.3 Hz, 4H), 7.62 (d, *J* = 8.5 Hz, 4H), 7.39 (td, *J* = 9.1, 6.7 Hz, 1H), 7.18 (ddd, *J* = 11.9, 9.0, 2.6 Hz, 1H), 6.96 (td, *J* = 8.5, 2.7 Hz, 1H), 6.48 (s, 1H), 5.76 (s, 0H), 5.66 (q, *J* = 4.2 Hz, 2H), 5.64 (s, 1H), 4.58 (q, *J* = 14.3 Hz, 2H), 4.28–4.25 (m, 3H), 3.41 (q, *J* = 6.2, 5.1 Hz, 5H), 3.35–3.24 (m, 3H), 3.21 (d, *J* = 15.6 Hz, 1H), 2.99 (d, *J* = 4.5 Hz, 2H), 2.92 (d, *J* = 4.9 Hz, 1H), 2.77–2.72 (m, 1H), 2.37 (q, *J* = 6.4, 5.6 Hz, 2H), 2.33–2.25 (m, 1H), 1.97 (s, 3H), 1.23 (s, 1H). ^13^C NMR (176 MHz, DMSO) δ 168.61, 168.04, 166.62, 165.07, 161.62 (dd, *J* = 245.7, 12.3 Hz), 158.61 (dd, *J* = 246.8, 12.2 Hz), 150.45, 145.03, 135.17, 131.77, 131.68, 129.37, 126.07 (d, *J* = 30.6 Hz), 121.97, 110.79 (d, *J* = 19.7 Hz), 103.83 (t, *J* = 27.1 Hz), 72.98 (d, *J* = 4.6 Hz), 58.59 (d, *J* = 10.8 Hz), 56.54, 52.36, 52.25, 51.93, 51.85, 48.84, 45.24, 45.11, 42.56, 40.74, 40.41, 38.14, 21.06. ESI-MS (*m*/*z*): C_30_H_33_ClF_2_N_6_O_4_S = 647.1900 [M + H]^+^.

##### General Procedure **G** for the Synthesis of Compounds **34**–**35**

To a solution of the corresponding triazolyl derivative **20** or **23** (1.0 mmol, 1.0 equiv) in dry DMF (10 mL) were added compound **33** (1.0 mmol, 1.0 equiv) and K_2_CO_3_ (1.0 mmol, 1.0 equiv). The reaction mixture was stirred vigorously at room temperature for 18 h. Upon completion, the solvent was removed under reduced pressure. The residue was diluted with water (50 mL) and EtOAc (100 mL). The organic layer was separated, washed with water (2 × 20 mL), and concentrated under reduced pressure. The resulting precipitate was collected by filtration, washed with diethyl ether (5 mL), and purified by silica gel column chromatography using CH_2_Cl_2_/MeOH (50:1 to 25:1, *v*/*v*) as the eluent to afford compounds **34** and **35** as white amorphous powders.

##### Synthesis of Compound **36**

K_2_CO_3_ (10.1 g, 73.1 mmol) was added to a solution of compound **33** (2.5 g) in dry DMF (36 mL). A stream of gaseous ammonia from a cylinder was then bubbled through the reaction mixture at room temperature until 2.3 g of ammonia had been introduced. The resulting mixture was stirred at room temperature for 18 h, after which the solvent was removed under reduced pressure. The residue was poured into water, and the precipitated product was collected by filtration, washed with water, and dried to afford compound **36**. NMR methods and X-ray structural analysis (Figures S1 and S2).

^1^H NMR (400 MHz, DMSO) δ = 8.02 (t, *J* = 6.9 Hz, 1H), 7.65–7.59 (m, 2H), 7.50–7.42 (m, 2H), 6.86 (s, 1H), 5.30 (s, 1H), 3.95 (q, *J* = 5.3 Hz, 1H), 3.35 (ddt, *J* = 13.0, 6.0, 3.1 Hz, 2H), 3.19–3.13 (m, 1H), 3.13–3.06 (m, OH), 2.83 (dd, *J* = 14.6, 6.3 Hz, 1H).

### 3.8. Crystal Structure Determination of ***36***

A crystal of the compound **36** suitable for X-ray structure determination was mounted on a CCD area detector D8 Venture diffractometer under a stream of cooled nitrogen, and X-ray reflections were measured (λMoK_α_, *ω*-scan mode, temperature 150 K). Olex2 (Intrinsic Phasing) was used for structure solution and refinement [50]. The structure was solved with the SHELXT [51] structure solution program and refined with the XH [52] refinement package using Least Squares minimization. Crystal Data for C_12_H_12_ClNO_2_S (M = 269.23 g/mol): monoclinic, space group P21/c (no. 14), crystal size 0.22 × 0.16 × 0.02 mm^3^, a = 11.0248(8) Å, b = 7.2484(5) Å, c = 30.757(2) Å, β = 91.803(2)°, V = 2456.7(3) Å^3^, Z = 8, T = 150.0 K, μ(MoK_α_) = 0.469 mm^−1^, D_calc_ = 1.459 g/cm^3^, 30,369 reflections measured (2.64° ≤ 2*θ* ≤ 54°), 5428 unique (R_int_ = 0.0507, R_σ_ = 0.0390) which were used in all calculations. The final R_1_ was 0.0929 (>2*θ* (I)), and wR_2_ was 0.2106 (all data), GOOF 1.186. The structure (crystallographic data, atomic coordinates, anisotropic displacement parameters, bond angles and distances, and torsion angles) has been deposited at the Cambridge Crystallographic Database. The CCDC Deposition Number 2530809.

## 4. Conclusions

In this study, we designed, synthesized, and biologically profiled a focused library of triazole-sulfur heterocycle hybrids in which the linker architecture was systematically varied to incorporate alkylamine, amide, 2-hydroxypropyl, and seven-membered thiazepane motifs. The synthetic approaches provided access to four series of hybrid compounds (Figure 1, Figure 2, Figure 3, Figure 4 and Figure 5), including the unexpected thiazepane derivatives **34** and **35** formed through a base-induced rearrangement. Their structures were established by 2D NMR spectroscopy, while that of the related model compound **36** was additionally confirmed by X-ray crystallography. The target compounds were evaluated against panels of reference fungal strains and clinical *Candida* isolates.

Structure–activity relationship analysis demonstrated that amide and cyclic amine linkers generally provided greater antifungal potency than simple N-alkyl-substituted amide linkers. N-Methylation of the proximal linker nitrogen produced variable and strain-dependent effects. Replacement of the thiazolidine-2,4-dione ring with a seven-membered thiazepane scaffold preserved activity against selected fungal species but substantially altered the activity against *C. albicans* strains. Several compounds were more active than fluconazole and, against selected isolates, showed activity comparable to or greater than that of itraconazole or voriconazole (Table 1 and Table 2).

Molecular docking was used to generate putative CYP51-binding models and rationalize the observed structure–activity relationships. The calculations suggested that compounds containing a basic nitrogen atom in the linker may form a cation–π interaction with Tyr118 and, depending on linker geometry, additional hydrogen bonds within the CYP51 substrate-access channel. These predicted interactions remain to be experimentally validated. Independently, fluorescence microscopy indicated that compounds **6a** and **28** perturb sterol-associated cellular homeostasis. The phenotypes resembled those induced by voriconazole and differed from those of the *Δerg3* mutant, supporting an effect on sterol homeostasis rather than rapid nonspecific membrane disruption. Consistent with these observations, SCRAPPY profiling showed that compounds **6a** and **28** produced protein-response profiles broadly resembling those of the reference azoles, including increased abundance of the ergosterol-biosynthesis proteins Erg3 and Erg10, the efflux pump Pdr5, and the stress-response protein Gpd1. These findings support an azole-like cellular response but do not establish direct CYP51 inhibition or exclude additional molecular targets. Prolonged exposure to compound 28 resulted in secondary loss of membrane integrity and more pronounced cell-wall and morphological alterations.

Compound **28** emerged as the most promising exploratory lead in the series. It exhibited low MIC values against *C. parapsilosis* isolates, including an MIC of 0.06 μg·mL^−1^ at 24 h against ATCC 22019, and retained measurable activity against the azole-resistant C. albicans 8R strain, with MIC values of 0.5 and 8 μg·mL^−1^ after 24 and 48 h, respectively. In the preliminary acute oral toxicity study, a single dose of 300 mg/kg caused no mortality or visible clinical signs of intoxication during the 14-day observation period, although moderate changes in food and water consumption were observed. By contrast, administration at 2000 mg/kg caused substantial mortality. Compound **28** also displayed concentration- and time-dependent cytotoxicity in mammalian cells. In human liver microsomes, it was rapidly metabolized, with a half-life of approximately 3.9 min, predominantly through CYP3A4. These findings identify mammalian-cell cytotoxicity and metabolic stability as important priorities for further optimization. Compound **35** showed substantially greater microsomal stability but strongly inhibited human CYP3A4 and CYP2C9, indicating a potential risk of drug–drug interactions and reducing its attractiveness for further development in its current form.

Overall, the linker-focused hybridization strategy yielded a chemically diverse series of compounds with promising antifungal activity and identified compound **28** as a lead scaffold for continued medicinal chemistry optimization. Further work should focus on metabolite identification, experimental mapping of metabolic soft spots, improvement of microsomal stability and cytotoxicity, direct validation of the molecular target, and pharmacokinetic and in vivo efficacy studies. Evaluation of individual stereoisomers and broader panels of fungal pathogens, including additional *Cryptococcus spp*., Mucorales, and dermatophytes, will help define the therapeutic potential and spectrum of optimized analogues of compound **28**.

## Figures and Tables

**Table 1:** Results of the comparative microbiological evaluation against fungal *strains C. parapsilosis* ATCC 22019, *C. albicans* 604M, *C. krusei* 432M, *C. auris* VGNKI, and *A. fumigatus* ATCC 4664.

Compounds	MIC (µg/mL), 24/48 h								
	*C. parapsilosis* ATCC 22019		*C. albicans* 604M		*C. krusei* 432M		*C. auris* VGNKI		*A. fumigatus* ATCC 46645
	24 h	48 h	24 h	48 h	24 h	48 h	24 h	48 h	48 h
**6a**	0.06	0.125	≤0.015	≤0.015	4	8	0.25	0.25	16
**6** **b**	4	4	0.25	0.25	>32	>32	8	>32	>32
**6c**	2	8	0.25	16	>32	>32	8	>32	>32
**6d**	4	8	0.5	16	>32	>32	16	>32	>32
**7e**	4	4	0.5	0.5	>32	>32	1	1	8
**8f**	0.25	0.5	>32	>32	4	8	>32	>32	16
**9g**	0.125	0.125	<0.015	<0.015	8	8	0.5	1	8
**13** **e**	0.125	0.5	0.25	0.5	4	8	2	4	16
**13g**	0.06	0.06	>32	>32	4	8	>32	>32	>32
**13f**	0.25	0.5	>32	>32	8	8	≥32	>32	>32
Fluco	4	4	>32	>32	>32	>32	>32	>32	>32
Vori	0.06	0.06	0.06	0.06	1	1	8	8	0.5
Posa	0.015	0.015	0.125	0.25	0.125	0.5	2	4	1

Fluco–Fluconazole, Vori–Voriconazole, Posa–Posaconazole.

**Table 2:** Comparative antifungal activity of the synthesized hybrid compounds against clinical *Candida* spp. isolates.

Strains	MIC (µg/mL), 24/48 h						
	28	31	32	34	35	Fluconazol	Itraconazol
*C. parapsilosis* ATCC 22019	0.06/0.25	0.0125/0.0125	0.0125/0.0125	0.125/0.5	0.25/0.25	4/4	0.125/0.125
*C. parapsilosis* 58L	0.06/0.25	0.06/0.06	0.0125/0.025	0.5/1	0.125/0.25	8/16	0.25/0.25
*C. albicans* 8(R)	0.5/8	>32/>32	>32/>32	>32/>32	>32/>32	>32/>32	16/32
*C. albicans* 604M	0.125/0.25	0.5/0.5	1/2	32/32	16/16	>32/>32	16/32
*C. krusei* 432M	2/4	8/16	16/16	8/16	8/16	>32/>32	0.5/1
*C. tropicalis* 3019	1/1	1/2	2/4	4/4	2/4	16/16	2/2
*C. glabrata* 61L	0.25/4	2/4	4/4	2/8	2/8	8/16	0.5/0.5
*C. utilis* 84	1/2	0.5/1	1/2	2/4	1/2	2/4	0.125/0.125

**Table 3:** Comparative microbiological evaluation against dermatophyte strains *M. canis* B-200 and *T. rubrum* U.2002.

	MIC (µg/mL)	
Compounds	*M. canis* B-200	*T. rubrum* U.2002
**7e**	1	1
**13e**	4	2
**28**	0.25	0.25
**31**	0.03	0.03
**32**	0.125	0.5
Fluconazole	8	4
Itraconazole	0.015	0.015
Amphotericin B	8	2

## Data Availability

The original contributions presented in the study are included in the article/Supplementary Materials; further inquiries can be directed to the corresponding author.

## Supplementary Materials

The following supporting information can be downloaded at https://www.mdpi.com/article/10.3390/ph19081260/s1. Figure S1, Structure of two independent molecules **36**; atom displacement ellipsoids are shown at the 50% probability level; Figure S2, Superposition of the two molecules. Shows a superposition of the molecules. Independent molecules have very close geometry; Figure S3, Predicted binding poses of docked ligands. (a) Binding poses of 6a (green) and 6d (purple). Figures depicting key polar interactions of ligands: (b) 13e, (c) 13g, (d) 31, (e) 32, (f) 28, (g) 34; Figure S4, Minimal inhibitory concentrations (MICs) of compounds **28** and **6a** against *S. cerevisiae* BY4742. The MIC for both compounds was determined as 50 μg/mL (clear wells) and used for all experiments for *S. cerevisiae*; Figure S5, Late-stage cellular effects of compound **28** in *S. cerevisiae* BY4742 after 16 h of exposure. Cells were incubated with compound 28 at 50 μg/mL (1× MIC) and 100 μg/mL (2× MIC) for 16 h and stained with CFW and PI. In contrast to the 4-h treatment (Figure 7), where the plasma membrane remained intact (PI^−^), extended incubation led to pronounced PI uptake, indicative of membrane permeabilization. CFW staining was also increased, and cells exhibited loss of rounded morphology, reduced size, and granular cytoplasm. Untreated WT served as control.; Figure S6, Representative phase-contrast images of cells treated with DMSO or compound **28** at 8, 16, and 32 μg/mL for 8, 24, and 48 h. Scale bars are shown in the images; Figure S7–S80, ^1^H and ^13^C NMR spectra of synthesized compounds; Figure S81–S112, ESI-MS spectra of the synthesized compounds; Figure S113–S131, HPLC chromatograms of the synthesized compounds. Table S1, Bond lengths (d, Å) and bond angles (ω, °); Table S2, Characteristic binding descriptors of the docked ligands: cLogP—calculated distribution coefficient n-octanol/water; HB—formation of the hydrogen bond between the side chain arm of the ligand and the residue of the substrate access channel; r [Å]—distance between the basic nitrogen and the center of the Y118 phenyl ring; Table S3, Chemical shifts of the ^1^H and ^13^C signals of the thiazepane compounds **34**, **35** and **36**. References [53,54,55,56] are cited in the Supplementary Materials.
