# Supplementary material for: Linker Engineering of Hybrid Triazole-Thiazolidine Antifungals Identifies a Promising Lead Against Drug-Resistant Candida Species
Source: Pharmaceuticals (Basel). 2026 Aug 10;19(8):1260. doi: 10.3390/ph19081260 (PMC13516150; doi:10.3390/ph19081260)
Supplement: Supplementary file 1 [file pharmaceuticals-19-01260-s001.zip › pharmaceuticals-4468088-supplementary.pdf]

Supplementary Material

Linker Engineering of Hybrid Triazole–Thiazolidine Antifungals Identifies a Promising Lead against Drug-Resistant *Candida* Species

Alexander Yu. Rudenko[1,2*], Olga A. Komarova[3], Alexander Yu.Simonov[4], Ratislav M. Ozhiganov[1,2], Dmitrii A. Averianov[2], Sofiia R. Kuklich[1], Ekaterina A. Guseva[1,2], Sofya Y. Sokolskaya[5], L.G. Kuz'mina[6], Natalia E. Grammatikova[7],Alexander B. Kulko[8], Victoria A. Bidiuk[9], Sofia S. Mariasina [2,10], Vasiliy A. Ivlev[10], Peter V. Sergiev[1,2], Vladimir I. Polshakov[2], Alexey B. Mantsyzov[2], Igor B. Levshin[4]

[1] Belozersky Institute of Physico-Chemical Biology, M.V. Lomonosov Moscow State University, Moscow, Russia
[2] Chemical Department, M.V. Lomonosov Moscow State University, Moscow, Russia
[3] Shemyakin-Ovchinnikov Institute of Bioorganic Chemistry, Miklukho-Maklaya 16/10, 117997 Moscow, Russia
[4] Gause Institute of New Antibiotics, 11 B. Pirogovskaya Street, 119021 Moscow, Russia
[5] Faculty of Fundamental Medicine, Lomonosov Moscow State University, 119991 Moscow, Russia
[6] N.S. Kurnakov Institute of General and Inorganic Chemistry, Russian Academy of Sciences, 119991 Moscow, Russia
[7] M.V. Lomonosov Institute of Fine Chemical Technologies, MIREA-Russian Technological University, 119571 Moscow, Russia
[8] Clinical Antituberculosis Center, Moscow Government Health Department Scientific, st. Stromynka, 10, 107014 Moscow, Russia
[9] Federal Research Center of Biotechnology of the RAS, 33-2 Leninsky prospect, Moscow, 119071, Russia
[10] Research and Educational Resource Center "Pharmacy", RUDN University, Moscow, Russia

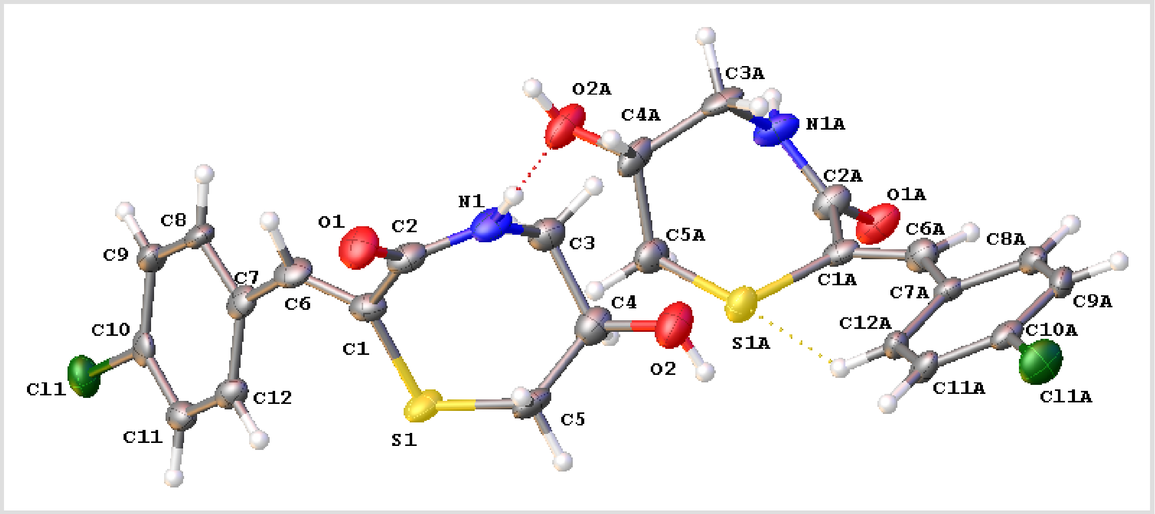

Supplementary Figure S1. Structure of two independent molecules **36**; atom displacement ellipsoids are shown at the 50% probability level.

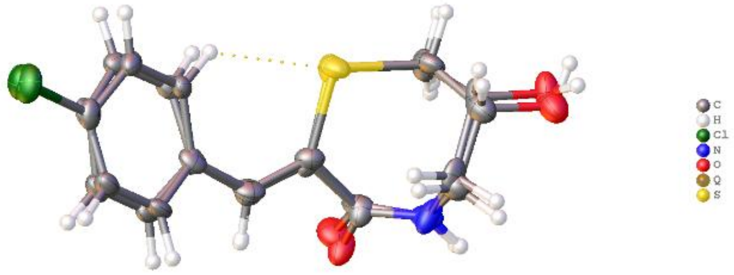

Supplementary Figure S2. Superposition of the two molecules. Shows a superposition of the molecules. Independent molecules have very close geometry.

The seven-membered heterocycle of both molecules has a conformation of somewhat twisted boat, with the C5/C5A and N1/N1A atoms displaced from the mean plane of C3C4SC1/C3AC4ASAC1 atoms.

Table S1 lists selected bond lengths and angles. In the independent molecules the corresponding parameters are equal within the experimental error.

Supplementary Table S1. Bond lengths (d, Å) and bond angles (ω˚).

Bond	d	Bond	d	Angle	ω	Angle	ω
Cl1-C10	1.743(5)	Cl1A-C10A	1.751(5)	C1-S1-C5	103.3(3)	C1A-S1A-C5A	103.6(3)
S1-C1	1.765(5)	S1A-C1A	1.770(5)	C2-N1-C3	124.1(5)	C2A-N1A-C3A	124.2(4)
S1-C5	1. 810(6)	S1A-C5A	1.803(5)	C2-C1-S1	119.1(4)	C2A-C1A-S1A	117.3(4)
O1-C2	1.234(7)	O1A-C2A	1.235(6)	C6-C1-S1	124.3(4)	C6A-C1A-S1A	124.8(4)
O2-C4	1.419(7)	O2A-C4A	1.427(6)	O1-C2-N1	123.3(5)	O1A-C2A-N1A	122.4(5)
N1-C2	1.339(7)	N1A-C2A	1.348(7)	O1-C2-C1	119.4(5)	O1A-C2A-C1A	121.3(5)
N1-C3	1.446(7)	N1A-C3A	1.458(7)	N1-C2-C1	117.3(5)	N1A-C2A-C1A	116.1(4)
C1-C2	1.512(7)	C1A-C2A	1.507(7)	N1-C3-C4	114.1(4)	N1A-C3A-C4A	112.6(4)
C1-C6	1.330(8)	C1A-C6A	1.332(7)	O2-C4-C3 ….	109.4(5)	O2A-C4A-C3A	112.1(5)
C3-C4	1.517(8)	C3A-C4A	1.527(8)	O2-C4-C5	109.6(5)	O2A-C4A-C5A	106.1(4)
C4-C5	1.536(8)	C4A-C5A	1.518(7)				
C6-C7	1.463(7)	C6A-C7A	1.484(7)				
C7-C8	1.398(7)	C7A-C8A	1.395(7)				

C7-C12 1.410(7)	C7A-C12A 1.400(7)		
C8-C9 1.374(8)	C8A-C9A 1.384(7)		
C9-C10 1.386(7)	C9A-C10A 1.380(7)		
C10-C11 1.379(7)	C10A-C11A 1.376(7)		
C11-C12 1.382(7)	C11A-C12A 1.390(7)		

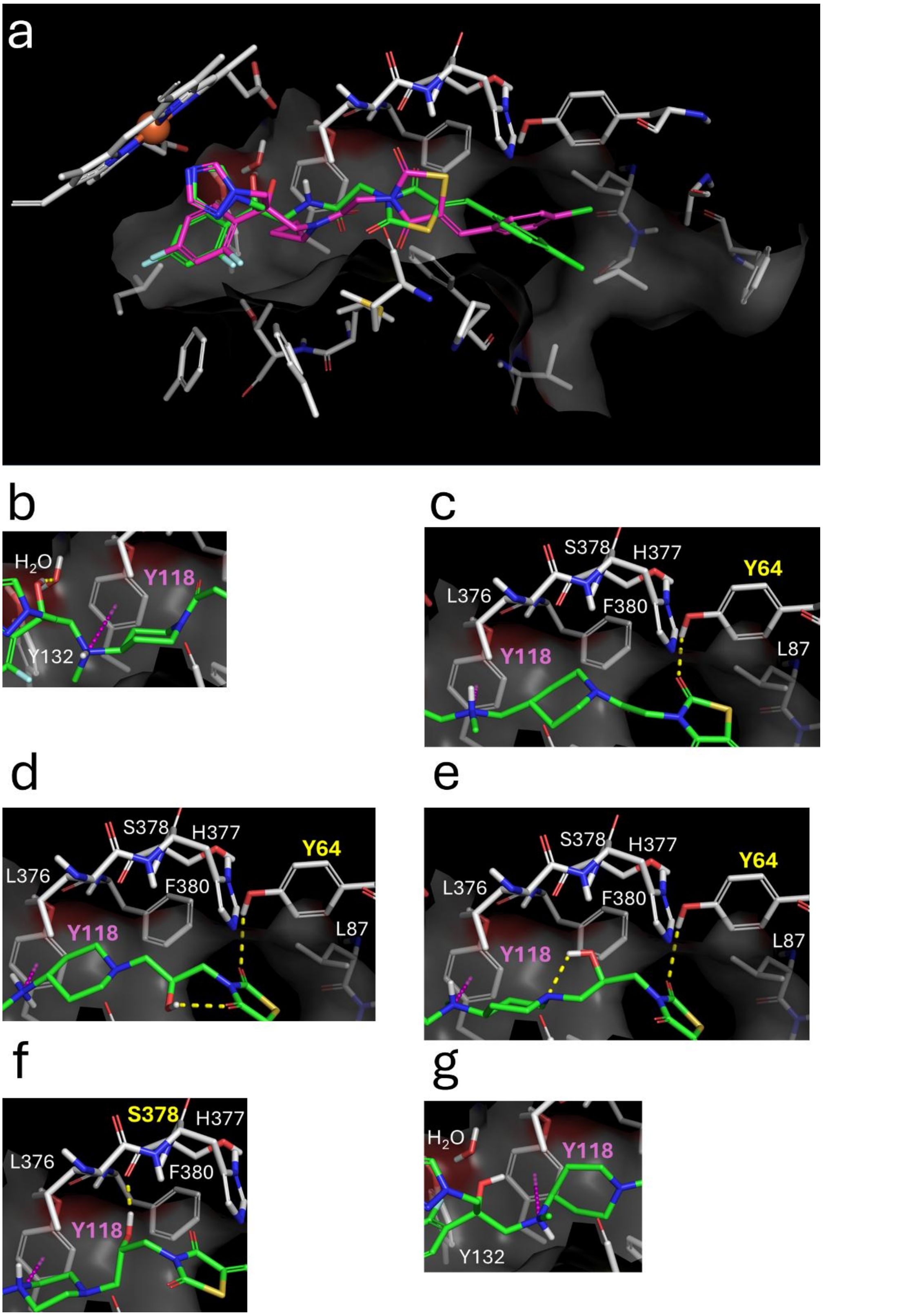

Supplementary Figure S3. Predicted binding poses of docked ligands. (a) Binding poses of 6a (green) and 6d (purple). Figures depicting key polar interactions of ligands: (b) 13e, (c) 13g, (d) 31, (e) 32, (f) 28, (g) 34.

Supplementary Table S2. Characteristic binding descriptors of the docked ligands: cLogP – calculated distribution coefficient n-octanol/water; HB – formation of the hydrogen bond between the side chain arm of the ligand and the residue of the substrate access channel; r [Å] – distance between the basic nitrogen and the center of the Y118 phenyl ring.

ID	cLogP	r [Å]	HB
6a	1.9	NA	-
6c	3.0	NA	-
6d	2.7	NA	-
8f	3.4	NA	-
13f	3.6	NA	-
7e	2.4	4.7	-
13e	2.6	5.4	-
9g	2.6	4.9	+
13g	2.8	5.5	+
32	2.2	4.2	+
31	2.4	4.4	+
28	2.0	4.7	+
34	2.9	5.8	+
35	2.5	ND	ND

NA – not applicable, ND – not determined.

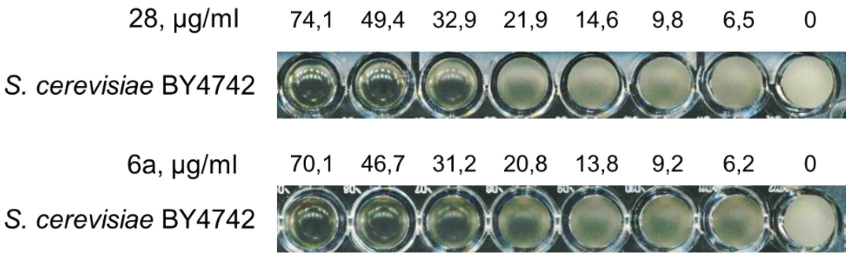

Supplementary Figure S4. Minimal inhibitory concentrations (MICs) of compounds 28 and 6a against *S. cerevisiae* BY4742. The MIC for both compounds was determined as 50 µg/mL (clear wells) and used for all experiments for *S. cerevisiae.*

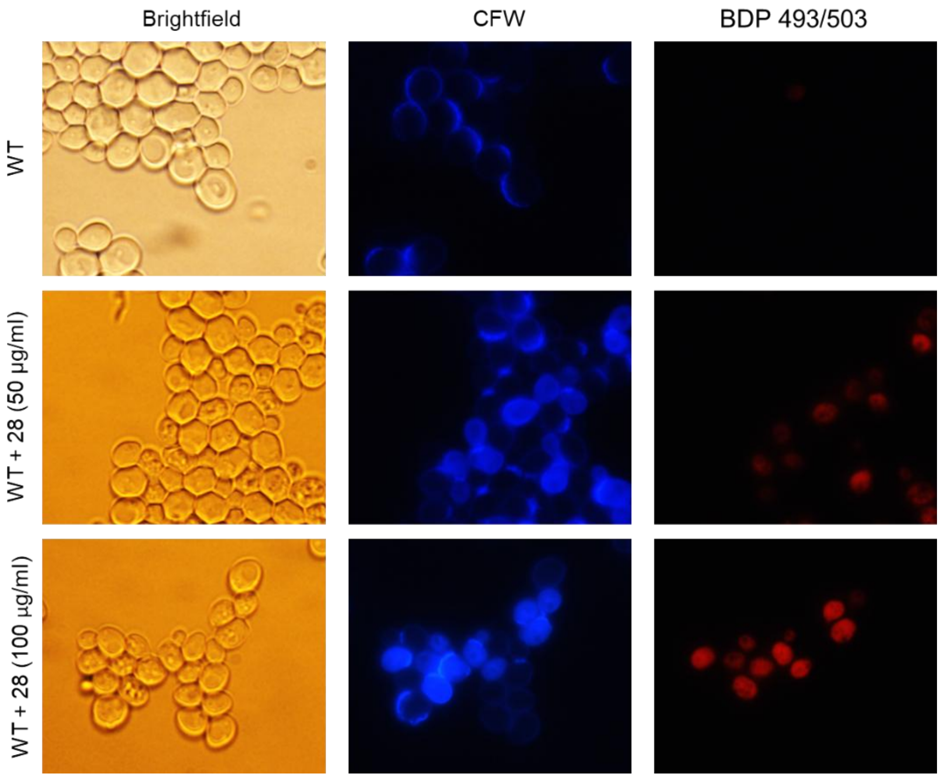

Supplementary Figure S5. Late-stage cellular effects of compound 28 in S. cerevisiae BY4742 after 16 h of exposure. Cells were incubated with compound 28 at 50 µg/mL (1× MIC) and 100 µg/mL (2× MIC) for 16 h and stained with CFW and PI. In contrast to the 4-h treatment (Fig. 7), where the plasma membrane remained intact (PI^{-}), extended incubation led to pronounced PI uptake, indicative of membrane permeabilization. CFW staining was also increased, and cells exhibited loss of rounded morphology, reduced size, and granular cytoplasm. Untreated WT served as control.

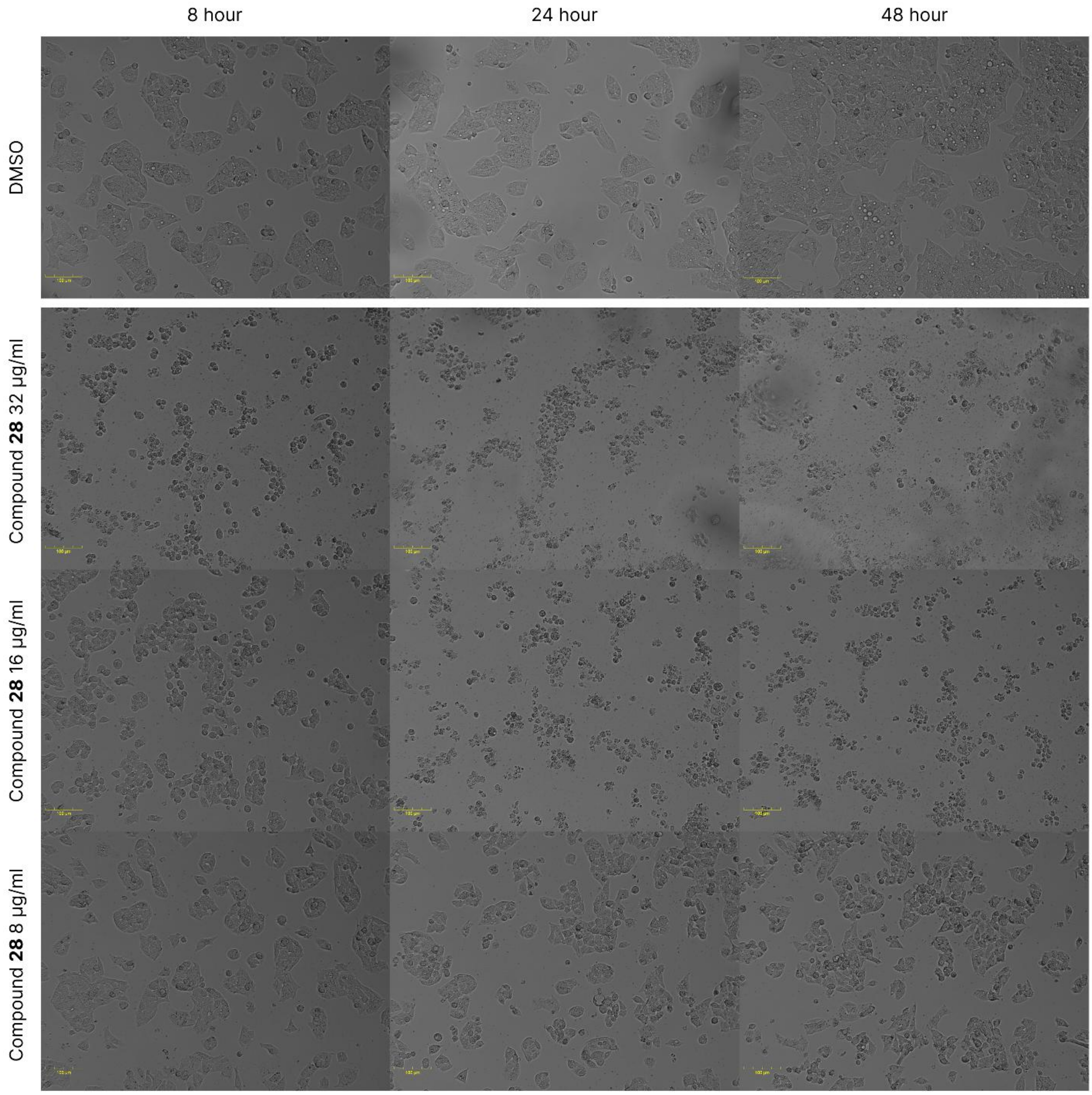

Supplementary Figure S6. Representative phase-contrast images of cells treated with DMSO or compound **28** at 8, 16, and 32 µg/mL for 8, 24, and 48 h. Scale bars are shown in the images.

1-amino-2-(2,4-difluorophenyl)-3-(1H-1,2,4-triazol-1-yl)propan-2-ol (2a) [25,31]

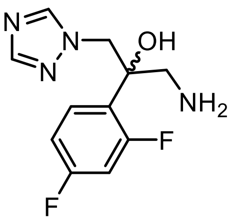

The amino alcohol **2a** has previously been prepared via azide intermediates as reported in the literature [25,31]. In the present work, compound **2a** was obtained by a milder and safer epoxide ring-opening approach using aqueous ammonia. To a solution of 1-((2-(2,4-difluorophenyl)oxiran-2-yl)methyl)-1H-1,2,4-triazole (1, 11.8 g, 49 mmol) in i-PrOH (250 mL) was added concentrated aqueous ammonia (25 wt%, 25 mL, ca. 0.30 mol). The reaction mixture was stirred for 10 h at ambient temperature. After completion, the solvent was removed under reduced pressure. The crude residue was purified by column chromatography on silica gel using a gradient elution of CH_2Cl_2/MeOH/aqueous NH_3 as follows (50:0:0 – 25:1:0 – 10:1;0.1 – 5:1:0.1). This procedure afforded **2a** as a colorless oil (7.59 g, 74% yield) along with (Z)-2-(2,4-difluorophenyl)-3-(1H-1,2,4-triazol-1-yl)prop-2-en-1-ol (**3**) as a minor by-product obtained as an oil (0.32 g, 1.35 mmol, 3%).

^{1}H NMR (600 MHz, $CDCl_3$) δ = 8.13 (s, 1H), 7.90 (s, 1H), 7.52 (m, 1H), 6.80 (m, 2H), 4.76 (m, 3H), 3.68 (d, J=12.72, 1H), 3.54 (d, J=12.72, 1H), 2.91 (d, 2H, $^3J_{HH}$ = 1.10). **^{13}C NMR (**100 MHz, $CDCl_3$) δ = 163.9, 157.5, 151.1, 144.5, 130.3, 124.3, 111.3, 104.4, 74.4, 55.7, 47.1 **ESI-MS (m/z):** $C_{11}H_{12}F_2N_4O$ = 255.0 $[M+H]^+$ Spectral data in according with previously described [31].

2-(2,4-difluorophenyl)-1-(methylamino)-3-(1H-1,2,4-triazol-1-yl)propan-2-ol (2b) [32]

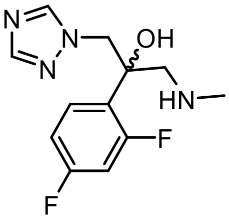

Compound **2b** was synthesized from the corresponding epoxide according to a literature procedure [32] with minor modifications. To a solution of **1** (1.75 g, 5.0 mmol) in anhydrous iso-propanol (25 mL) was added methylamine (1.39 mL, 33% solution in water, 10 mmol). The reaction mixture was heated at 60 °C and stirred for 8 h. Reaction progress was monitored by TLC. After completion, the reaction mixture was concentrated under reduced pressure. The residue was dissolved in ethyl acetate, and the resulting solution was subjected to purification by silica gel column chromatography using dichloromethane/methanol (25:1, v/v) as the eluent to afford compound 2b as a colorless oil in 87% yield.

^{1}H NMR (600 MHz, $CDCl_3$) δ = 8.1 (s, 1H), 7.7 (s, 1H), 7.5 (td, J=8.9, 6.5, 1H), 6.8 – 6.7 (m, 2H), 4.6 (d, J=14.3, 1H), 4.5 (d, J=14.2, 1H), 3.1 (d, J=12.4, 1H), 2.7 (d, J=12.4, 1H), 2.3 (s, 3H). **ESI-MS (m/z):** $C_{12}H_{14}F_2N_4O$ = 269.1 $[M+H]^+$ Spectral data in according with previously described[32].

2-(2,4-difluorophenyl)-1-(propylamino)-3-(1H-1,2,4-triazol-1-yl)propan-2-ol (2c) [53]

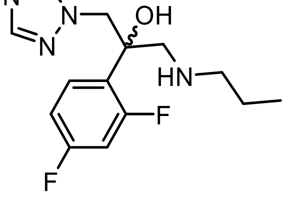

Compound **2c** was synthesized from the corresponding epoxide **1** and propyl amine according to a literature procedure [53] with minor modifications. Compound **2c** was prepared according to General Procedure **A**. Yield 78%.

^{1}H NMR (400 MHz, $CDCl_3$) δ = 8.1 (s, 1H), 7.8 (s, 1H), 7.5 (q, *J*=8.6, 1H), 6.8 – 6.7 (m, 2H), 4.6 – 4.4 (m, 2H), 3.1 (d, *J*=12.6, 1H), 2.8 (d, *J*=12.5, 1H), 2.4 (t, *J*=7.0, 2H), 1.4 – 1.3 (m, 2H), 0.8 (t, *J*=7.4, 3H). **^{13}C NMR** (101 MHz, $CDCl_3$) δ = 162.7 (dd, *J*=249.6, 12.1), 159.0 (dd, *J*=247.0, 11.7), 151.1, 144.6, 129.8 (dd, *J*=9.4, 6.1), 126.0 – 124.5 (m), 112.8 – 110.8 (m), 104.1 (t, *J*=26.3), 73.9 – 72.4 (m), 56.7 – 55.7 (m), 55.1 – 53.6 (m), 51.8, 22.9, 11.4. **ESI-MS (m/z):** $C_{14}H_{18}F_2N_4O$ = 297.1 $[M+H]^+$

2-(2,4-difluorophenyl)-3-(1H-1,2,4-triazol-1-yl)prop-2-en-1-ol (3) [54]

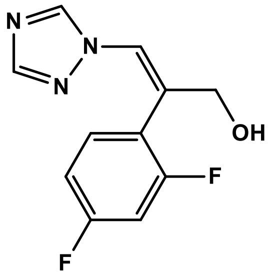

The structure of compound **3** was established based on the analysis of ^{1}H and ^{13}C NMR spectra. In the ^{1}H NMR spectrum, in addition to two singlets belonging to the triazole fragment (8.82 and 8.22 ppm) and characteristic multiplets of the 2,4-difluorophenyl ring (7.12 – 7.57 ppm), a singlet at 7.26 ppm (1 proton), a triplet at 5.06 (1 proton) and a doublet at 4.65 ppm (2 protons) are observed. The doublet and triplet correspond to the CH2-OH fragment. Addition of a small amount of D_2O to a solution of compound **3** leads to the disappearance of the triplet (due to the exchange of a hydroxyl proton for deuterium) and degeneration of the doublet of methylene protons into a singlet. This unambiguously proves that the triplet and doublet belong to the CH2-OH fragment. The singlet at 7.26 clearly belongs to the olefinic proton. This structure is also confirmed by ^{13}C NMR data. Thus, the chemical shift of 58.2 ppm corresponds to a methylene group with a hydroxyl substituent. The chemical shifts of the ^{13}C double bond resonances (123.2 and 130.0 ppm) are also fully consistent with the structure **3**.

^{1}H NMR (400 MHz, DMSO) δ = 8.82 (s, 1H), 8.22 (s, 1H), 7.51 – 7.57 (m, 1H), 7.27 – 7.33 (m, 1H), 7.13 – 7.17 (m, 1H), 5.06 (t, *J*=6.9, 1H), 4.65 (t, *J*=6.9, 2H).
^{13}C NMR (101 MHz, DMSO) δ 161.90 (dd, J = 246.4, 11.9 Hz), 159.91 (dd, J = 249.2, 12.9 Hz), 152.16, 145.69, 132.09 (dd, J = 9.7, 5.5 Hz), 129.96 , 123.31 (d, J = 2.8 Hz), 122.52 (dd, J = 14.7, 3.8 Hz), 111.45 (dd, J = 21.3, 3.6 Hz), 104.10 (t, J = 26.5 Hz), 58.16 (d, J = 2.3 Hz).
ESI-MS (m/z): $C_{11}H_9F_2N_3O$ = 238.0 $[M+H]^+$

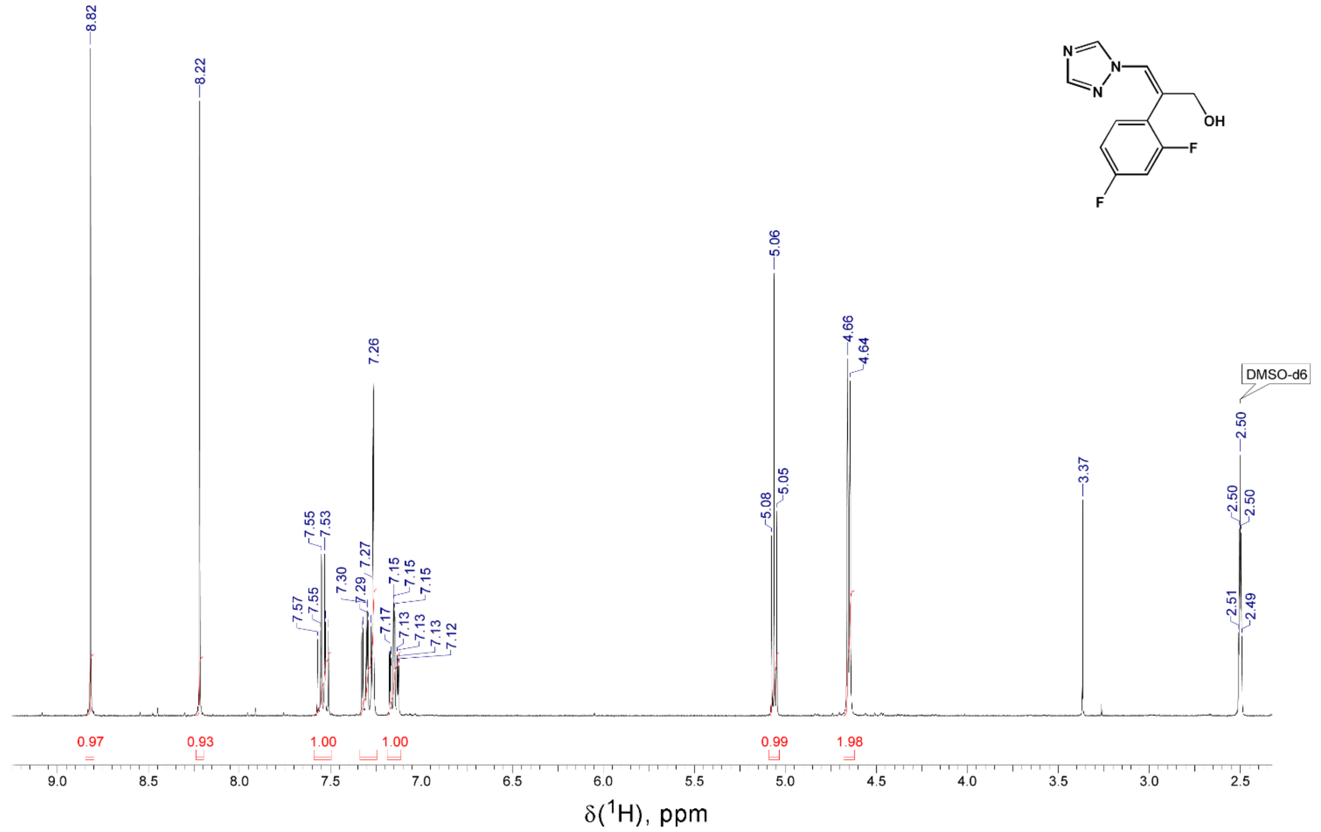

Supplementary Figure S7. 1D NMR ^{1}H spectrum of compounds **3** in DMSO-d$_6$ recorded at 400 MHz frequency and 25°C.

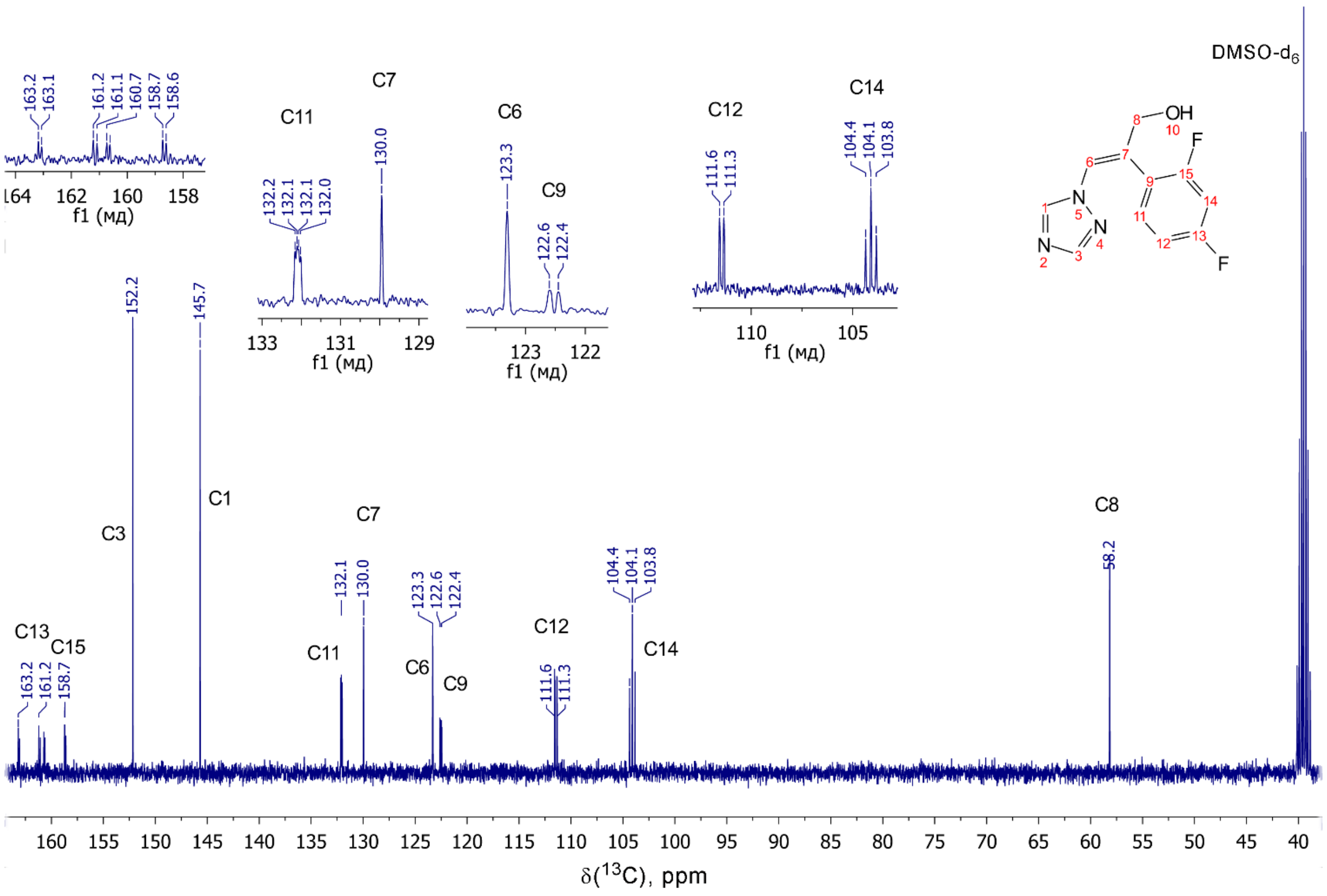

Supplementary Figure S8. 1D NMR ^{13}C spectrum of compounds **3** in DMSO-d$_6$ recorded at 400 MHz frequency and 25°C. Signal assignments are shown.

3-((1H-1,2,4-triazol-1-yl)methyl)-6-fluoro-1-propylindolin-3-ol (5)

Compound 5 was prepared according to General Procedure A, except that iso-propanol was used as the solvent and the reaction was conducted at 60°C for 16 h. Yield 8%.

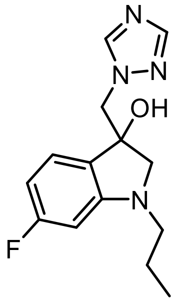

^{1}H NMR (600 MHz, $CDCl_3$) δ 7.97 (s, 1H), 7.82 (d, *J* = 2.7 Hz, 1H), 6.64 (dd, *J* = 8.1, 5.6 Hz, 1H), 6.20 (ddd, *J* = 9.2, 8.1, 2.2 Hz, 1H), 6.09 (dd, *J* = 10.4, 2.2 Hz, 1H), 4.37 – 4.27 (m, 2H), 3.45 (d, *J* = 10.3 Hz, 1H), 3.17 (d, *J* = 10.3 Hz, 1H), 2.98 (dt, *J* = 13.4, 7.5 Hz, 1H), 2.87 (ddd, *J* = 13.9, 7.9, 6.6 Hz, 1H), 1.52 – 1.44 (m, 2H), 0.85 (t, *J* = 7.4 Hz, 3H). **^{13}C NMR** (151 MHz, $CDCl_3$) δ 164.2 (d, *J*=246.4), 152.3, 150.3, 143.4, 124.2, 123.1, 102.8, 94.3, 76.8, 62.4, 55.1, 48.6, 19.3, 10.5. **ESI-MS (m/z):** $C_{14}H_{17}FN_4O$ = 277.1 $[M+H]^+$

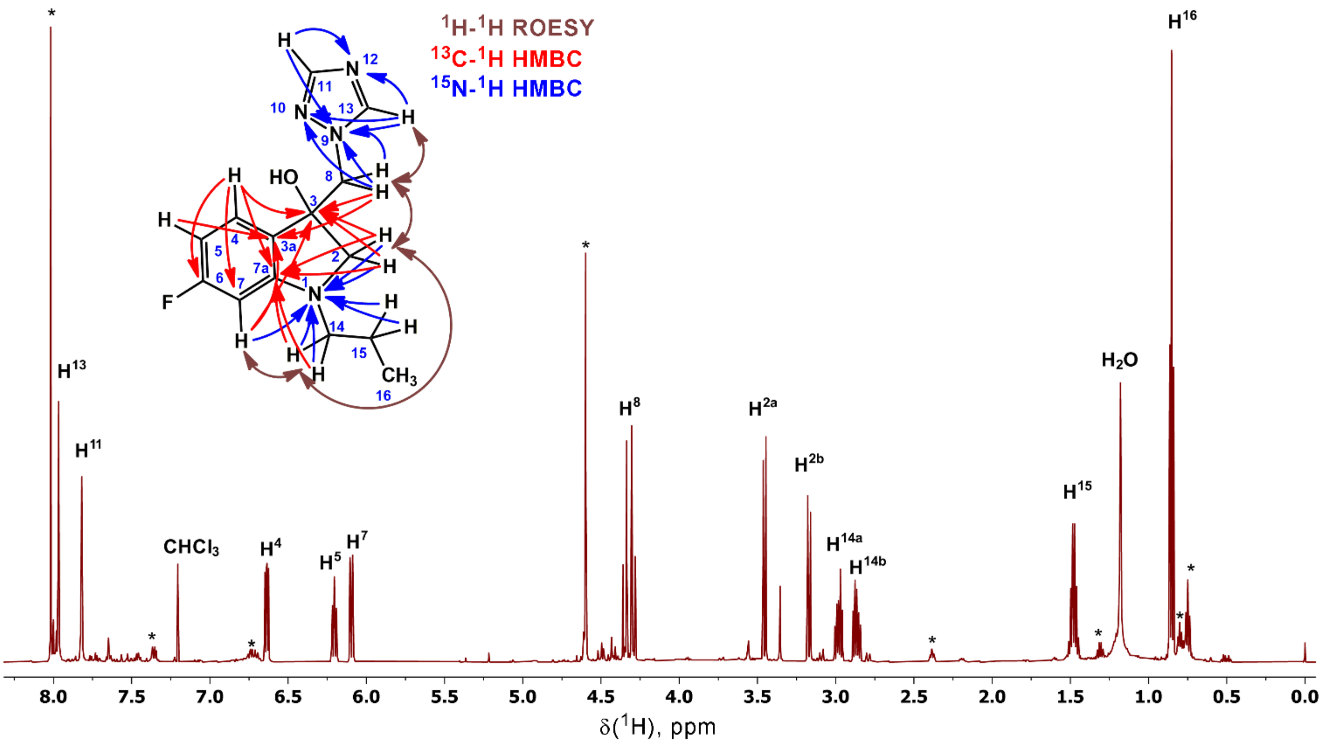

Supplementary Figure S9. 1D NMR ^{1}H spectrum of compounds 5 in $CDCl_3$ recorded at 600 MHz frequency and 25°C. Signal assignments are indicated. The signals of impurities are marked with an asterisk. The structural formula also shows correlations between the ^{1}H and ^{13}C nuclei, as observed in the ROESY (brown arrows), ^{13}C-^{1}H HMBC (red arrows), and ^{15}N-^{1}H HMBC (blue arrows) 2D NMR spectra.

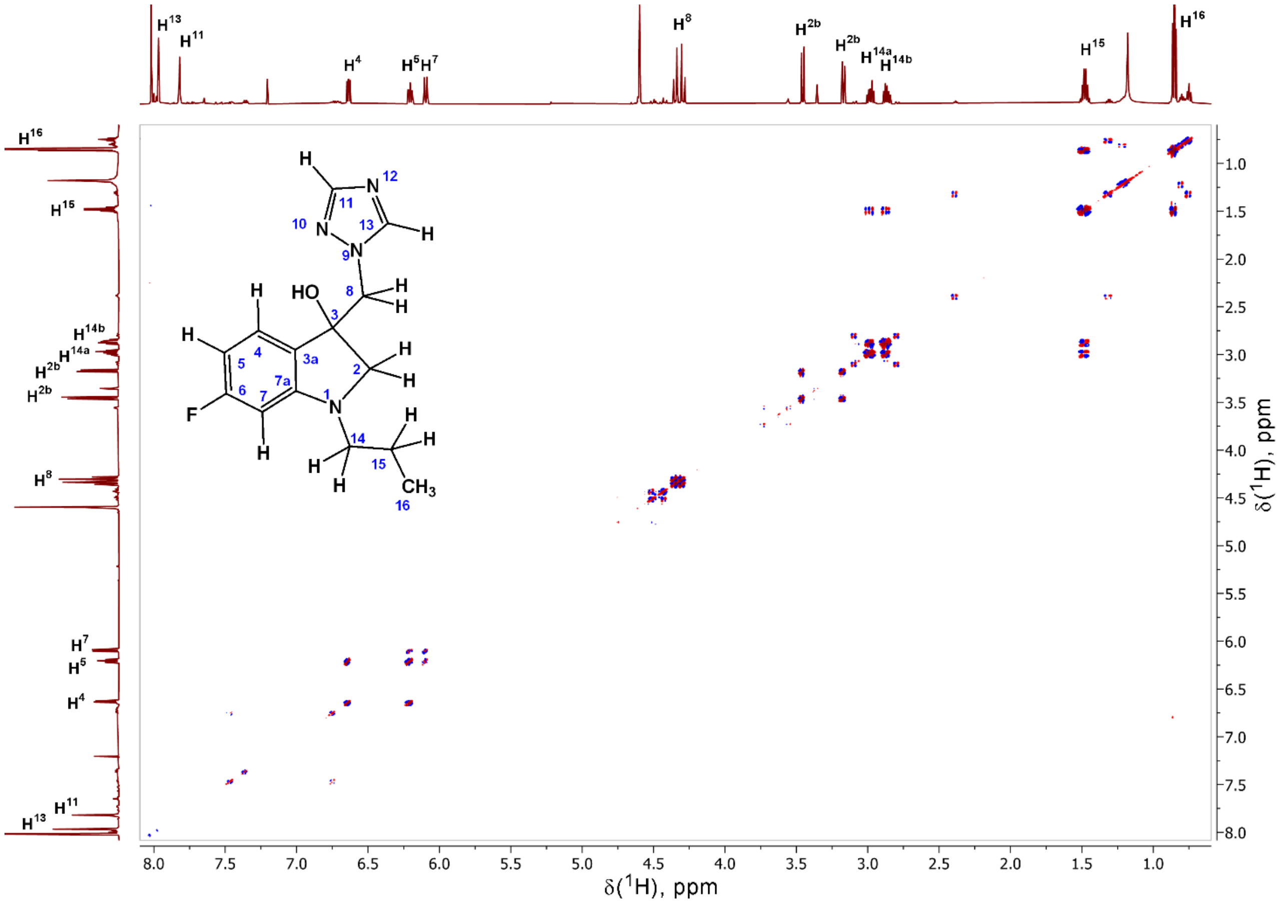

Supplementary Figure S10. 2D DQF COSY spectrum of the compound 5 recorded in $CDCl_3$ at 600 MHz and 298K.

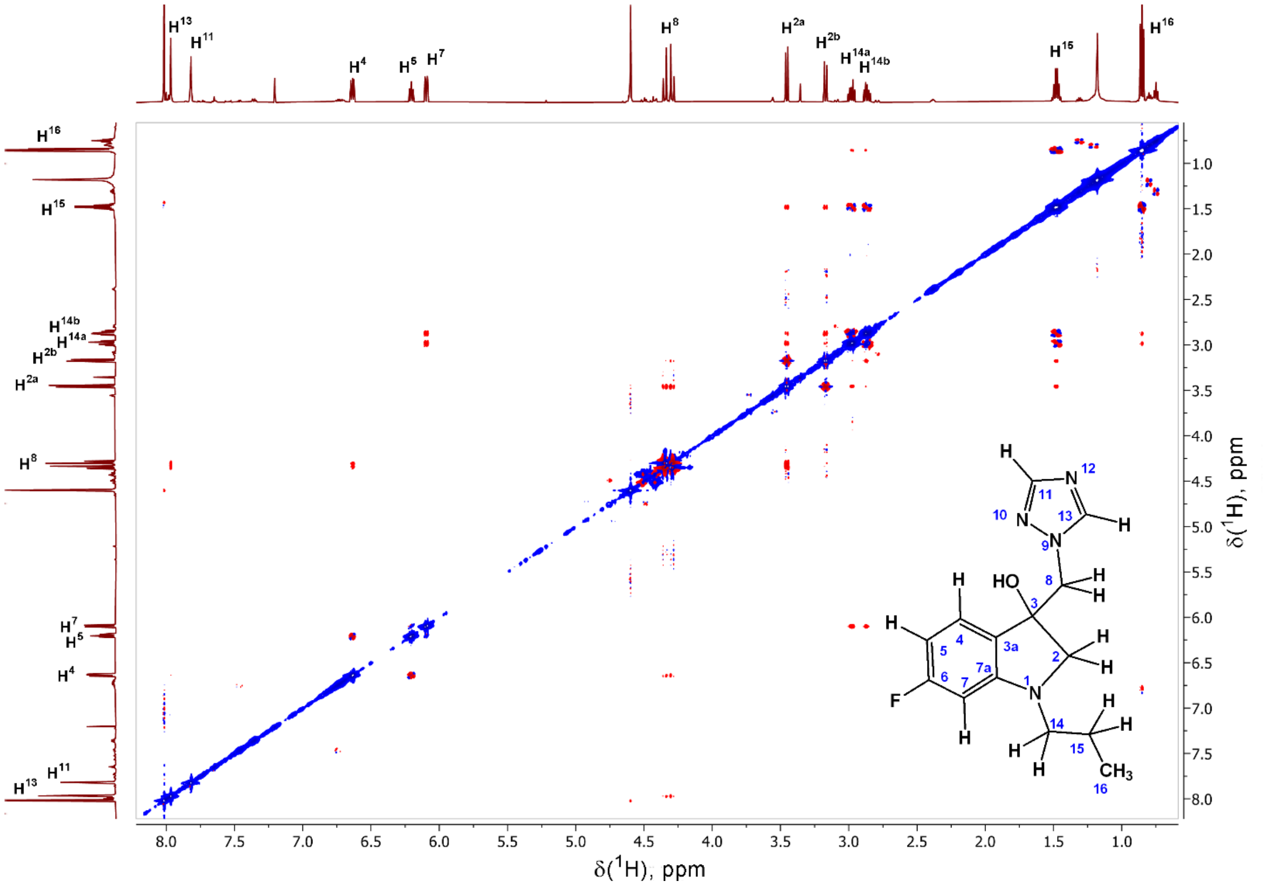

Supplementary Figure S11. 2D ROESY spectrum (mixing time 320 ms) of the compound 5 recorded in $CDCl_3$ at 600 MHz and 298K.

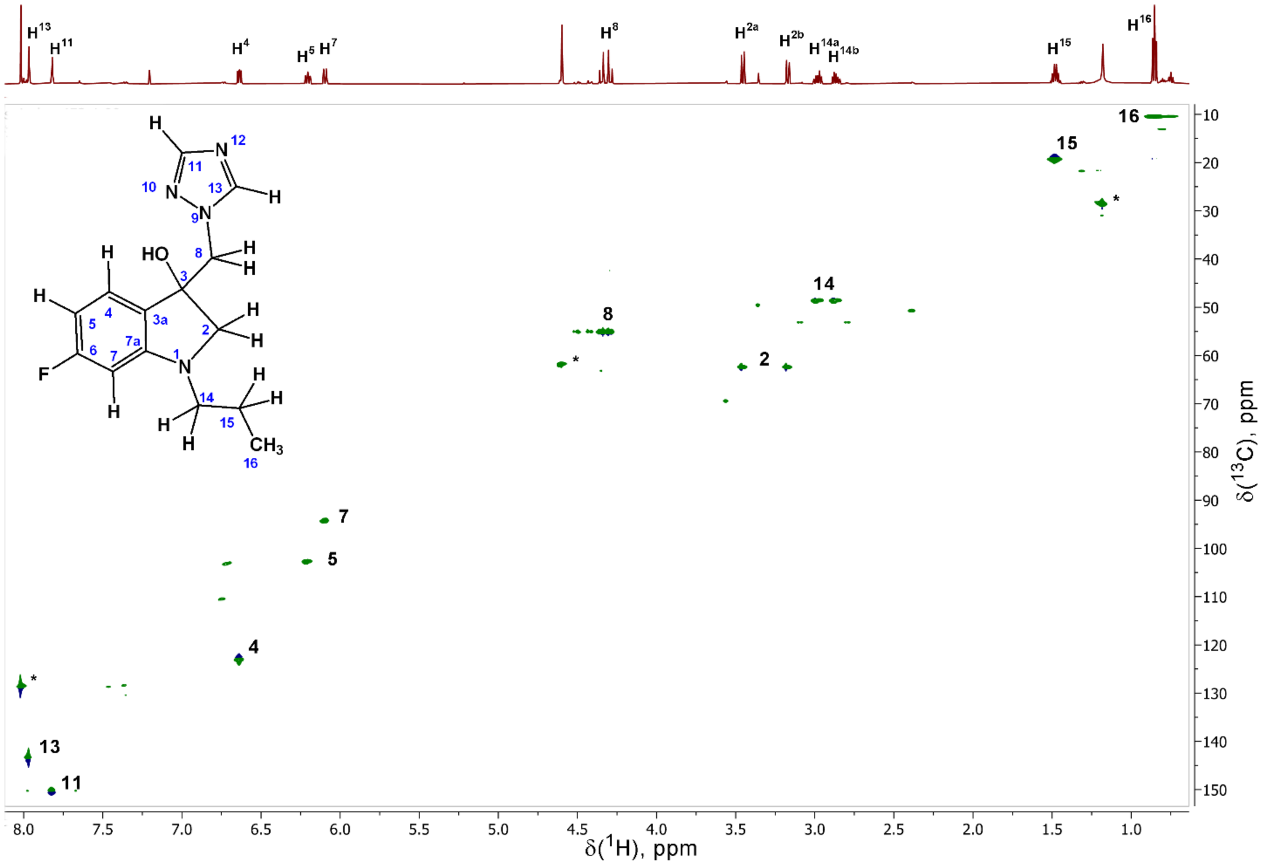

Supplementary Figure S12. 2D ^{13}C-^{1}H HSQC spectrum of the compound 5 recorded in $CDCl_3$ at 600 MHz and 298K.

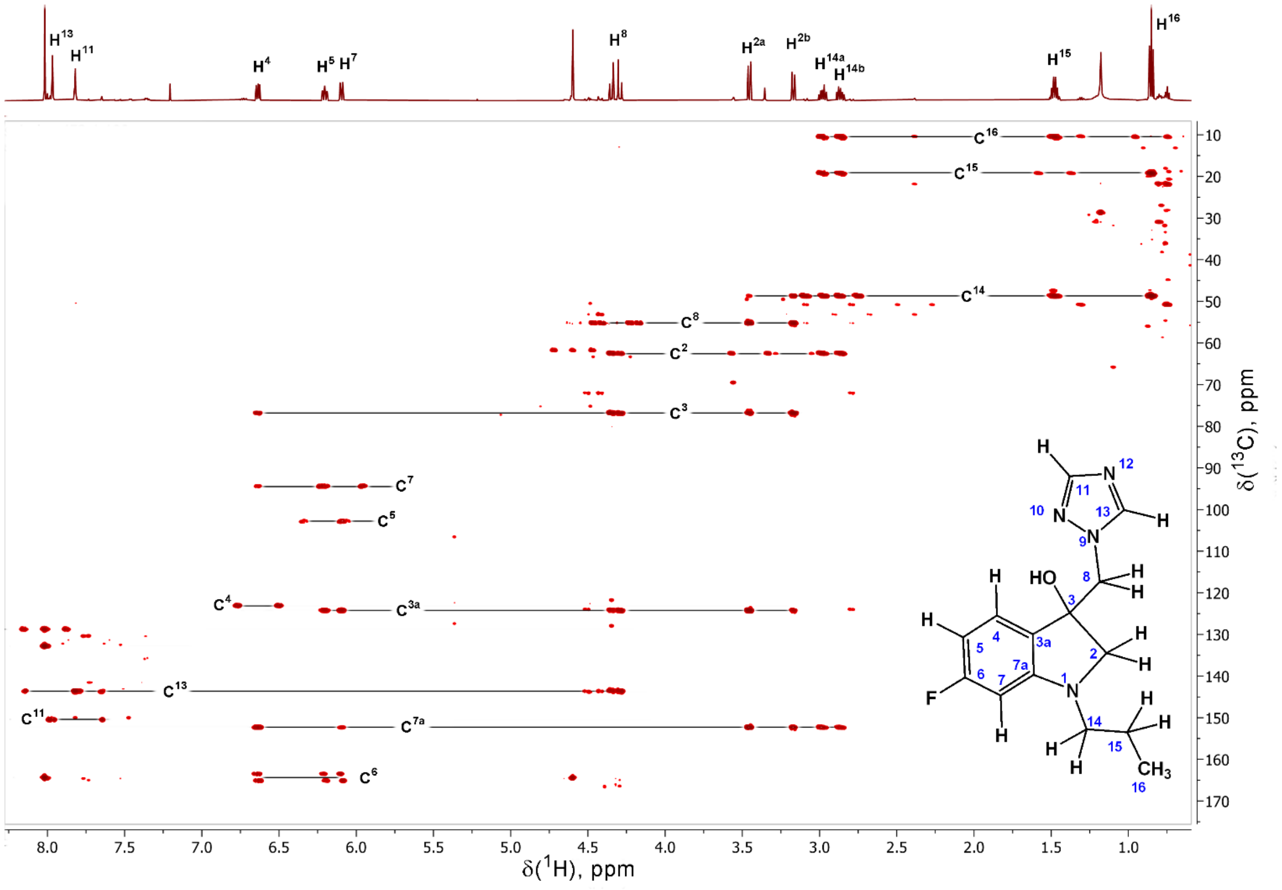

Supplementary Figure S13. 2D ^{13}C-^{1}H HMBC spectrum of the compound 5 recorded in $CDCl_3$ at 600 MHz and 298K.

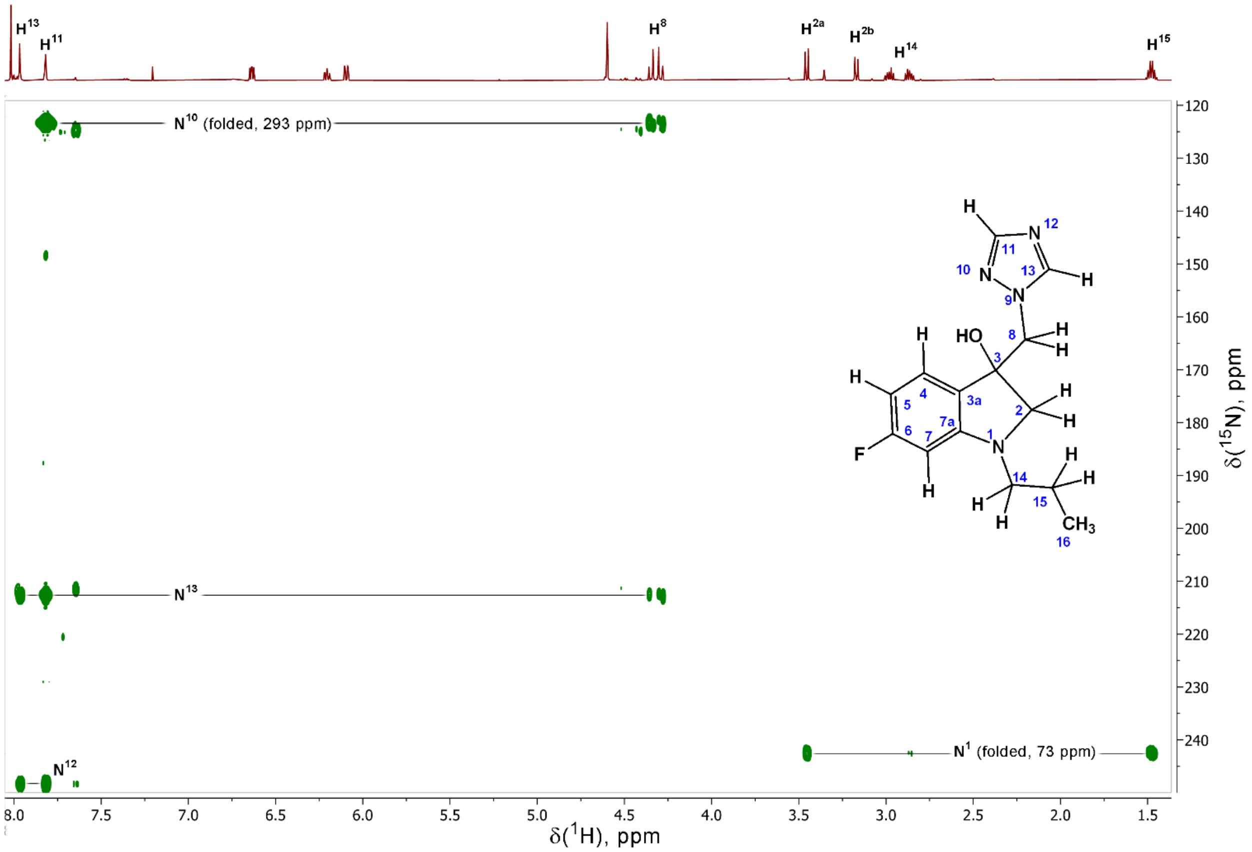

Supplementary Figure S14. 2D ^{15}N-^{1}H HMBC spectrum of the compound 5 recorded in $CDCl_3$ at 600 MHz and 298K.

1-(cyclopropylamino)-2-(2,4-difluorophenyl)-3-(1H-1,2,4-triazol-1-yl)propan-2-ol (2d) [55, 56]

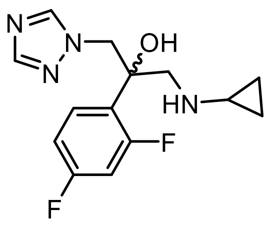

Compound **2d** was prepared from epoxide **1** and cyclopropylamine according to General Procedure A, except that the reaction was conducted in ethanol under reflux overnight. After completion of the reaction, the solvent was removed under reduced pressure. The residue was dissolved in dichloromethane and washed with aqueous citric acid, followed by water. The organic layer was dried over anhydrous sodium sulfate, filtered, and concentrated in vacuo. The crude product was purified by silica gel column chromatography (SiO_2, dichloromethane/ethyl acetate, 95:5, v/v) to afford compound **2d** as a light-yellow foam. Yield 75%.

^{1}**H NMR** (500 MHz, $CDCl_3$): 8.12 (s, 1H), 7.82 (s, 1H), 7.57 (m, 1H), 6.82 (m, 2H), 4.57 (dd, J_1=52.1 Hz, J_2=14.2 Hz, 2H), 3.98 (bs, 2H), 3.16 (d, J=12.6 Hz, 1H), 2.90 (d, J=12.6 Hz, 1H), 2.47 (m, 2H), 1.39 (m, 2H), 0.84 (t, J=7.3 Hz, 3H). **ESI-MS (m/z):** $C_{14}H_{16}F_2N_4O$ = 295.1 $[M+H]^+$ Spectral data in according with previously described[55].

tert-butyl 4-((2-(2,4-difluorophenyl)-2-hydroxy-3-(1H-1,2,4-triazol-1-yl)propyl)amino)piperidine-1-carboxylate (2e) [55]

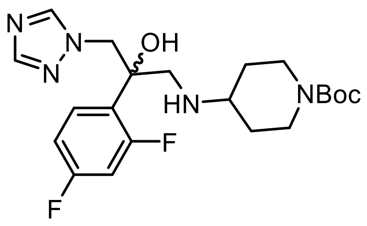

The compound **2e** has previously been prepared from **2a** and tert-butyl 4-oxopiperidine-1-carboxylate intermediates as reported in the literature[55]. In the present work, compound 2e was obtained according to General Procedure **A**. Yield 86%.

^{1}**H NMR** (600 MHz, $CDCl_3$) δ = 7.9 (s, 1H), 7.5 (s, 1H), 7.3 – 7.2 (m, 1H), 6.6 – 6.5 (m, 2H), 4.4 (d, *J*=14.3, 1H), 4.3 (d, *J*=14.3, 1H), 3.7 (s, 2H), 2.9 (d, *J*=12.5, 1H), 2.7 (d, *J*=12.5, 1H), 2.4 (s, 2H), 2.2 – 2.1 (m, 1H), 1.9 (s, 1H), 1.5 – 1.4 (m, 2H), 1.2 (s, 9H), 0.9 – 0.8 (m, 2H). ^{13}**C NMR** (151 MHz, $CDCl_3$) δ = 162.3 (dd, *J*=249.3, 12.1), 158.7 (dd, *J*=247.2, 11.7), 154.2, 150.6, 144.3, 130.0 – 129.3 (m), 124.8 (dd, *J*=13.2, 3.7), 111.0 (dd, *J*=20.4, 3.2), 103.8 (t, *J*=26.4), 79.0, 73.0 (d, *J*=5.3), 55.6 (d, *J*=4.8), 54.7, 51.3 (d, *J*=4.2), 42.1 (d, *J*=92.9), 32.2 (d, *J*=39.3), 28.0. **ESI-MS (m/z):** $C_{21}H_{29}F_2N_5O_3$= 438.2 $[M+H]^+$

tert-butyl 4-(4-((2-(2,4-difluorophenyl)-2-hydroxy-3-(1H-1,2,4-triazol-1-yl)propyl)amino)phenyl)piperazine-1-carboxylate (2f)

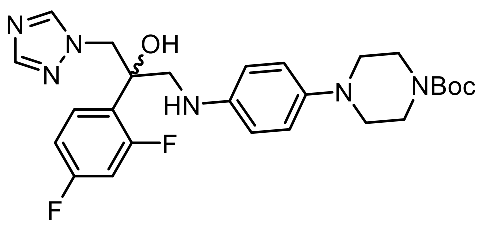

Compound **2f** was prepared according to General Procedure **A**. Yield 44%.

^{1}H NMR (600 MHz, DMSO) δ = 8.3 (s, 1H), 7.7 (s, 1H), 7.4 (q, *J*=7.7, 1H), 7.1 (t, *J*=7.7, 1H), 6.9 (td, *J*=8.5, 2.5, 1H), 6.7 (d, *J*=8.2, 2H), 6.5 (d, *J*=8.2, 2H), 6.0 (s, 1H), 4.9 (s, 1H), 4.7 (d, *J*=14.2, 1H), 4.6 (d, *J*=14.2, 1H), 3.5 – 3.5 (m, 1H), 3.4 – 3.4 (m, 5H), 2.8 (s, 4H), 1.4 (s, 9H). **^{13}C NMR** (151 MHz, DMSO) δ = 161.7 (dd, *J*=245.6, 12.6), 159.0 (dd, *J*=247.0, 12.1), 153.8, 150.5, 144.9, 143.3, 142.7, 130.4 – 130.0 (m), 125.3 (d, *J*=12.9), 118.5, 110.8 (d, *J*=20.9), 104.9 – 102.3 (m), 78.9, 74.8 (d, *J*=4.5), 55.2 (d, *J*=3.9), 51.2, 50.5, 44.4 – 42.4 (m), 40.1, 28.1. **ESI-MS (m/z):** Found mass for $C_{26}H_{32}F_2N_6O_3$ = 515.2 $[M+H]^+$.

tert-butyl 4-(((2-(2,4-difluorophenyl)-2-hydroxy-3-(1H-1,2,4-triazol-1-yl)propyl)amino)methyl)piperidine-1-carboxylate (2g) [36]

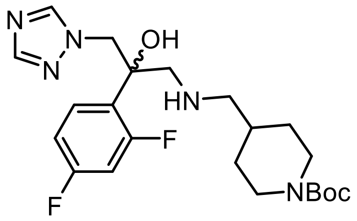

The compound 2g has previously been prepared from 2a and tert-butoxycarbonyl-4-formyl piperidine intermediates as reported in the literature **[1]**. In the present work, compound **2g** was prepared according to General Procedure **A**. Yield 86%.

^{1}H NMR (600 MHz, $CDCl_3$) δ = 8.1 (s, 1H), 7.8 (s, 1H), 7.6 (q, *J*=6.5, 1H), 6.9 – 6.8 (m, 1H), 6.8 – 6.8 (m, 1H), 4.6 (d, *J*=14.3, 1H), 4.5 (d, *J*=14.3, 1H), 4.2 – 4.0 (m, 2H), 3.2 (d, *J*=12.6, 1H), 3.0 – 2.9 (m, 1H), 2.7 – 2.6 (m, 2H), 2.4 (d, *J*=6.7, 2H), 1.6 (d, *J*=13.1, 2H), 1.5 (s, 1H), 1.4 (s, 9H), 1.0 (d, *J*=12.9, 2H). **^{13}C NMR** (151 MHz, $CDCl_3$) δ = 163.0 (dd, *J*=251.2, 11.8), 158.8 (dd, *J*=246.2, 11.7), 154.9, 151.6, 145.0, 130.1 – 129.7 (m), 125.1 – 124.9 (m), 111.8 (d, *J*=20.6), 104.4 (t, *J*=26.4), 79.6, 73.3 (d, *J*=5.6), 56.1 (d, *J*=4.2), 55.8, 54.6 (d, *J*=3.7), 44.8 – 42.6 (m), 36.2, 30.1, 28.6. **ESI-MS (m/z):** $C_{22}H_{31}F_2N_5O_3$ = 452.2 $[M+H]^+$

4-((2-(2,4-difluorophenyl)-2-hydroxy-3-(1H-1,2,4-triazol-1-yl)propyl)amino)piperidin-1-ium chloride (4e) [55]

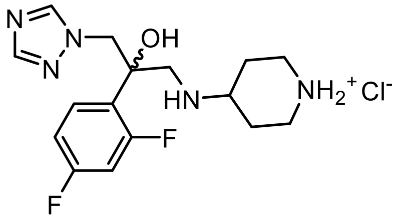

Compound **4e** was prepared according to General Procedure **B**. Yield 92%.

1H NMR (400 MHz, DMSO) δ = 8.3 (s, 1H), 7.7 (s, 1H), 7.4 (td, J=8.9, 6.8, 1H), 7.1 (ddd, J=11.9, 9.1, 2.6, 1H), 6.9 (td, J=8.5, 2.6, 1H), 4.5 (s, 2H), 2.9 – 2.8 (m, 1H), 2.8 – 2.5 (m, 4H), 2.1 (t, J=11.4, 2H), 1.7 – 1.5 (m, 2H), 1.2 (qt, J=9.5, 4.6, 2H). **^{13}C NMR** (101 MHz, DMSO) δ = 162.0 (dd, *J*=245.5, 12.5), 159.4 (dd, *J*=246.6, 12.2), 150.8, 145.3, 130.1 (dd, *J*=9.6, 6.5), 126.8 (dd, *J*=13.1, 3.6), 111.1 (d, *J*=20.3), 104.9 – 103.7 (m), 74.8 (m), 63.7 (d, *J*=3.2), 56.1, 53.7 (d, *J*=5.8), 48.1, 34.0 **ESI-MS (m/z):** Found mass for $C_{16}H_{22}F_2N_5O$= 338.1 $[M+1]^+$

4-(4-((2-(2,4-difluorophenyl)-2-hydroxy-3-(1H-1,2,4-triazol-1-yl)propyl)amino)phenyl)piperazin-1-ium chloride (4f)

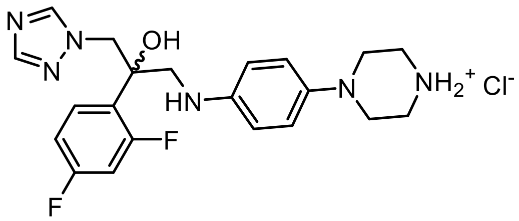

Compound **4f** was prepared according to General Procedure **B**. Yield 91%.

^{1}H NMR (600 MHz, DMSO) δ = 8.8 (s, 2H), 8.3 (s, 1H), 7.8 (s, 1H), 7.5 – 7.3 (m, 1H), 7.2 – 7.1 (m, 1H), 7.0 – 6.9 (m, 1H), 6.8 (d, *J*=8.3, 2H), 6.6 (d, *J*=8.3, 2H), 4.7 – 4.6 (m, 2H), 3.5 (d, *J*=13.3, 1H), 3.4 (d, *J*=13.3, 1H), 3.2 (s, 4H), 3.2 – 3.1 (m, 4H). **^{13}C NMR** (151 MHz, DMSO) δ = 161.8 (dd, *J*=245.4, 12.4), 159.2 (dd, *J*=245.8, 13.2), 150.5, 144.9, 143.0, 141.9, 130.3, 125.1, 118.4, 114.0, 110.8 (d, *J*=20.2), 104.4 – 103.0 (m), 74.7, 55.2, 51.4, 47.4, 43.0, 40.1.**ESI-MS (m/z):** Found mass for $C_{21}H_{25}F_2N_6O$= 415.1 $[M+1]^+$

4-(((2-(2,4-difluorophenyl)-2-hydroxy-3-(1H-1,2,4-triazol-1-yl)propyl)amino)methyl)piperidin-1-ium chloride (4g) [32]

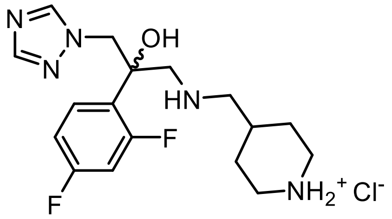

Compound **4g** was prepared according to General Procedure **B**. Yield 75%.

^{1}H NMR (600 MHz, DMSO) δ = 8.8 – 8.7 (m, 1H), 8.6 – 8.5 (m, 1H), 8.5 – 8.4 (m, 2H), 8.3 (s, 1H), 7.8 (s, 1H), 7.5 – 7.4 (m, 1H), 7.3 – 7.2 (m, 1H), 7.1 – 7.0 (m, 1H), 4.7 – 4.6 (m, 2H), 3.6 – 3.5 (m, 1H), 3.4 – 3.3 (m, 1H), 3.3 (d, *J*=13.2, 2H), 2.9 – 2.8 (m, 4H), 2.0 – 1.9 (m, 1H), 1.8 (d, *J*=17.1, 2H), 1.4 – 1.3 (m, 2H). **^{13}C NMR** (151 MHz, DMSO) δ = 162.6 (dd, *J*=247.4, 12.6), 159.6 (dd, *J*=248.2, 13.8), 151.0, 145.3, 130.3, 122.6 (d, *J*=13.1), 111.4 (d, *J*=20.3), 104.5 (t, *J*=26.6), 72.1 (d, *J*=4.4), 54.9, 52.3, 42.5, 40.1, 29.8, 26.0 (d, *J*=6.1). **ESI-MS (m/z):** Found mass for $C_{17}H_{24}F_2N_5O$= 352.1 $[M+1]^+$

tert-butyl (Z)-((1-(2-(5-(4-chlorobenzylidene)-2,4-dioxothiazolidin-3-yl)acetyl)piperidin-4-yl)methyl)carbamate (11)

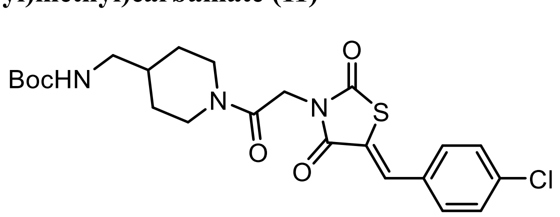

Compound **11** was prepared according to General Procedure **B**. Yield 63%.

^{1}H NMR (600 MHz, $CDCl_3$) δ = 7.9 (s, 1H), 7.4 (s, 4H), 4.7 (s, 1H), 4.6 – 4.5 (m, 3H), 3.8 (d, *J*=13.4, 1H), 3.2 – 3.1 (m, 2H), 3.1 – 3.0 (m, 1H), 2.6 (t, *J*=12.9, 1H), 1.8 (d, *J*=13.0, 1H), 1.7 (d, *J*=13.0, 2H), 1.7 – 1.5 (m, 2H), 1.4 (s, 9H), 1.3 – 1.2 (m, 1H), 1.2 – 1.1 (m, 1H). **^{13}C NMR** (151 MHz, $CDCl_3$) δ = 167.7, 166.1, 162.6, 156.2, 136.8,

132.9, 131.9, 131.4, 129.7, 122.3, 79.6, 45.9, 44.9, 42.7, 42.6, 36.8, 30.1, 29.2, 28.5. **ESI-MS (m/z):** Found mass for $C_{23}H_{28}ClN_3O_5S$= 494.1 $[M+1]^+$

tert-butyl 4-((2-(2,4-difluorophenyl)-2-hydroxy-3-(1H-1,2,4-triazol-1-yl)propyl)(methyl)amino)piperidine-1-carboxylate (12e) [55]

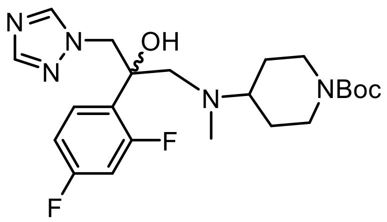

Compound **12e** was prepared according to General Procedure **D**. Yield 59%.

^{1}**H NMR** (500 MHz, DMSO-d6): 9.48 (bs, 1H), 9.25 (bs, 1H), 9.10 (bs, 1H), 8.61 (s, 1H), 7.86 (s, 1H), 7.49 (m, 1H), 7.31 (m, 1H), 7.07 (m, 1H), 4.83 (m, 1H), 4.73 (m, 1H), 3.38 (m, 4H), 3.14 (m, 2H), 2.88 (m, 2H), 2.71 (m, 1H), 2.42 (m, 1H), 2.18 (m, 2H), 1.90 (s, 3H). **ESI-MS (m/z):** Found mass for $C_{22}H_{31}ClF_2N_5O_3$= 452.1 $[M+1]^+$

tert-butyl 4-(4-((2-(2,4-difluorophenyl)-2-hydroxy-3-(1H-1,2,4-triazol-1-yl)propyl)(methyl)amino)phenyl)piperazine-1-carboxylate (12f)

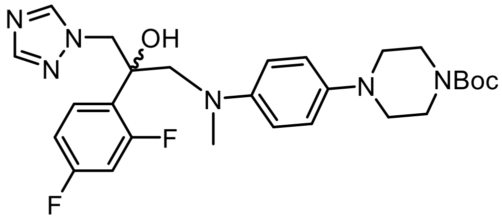

Compound **12f** was prepared according to General Procedure **D**. Yield 47%.

^{1}**H NMR** (600 MHz, DMSO) δ = 8.3 (s, 1H), 7.7 (s, 1H), 7.4 – 7.3 (m, 1H), 7.2 – 7.1 (m, 1H), 7.0 – 6.9 (m, 1H), 6.8 (d, *J*=8.9, 2H), 6.7 (d, *J*=8.9, 2H), 5.9 (s, 1H), 4.8 (d, *J*=14.3, 1H), 4.6 (d, *J*=14.3, 1H), 3.7 (d, *J*=15.3, 1H), 3.6 (d, *J*=15.3, 1H), 3.5 – 3.4 (m, 4H), 2.9 – 2.8 (m, 4H), 2.8 (s, 3H), 1.4 (s, 9H). ^{13}**C NMR** (151 MHz, DMSO) δ = 161.8 (dd, *J*=245.8, 12.5), 158.9 (dd, *J*=246.2, 12.1), 153.8, 150.5, 145.0 (d, *J*=10.0), 142.7, 130.4 – 129.9 (m), 125.4 (dd, *J*=13.2, 3.6), 118.1, 114.6, 113.6, 110.9 (d, *J*=20.2), 103.8 (t, *J*=27.0), 78.9, 76.2 (d, *J*=5.7), 60.4, 54.9 (d, *J*=6.1), 50.3, 43.3 (d, *J*=144.9), 40.2, 28.1.**ESI-MS (m/z):** Found mass for $C_{27}H_{34}F_2N_6O_3$ = 528.3 $[M]^+$

tert-butyl 4-(((2-(2,4-difluorophenyl)-2-hydroxy-3-(1H-1,2,4-triazol-1-yl)propyl)(methyl)amino)methyl)piperidine-1-carboxylate (12g) [32]

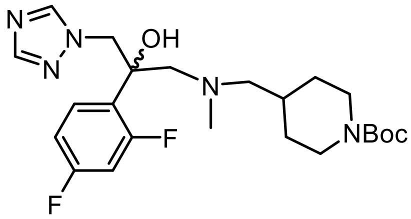

Compound **12g** was prepared according to General Procedure **D**. Yield 62%.

^{1}**H NMR** (600 MHz, $CDCl_3$) δ = 8.1 (s, 1H), 7.7 (s, 1H), 7.5 (d, *J*=8.8, 1H), 6.8 – 6.6 (m, 2H), 4.5 (d, *J*=14.3, 1H), 4.4 (d, *J*=14.3, 1H), 4.1 – 3.9 (m, 2H), 3.0 (d, *J*=13.5, 1H), 2.7 (d, *J*=13.5, 1H), 2.6 – 2.5 (m, 2H), 2.2 – 2.1 (m, 1H), 2.1 – 2.0 (m, 1H), 2.0 (s, 3H), 1.5 (d, *J*=13.3, 1H), 1.5 (d, *J*=13.2, 1H), 1.4 (s, 9H), 1.3 – 1.3 (m, 1H), 0.9

– 0.8 (m, 2H). **^{13}C NMR** (151 MHz, $CDCl_3$) δ = 162.7 (dd, *J*=249.7, 12.0), 158.9 (dd, *J*=246.8, 11.7), 154.8, 150.9, 144.8, 129.6 (dd, *J*=9.4, 5.8), 127.1 – 125.5 (m), 111.5 (d, *J*=20.5), 104.3 (t, *J*=26.3), 79.4, 71.9 (d, *J*=5.5), 65.3, 62.7, 56.4 (d, *J*=4.9), 44.0, 43.6 – 42.7 (m), 34.2, 30.3, 28.4.**ESI-MS (m/z):** Found mass for $C_{23}H_{33}F_2N_5O_3$ = 466.3 $[M+H]^+$

tert-butyl 4-(4-(N-(2-(2,4-difluorophenyl)-2-hydroxy-3-(1H-1,2,4-triazol-1-yl)propyl)acetamido)phenyl) piperazine-1-carboxylate (14f)

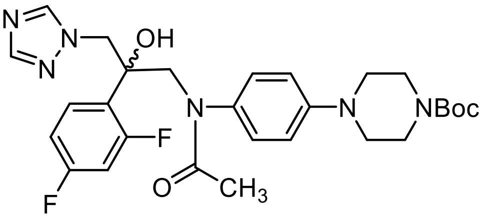

Compound **14f** was prepared according to General Procedure **E**. Yield 80%

^{1}H NMR (600 MHz, DMSO) δ = 8.3 (s, 1H), 7.7 (s, 1H), 7.4 (t, *J*=8.9, 1H), 7.0 – 6.9 (m, 2H), 6.8 – 6.6 (m, 4H), 6.4 (s, 1H), 4.5 (dd, *J*=21.1, 14.1, 2H), 4.4 (d, *J*=14.4, 1H), 3.9 (d, *J*=14.4, 1H), 3.5 – 3.4 (m, 4H), 3.1 – 3.0 (m, 4H), 1.7 (s, 3H), 1.4 (s, 9H). **^{13}C NMR** (151 MHz, DMSO) δ = 173.2, 161.9 (dd, *J*=246.1, 12.6), 158.8 (dd, *J*=246.5, 12.6), 153.9, 150.5, 149.6, 145.1, 135.1, 130.1, 127.9, 124.6 (d, *J*=12.1), 115.7, 110.8 (d, *J*=20.4), 103.6 (t, *J*=27.1), 79.0, 75.3 (d, *J*=5.0), 56.4, 55.5, 48.3 (d, *J*=96.9), 43.8 – 42.3 (m), 28.1, 22.4. **ESI-MS (m/z):** Found mass for $C_{28}H_{34}F_2N_6O_4$ = 557.3 $[M+H]^+$

tert-butyl 4-(4-(N-(2-(2,4-difluorophenyl)-2-hydroxy-3-(1H-1,2,4-triazol-1-yl)propyl)-4-fluorobenzamido) phenyl) piperazine-1-carboxylate (15f)

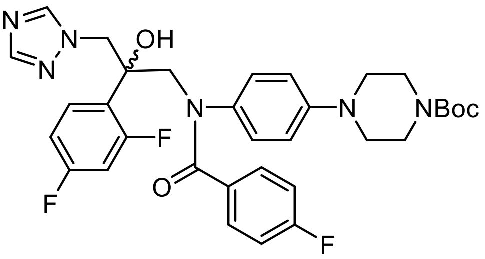

Compound **15f** was prepared according to General Procedure **E**. Yield 86%

^{1}H NMR (600 MHz, $CDCl_3$) δ = 8.3 (s, 1H), 7.8 (s, 1H), 7.7 (td, *J*=8.8, 6.5, 1H), 7.2 – 7.1 (m, 2H), 7.0 (s, 1H), 6.9 – 6.7 (m, 3H), 6.7 – 6.4 (m, 2H), 6.4 – 6.2 (m, 1H), 4.7 (d, *J*=14.1, 1H), 4.5 – 4.3 (m, 3H), 3.6 – 3.5 (m, 4H), 3.1 – 3.0 (m, 4H), 1.5 (s, 9H). **^{13}C NMR** (151 MHz, $CDCl_3$) δ = 172.9, 163.7 (d, *J*=252.2), 163.0 (dd, *J*=249.5, 12.3), 158.9 (dd, *J*=246.9, 11.8), 154.7, 151.1, 149.3, 145.1, 135.5, 131.7 (d, *J*=8.8), 130.8 – 130.3 (m), 130.1 (d, *J*=3.3), 127.7, 124.0 (d, *J*=11.1), 116.5, 115.1 (d, *J*=21.9), 111.5 (d, *J*=20.5), 103.7 (t, *J*=26.5), 80.3, 76.1 (d, *J*=4.6), 58.4 (d, *J*=3.6), 57.0 (d, *J*=5.1), 49.2, 44.8 – 42.3 (m), 28.5. **ESI-MS (m/z):** Found mass for $C_{33}H_{35}F_3N_6O_4$ = 637.1 $[M+H]^+$

tert-butyl 4-(4-(N-(2-(2,4-difluorophenyl)-2-hydroxy-3-(1H-1,2,4-triazol-1-yl)propyl)-4-(trifluoromethyl)benzamido)phenyl)piperazine-1-carboxylate (16f)

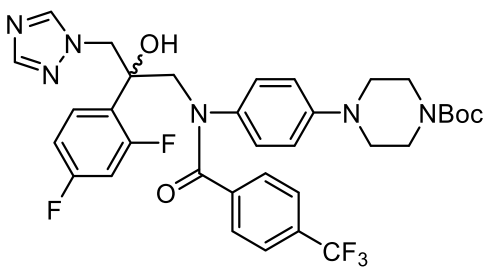

Compound **16f** was prepared according to General Procedure **E**. Yield 80%.

^{1}H NMR (600 MHz, $CDCl_3$) δ = 8.3 (s, 1H), 7.9 (s, 1H), 7.7 (td, *J*=8.9, 6.5, 1H), 7.4 (d, *J*=8.2, 2H), 7.3 (d, *J*=7.9, 2H), 6.8 (ddd, *J*=14.0, 7.1, 2.4, 1H), 7.0 – 5.8 (m, 4H), 6.4 (ddd, *J*=11.3, 8.5, 2.5, 1H), 4.7 (d, *J*=14.1, 1H), 4.5 – 4.4 (m, 3H), 3.6 – 3.5 (m, 4H), 3.1 – 3.0 (m, 4H), 1.5 (s, 9H). **^{13}C NMR** (151 MHz, $CDCl_3$) δ = 172.5, 163.1 (dd, *J*=249.9, 12.2), 159.0 (dd, *J*=246.9, 11.8), 154.7, 151.1, 149.3, 145.1, 137.8, 135.2, 132.1 (q, *J*=32.9), 130.4 (d, *J*=8.5), 129.5, 127.7, 125.0 (d, *J*=3.7), 124.4, 123.9 (d, *J*=14.8), 116.6, 111.6 (d, *J*=20.8), 103.8 (t, *J*=26.5), 80.3, 76.2 (d, *J*=4.6), 58.5, 57.0 (d, *J*=4.8), 49.3, 44.4 – 42.3 (m), 28.5. **ESI-MS (m/z):** Found mass for $C_{34}H_{35}F_5N_6O_4$ = 687.3 $[M+H]^+$

tert-butyl 4-(3-chloro-2-hydroxypropyl)piperazine-1-carboxylate (25) [36].

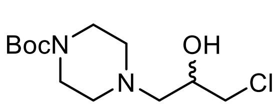

Compound **25** was previously reported. It was prepared according to General Procedure **F** from tert-butyl piperazine-1-carboxylate and epichlorohydrin in ethanol, following a literature procedure[36]. Yield: 86%.

^{1}H NMR (500 MHz, DMSO) δ = 1.42 (s, 9H), 2.27-2.42 (m, 6H), 3.22-3.32 (m, 4H), 3.52-3.56 (m, 1H), 3.79-3.89 (m, 1H), 5.04 (d, J = 4.9 Hz, 1H). **ESI-MS (m/z):** Found mass for $C_{12}H_{23}ClN_2O_3$ = 279.2 $[M+H]^+$ The spectral data were consistent with those previously reported[36].

1-(3-chloro-2-hydroxypropyl)piperidin-4-one (26) [33].

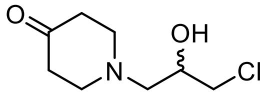

Compound 26 was prepared according to General Procedure F from piperidin-4-one and epichlorohydrin in distilled water, following a literature procedure. The spectral data were consistent with those previously reported [2,3]. The spectral data were consistent with those previously reported [2].

3-chloro-2-hydroxypropyl-(4-((2-(2,4-difluorophenyl)-2-hydroxy-3-(1H-1,2,4-triazol-1-yl)propyl)methylamino)piperidin-1-yl) (27)

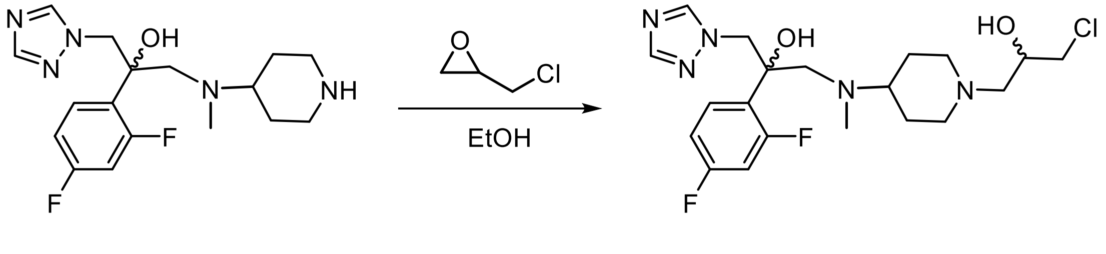

Compound **27** was prepared according to General Procedure **F** from **23** and epichlorohydrin after removed Boc protection from compound **12e**. Yield 76%.

tert-butyl-4-(3-(5-(4-chlorobenzylidene)-2,4-dioxothiazolidin-3-yl)-2-hydroxypropyl)piperazine-1-carboxylate (29) [36]

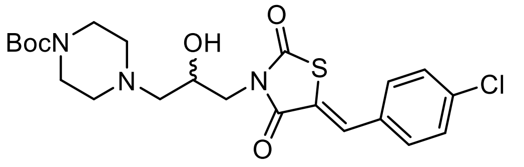

Compound **29** was previously described[36]. It was prepared according to General Procedure **G** and obtained as a colorless amorphous powder. Yield 36%.

^{1}H NMR (500 MHz, DMSO) δ = 1.32- 1.43 (m, 9H) 2.20-2.44 (m, 6H) 2.24-2.42 (m, 1H) 3.30 (br s, 4H) 3.63-3.76 (m, 2H) 3.89-4.04 (m, 1H) 5.06 (br d, J=4.77 Hz, 1H),7.56-7.69 (m, 4H) ,7.92 (s, 1H). **ESI-MS (m/z):** Found mass for $C_{22}H_{28}ClN_3O_5S$ = 481.8 $[M+H]^+$ The spectral data were consistent with those previously reported [36].

(Z)-5-(4-chlorobenzylidene)-3-(2-hydroxy-3-(4-oxopiperidin-1-yl)propyl)thiazolidine-2,4-dione (30)

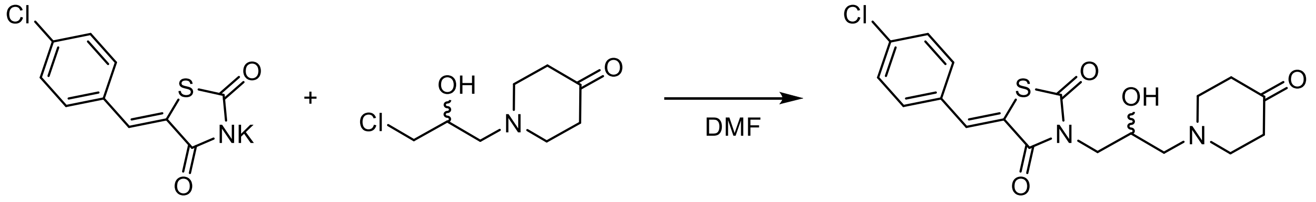

Compound **30** was prepared according to General Procedure **G** from potassium (Z)-5-(4-chlorobenzylidene)thiazolidine-2,4-dione and 1-(3-chloro-2-hydroxypropyl)piperidin-4-one (**25**). The product was obtained as a colorless amorphous powder. Yield 34%.

^{1}H NMR (500 MHz, DMSO) δ = 7.97 (s, 1H), 7.68 (d, J = 8.6 Hz, 2H), 7.58 (d, J = 8.6 Hz, 2H), 5.10 (s, J = 75.4 Hz, 1H), 3.94 (d, J = 5.8 Hz, 1H), 3.68 (d, J = 6.3 Hz, 2H), 2.75 (m, 2H), 2.65 (d, J = 10.9 Hz, 1H), 2.49 (d, J = 1.6 Hz, 2H), 2.46 – 2.42 (m, 2H), 2.28 – 2.25 (m, 4H) **ESI-MS (m/z):** $C_{18}H_{19}ClN_2O_4S$ = 395 $[M+H]^+$

(Z)-2-(4-chlorobenzylidene)-4-(4-((2-(2,4-difluorophenyl)-2-hydroxy-3-(1H-1,2,4-triazol-1-yl)propyl)(methyl)amino)piperidine-1-carbonyl)-6-hydroxy-1,4-thiazepan-3-one (34)

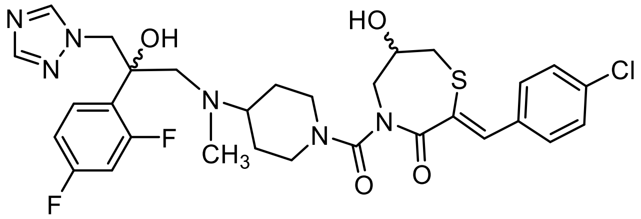

Compound **34** was prepared according to General Procedure **H.** Yield 37%.

ESI-MS (m/z): $C_{30}H_{33}ClF_2N_6O_4S$ = 647.1 $[M+H]^+$

NMR data – see table S3.

(Z)-2-(4-chlorobenzylidene)-4-(4-(2-(2,4-difluorophenyl)-2-hydroxy-3-(1H-1,2,4-triazol-1-yl)propyl)piperazine-1-carbonyl)-6-hydroxy-1,4-thiazepan-3-one (35)

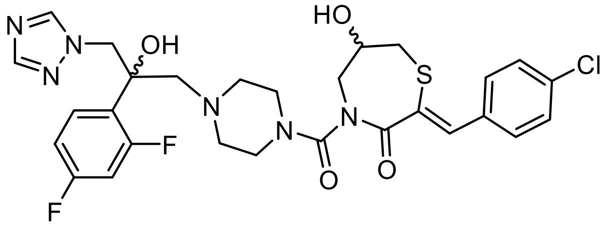

Compound **35** was prepared according to General Procedure **H.** Yield 43%.

ESI-MS (m/z): $C_{28}H_{29}ClF_2N_6O_4S$ = 619.1 $[M+H]^+$

NMR data – see table S3.

(Z)-2-(4-chlorobenzylidene)-6-hydroxy-1,4-thiazepan-3-one (36)

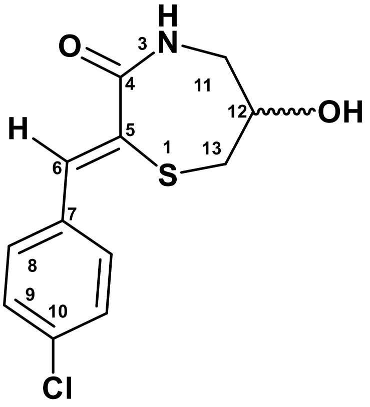

Compound **36** was prepared according to General Procedure **H.** Yield 79%.

ESI-MS (m/z): $C_{12}H_{12}ClNO_2S$ = 269.9 $[M]^+$
NMR data – see table S3
Crystallographic data Supplementary Fig S2 and Supplementary Tabel S1.

Identification of the seven-membered thiazepane ring in compounds 34–36.
The structures of compounds **34** and **35** were determined by NMR spectroscopy. Two-dimensional DQF-COSY and ROESY spectra, as well as heteronuclear ^{13}C-^{1}H HSQC and HMBC experiments, were acquired (see figures below). Complete signal assignment for ^{1}H and ^{13}C nuclei (Supplementary Table S3) was achieved through analysis of these spectra.
A key observation for identifying the rearranged thiazolidinyl fragment was the HMBC correlation between the carbon at position 5 and the protons of the methylene group at position 13. Furthermore, the protons of the second methylene group (C11) from the former epichlorohydrin moiety showed correlations to both the C4 and C2 carbonyl carbons (see figure below). The ^{1}H and ^{13}C chemical shifts of these methylene groups are consistent with the proposed structure. For instance, the signal for C13 appears at 27–28 ppm, which is characteristic of a methylene group bound to a sulfur atom.
To unambiguously confirm the thiazepane structure, model compound **36** was synthesized and its crystal structure was determined. The NMR chemical shifts of **36** (after accounting for substituent effects) correspond closely to those of compounds **34** and **35** (Table S1).

Supplementary Figure S12. Atom numbering and correlations between the ^{1}H and ^{13}C nuclei, as observed in the DQF-COSY (green arrows), ROESY (blue arrows) and ^{13}C-^{1}H HMBC (red arrows) 2D NMR spectra of the compounds **34** and **35**.

DQF-COSY
ROESY
^{13}C-^{1}H HMBC

34 **35**

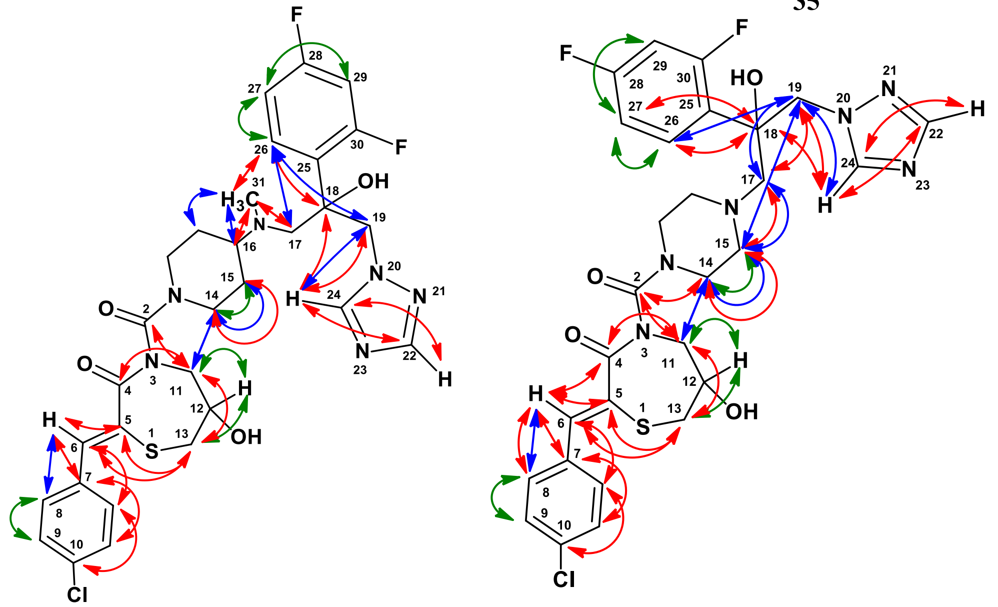

Table S3. Chemical shifts of the ^{1}H and ^{13}C signals of the thiazepane compounds **34**, **35** and **36**.

Position	35				34		36	
	DMSO-d$_6$		CDCl$_3$		DMSO-d$_6$		DMSO-d$_6$	
	^{13}C, ppm	^{1}H, ppm	^{13}C, ppm	^{1}H, ppm	^{13}C, ppm	^{1}H, ppm	^{13}C, ppm	^{1}H, ppm
2	157.12	-	156.80	-	156.88	-	-	8.02 (HN^3)
4	162.56	-	163.30	-	162.75	-	166.86	-
5	122.10	-	120.02	-	122.37	-	125.84	-
6	133.14	7.60	135.23	7.63	133.45	7.61	134.39	6.86
7	128.32	-	128.96	-	128.72	-	131.94	-
8	131.50	7.66	131.13	7.47	131.79	7.67	130.85	7.62
9	128.54	7.53	128.42	7.33	128.80	7.52	128.48	7.46
10	133.40	-	134.95	-	134.04	-	134.07	
11	43.30	3.36; 3.26	43.81	3.56; 3.40	43.71	3.40; 3.30	45.29	3.35
12	78.30	4.57; 6.74(OH)	78.94	4.61; 5.25(OH)	78.63	4.63	67.25	3.95; 5.30 (OH)
13	27.65	3.18; 3.10	27.65	3.00; 2.92	28.05	3.23; 3.13	34.78	3.09; 2.83
14	43.23	3.15	43.34	3.22	42.29; 42.16	2.67; 2.66	-	-
15	53.74	2.33	53.55	2.31	25.15; 26.16	2.03; 1.44 1.95; 1.55	-	-
16	-	-	-	-	65.75	3.43	-	-
17	63.37	2.83; 2.67	62.06	3.03; 2.66	57.42	3.73; 3.56	-	-
18	74.48	5.67(OH)	72.19	-	71.86	-	-	-
19	55.43	4.56	55.60	4.50	55.23	4.88; 4.70	-	-
22	150.28	7.73	150.78	7.75	149.62	7.95	-	-
24	144.76	8.28	144.31	8.08	145.22	8.77	-	-
25	125.99	-	125.26	-	123.05	-	-	-
26	129.65	7.39	128.98	7.48	130.65	7.46	-	-
27	110.56	6.95	111.36	6.77	111.77	7.06	-	-
28	158.77	-	158.36	-	158.95	-	-	-
29	103.57	7.14	104.07	6.77	104.67	7.30	-	-
30	161.49	-	162.37	-	162.63	-	-	-
31	-	-	-	-	40.07	2.38	-	-

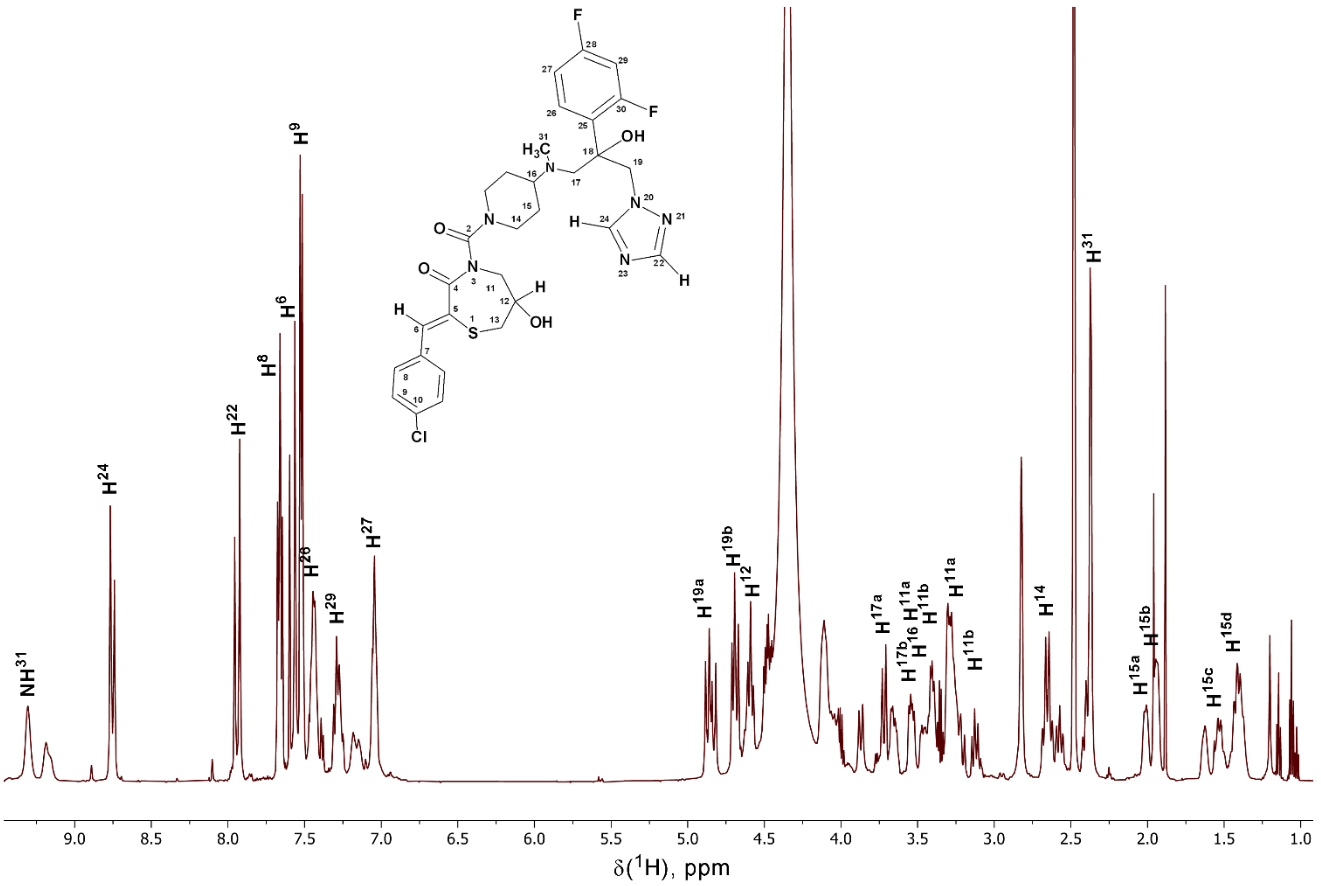

Supplementary Figure S15. 1D NMR ^{1}H spectrum of compound **34** as a mixture with other reaction products in DMSO-d_6 solution, measured at 600 MHz frequency and 25°C. Signal assignments were obtained based on the analysis of 2D NMR spectra.

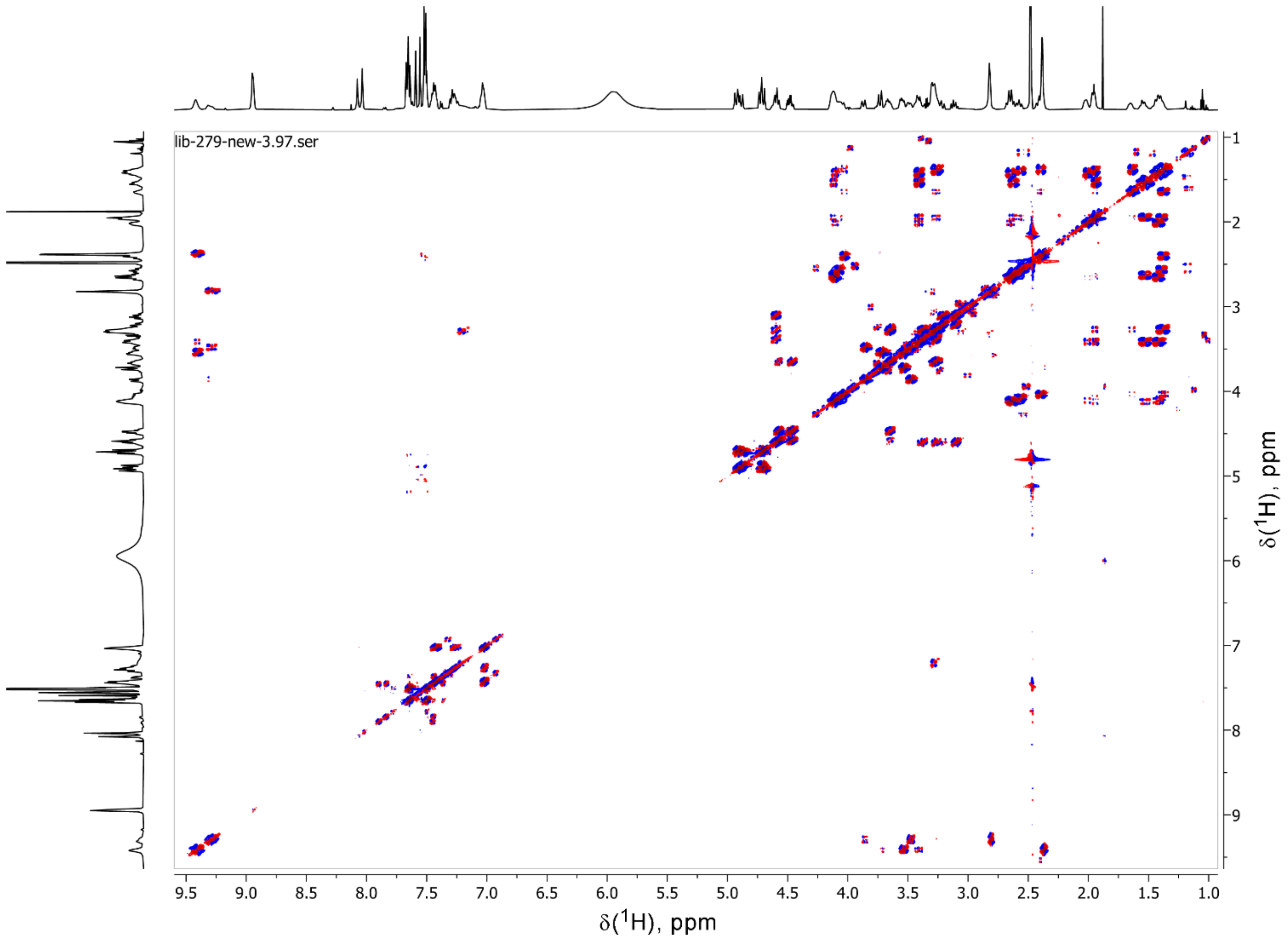

Supplementary Figure S16. 2D DQF-COSY spectrum of compound **34** as a mixture with other reaction products in DMSO-d$_6$ solution, measured at 600 MHz frequency and 25°C.

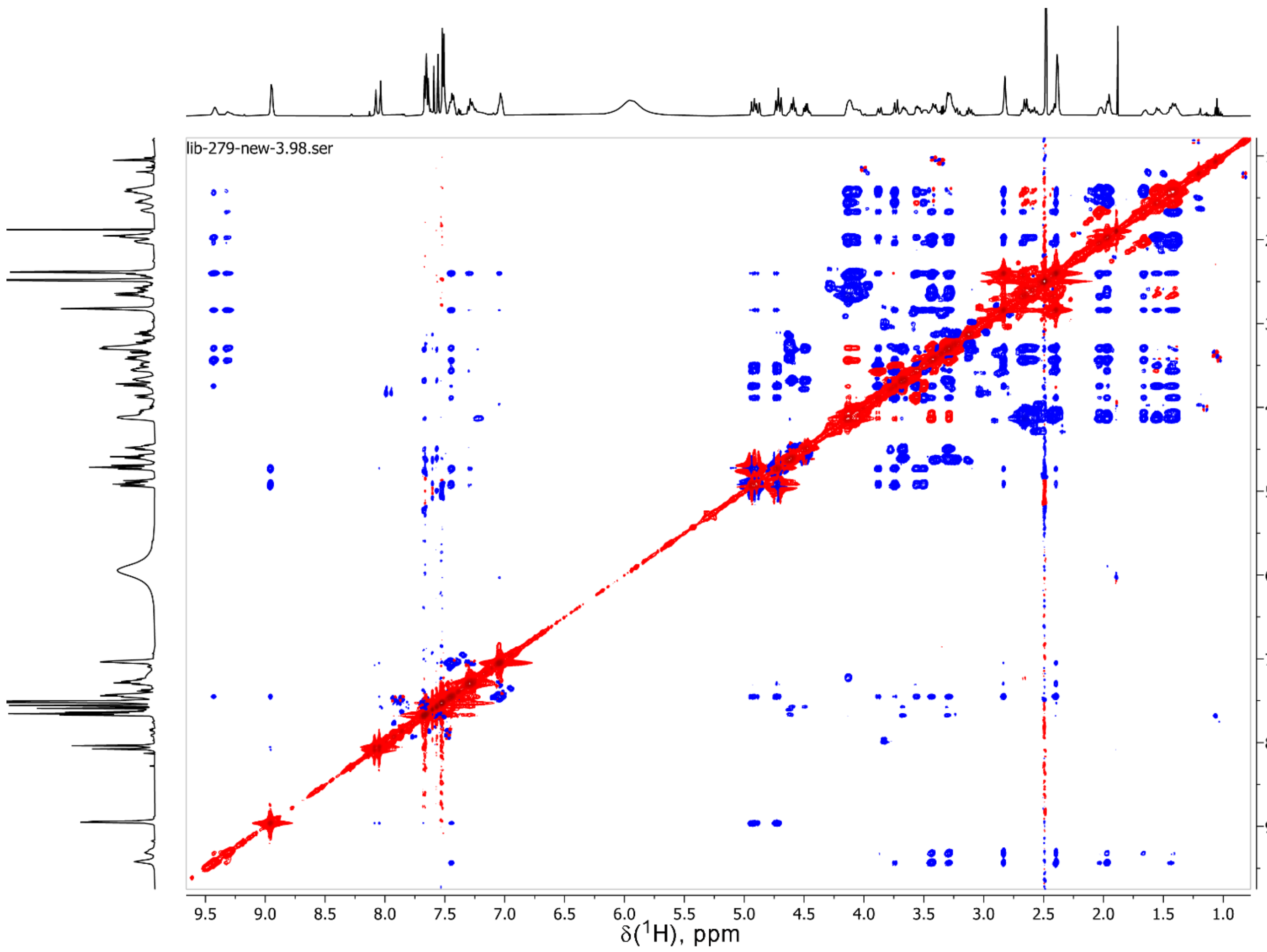

Supplementary Figure S17. 2D ROESY spectrum (mixing time 320 ns) of compound **34** as a mixture with other reaction products in DMSO-d$_6$ solution, measured at 600 MHz frequency and 25°C.

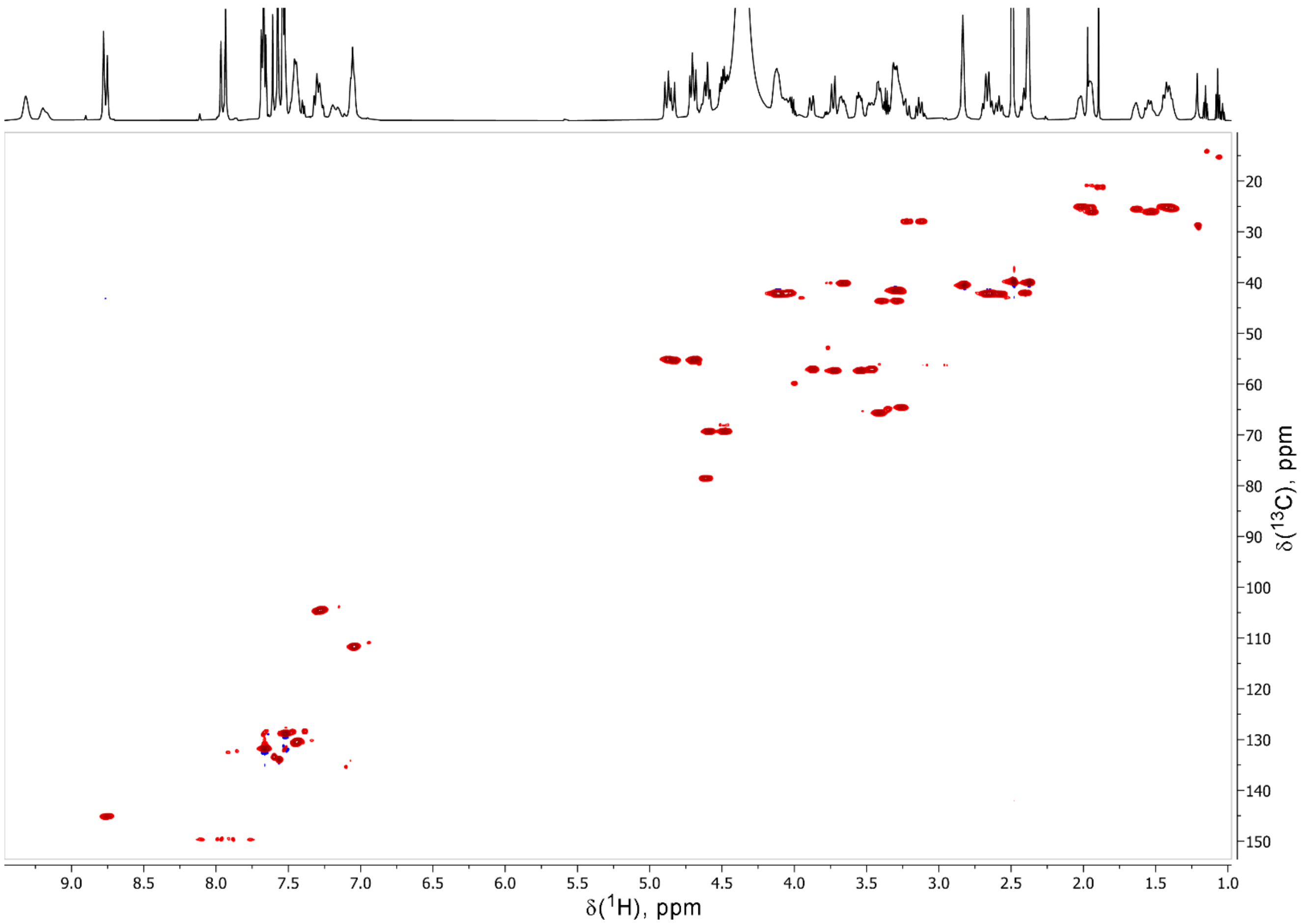

Supplementary Figure S18. 2D ^{13}C-^{1}H HSQC spectrum of compound **34** as a mixture with other reaction products in DMSO-d_6 solution, measured at 600 MHz frequency and 25°C.

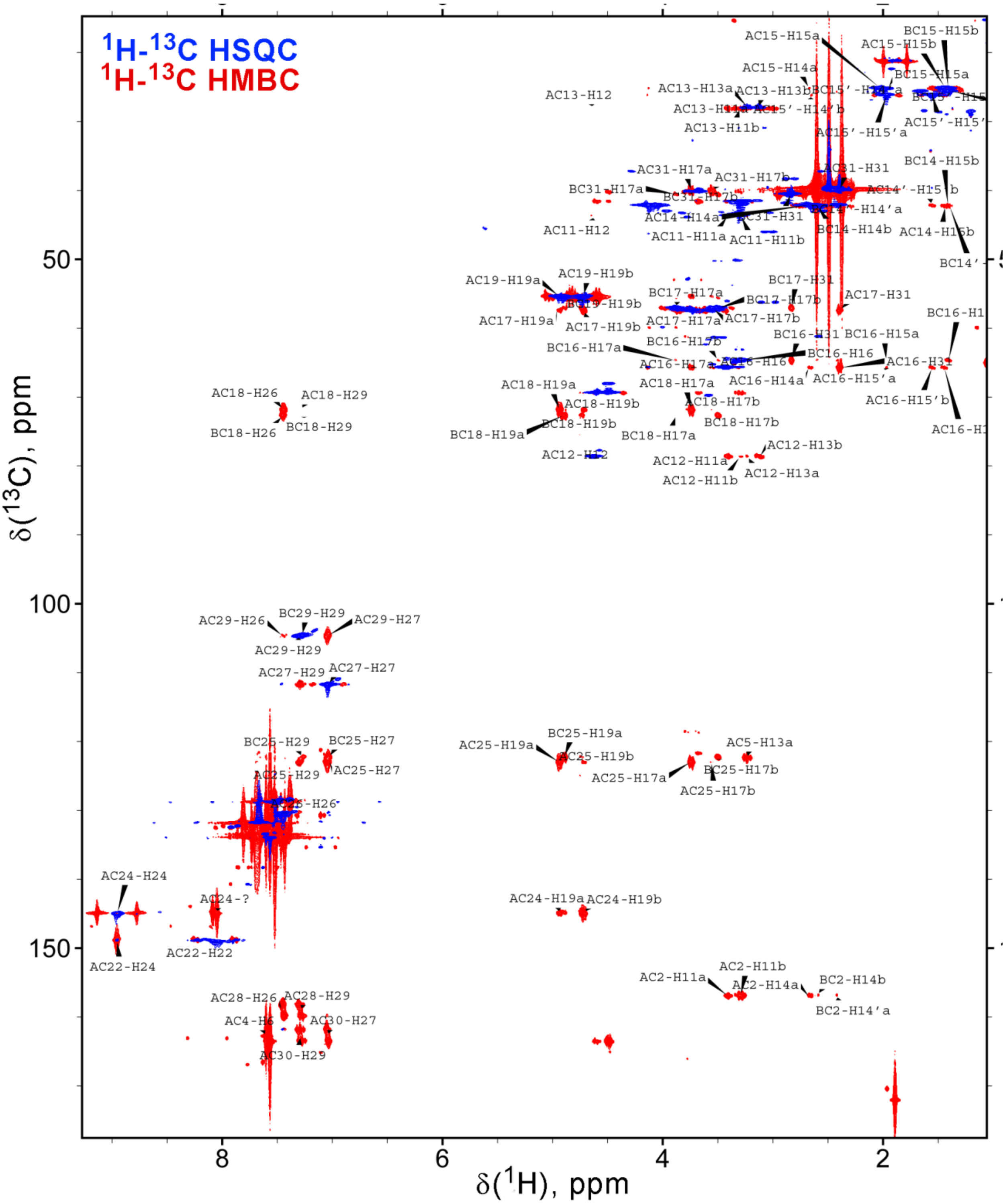

Supplementary Figure S19. Superposition of 2D ^{13}C-^{1}H HSQC (blue cross-peaks) and ^{13}C-^{1}H HMBC (red signals) spectra of compound **34** in DMSO-d_6 solution, measured at 600 MHz frequency and 25°C.

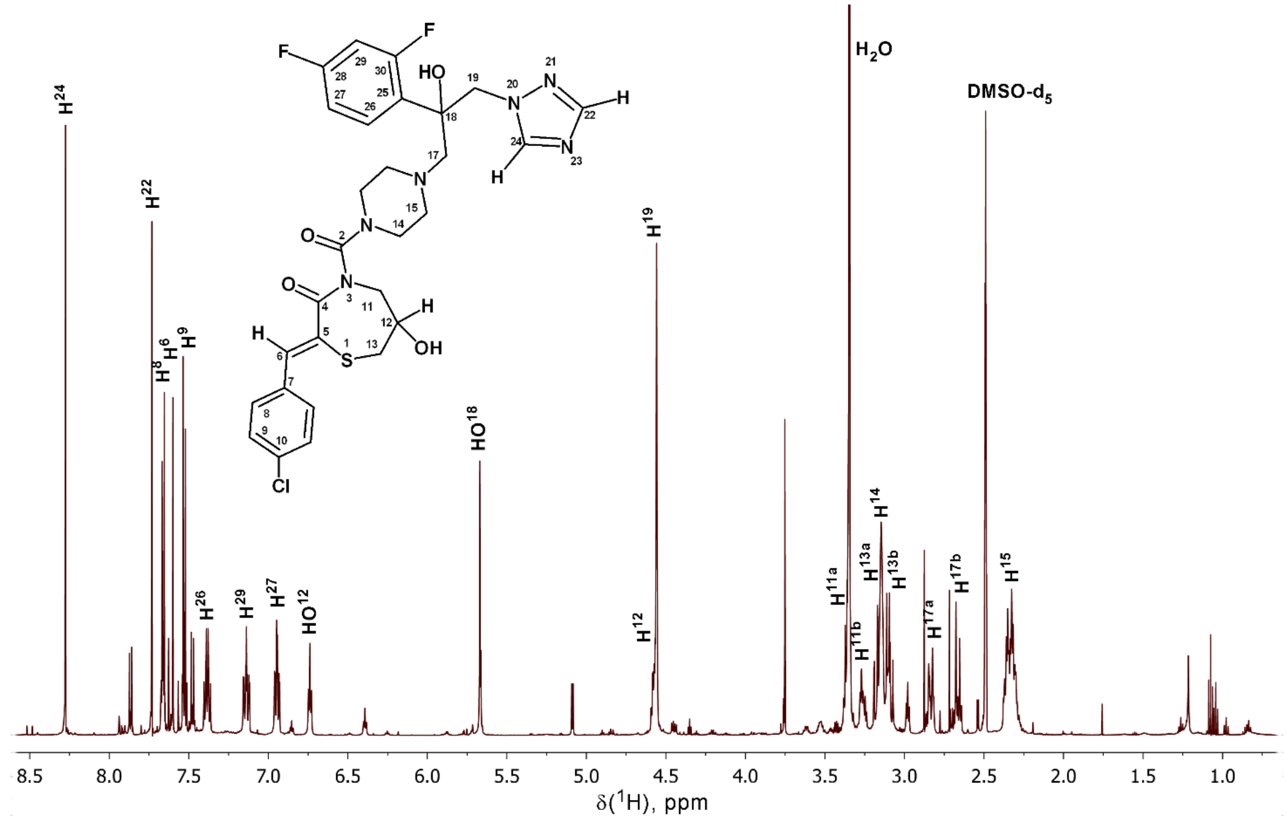

Supplementary Figure S20. 1D NMR ^{1}H spectrum of compound **35** as a mixture with other reaction products in DMSO-d_6 solution, measured at 600 MHz frequency and 25°C. Signal assignments were obtained based on the analysis of 2D NMR spectra.

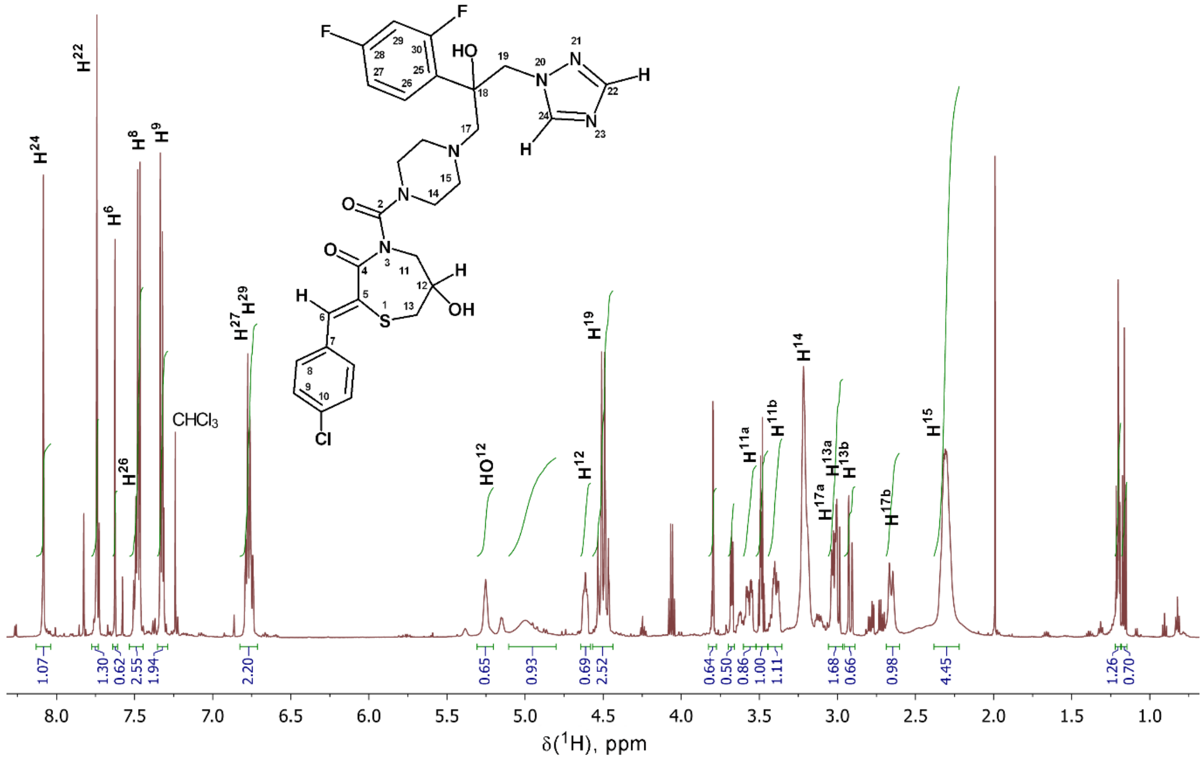

Supplementary Figure S21. 1D NMR ^{1}H spectrum of compound **35** as a mixture with other reaction products in $CDCl_3$ solution, measured at 600 MHz frequency and 25°C.

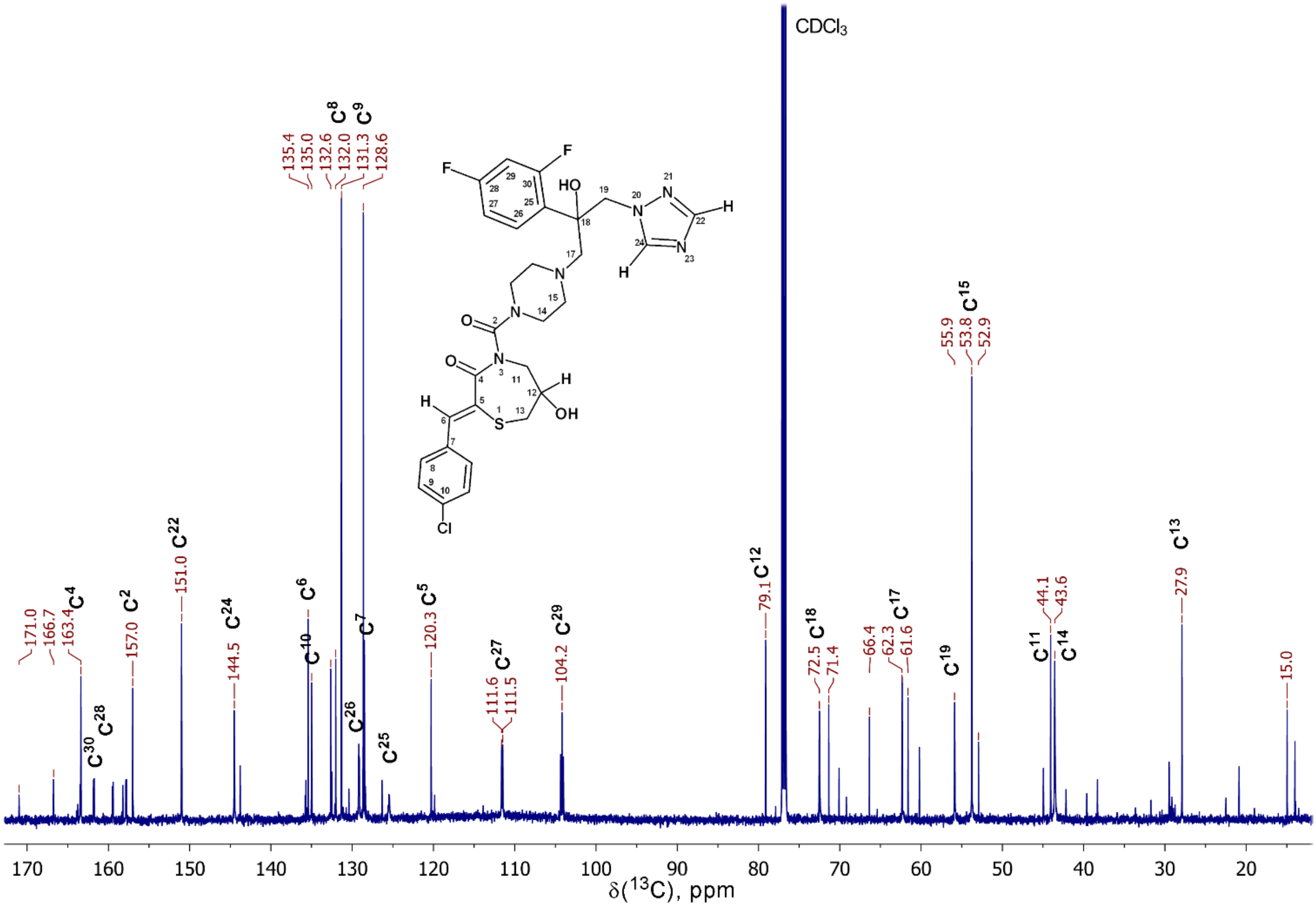

Supplementary Figure S22. 1D NMR ^{13}C spectrum of compound **35** as a mixture with other reaction products in $CDCl_3$ solution, measured at 125 MHz ^{13}C resonance frequency and 25°C. Signal assignments were obtained based on the analysis of 2D NMR spectra.

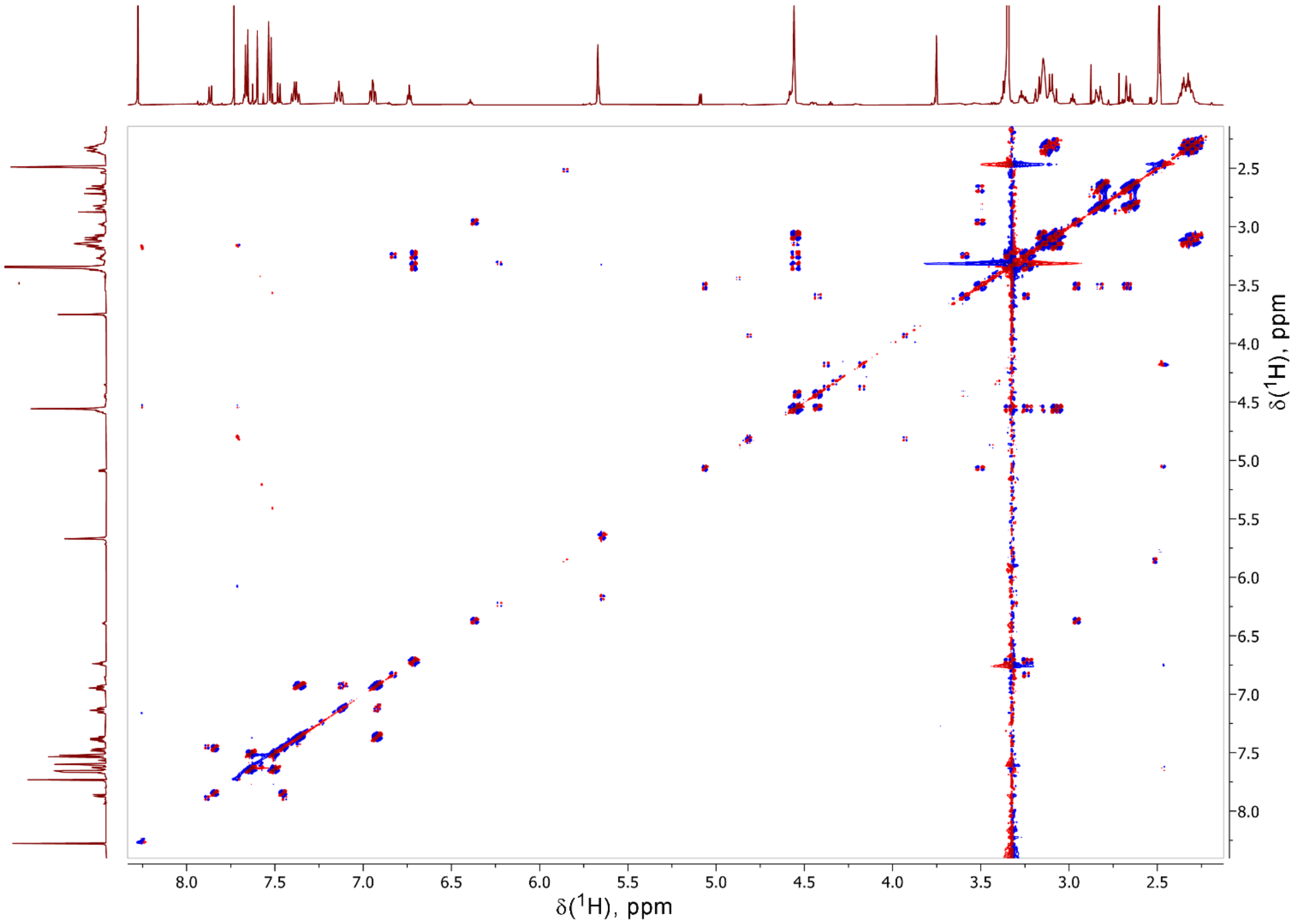

Supplementary Figure S23. 2D DQF-COSY spectrum of compound **35** in DMSO-d_6 solution, measured at 600 MHz frequency and 25°C.

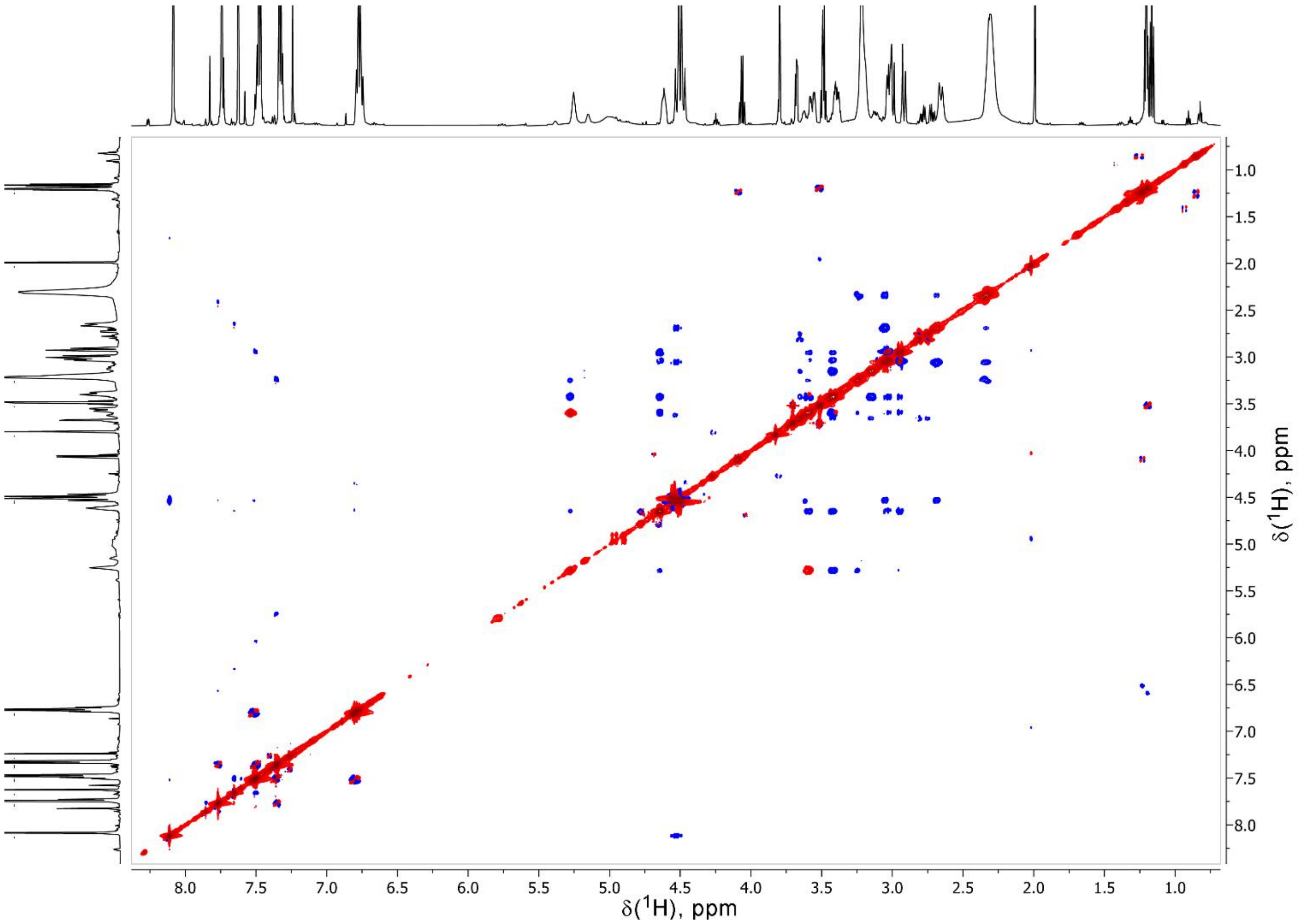

Supplementary Figure S24. 2D ROESY spectrum (mixing time 320 ms) of compound **35** in $CDCl_3$ solution, measured at 600 MHz frequency and 25°C.

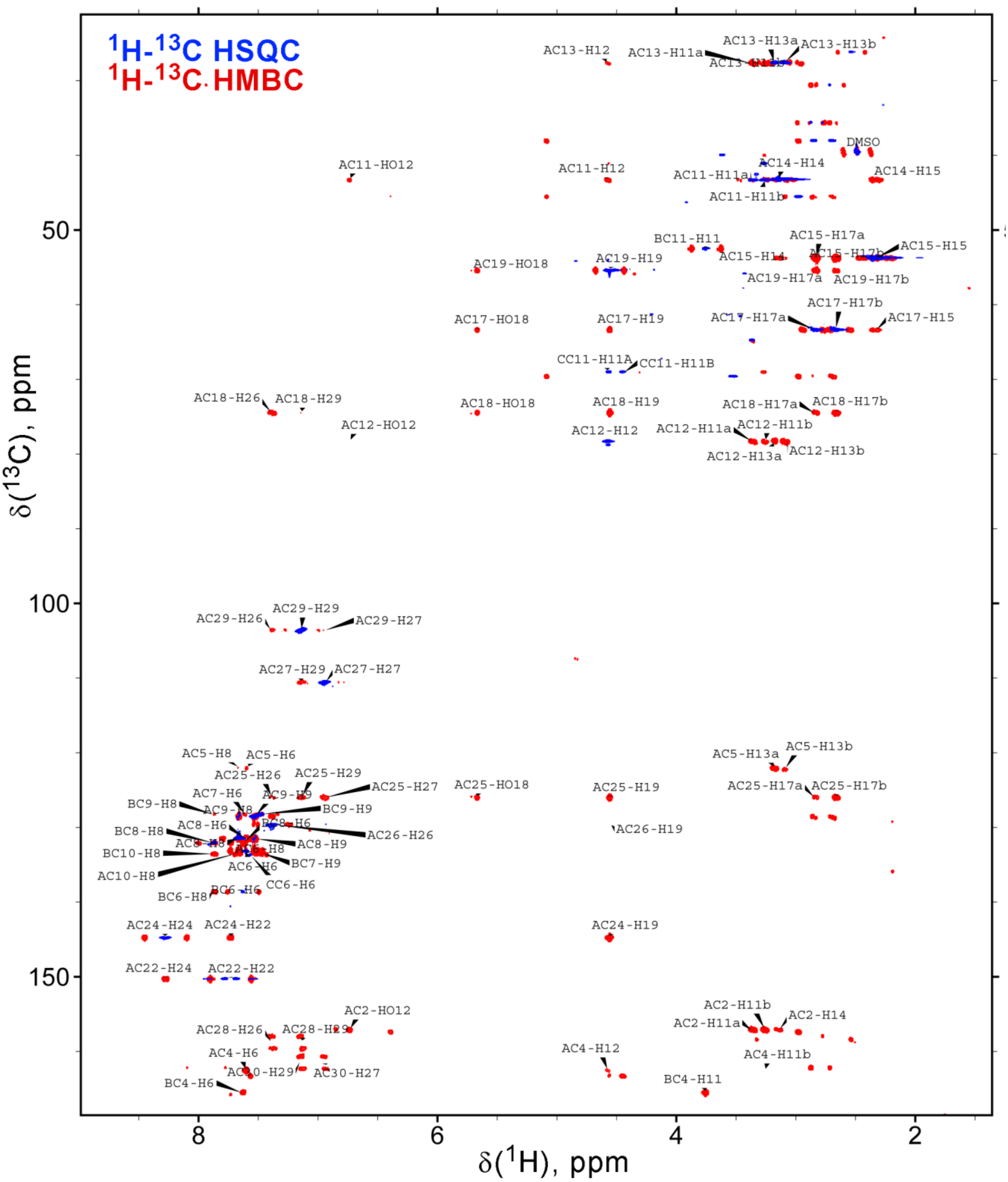

Supplementary Figure S25. Superposition of 2D $^{13}C-^{1}H$ HSQC (blue cross-peaks) and $^{13}C-^{1}H$ HMBC (red signals) spectra of compound **35** in DMSO-d_6 solution, measured at 600 MHz frequency and 25°C.

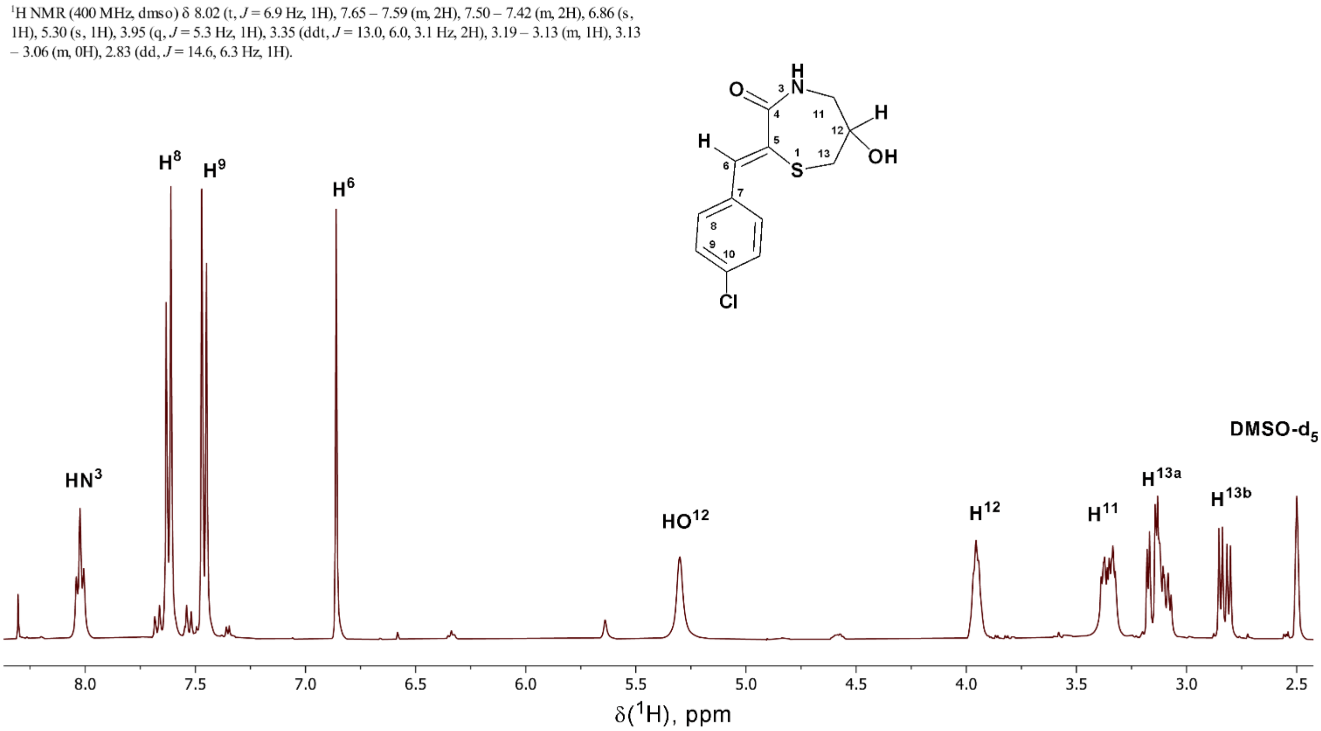

Supplementary Figure S26. 1D NMR ^{1}H spectrum of compound **36** in DMSO-d_6 solution, measured at 400 MHz frequency and 25°C.

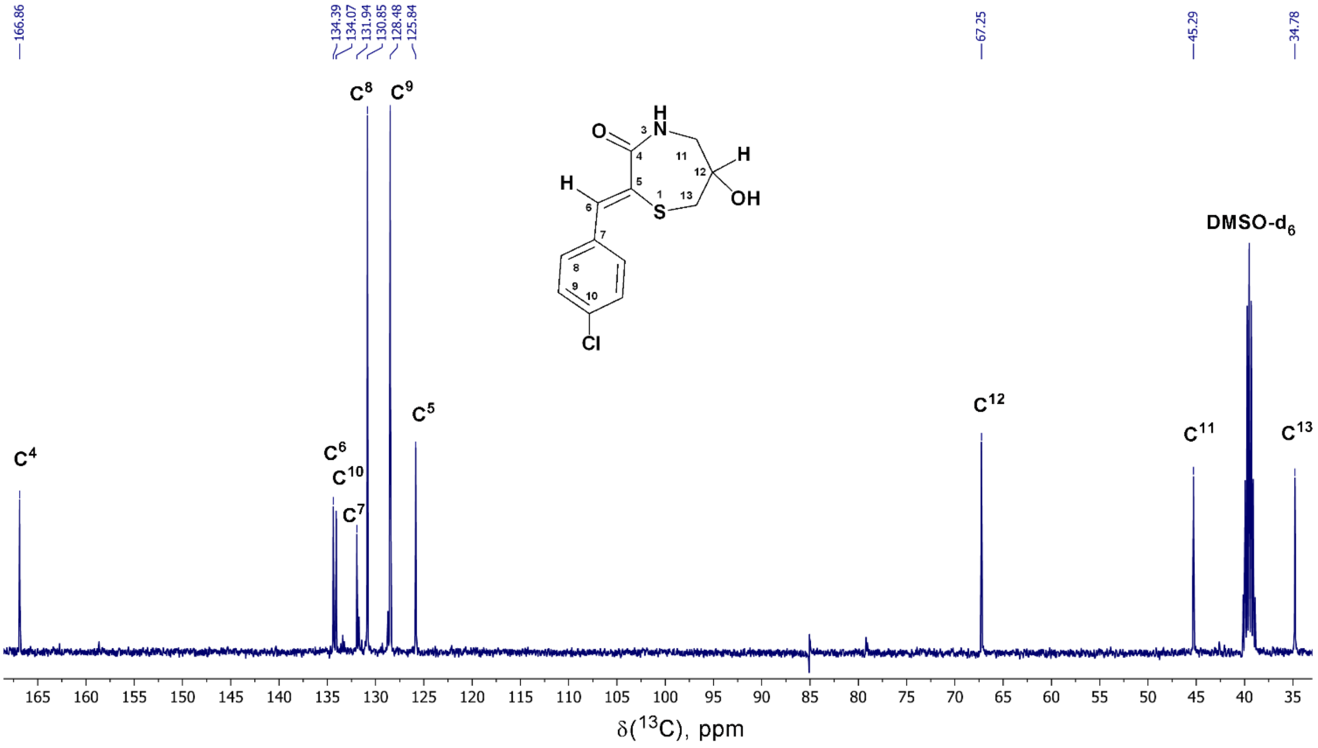

Supplementary Figure S27. 1D NMR ^{13}H spectrum of compound **36** in DMSO-d_6 solution, measured at 100 MHz ^{13}C frequency and 25°C.

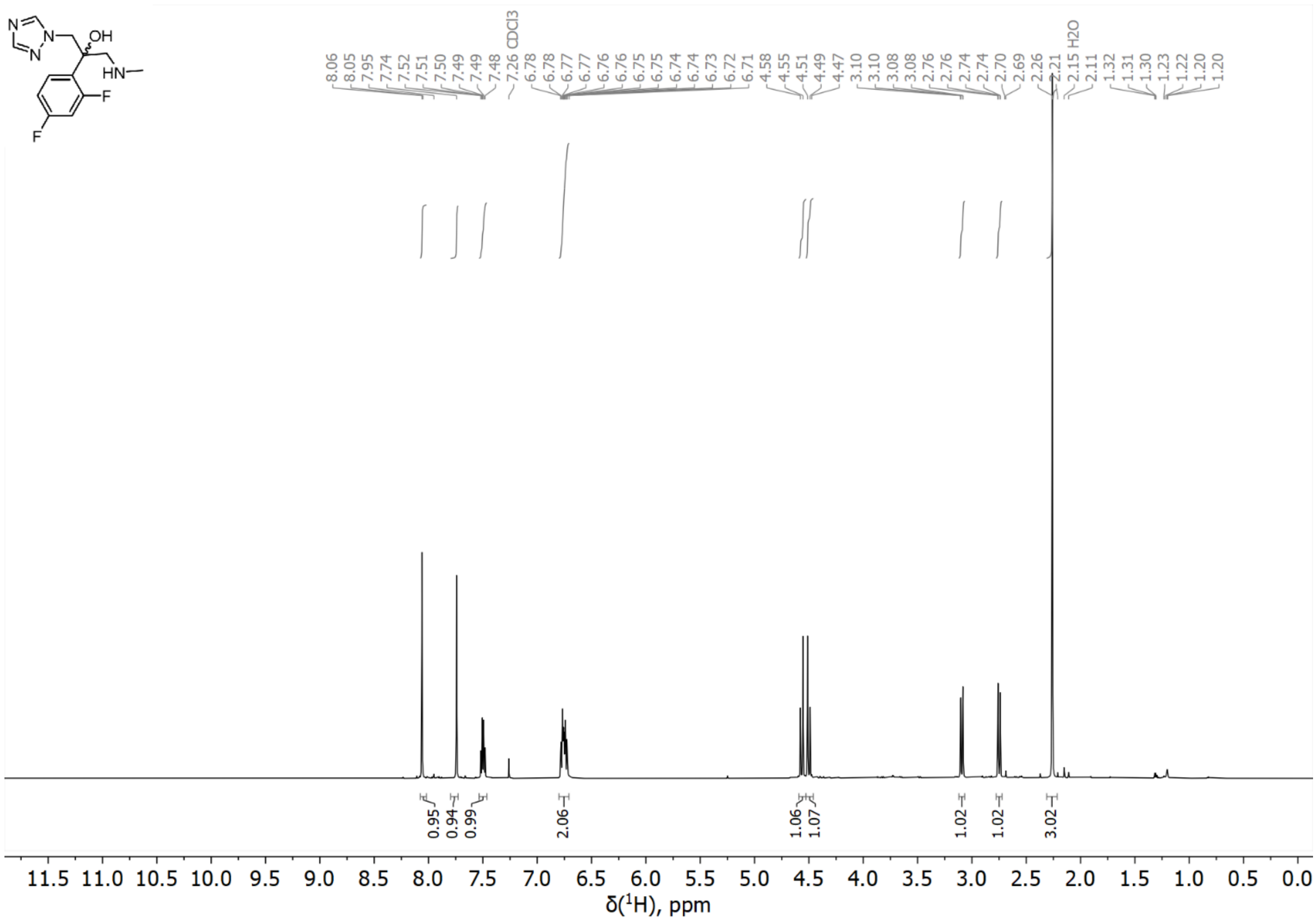

Supplementary Figure S28. ^{1}H NMR spectrum of **2b**

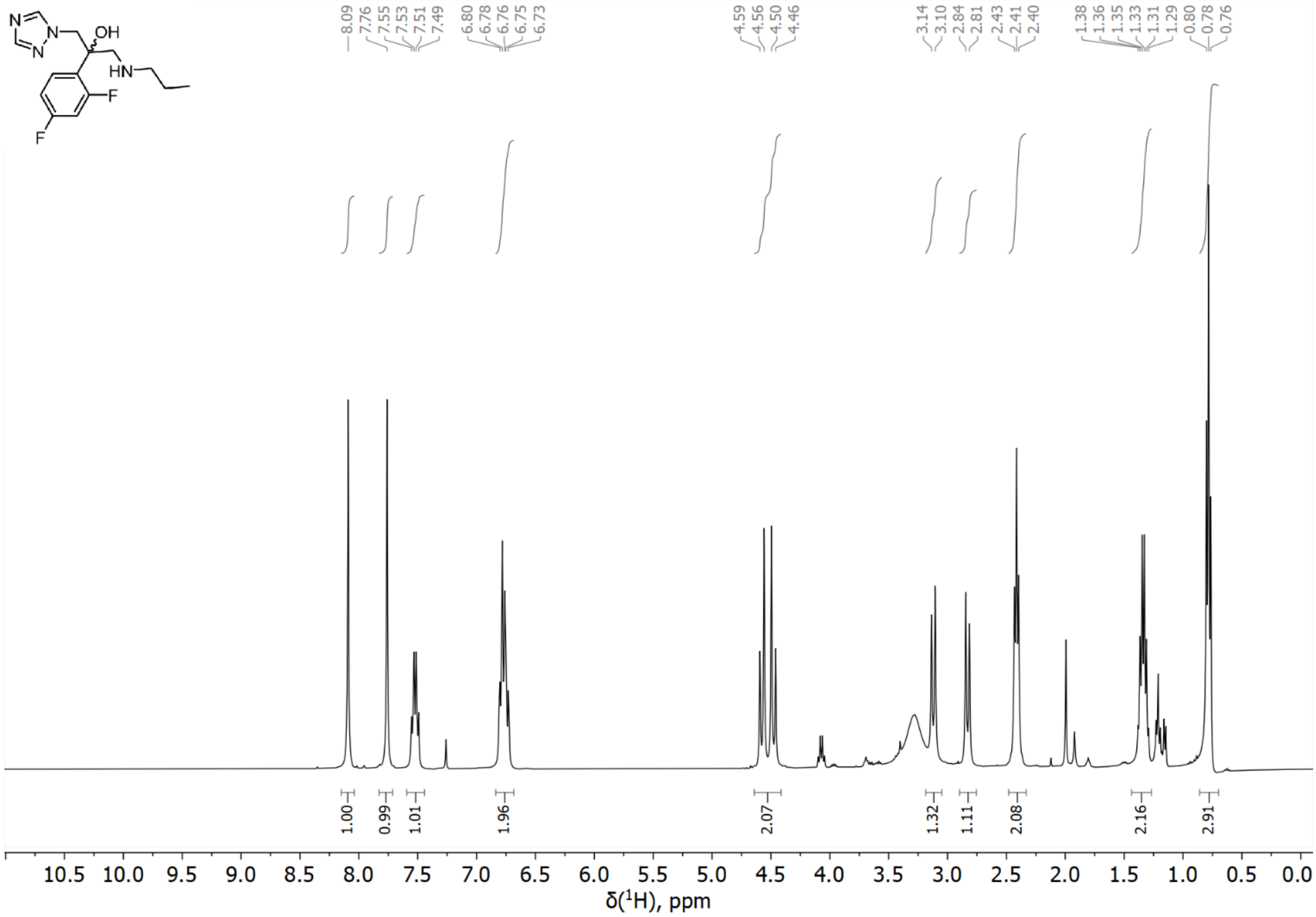

Supplementary Figure S29. ^{1}H NMR spectrum of **2c**

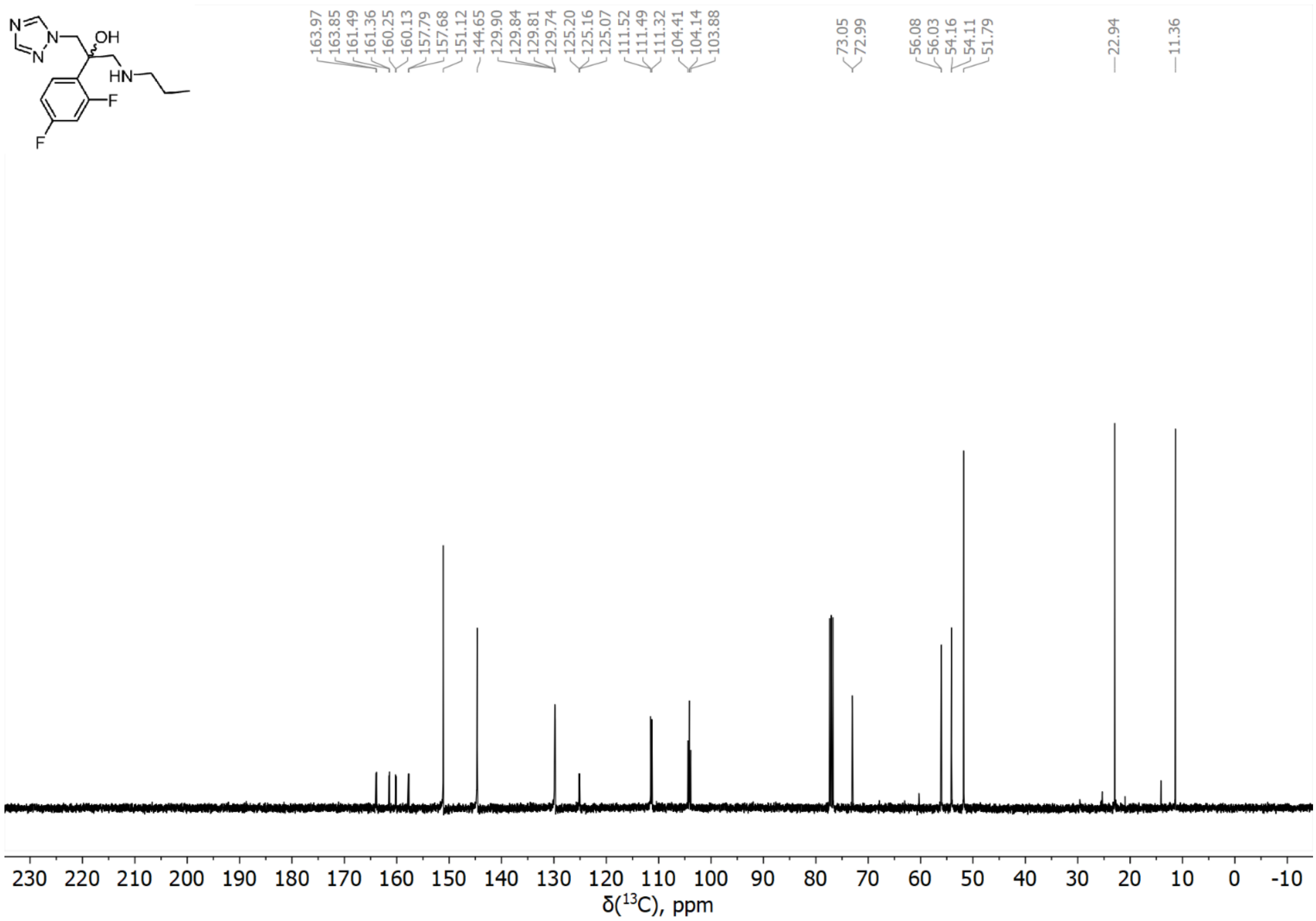

Supplementary Figure S30. ^{13}C NMR spectrum of **2c**

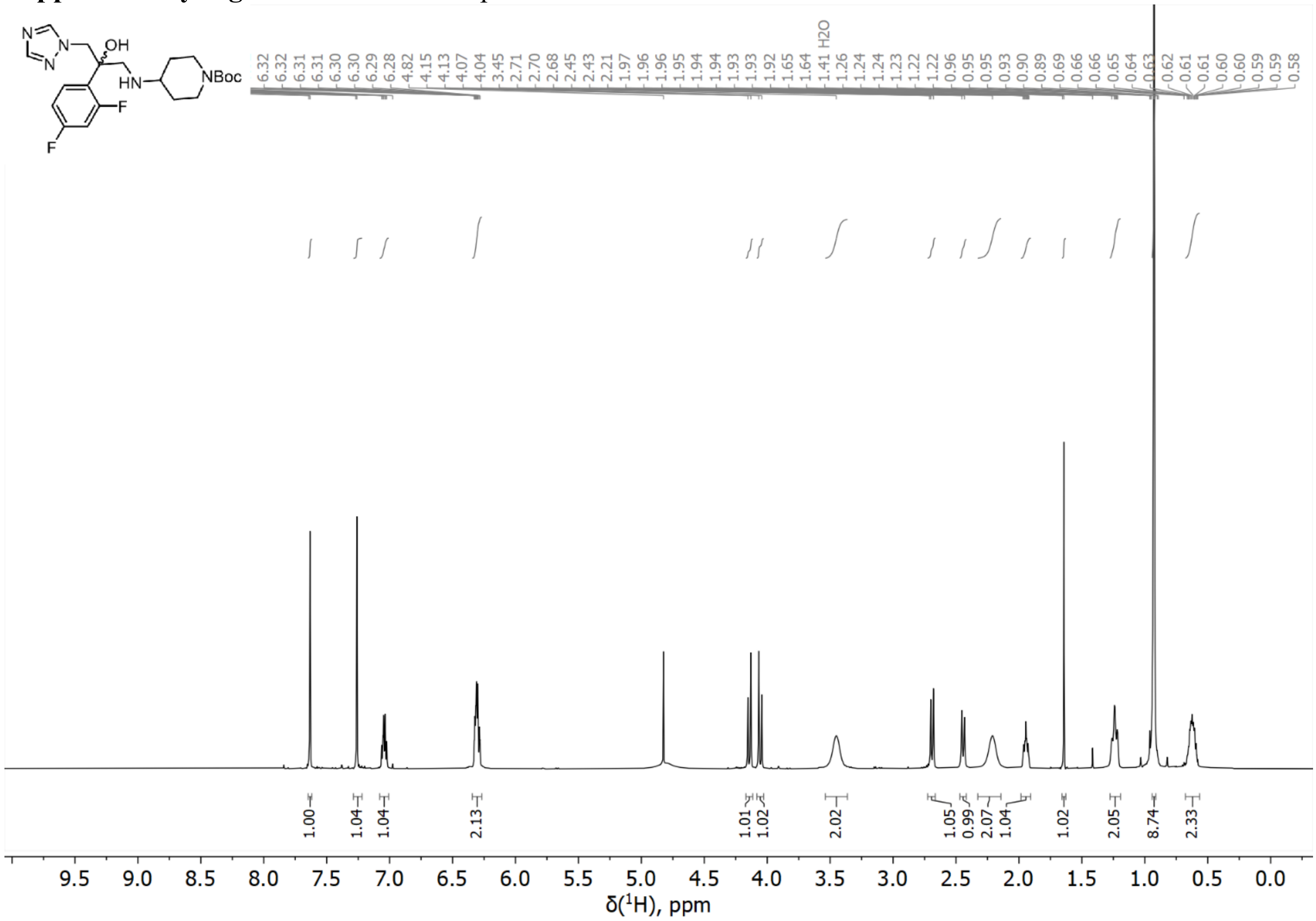

Supplementary Figure S31. ^{1}H NMR spectrum of **2e**

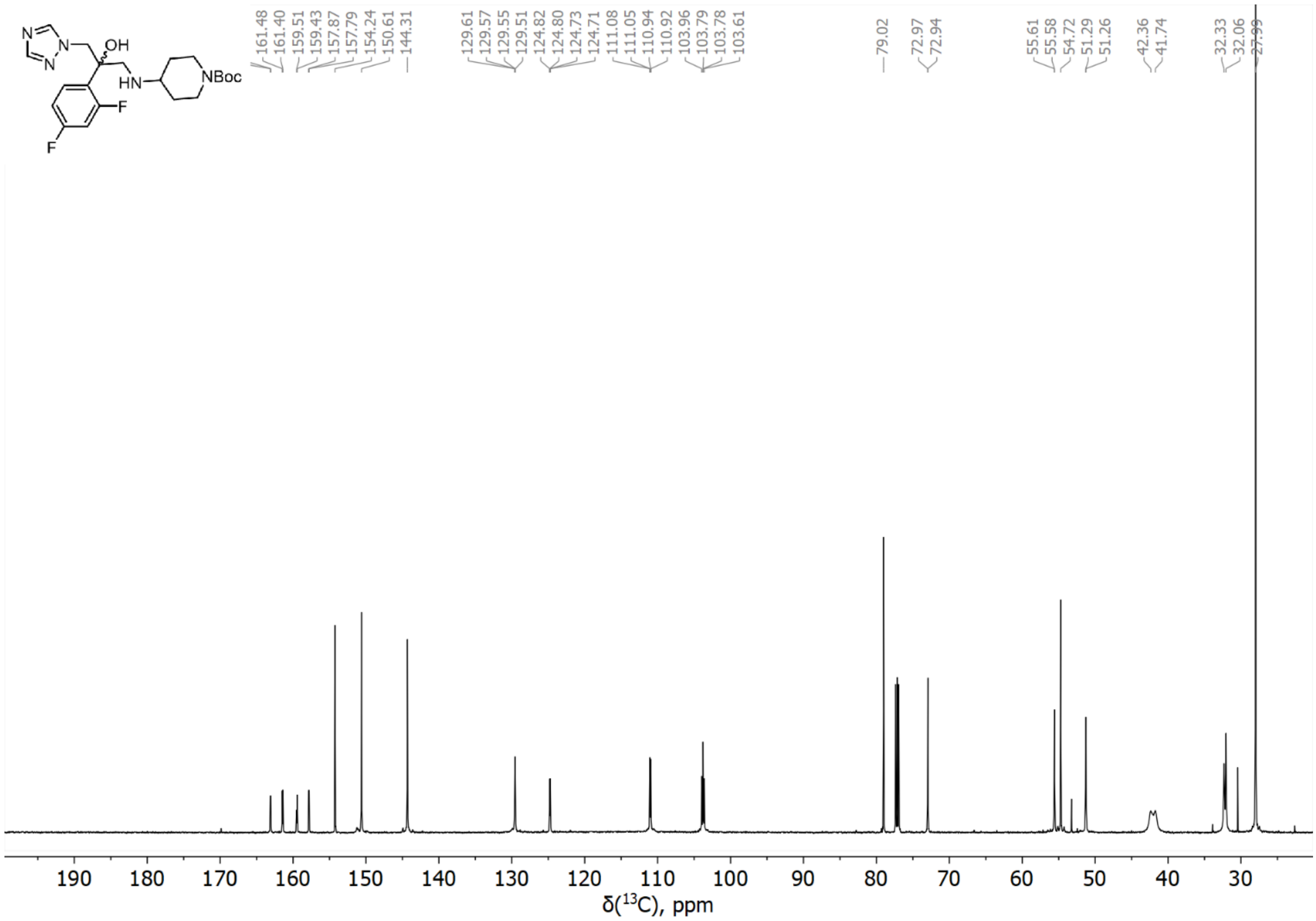

Supplementary Figure S32. ^{13}C NMR spectrum of **2e**

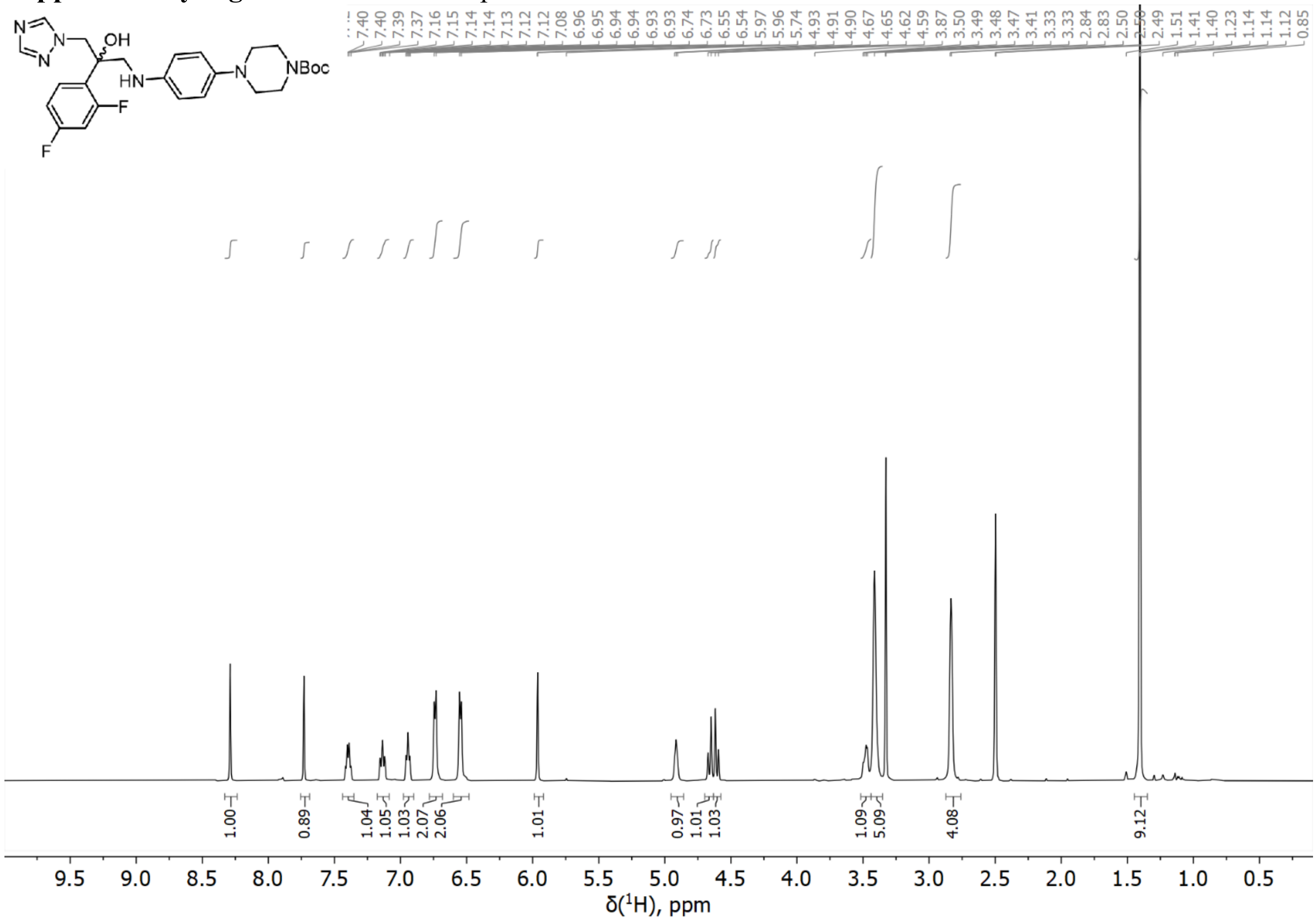

Supplementary Figure S33. ^{1}H NMR spectrum of **2f**

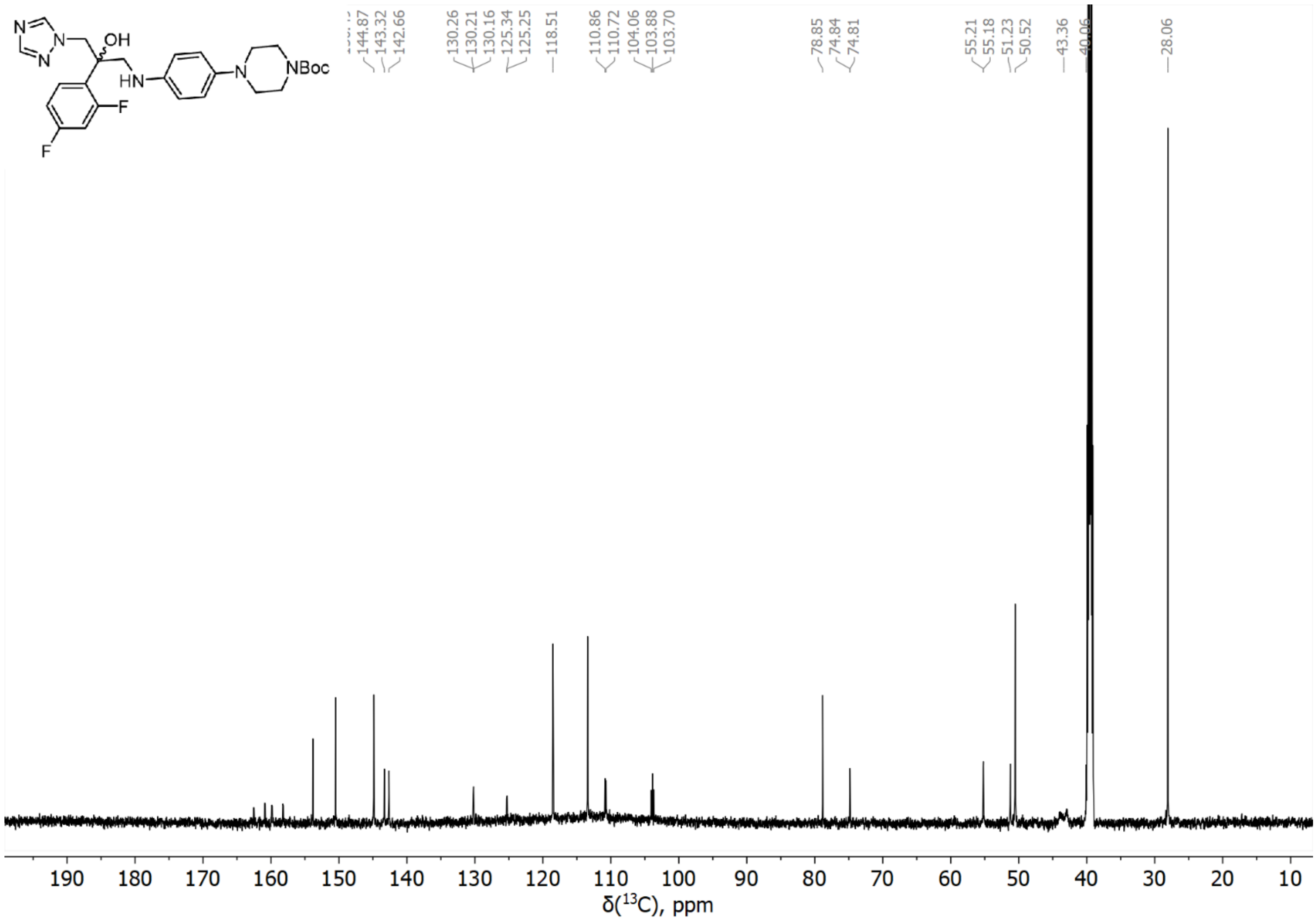

Supplementary Figure S34. ^{13}C NMR spectrum of **2f**

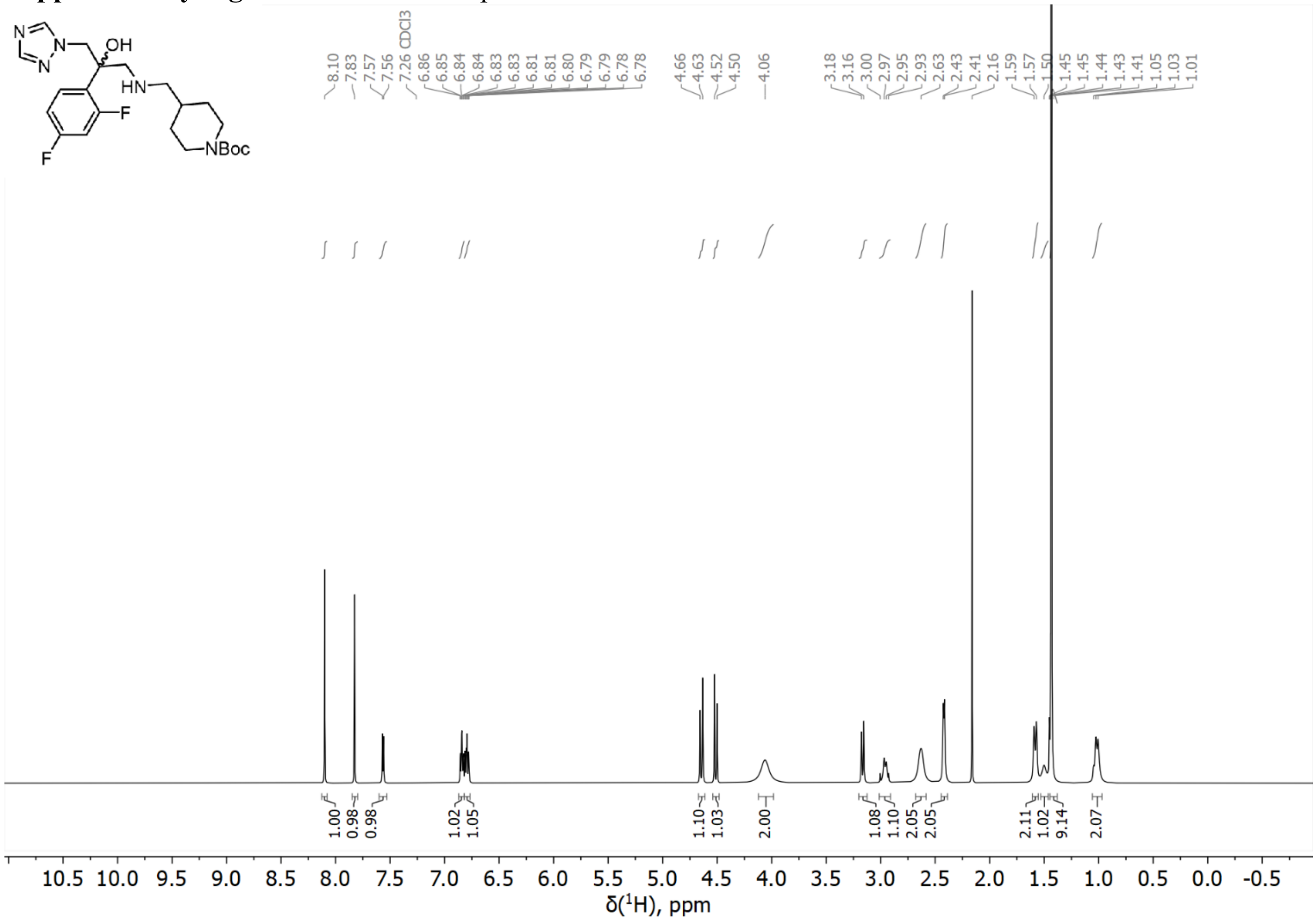

Supplementary Figure S35. ^{1}H NMR spectrum of **2g**

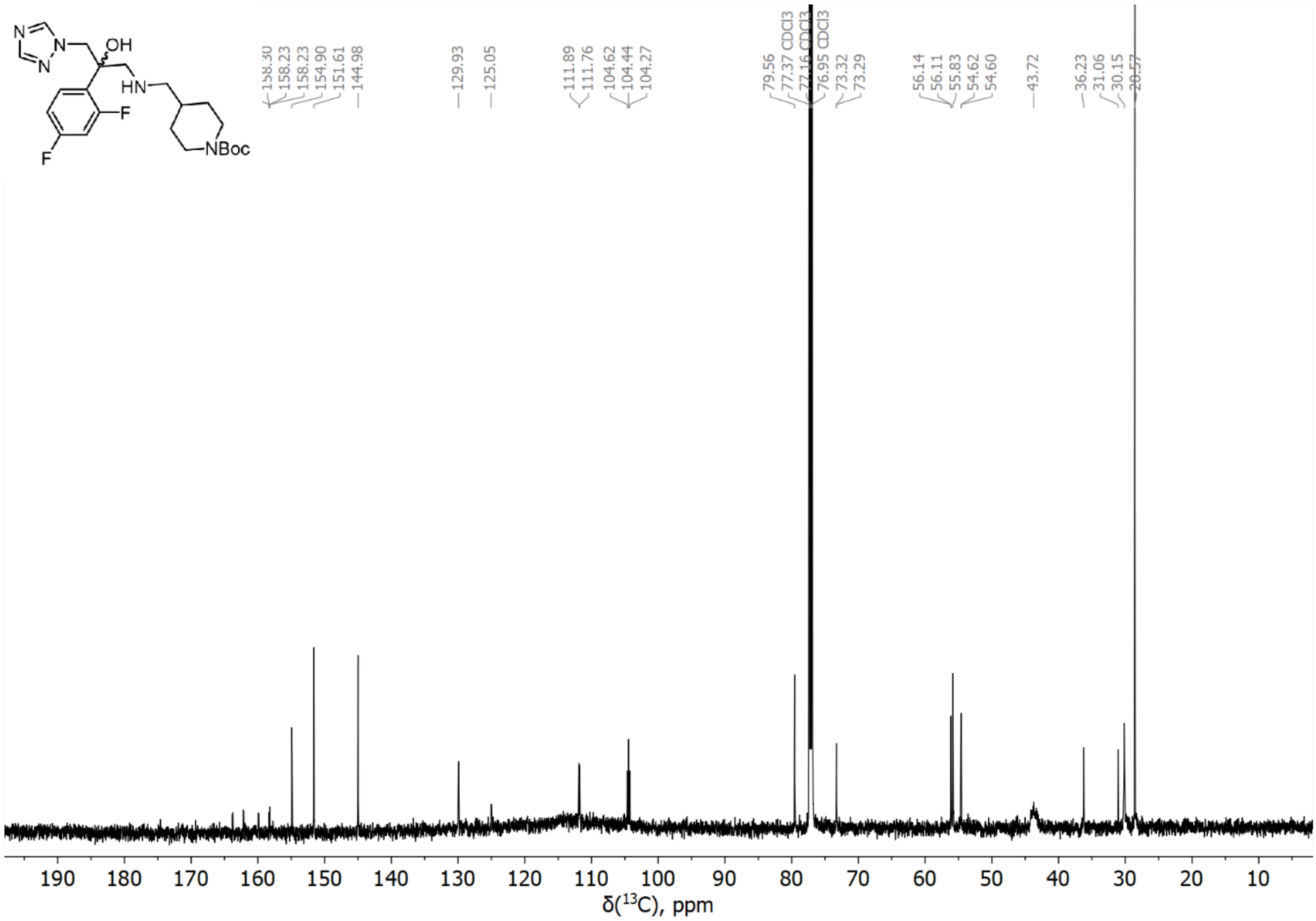

Supplementary Figure S36. ^{13}C NMR spectrum of **2g**

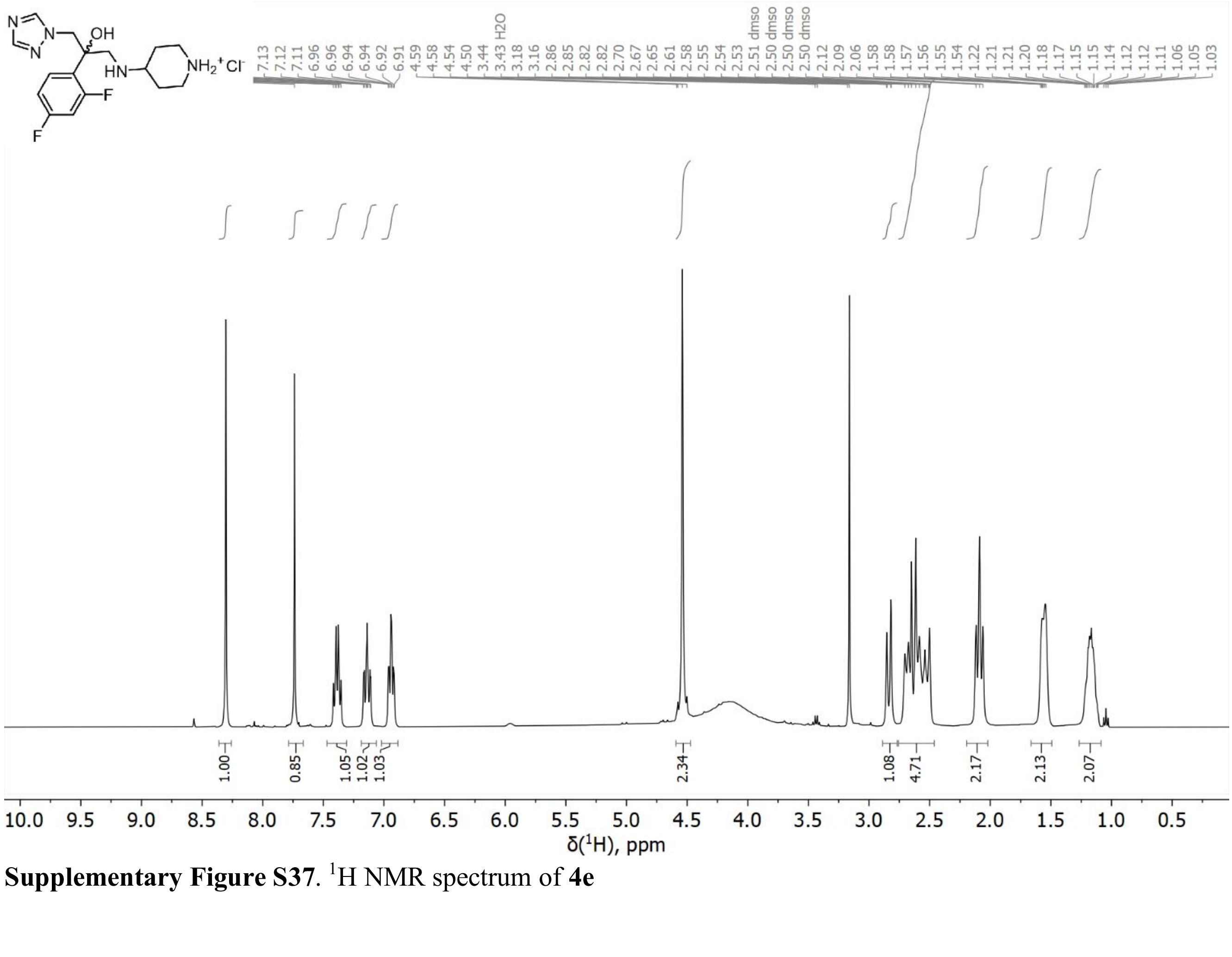

Supplementary Figure S37. ^{1}H NMR spectrum of **4e**

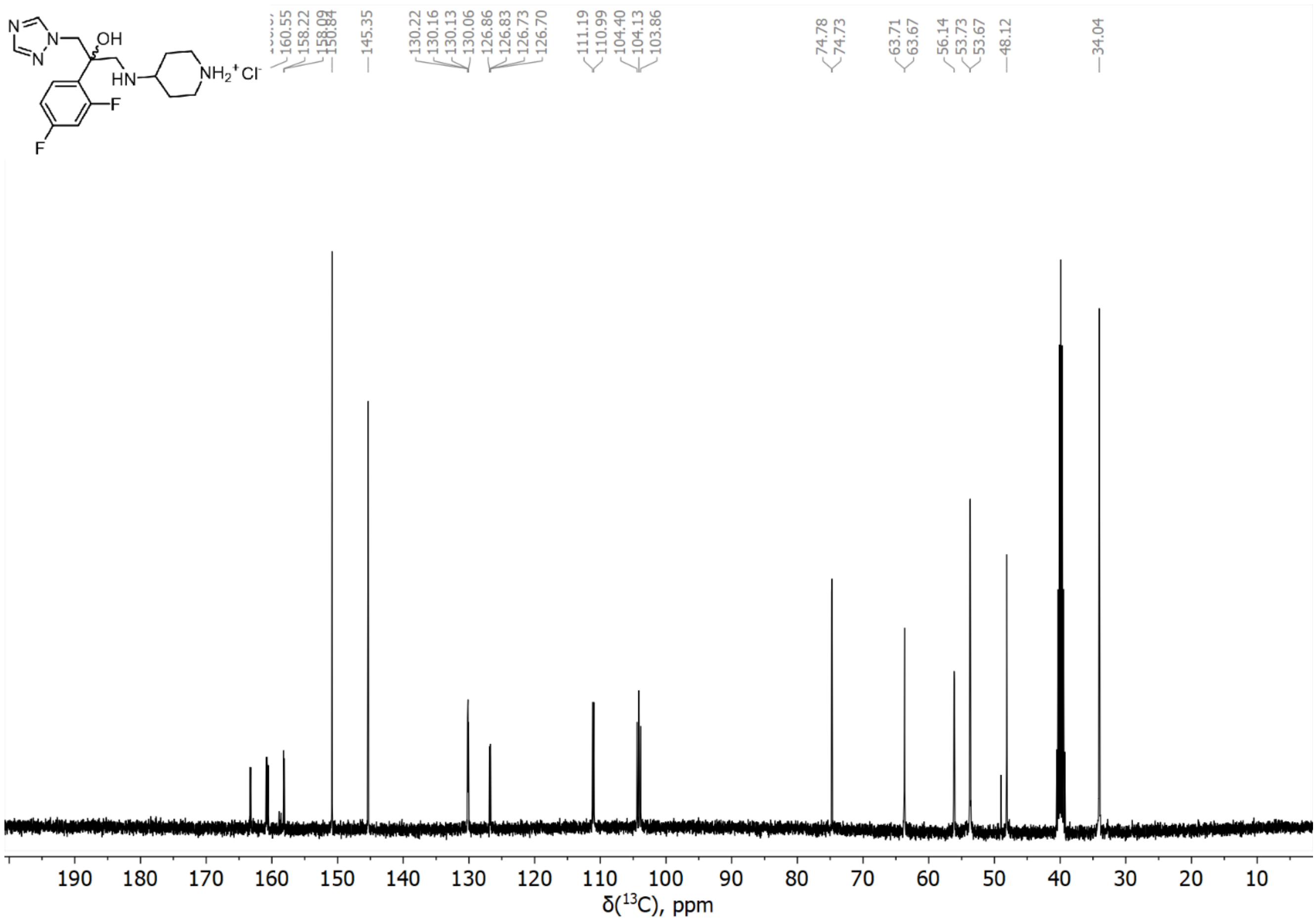

Supplementary Figure S38. ^{13}C NMR spectrum of **4e**

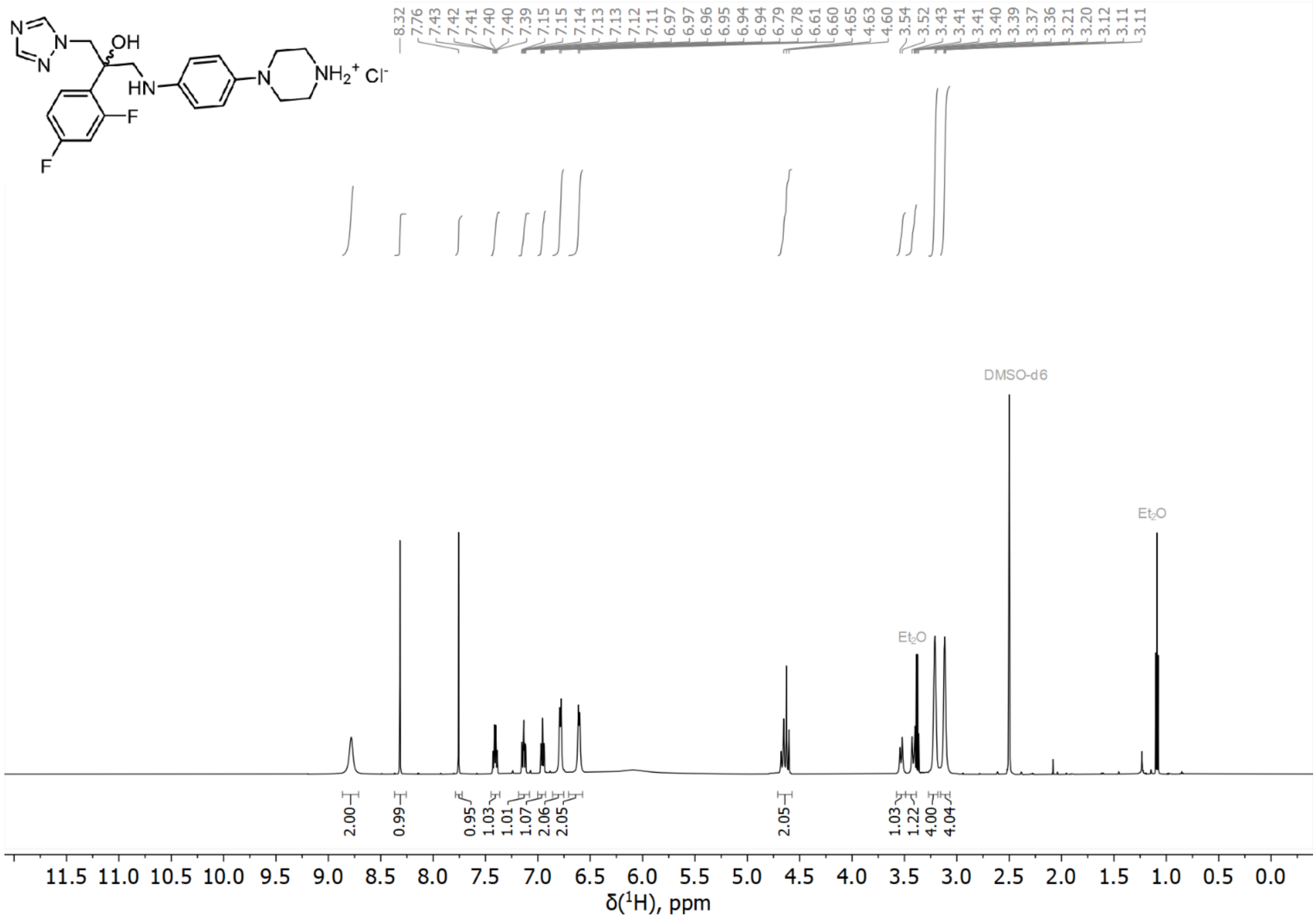

Supplementary Figure S39. ^{1}H NMR spectrum of **4f**

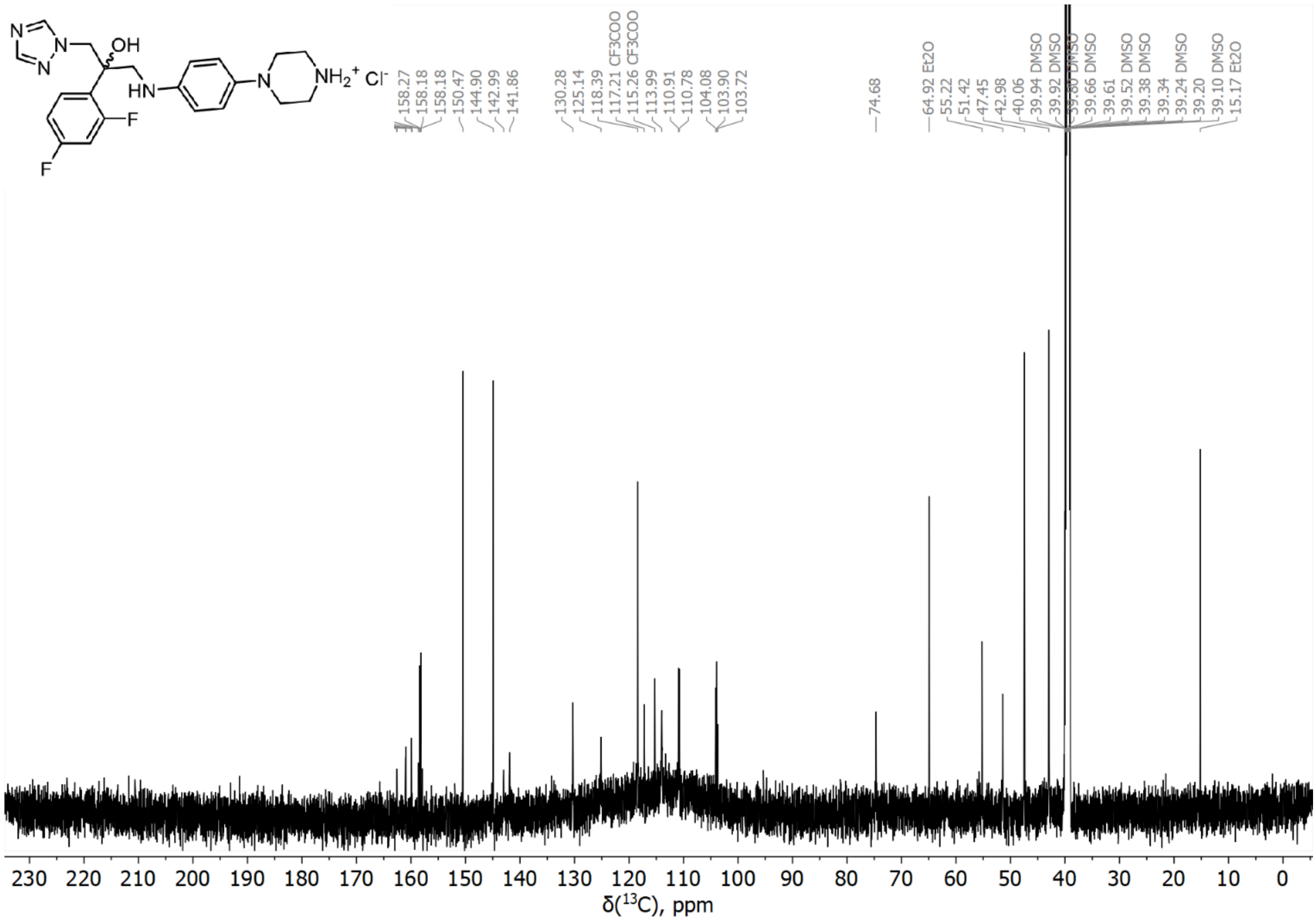

Supplementary Figure S40. ^{13}C NMR spectrum of **4f**

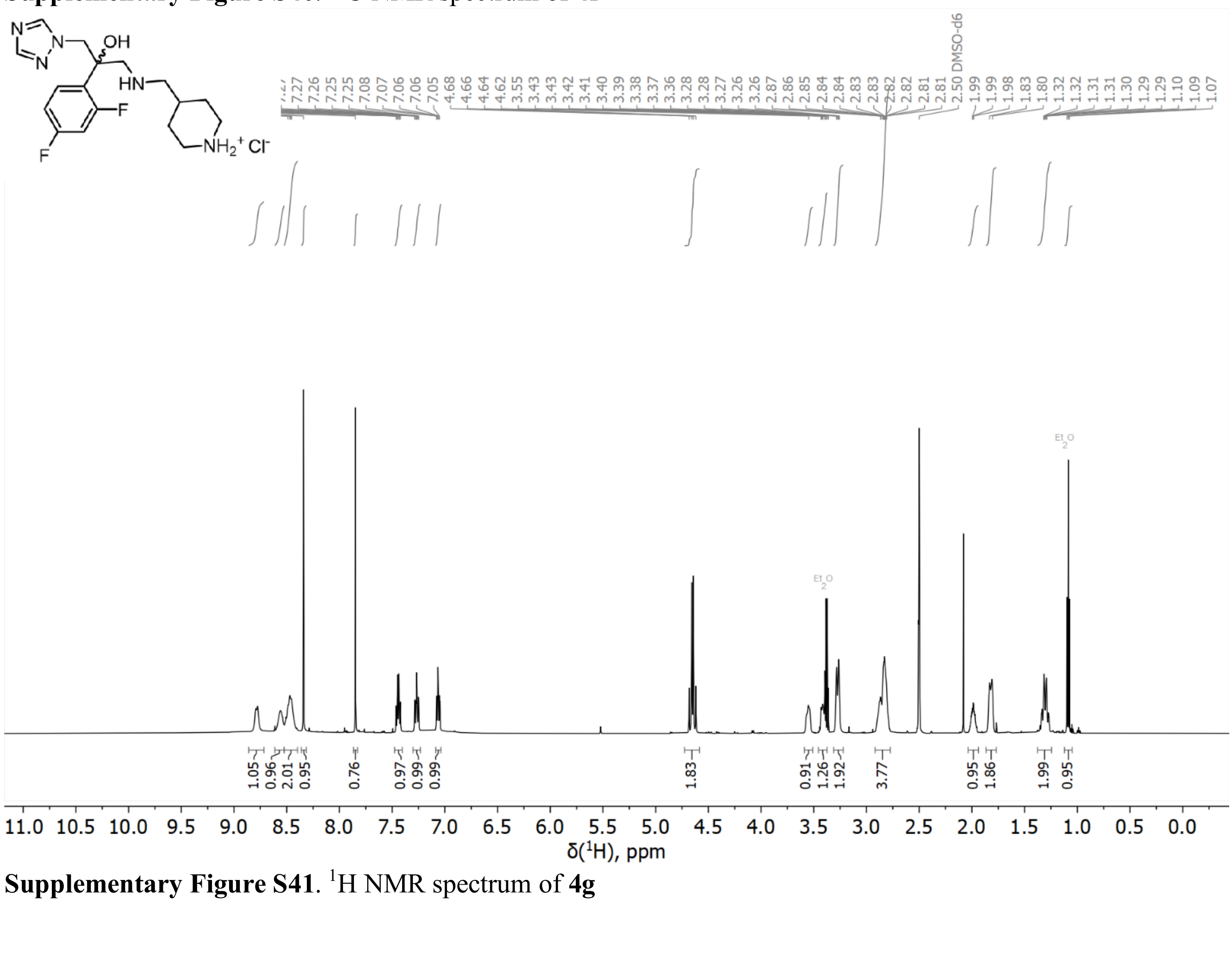

Supplementary Figure S41. ^{1}H NMR spectrum of **4g**

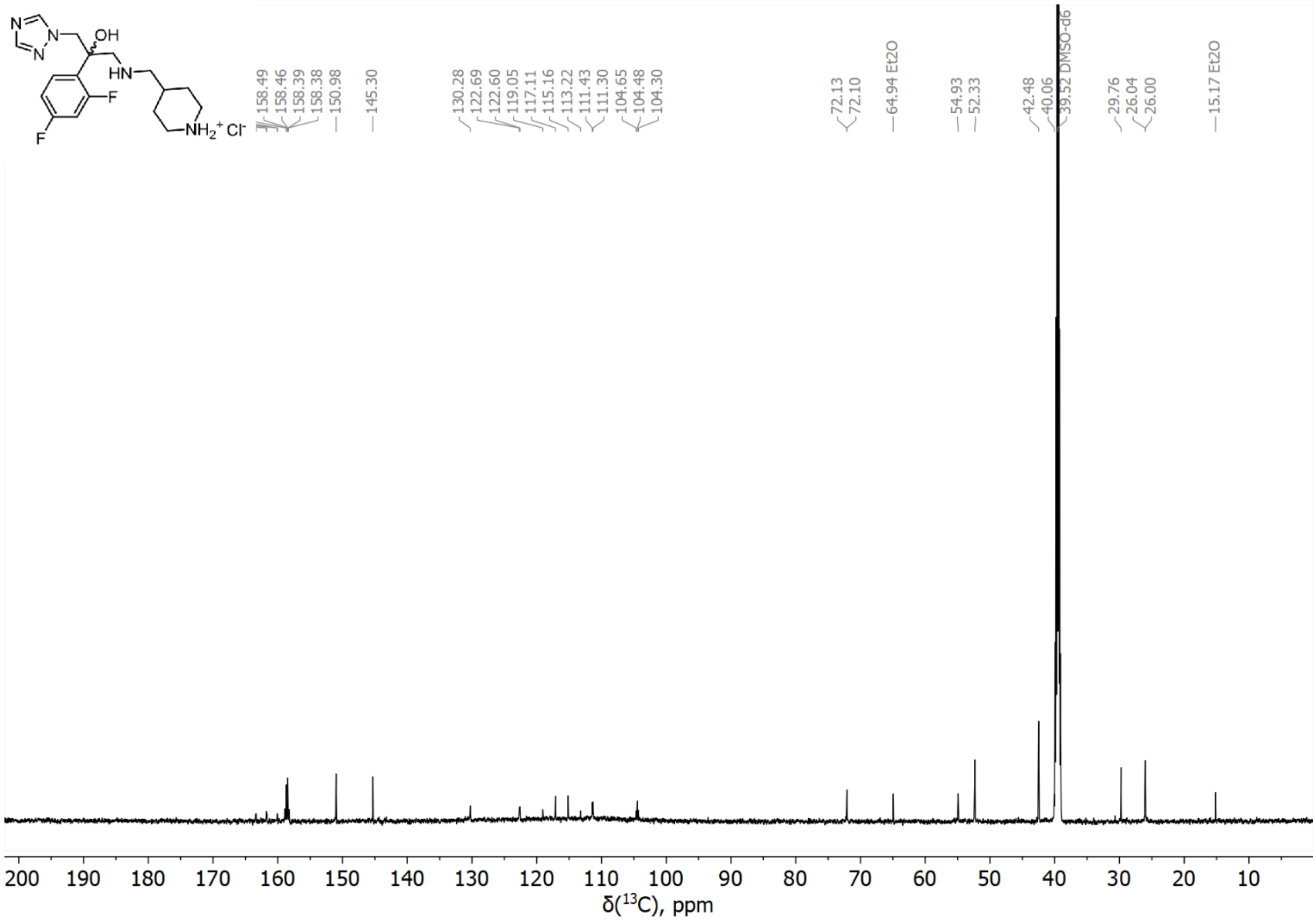

Supplementary Figure S42. ^{13}C NMR spectrum of **4g**

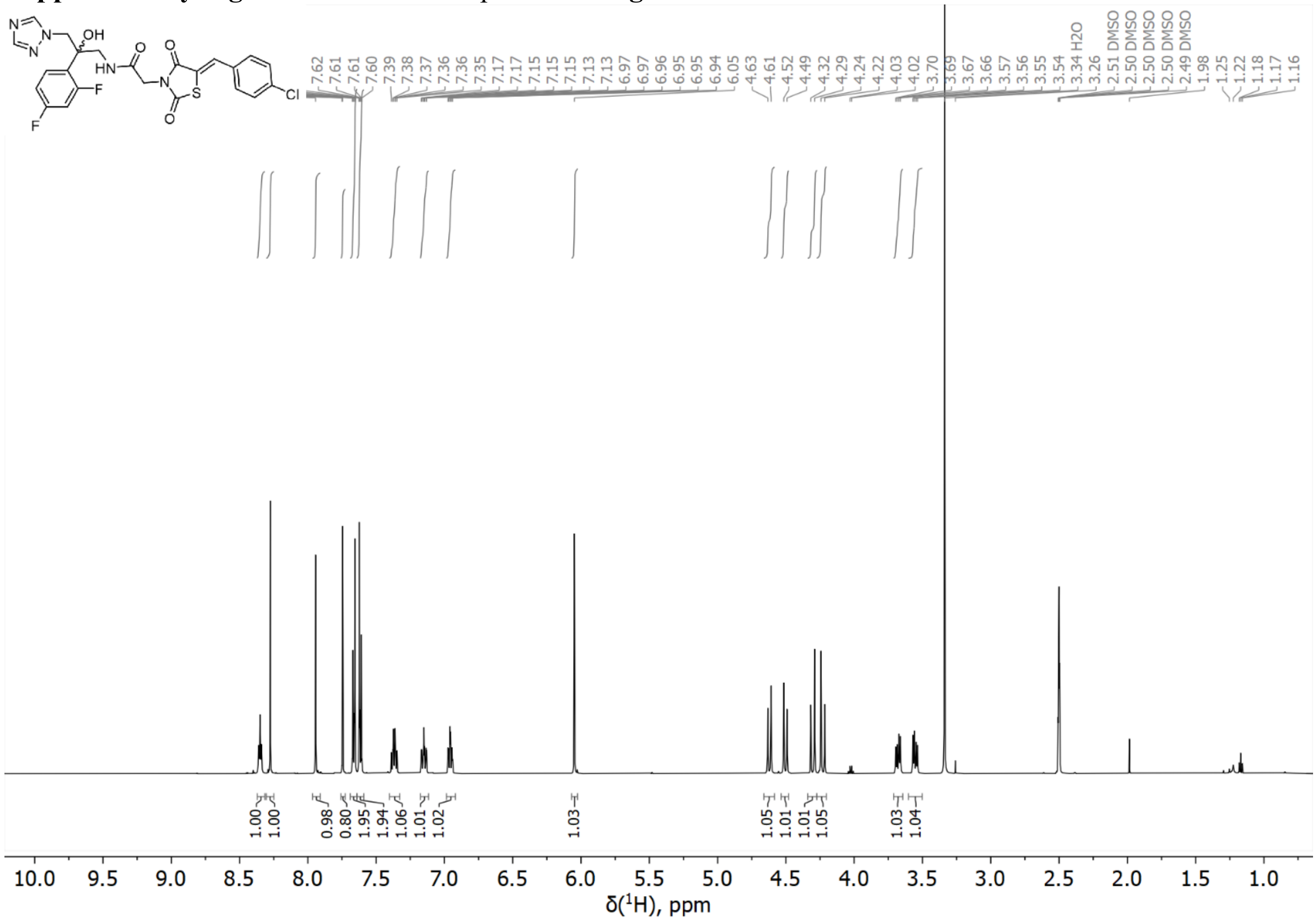

Supplementary Figure S43. ^{1}H NMR spectrum of **6a**

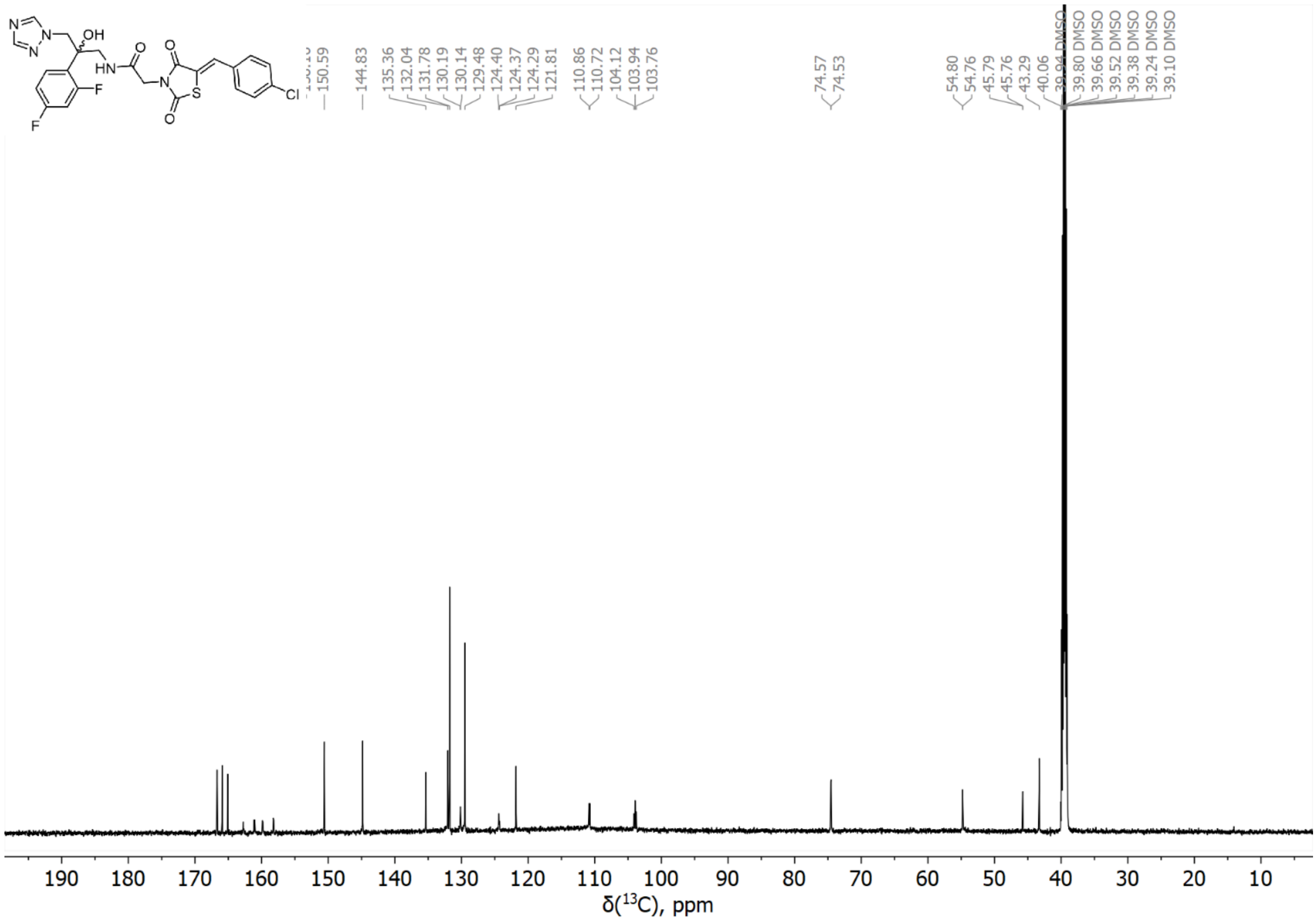

Supplementary Figure S44. ^{13}C NMR spectrum of **6a**

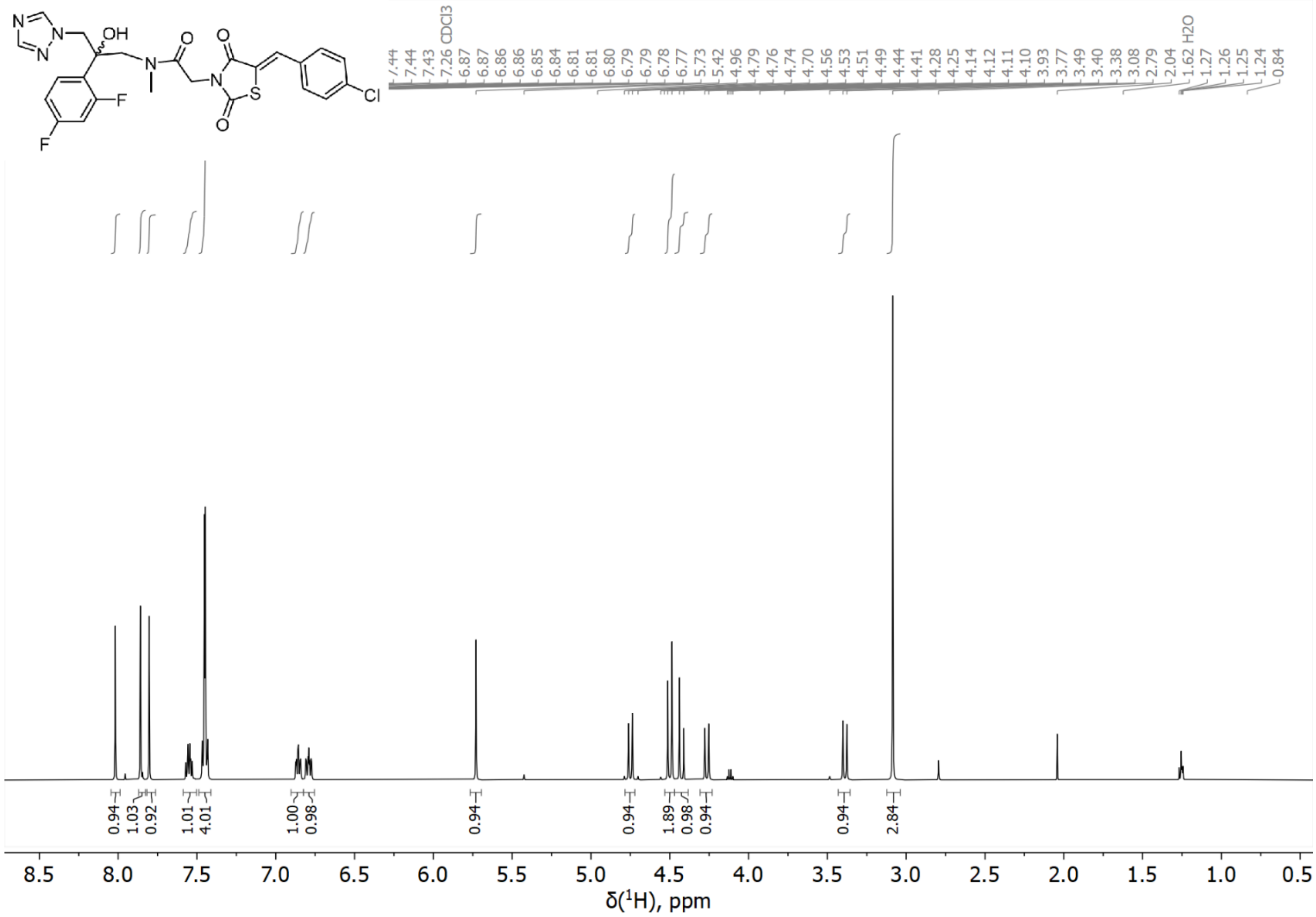

Supplementary Figure S45. ^{1}H NMR spectrum of **6b**

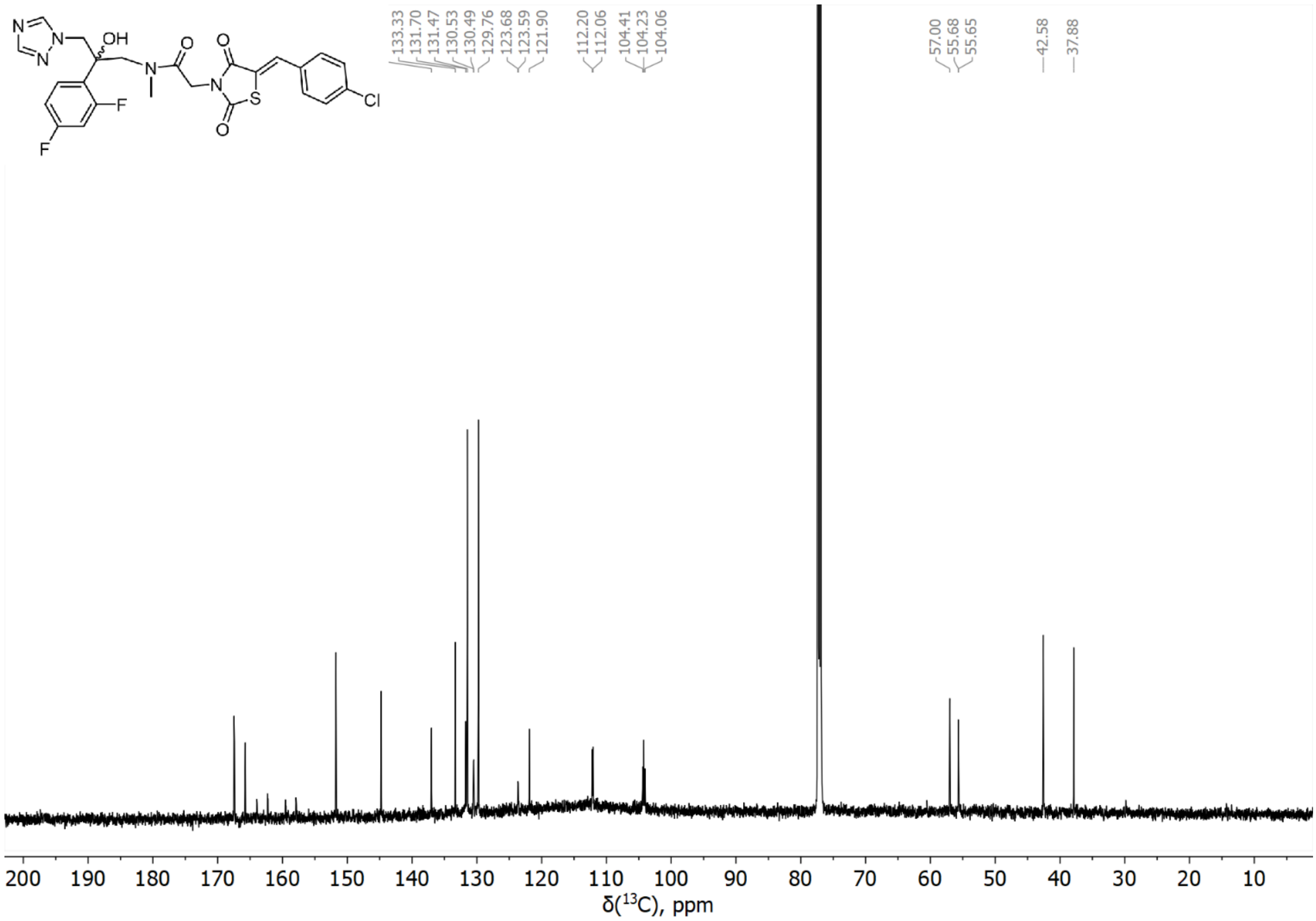

Supplementary Figure S46. ^{13}C NMR spectrum of **6b**

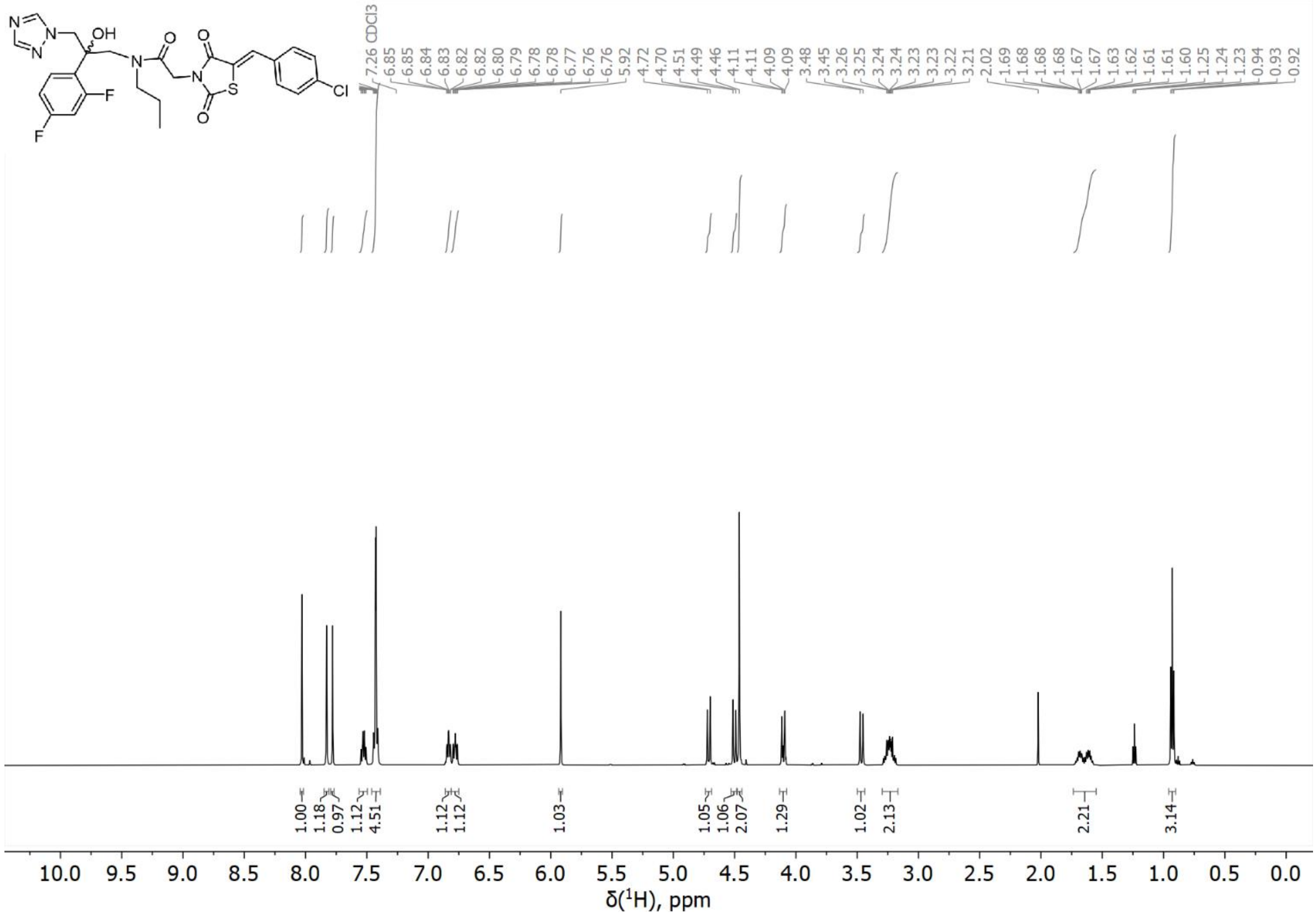

Supplementary Figure S47. ^{1}H NMR spectrum of **6c**

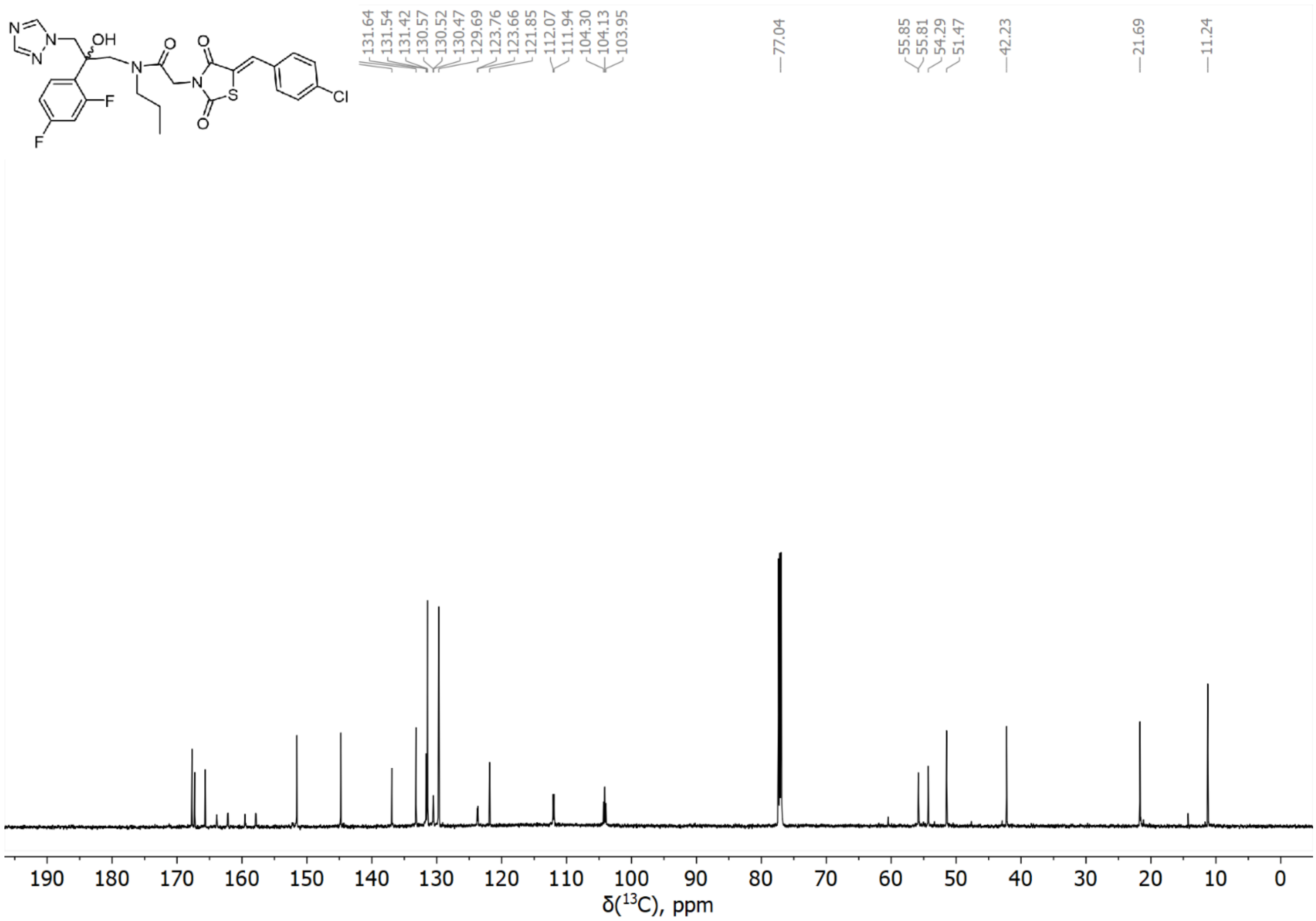

Supplementary Figure S48. ^{13}C NMR spectrum of **6c**

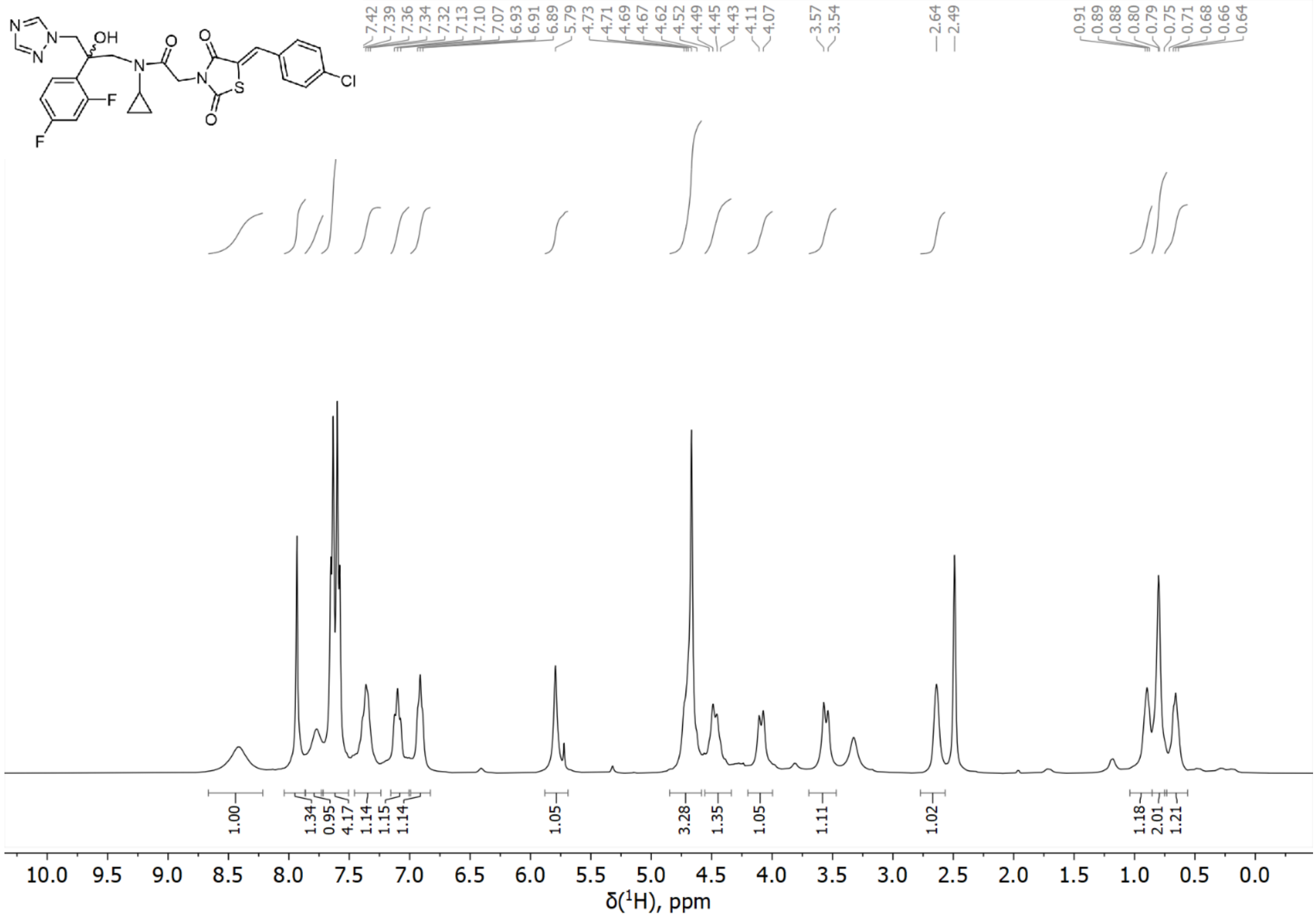

Supplementary Figure S49. ^{1}H NMR spectrum of **6d**

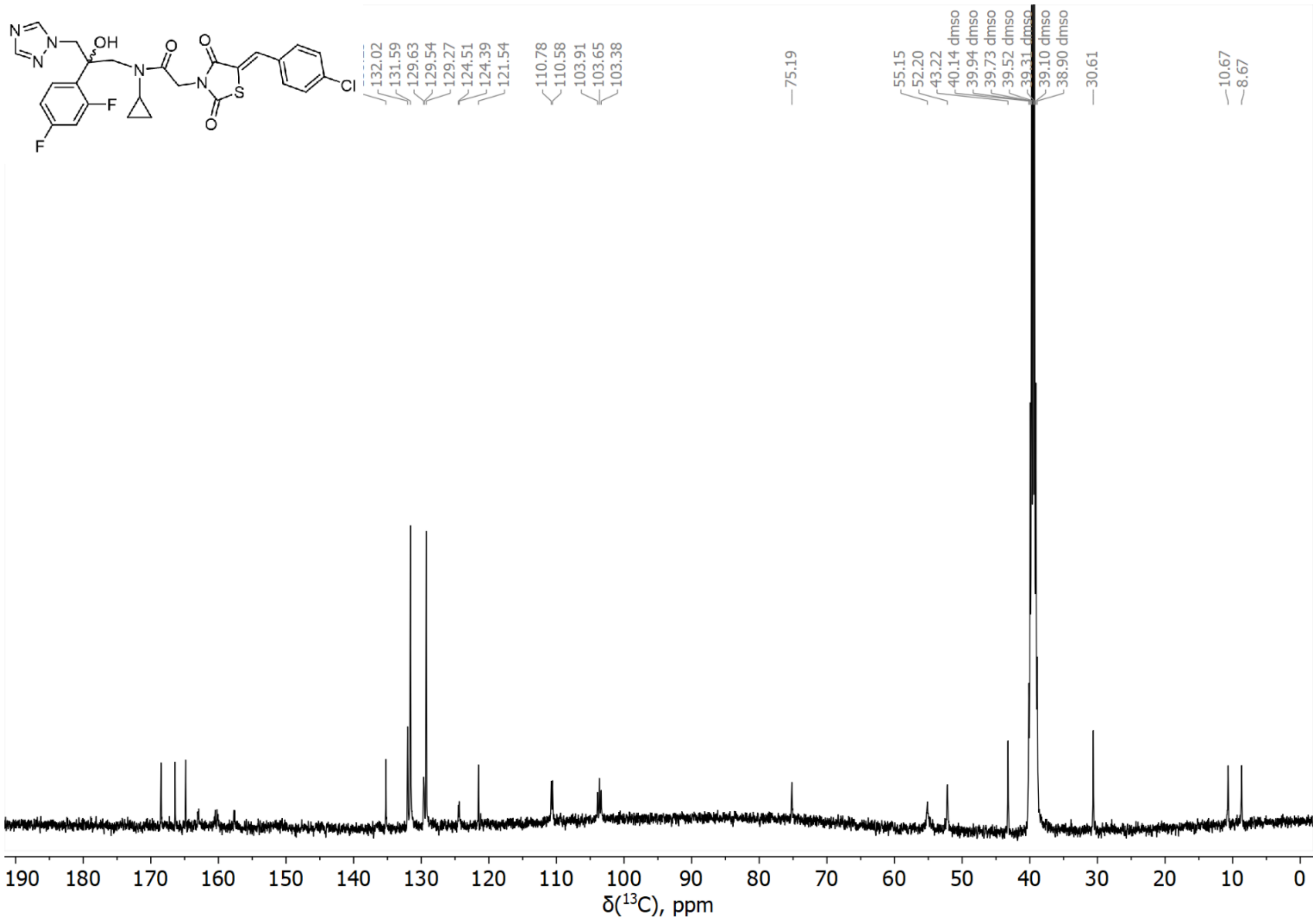

Supplementary Figure S50. ^{13}C NMR spectrum of **6d**

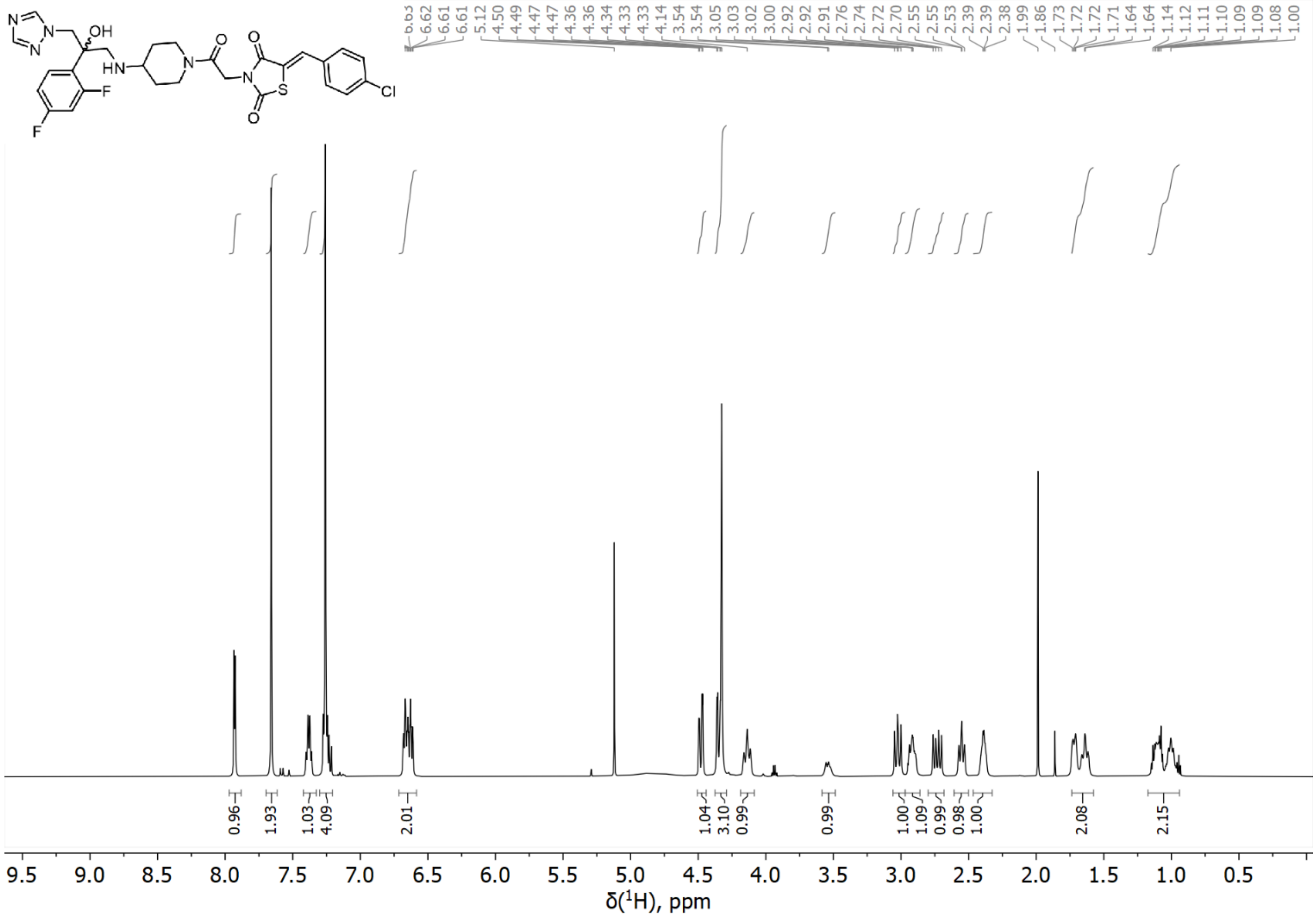

Supplementary Figure S51. ^{1}H NMR spectrum of **7e**

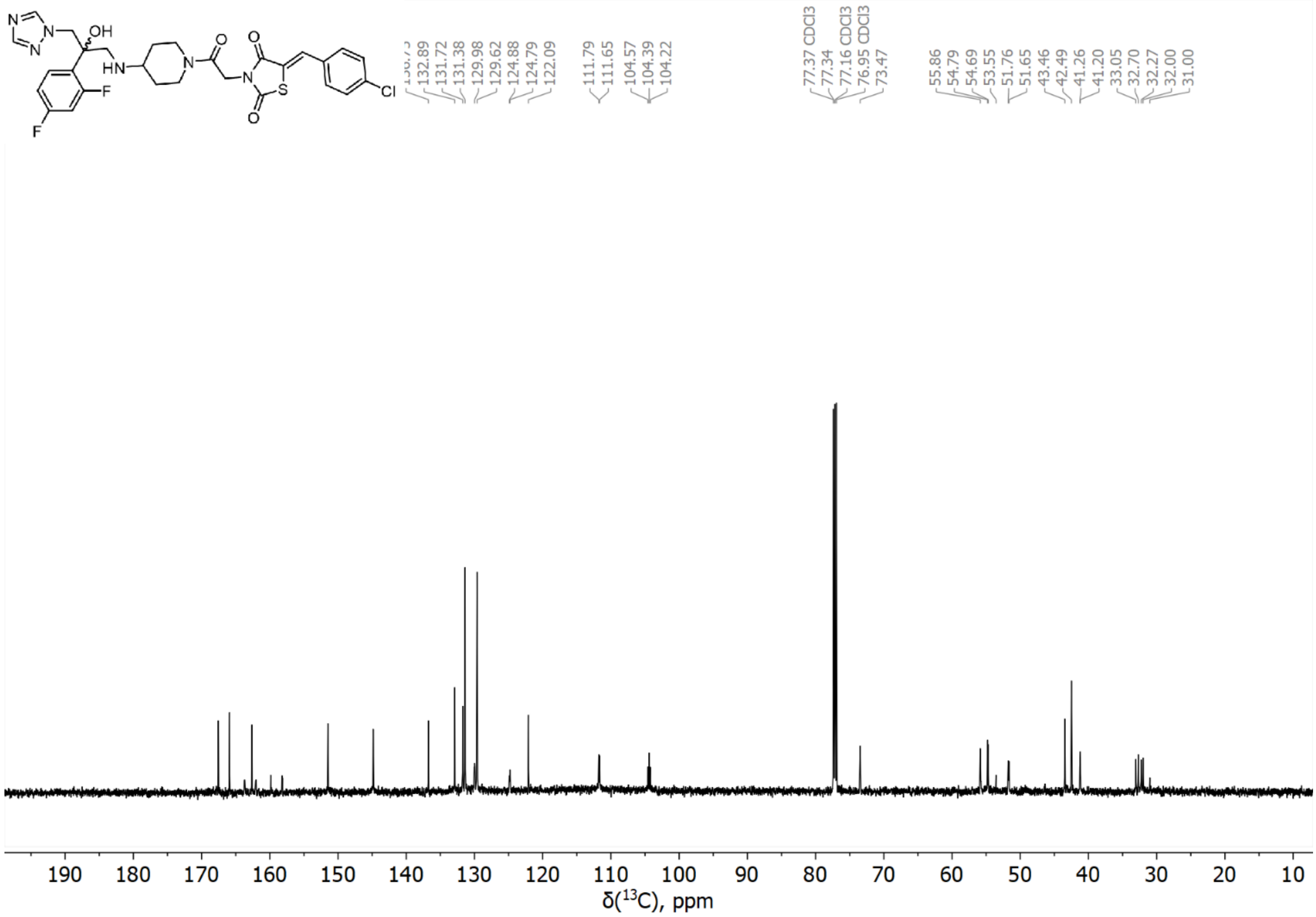

Supplementary Figure S52. ^{13}C NMR spectrum of **7e**

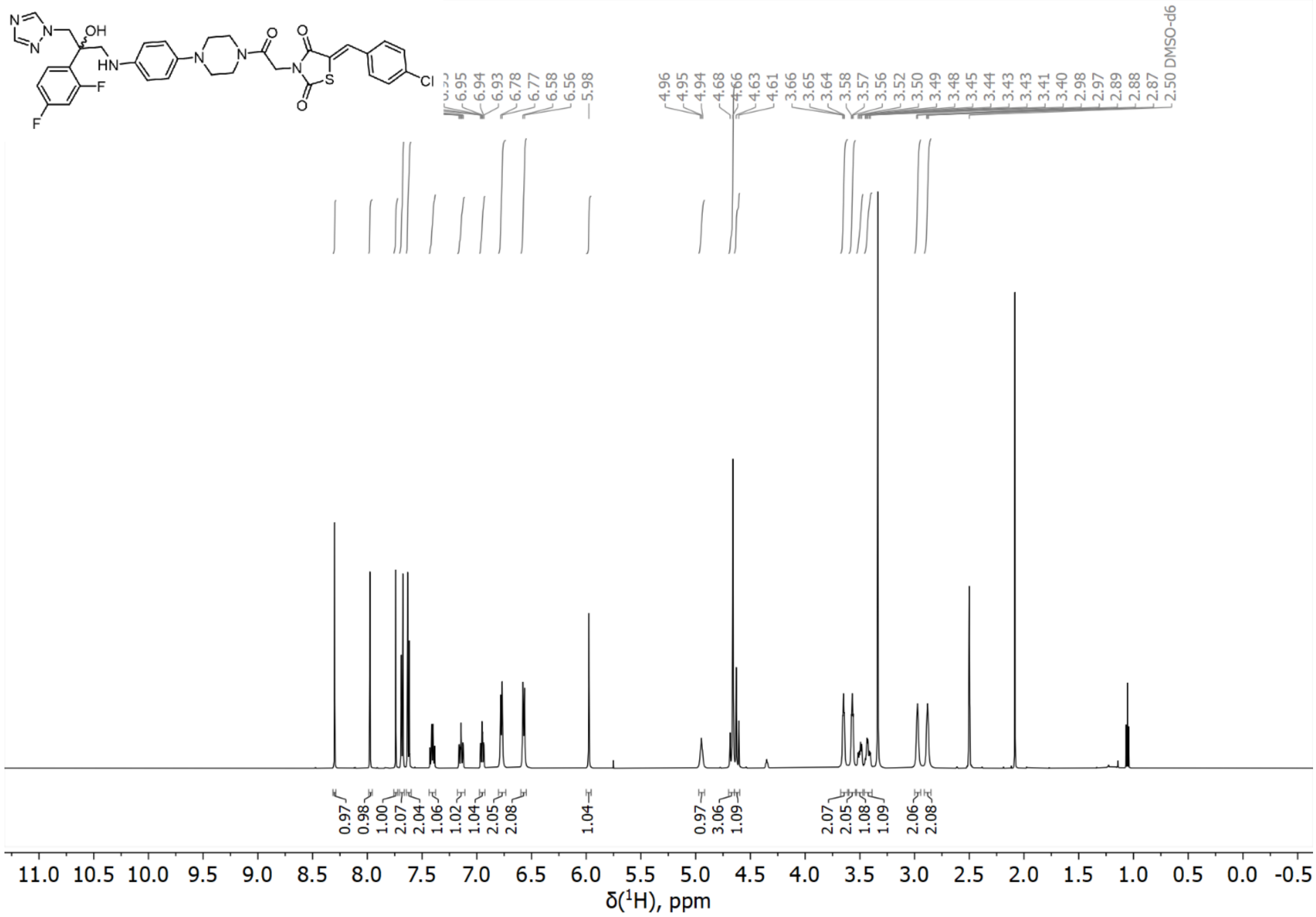

Supplementary Figure S53. ^{1}H spectrum of **8f**

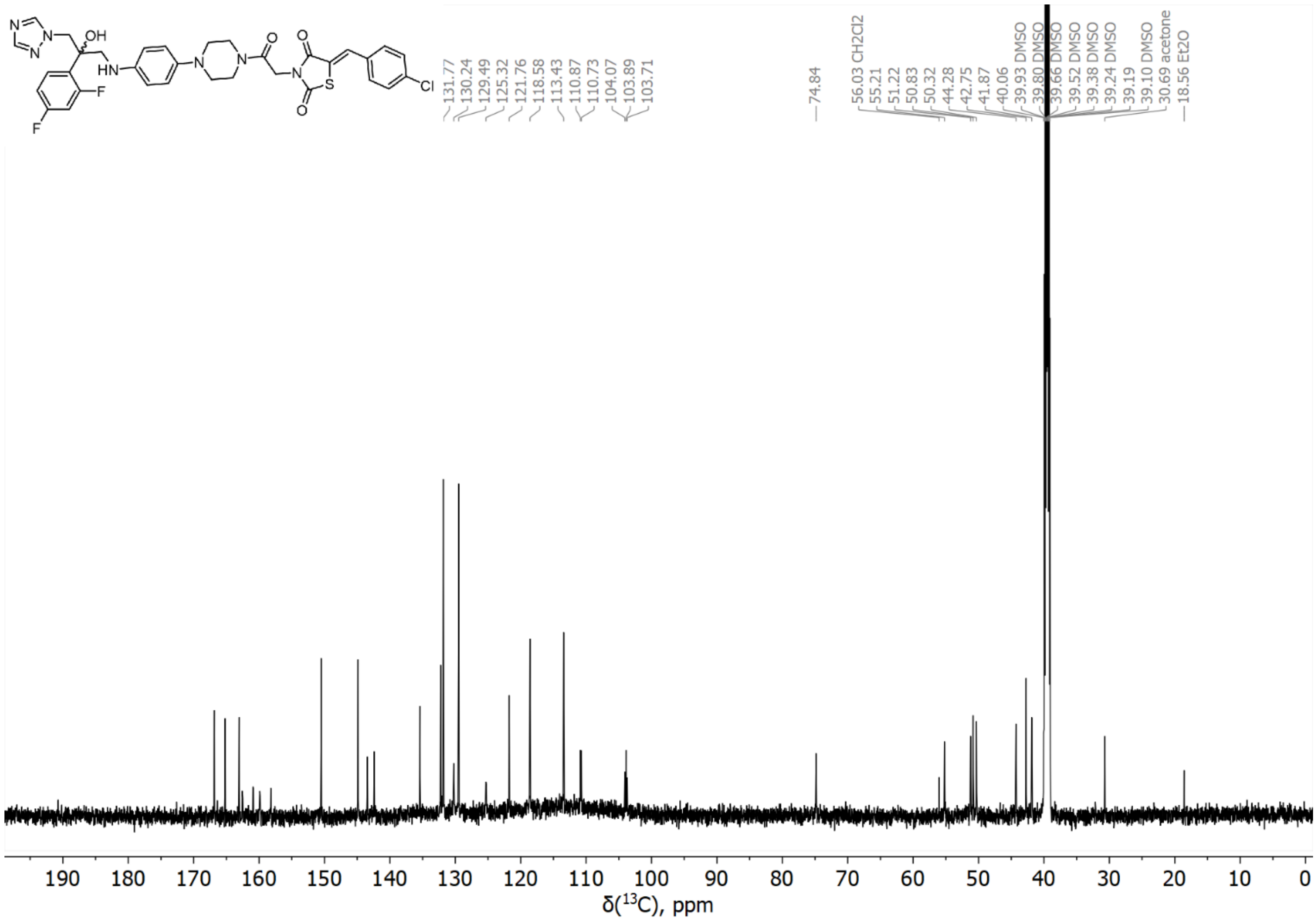

Supplementary Figure S54. ^{13}C NMR spectrum of **8f**

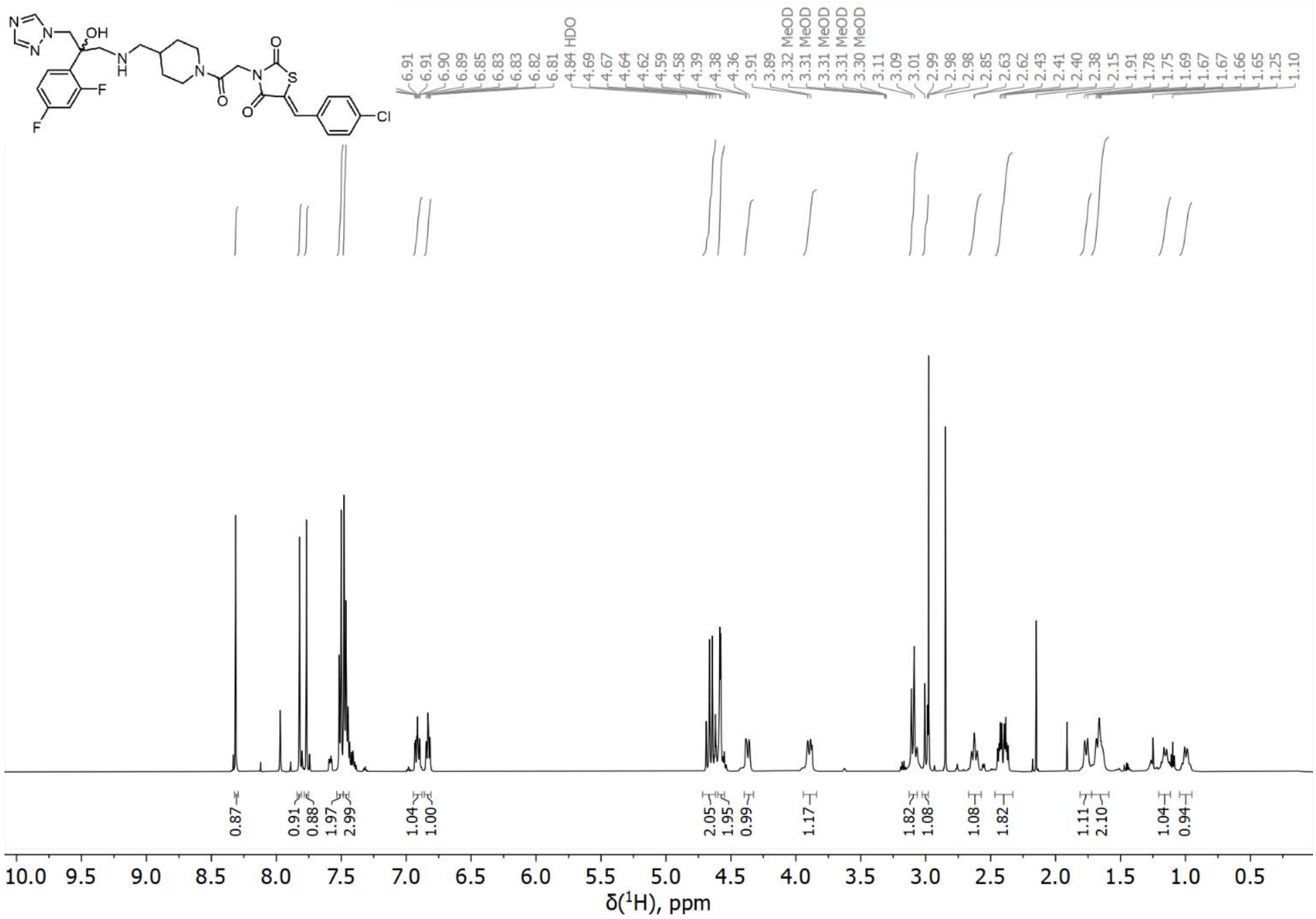

Supplementary Figure S55. ^{1}H NMR spectrum of **9g**

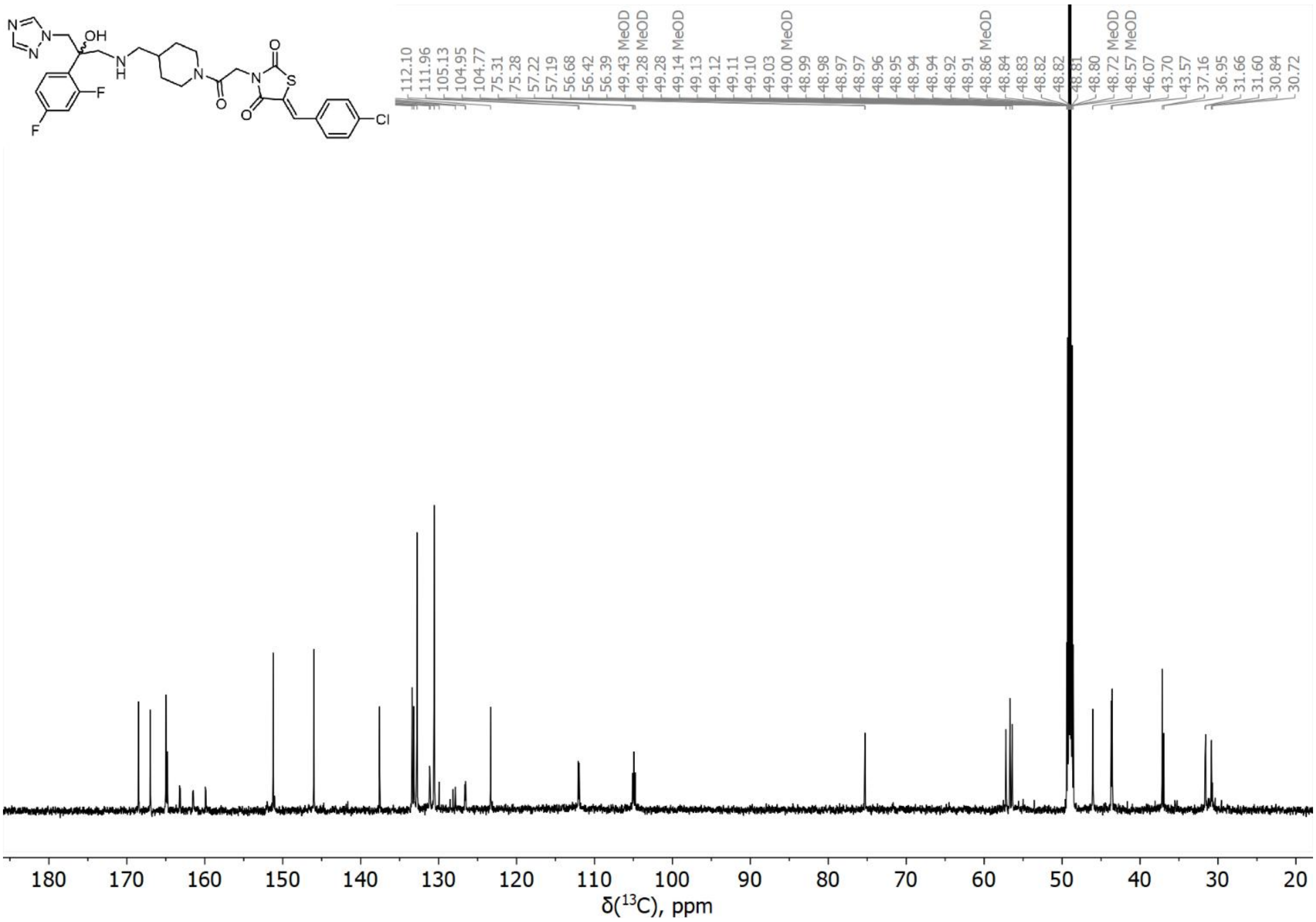

Supplementary Figure S56. ^{13}C NMR spectrum of **9g**

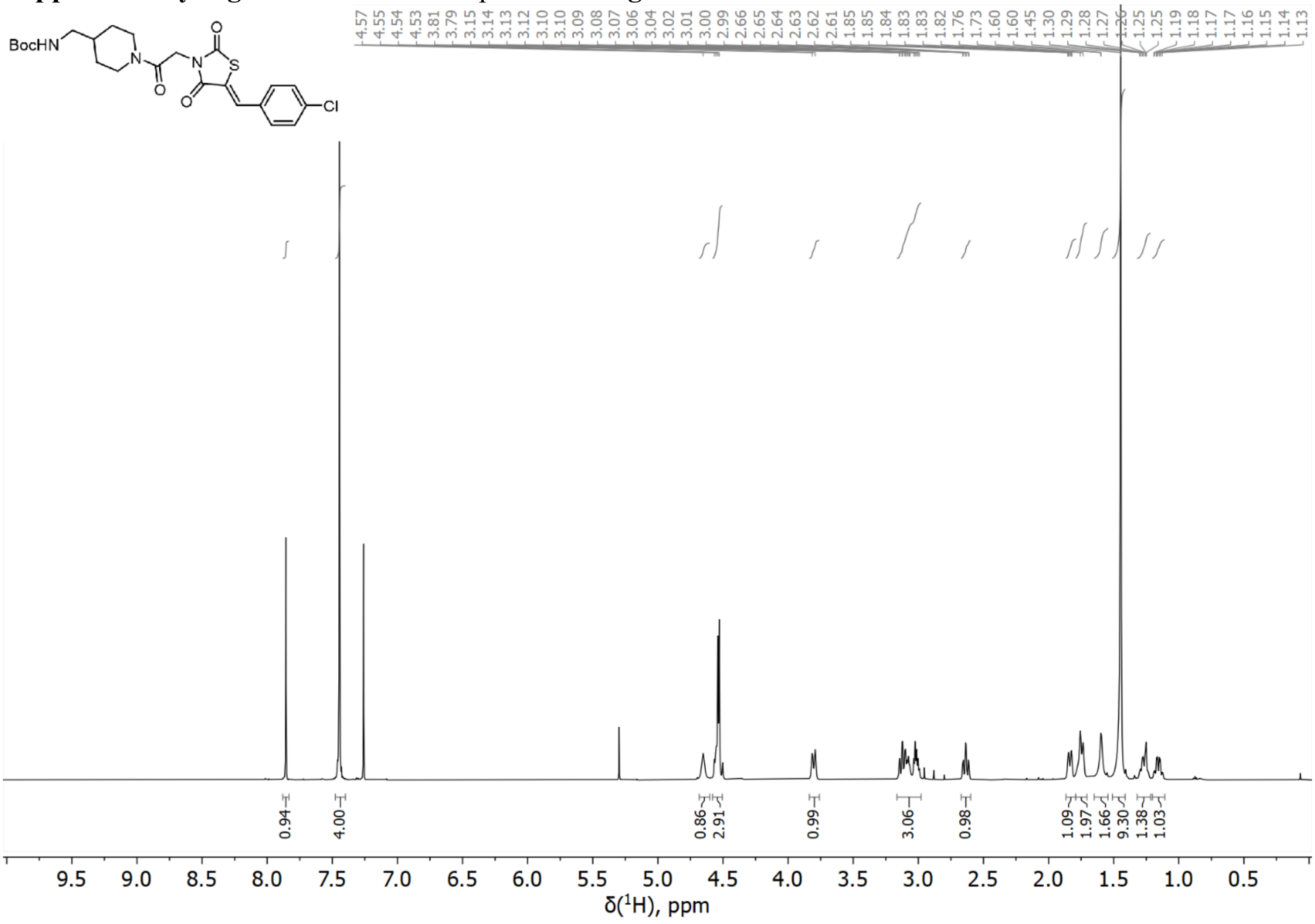

Supplementary Figure S57. ^{1}H NMR spectrum of **11**

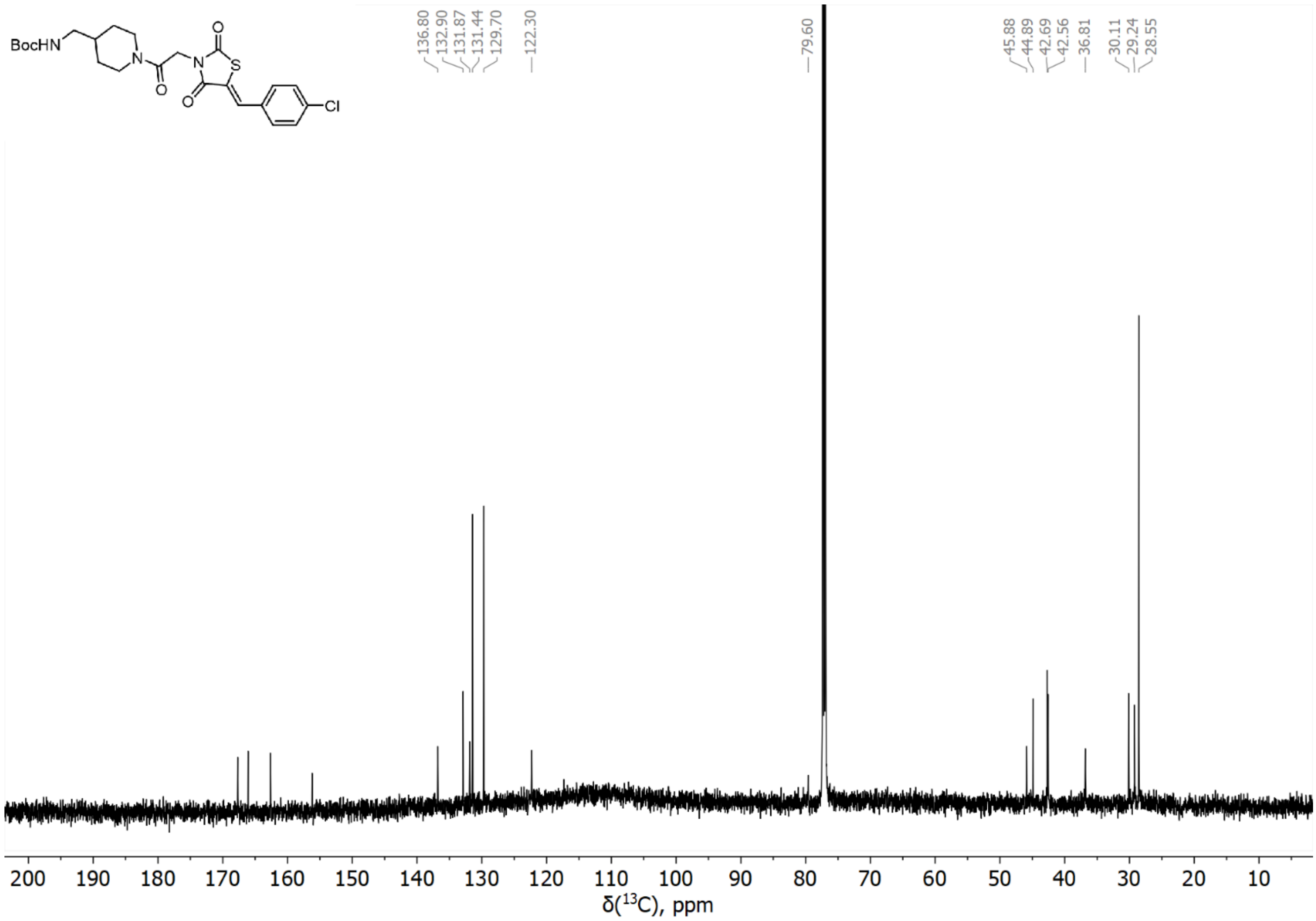

Supplementary Figure S58. ^{1}H NMR spectrum of **11**

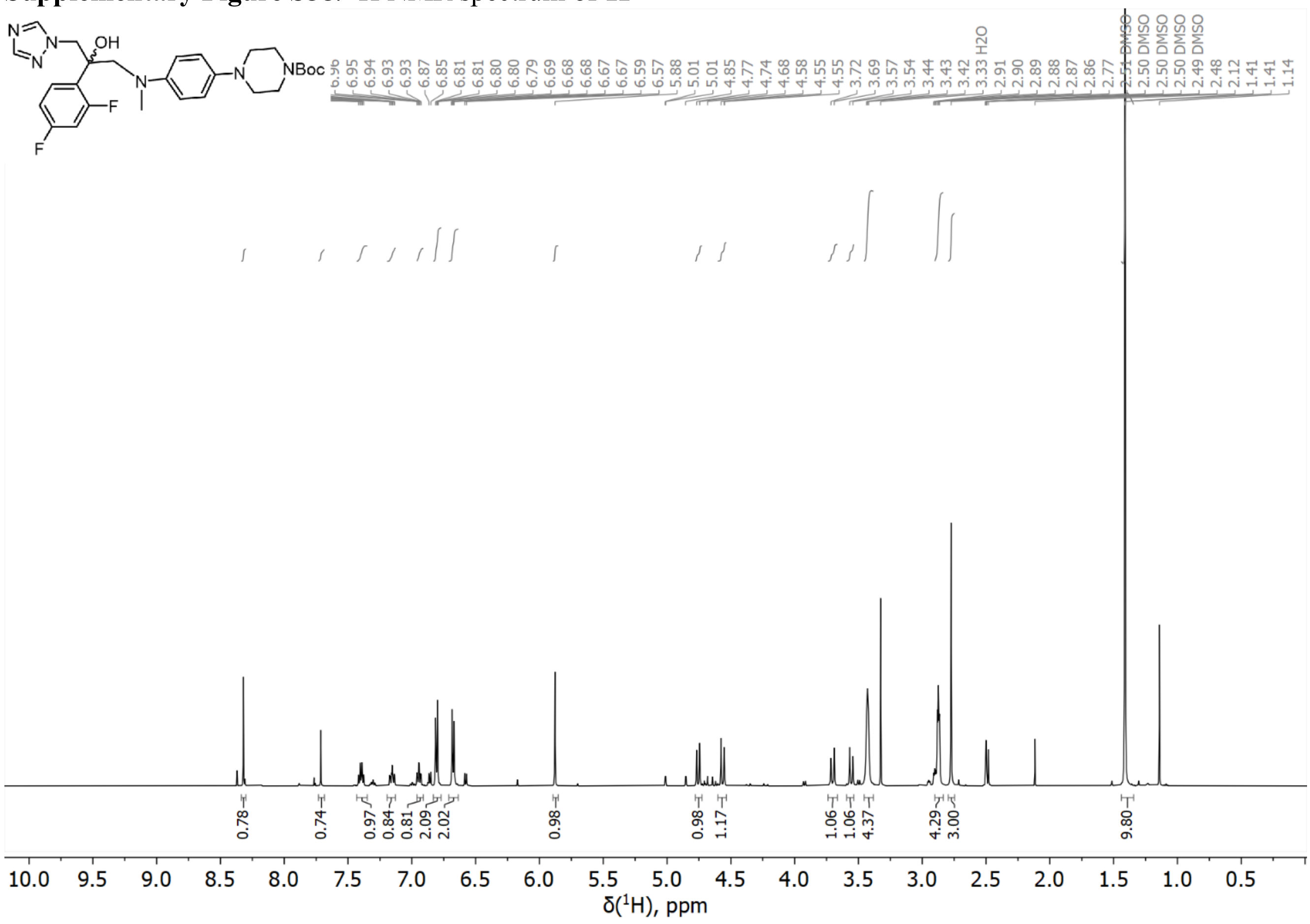

Supplementary Figure S59. ^{1}H NMR spectrum of **12f**

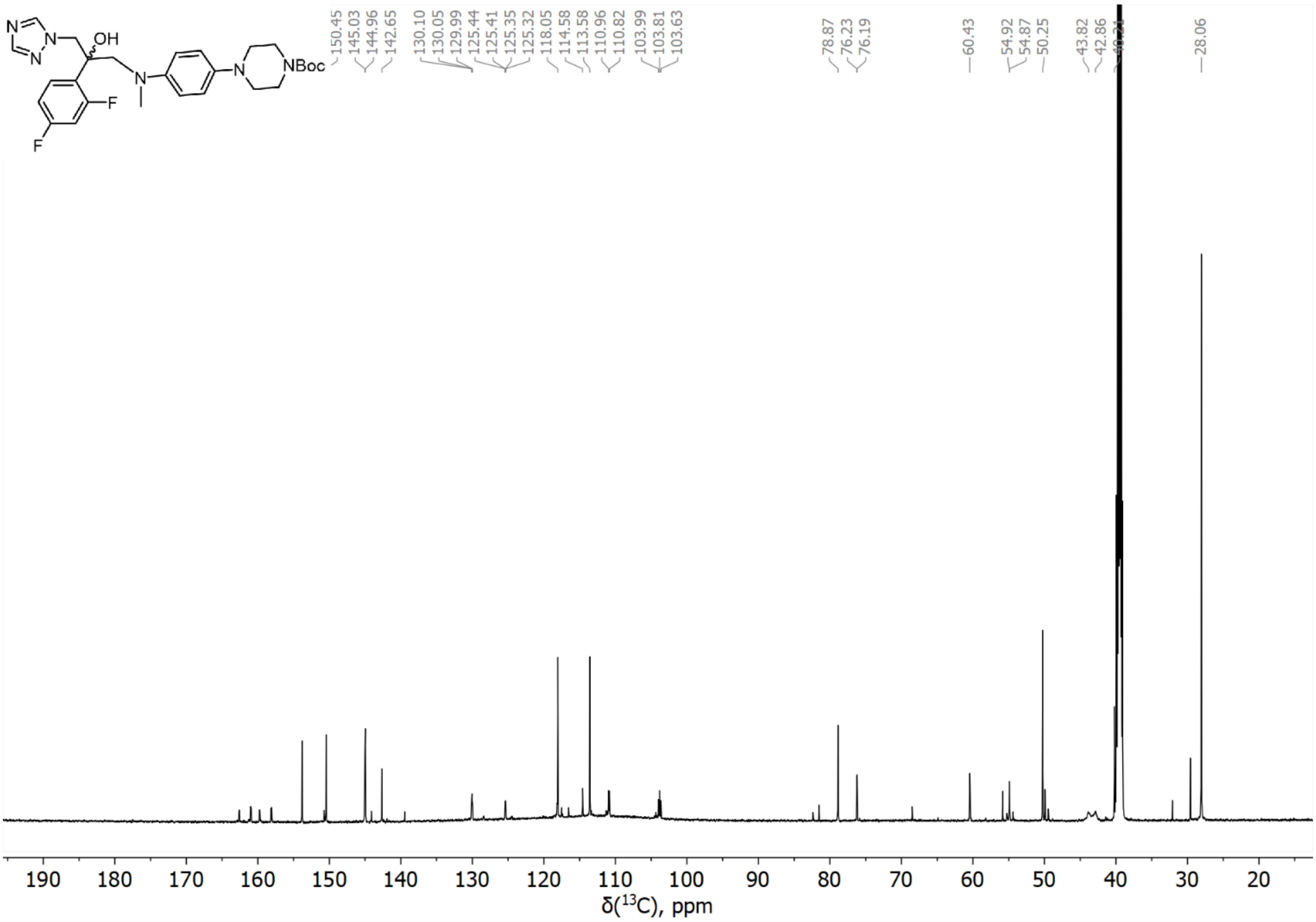

Supplementary Figure S60. ^{13}C NMR spectrum of **12f**

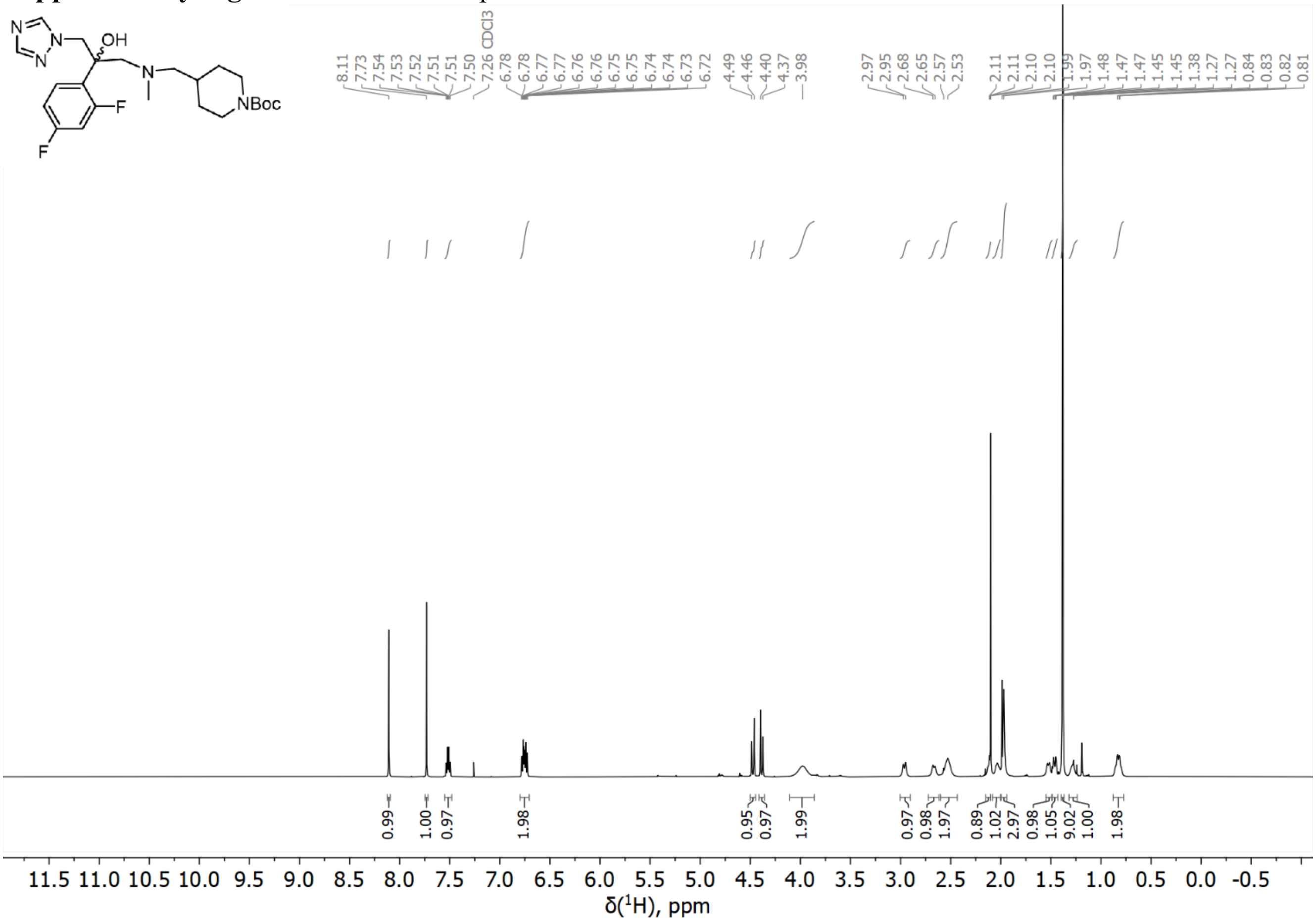

Supplementary Figure S61. ^{1}H NMR spectrum of **12g**

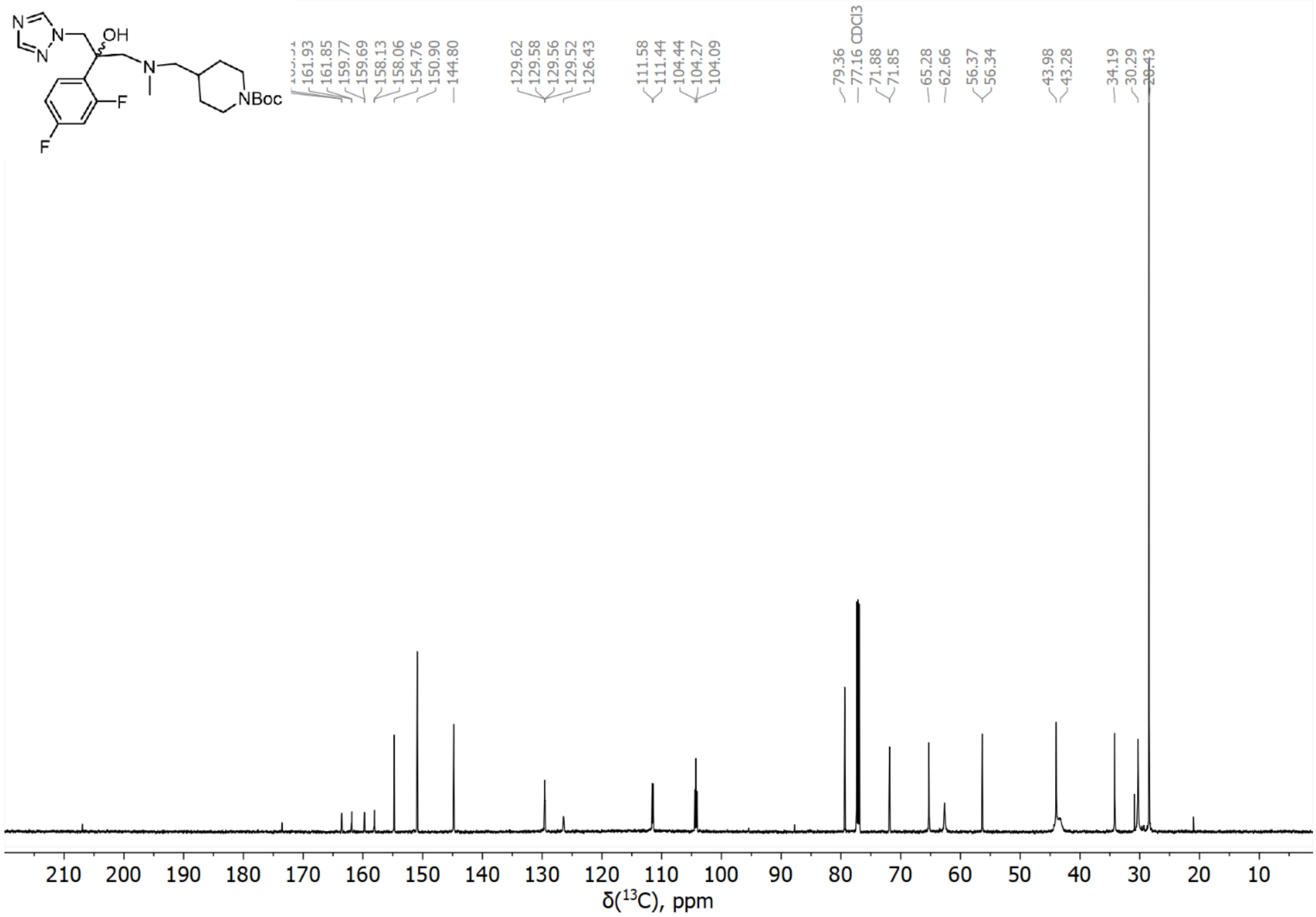

Supplementary Figure S62. ^{13}C NMR spectrum of **12g**

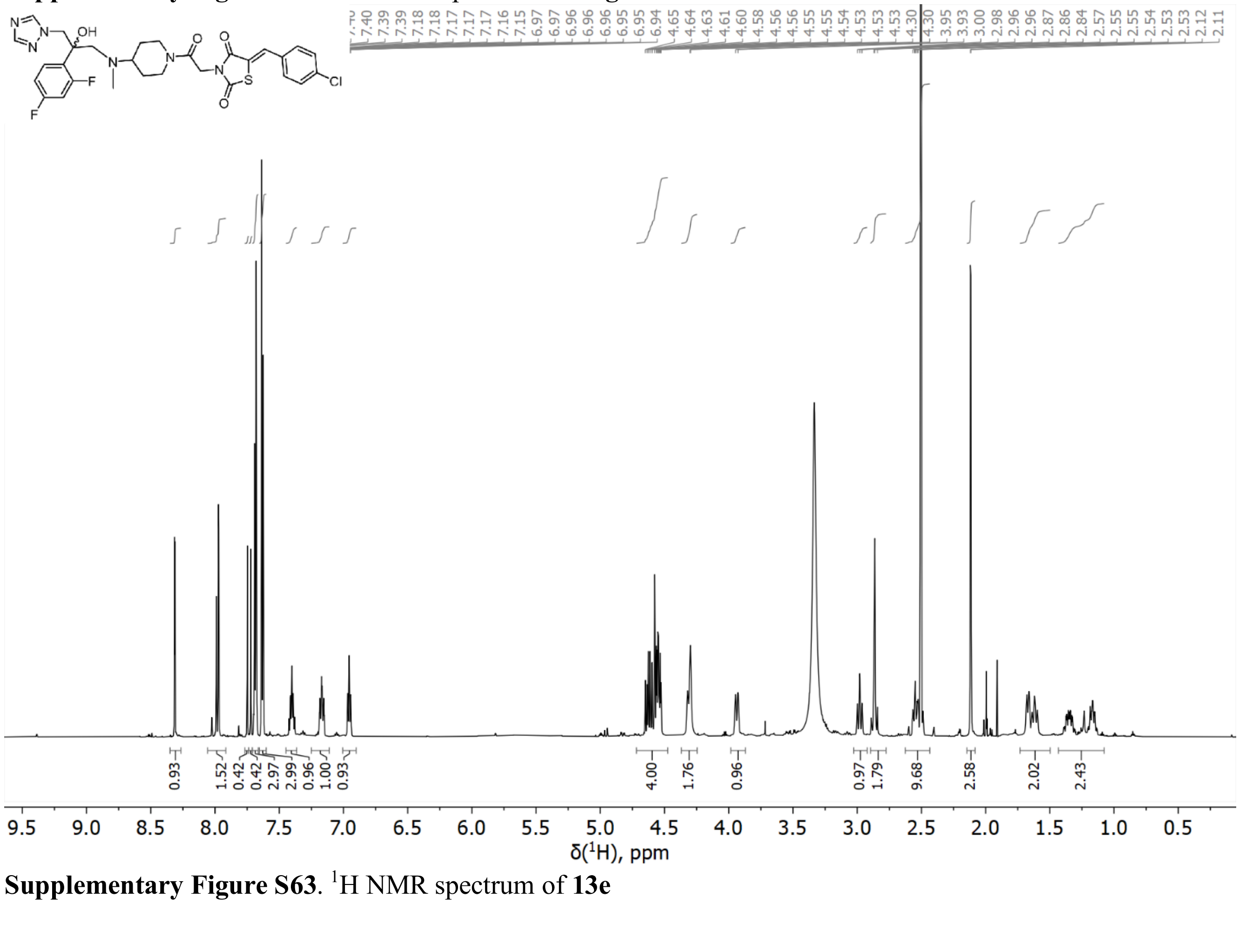

Supplementary Figure S63. ^{1}H NMR spectrum of **13e**

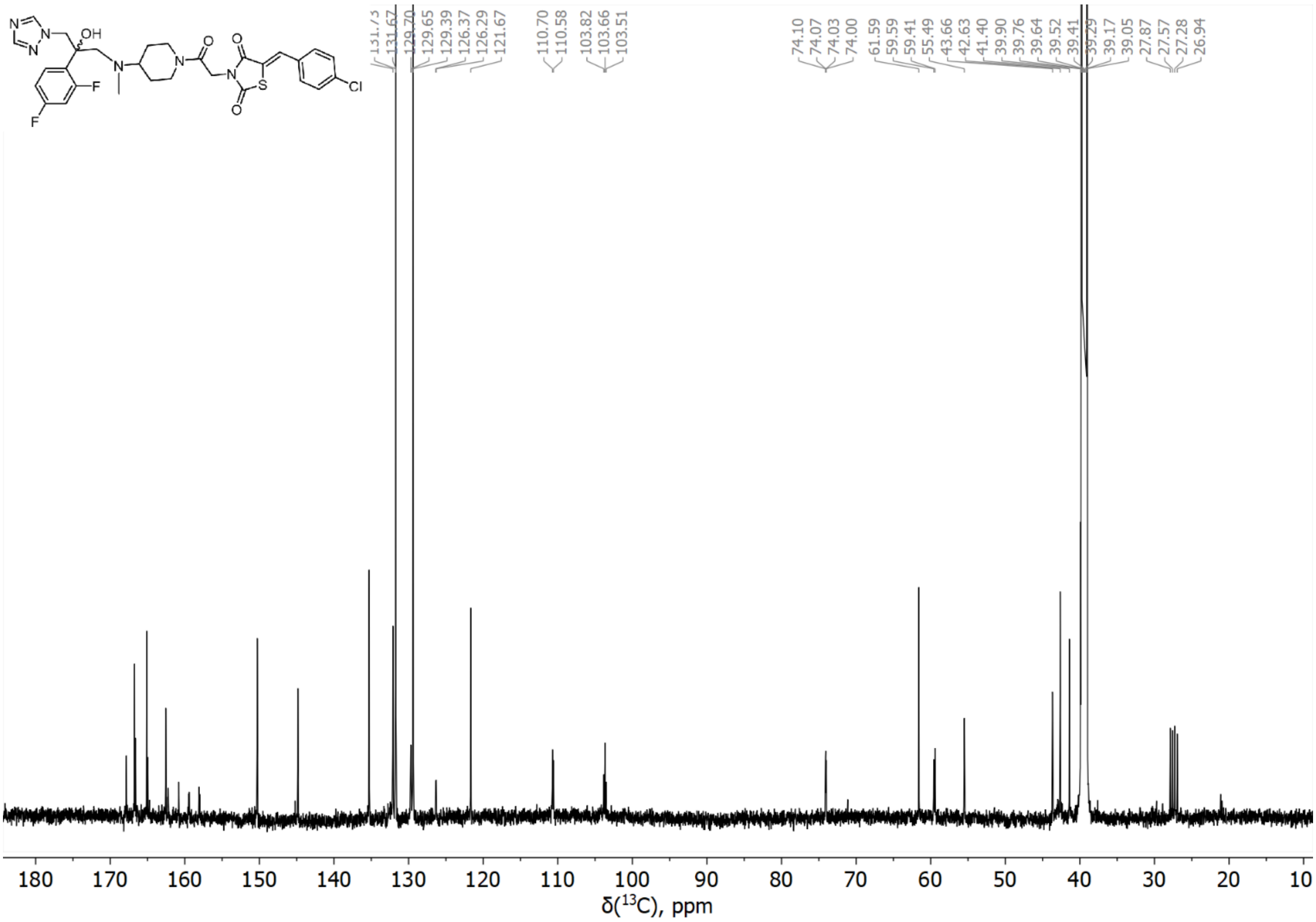

Supplementary Figure S64. ^{13}C NMR spectrum of **13e**

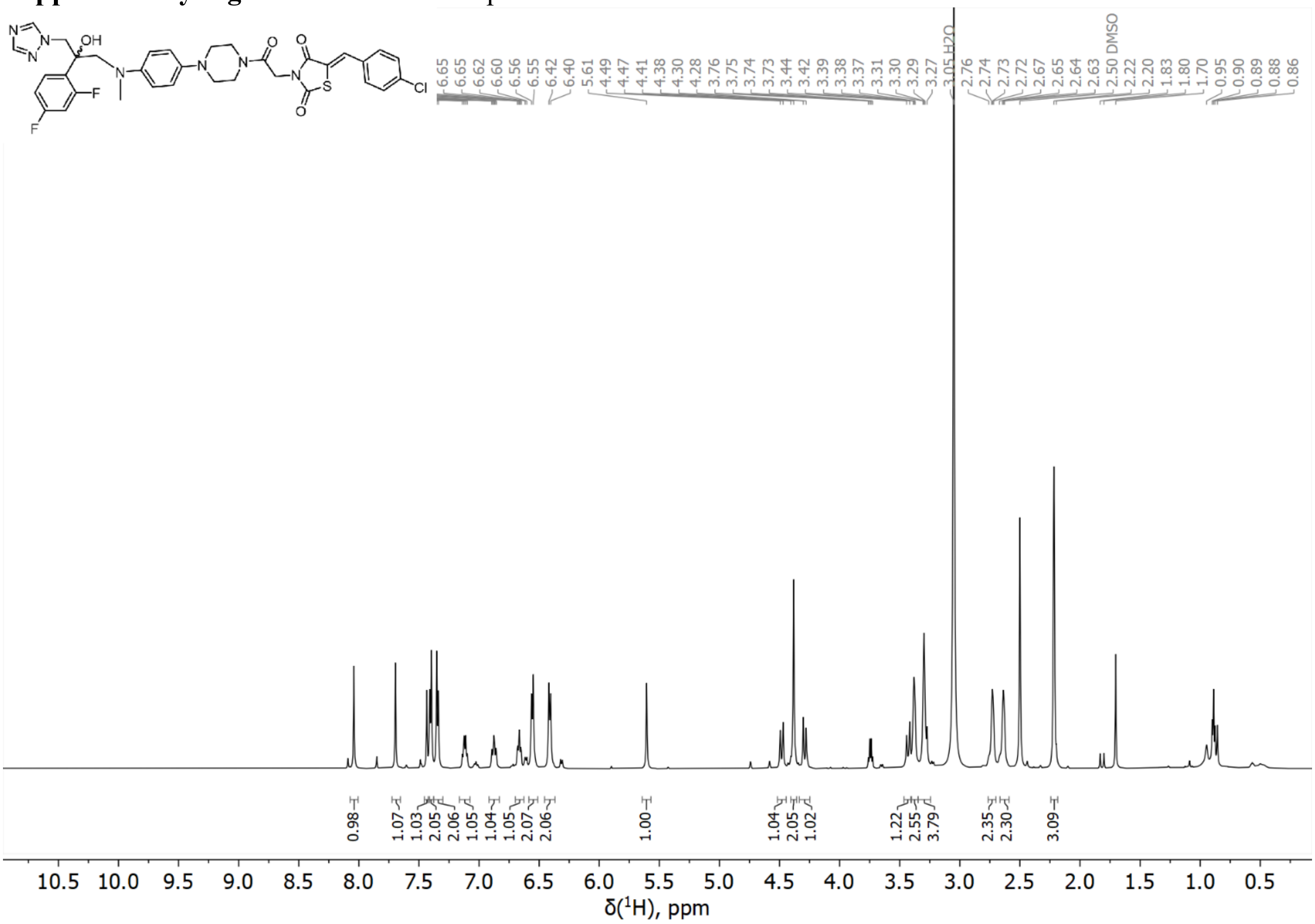

Supplementary Figure S65. ^{1}H NMR spectrum of **13f**

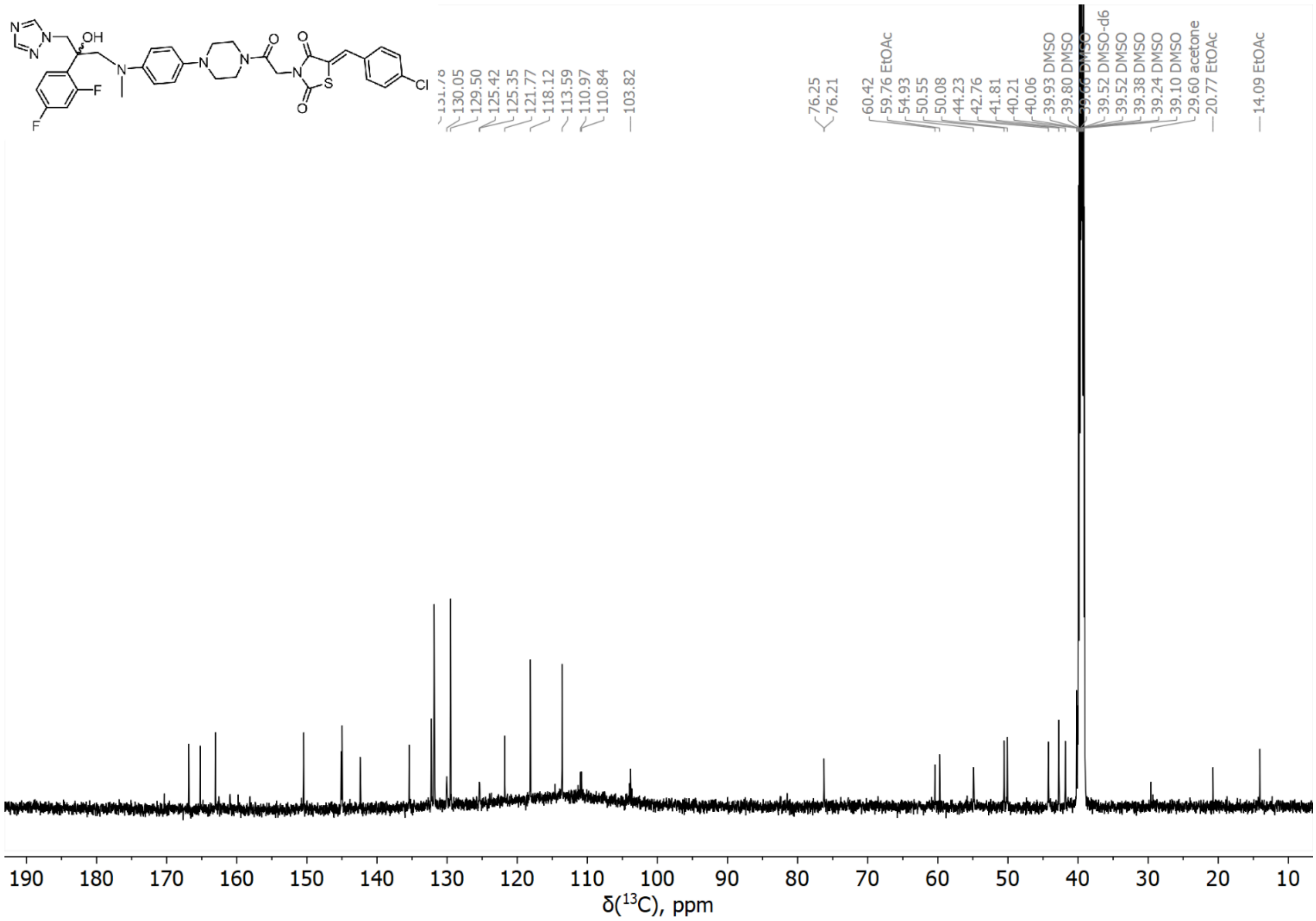

Supplementary Figure S66. ^{13}C NMR spectrum of **13f**

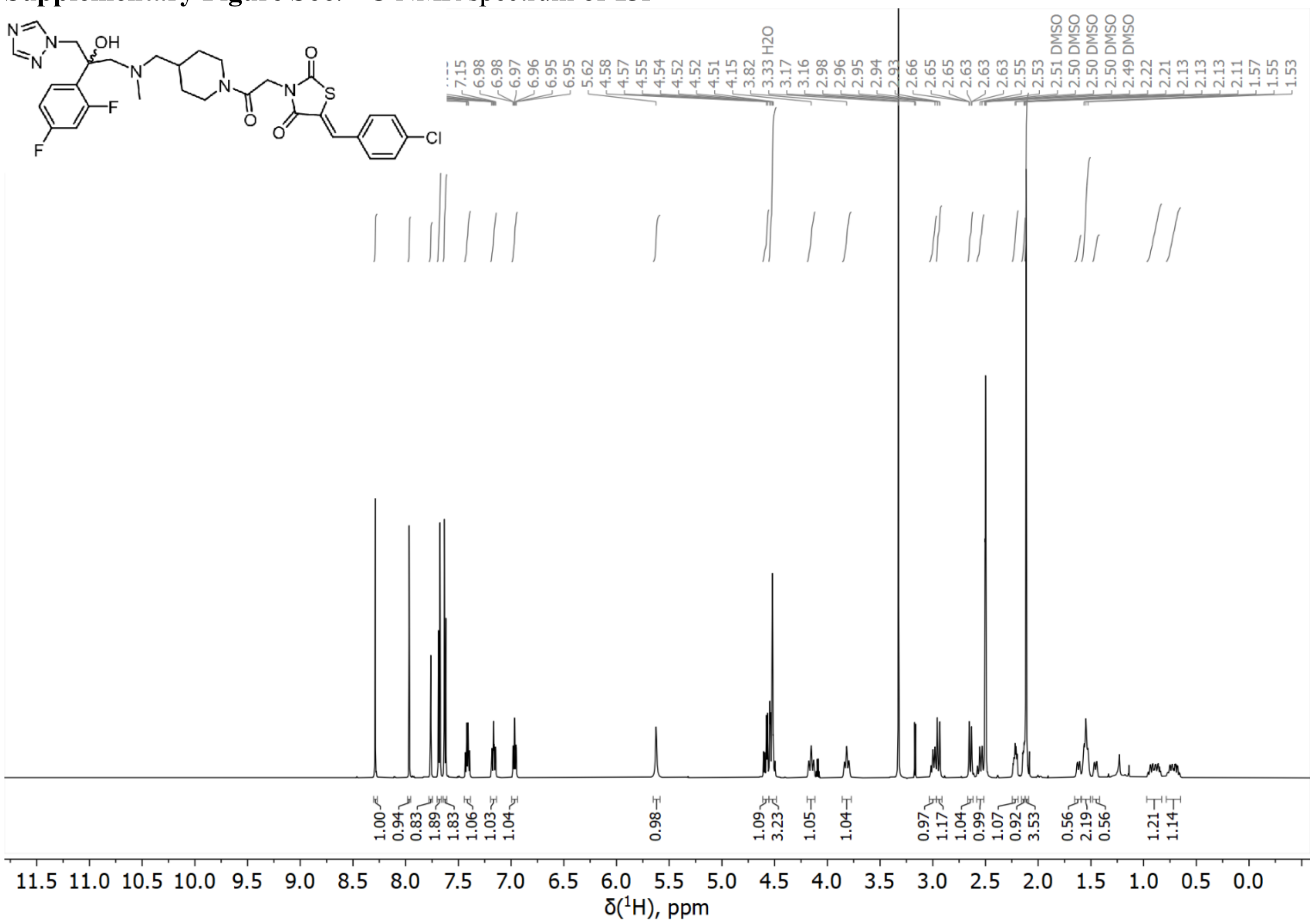

Supplementary Figure S67. ^{1}H NMR spectrum of **13g**

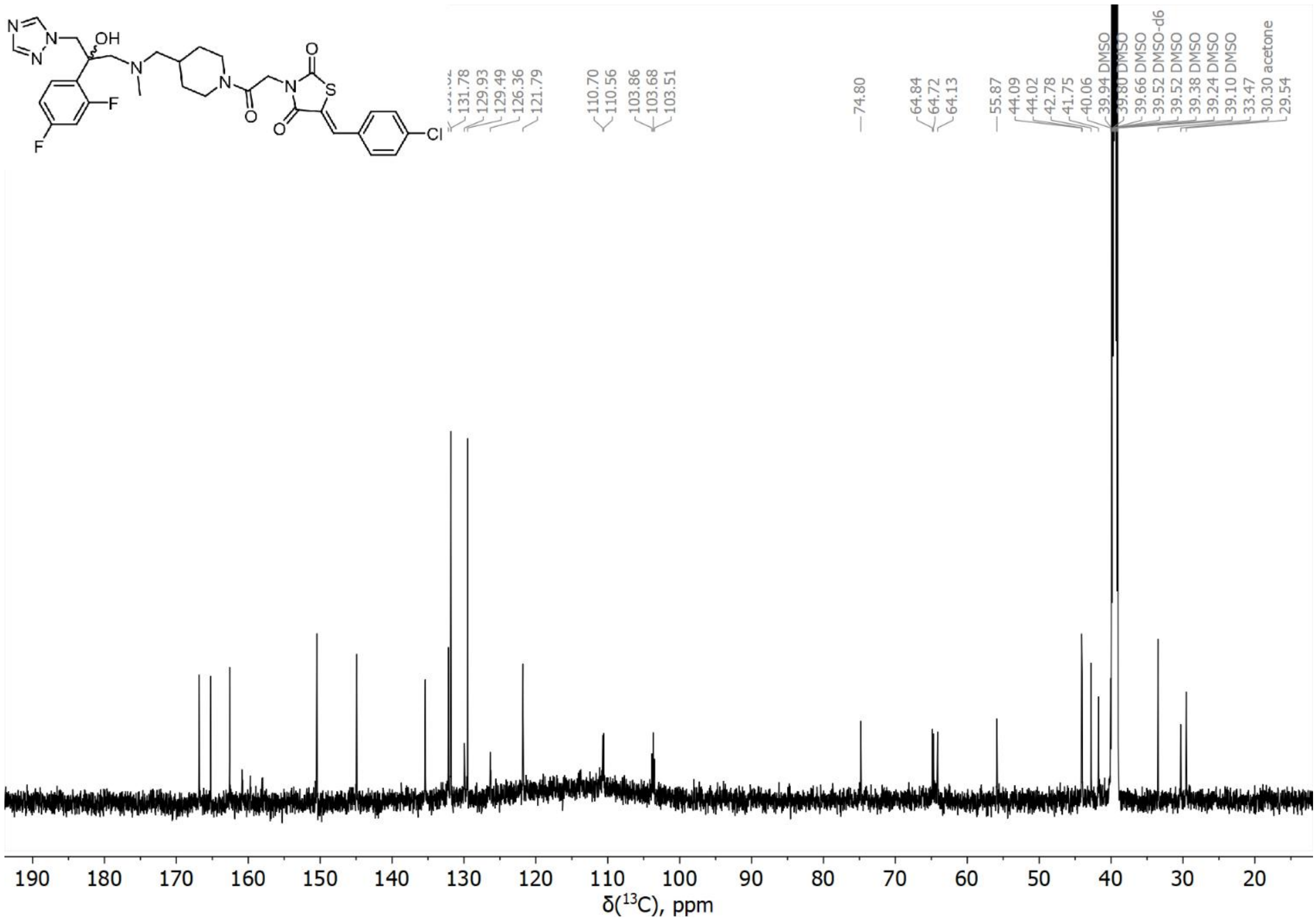

Supplementary Figure S68. ^{13}C NMR spectrum of **13g**

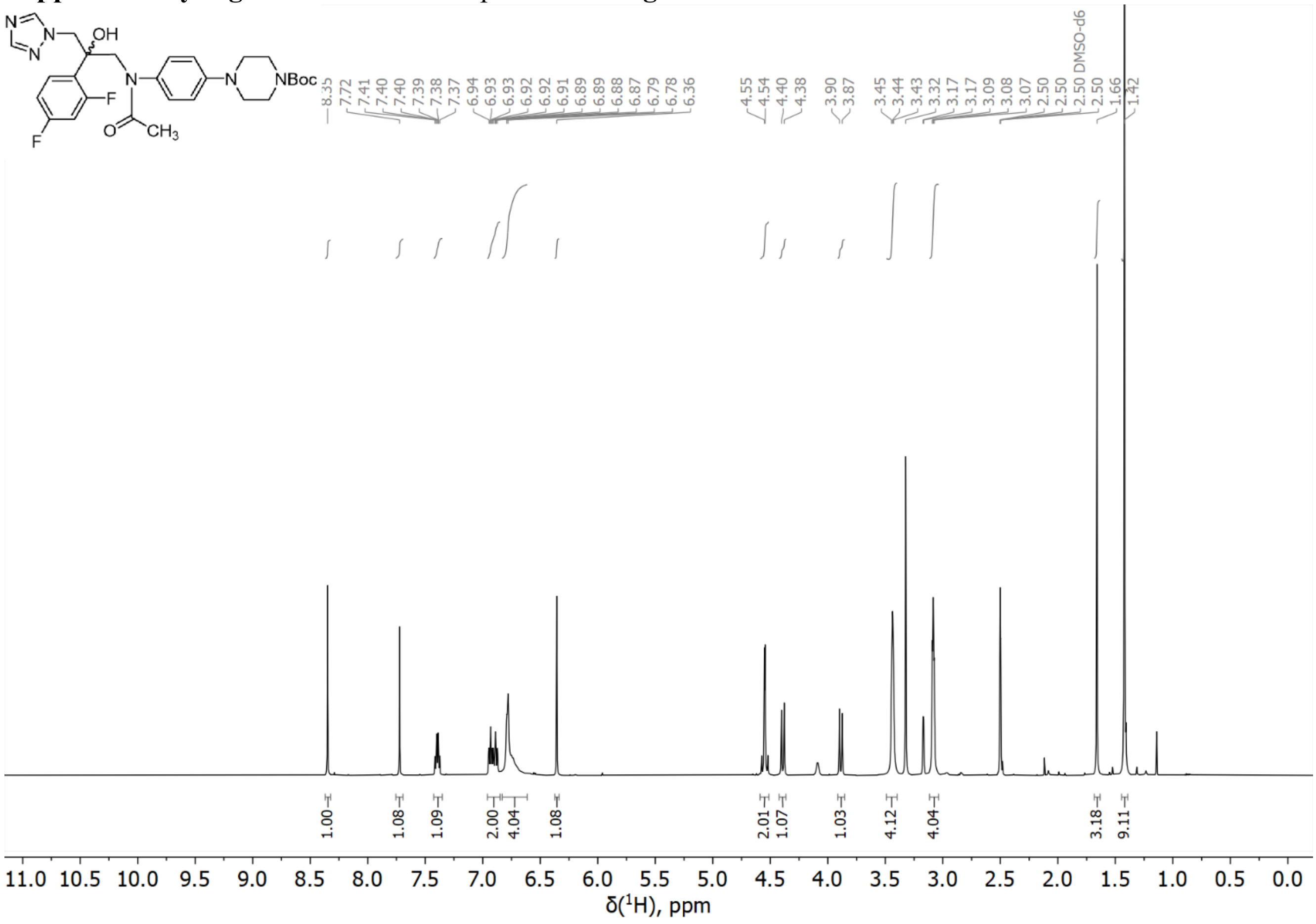

Supplementary Figure S69. ^{1}H NMR spectrum of **14f**

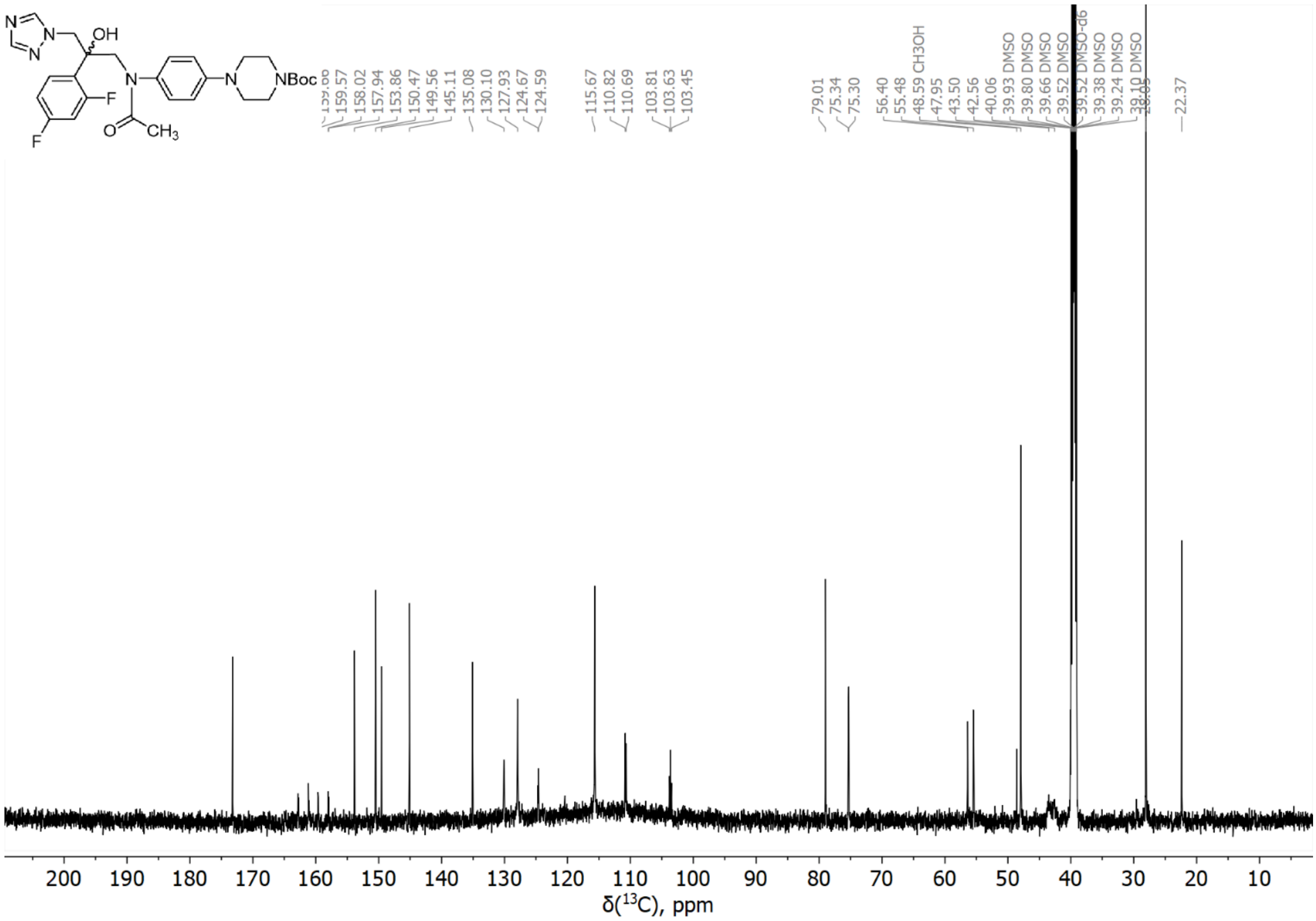

Supplementary Figure S70. ^{13}C NMR spectrum of **14f**

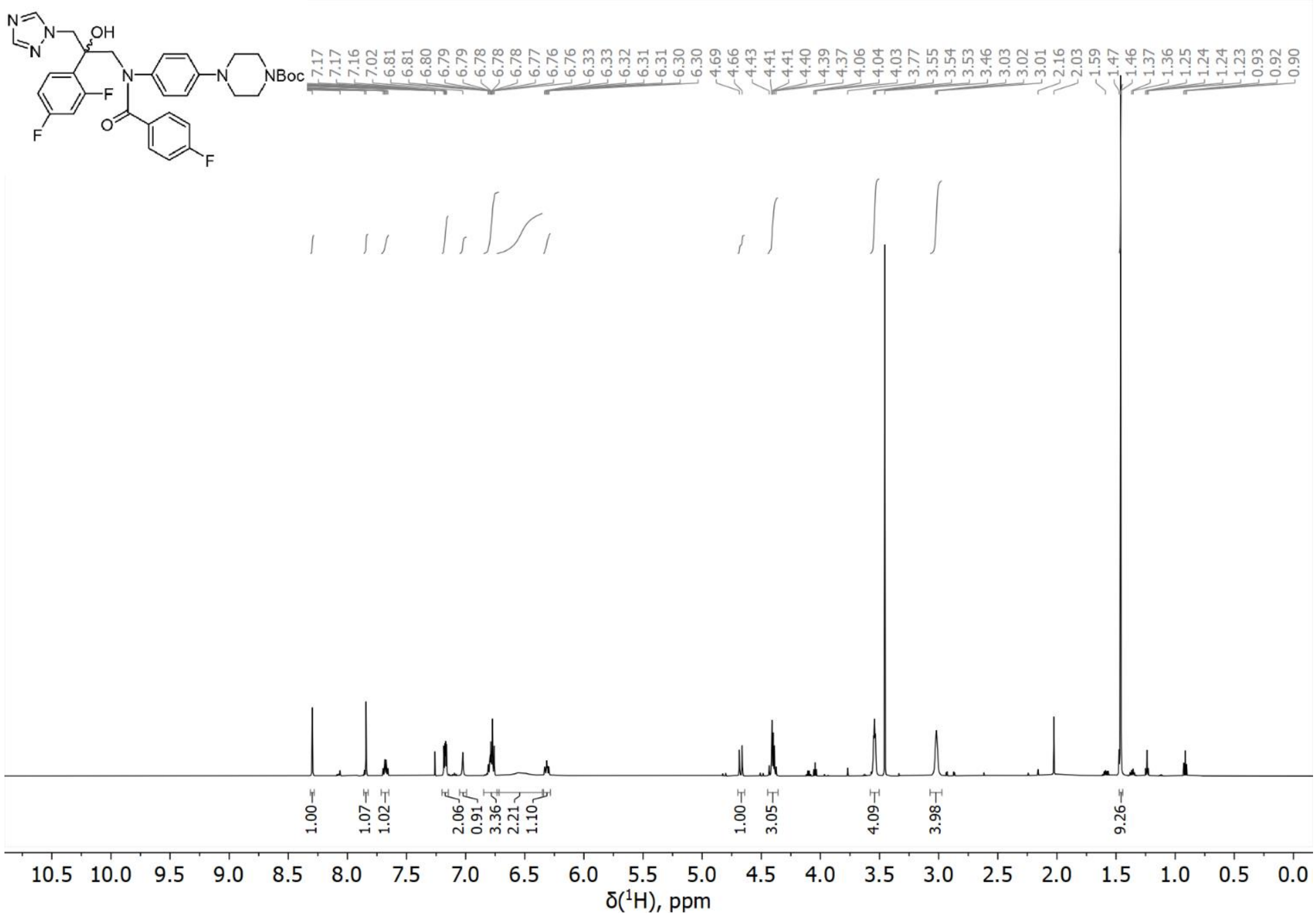

Supplementary Figure S71. ^{1}H NMR spectrum of **15f**

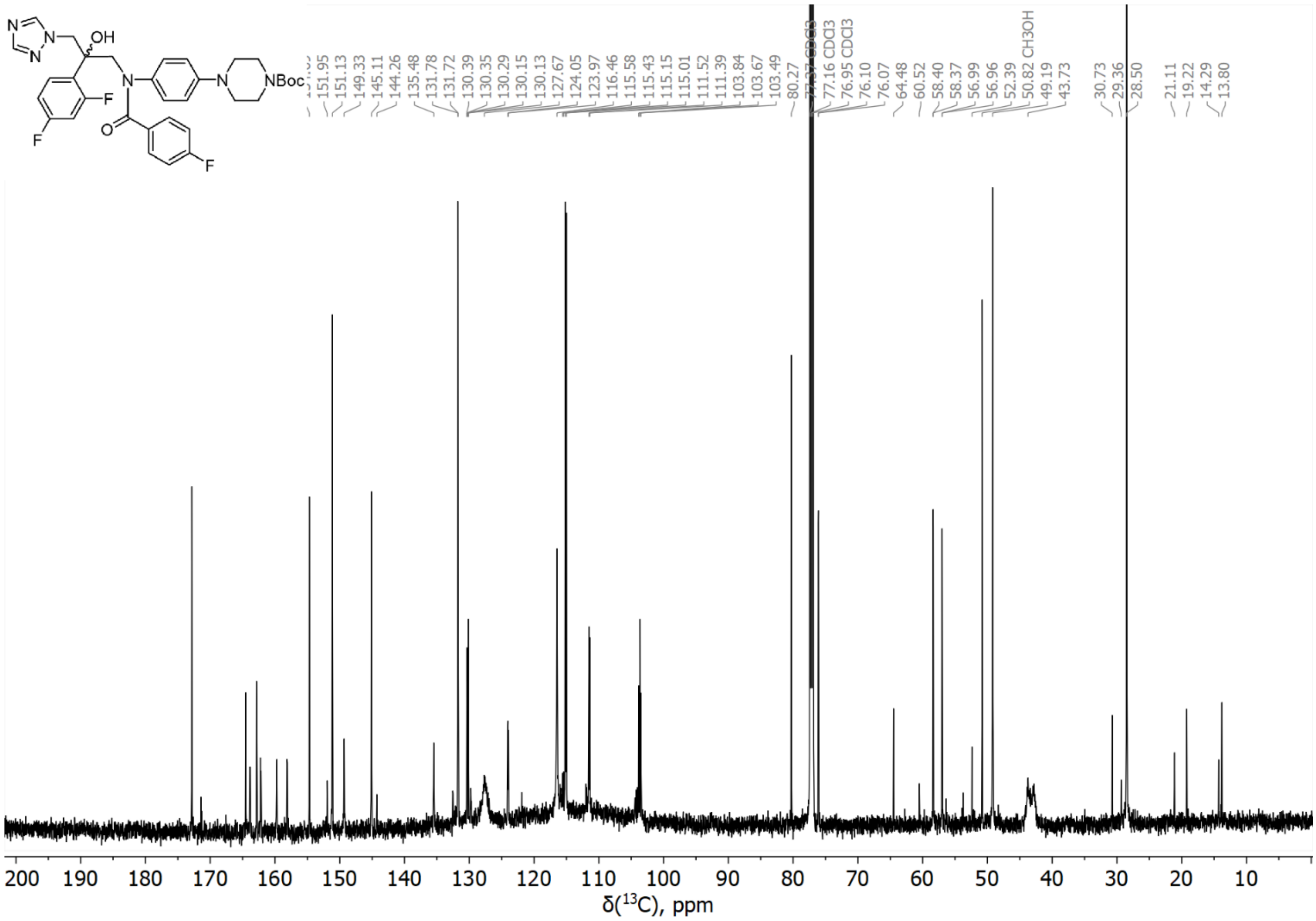

Supplementary Figure S72. ^{13}C NMR spectrum of **15f**

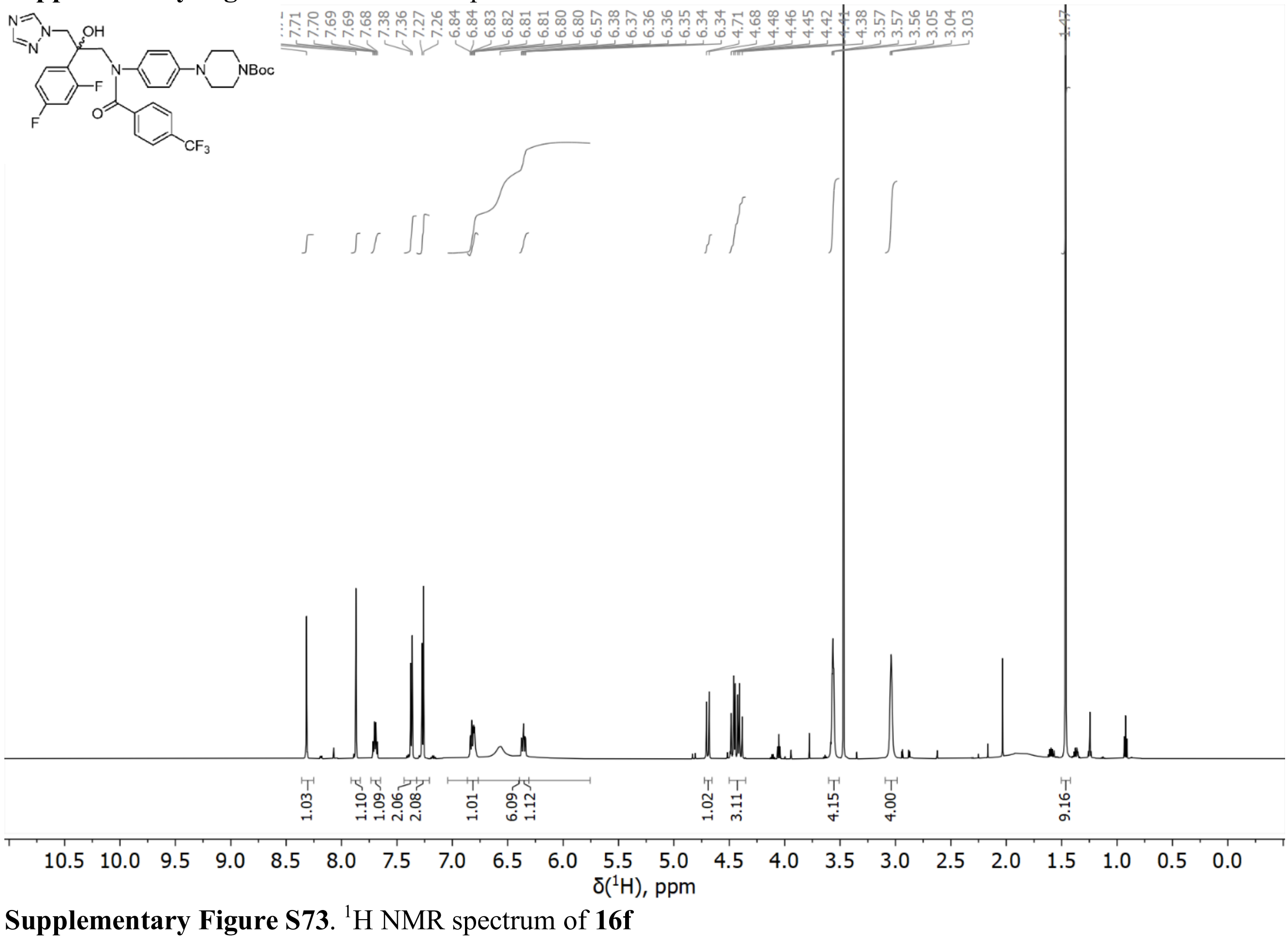

Supplementary Figure S73. ^{1}H NMR spectrum of **16f**

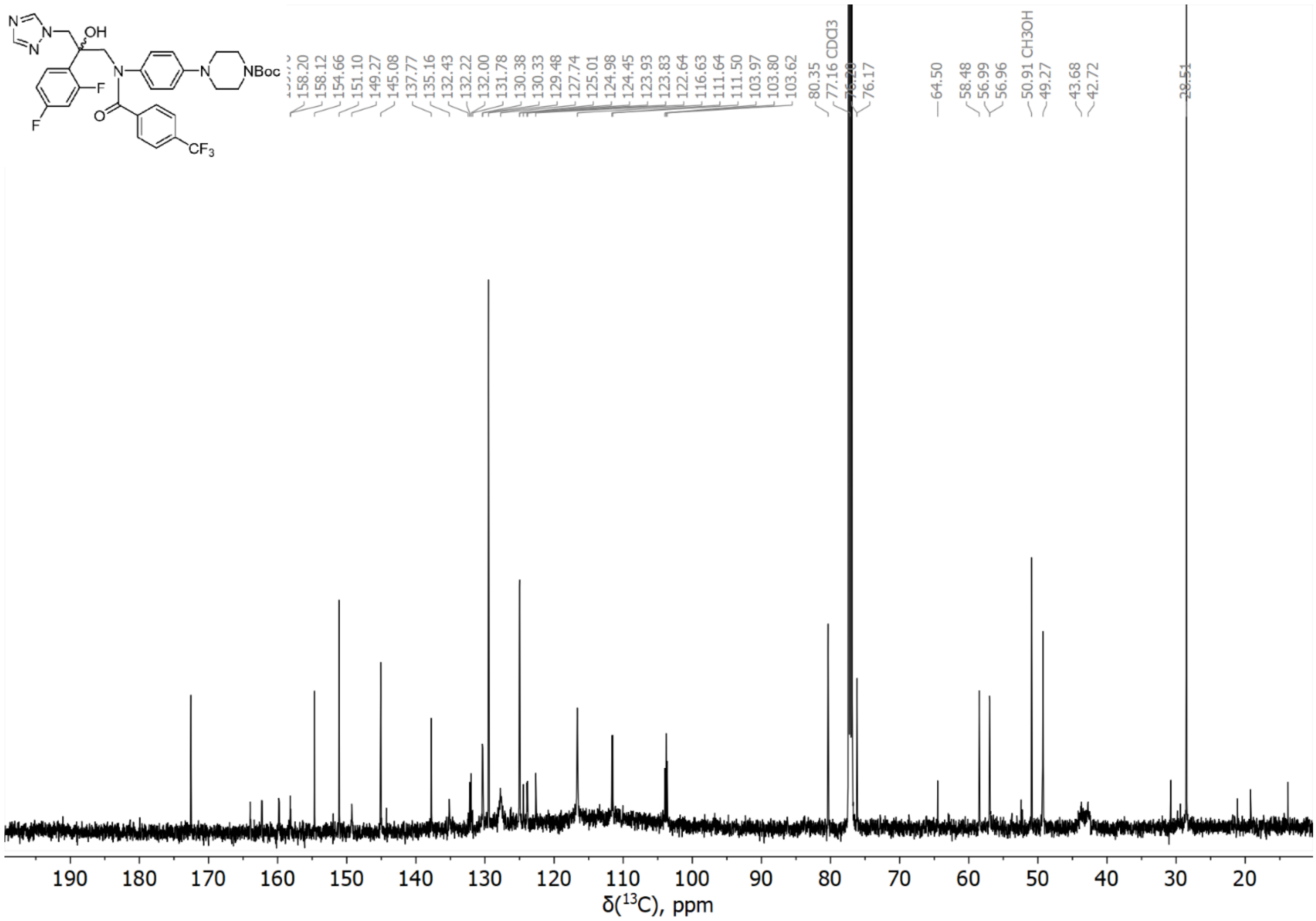

Supplementary Figure S74. ^{13}C NMR spectrum of **16f**

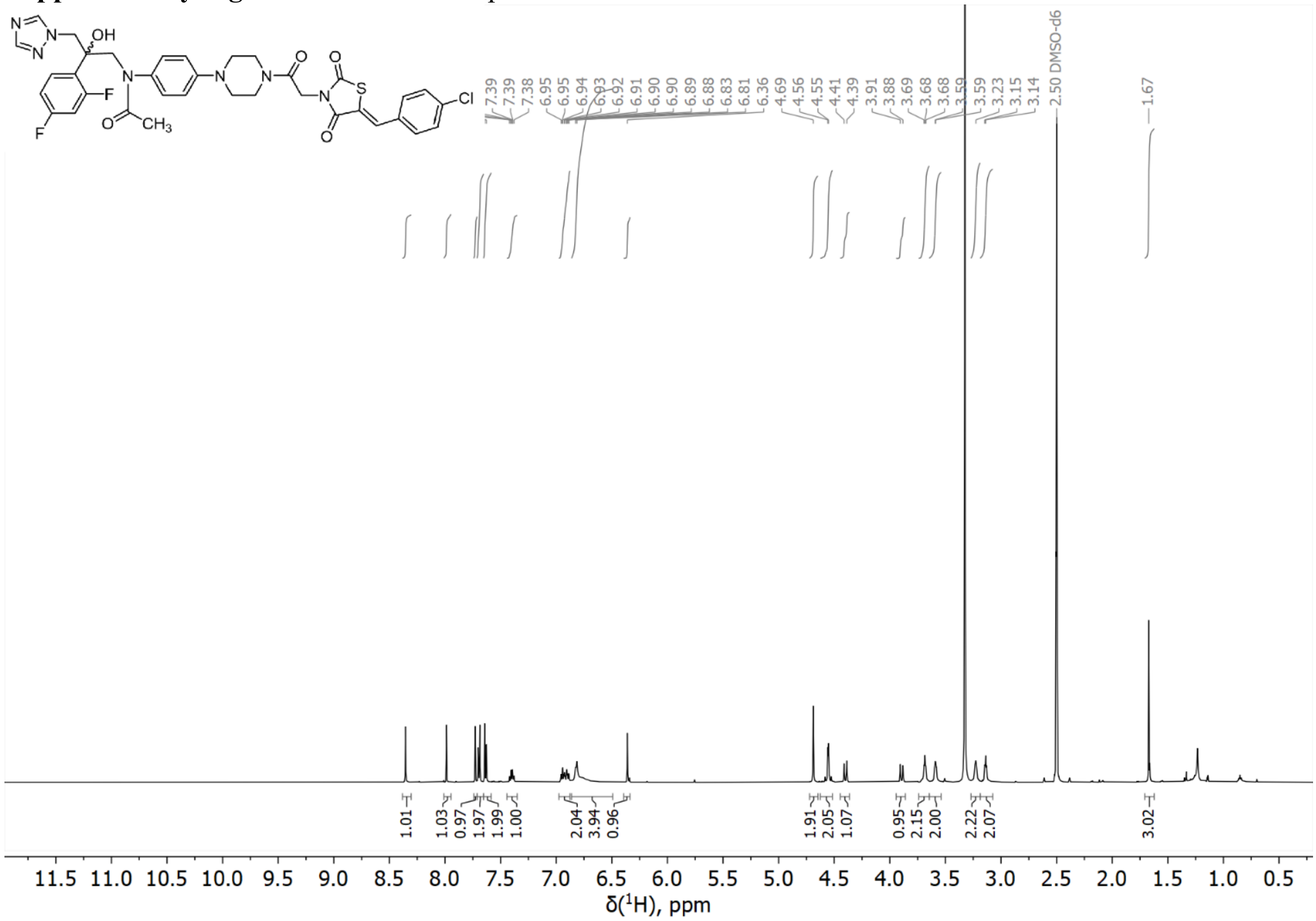

Supplementary Figure S75. ^{1}H NMR spectrum of **17f**

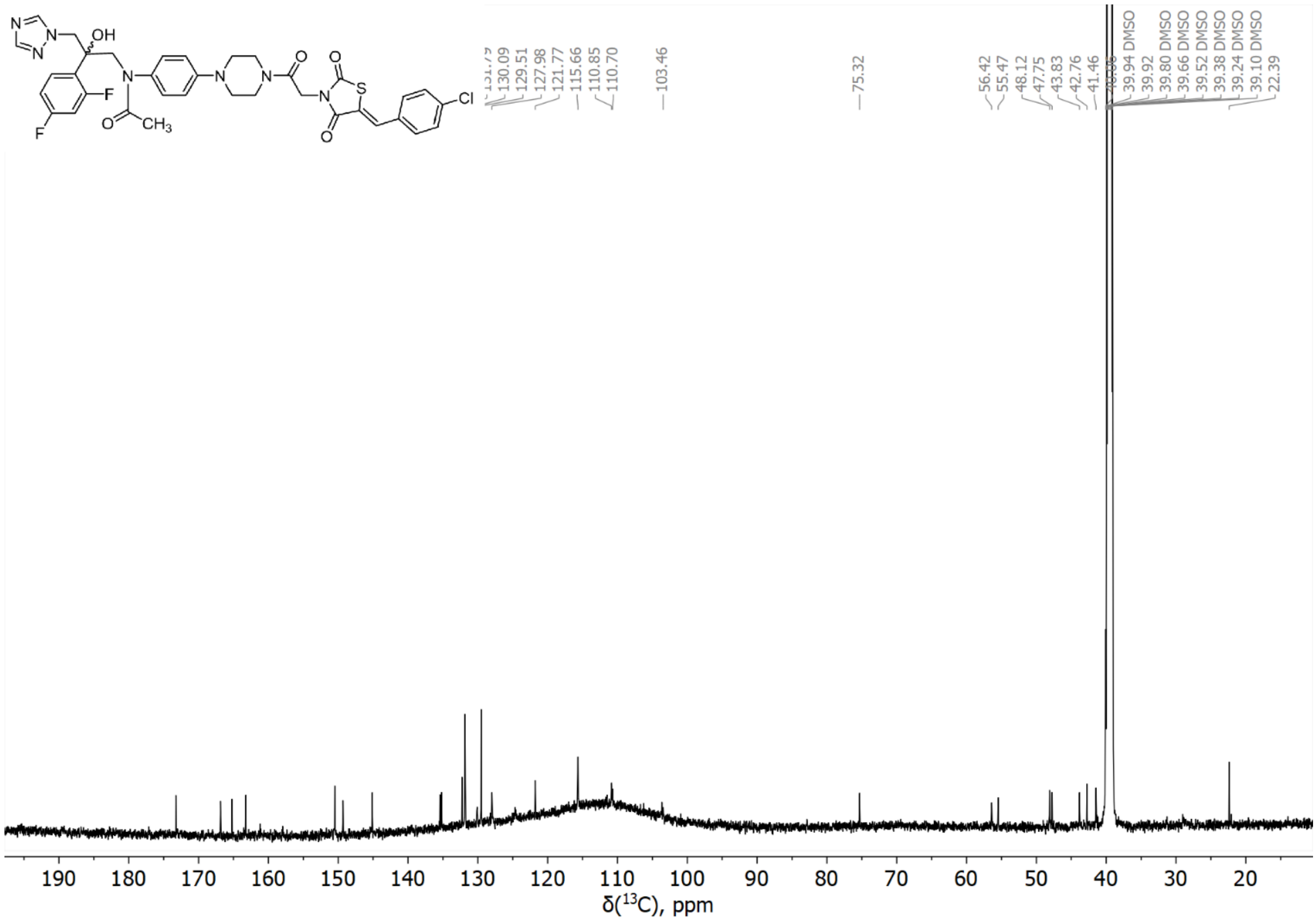

Supplementary Figure S76. ^{13}C NMR spectrum of **17f**

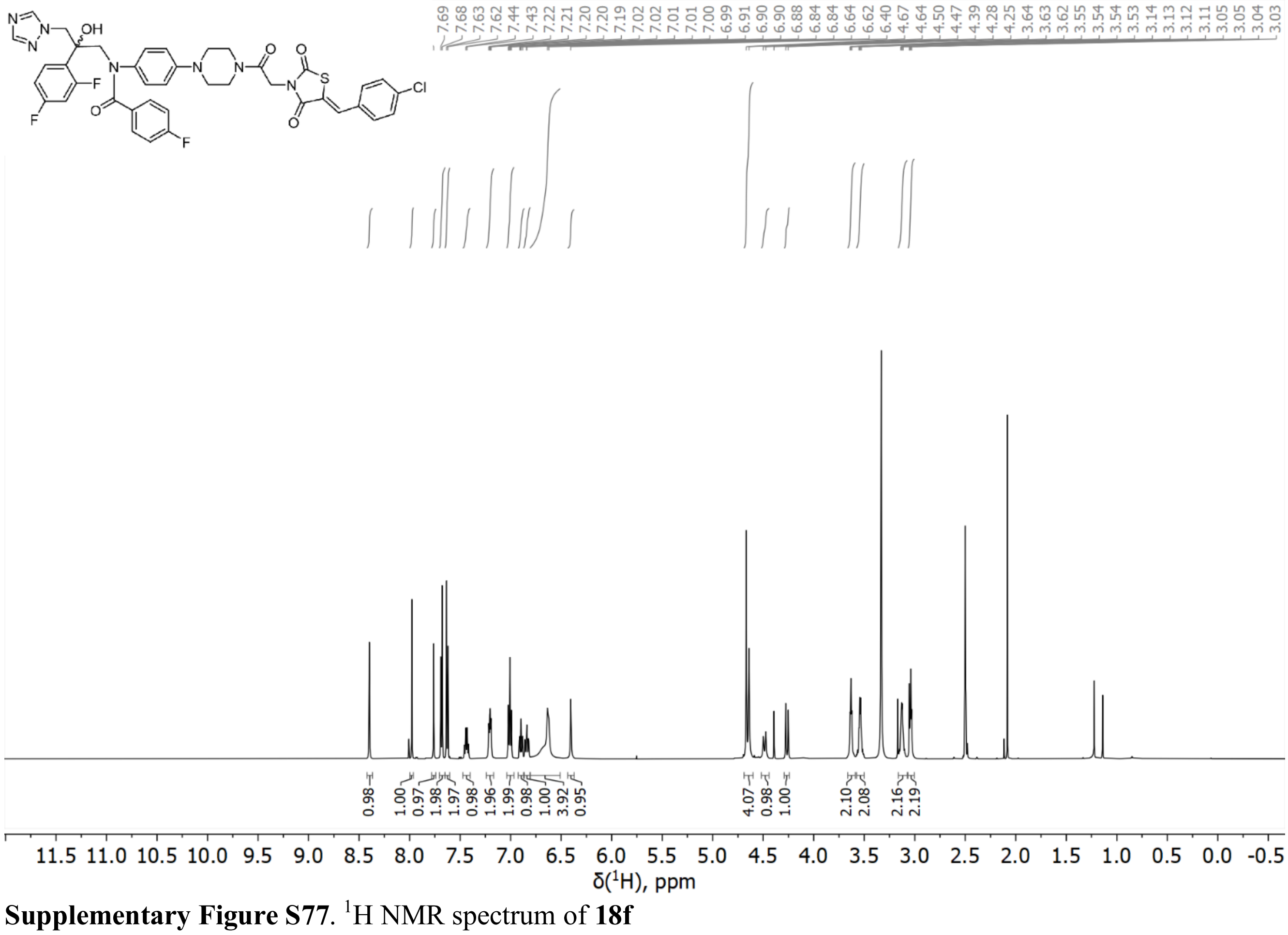

Supplementary Figure S77. ^{1}H NMR spectrum of **18f**

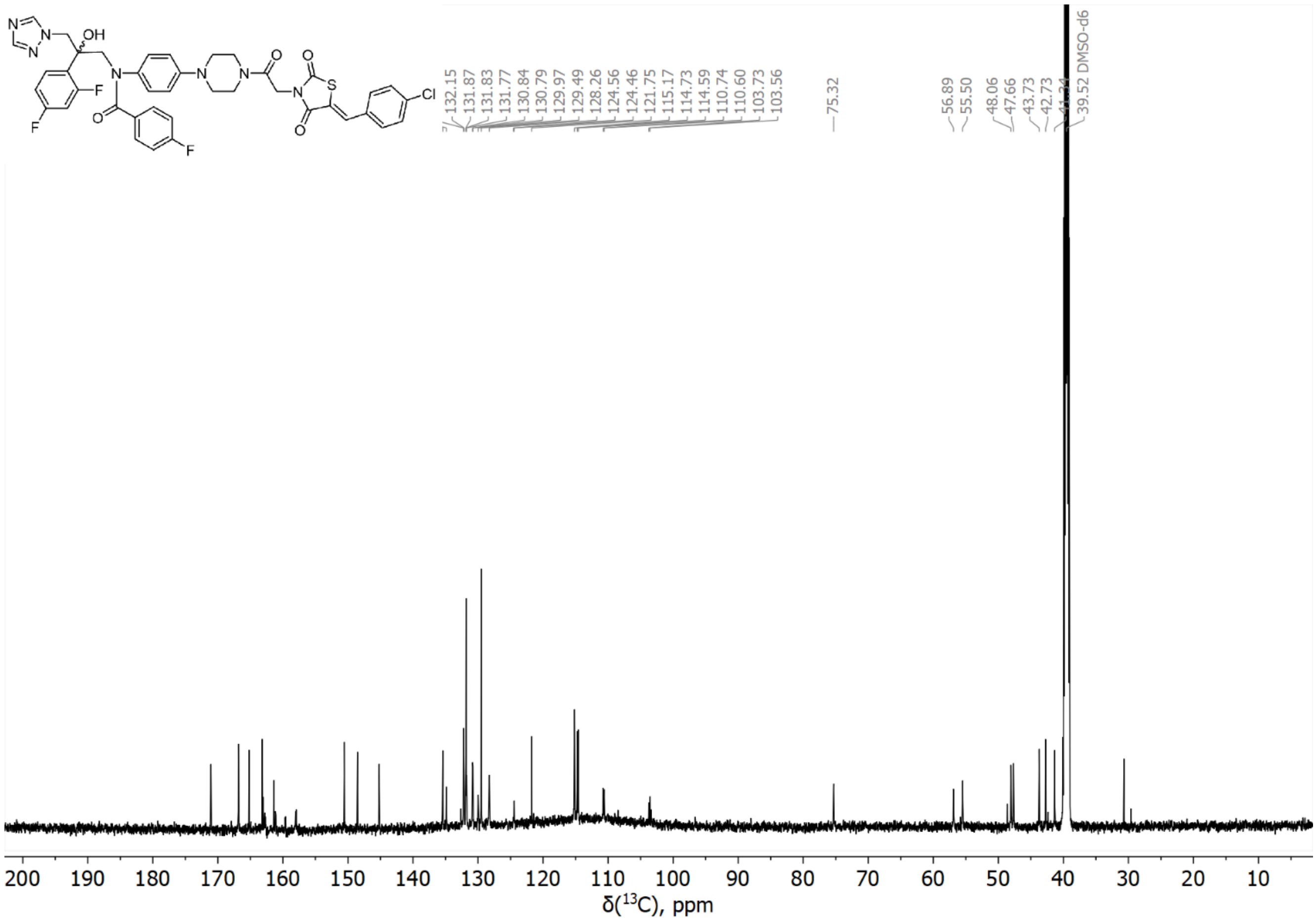

Supplementary Figure S78. ^{13}C NMR spectrum of **18f**

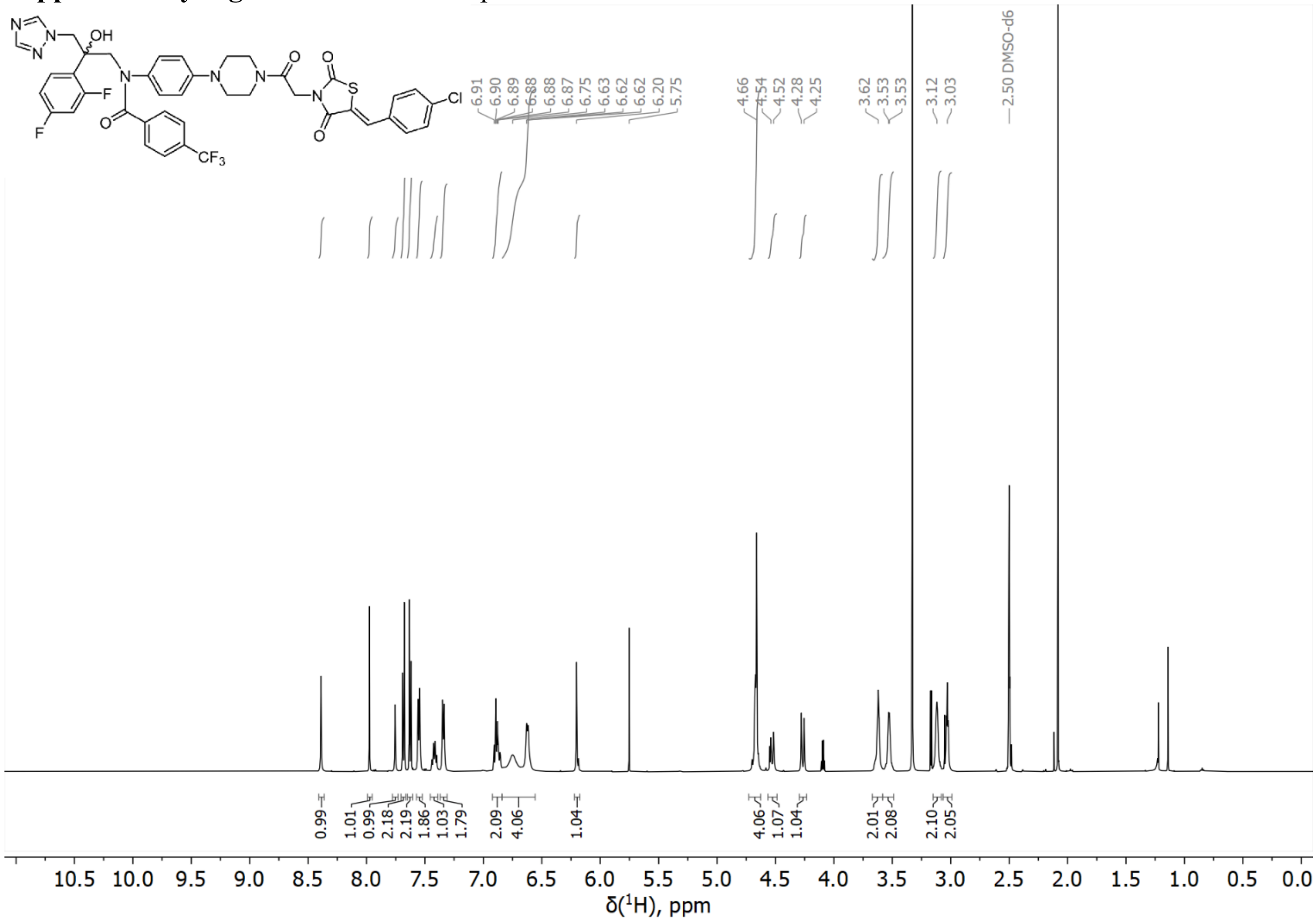

Supplementary Figure S79. ^{1}H NMR spectrum of **19f**

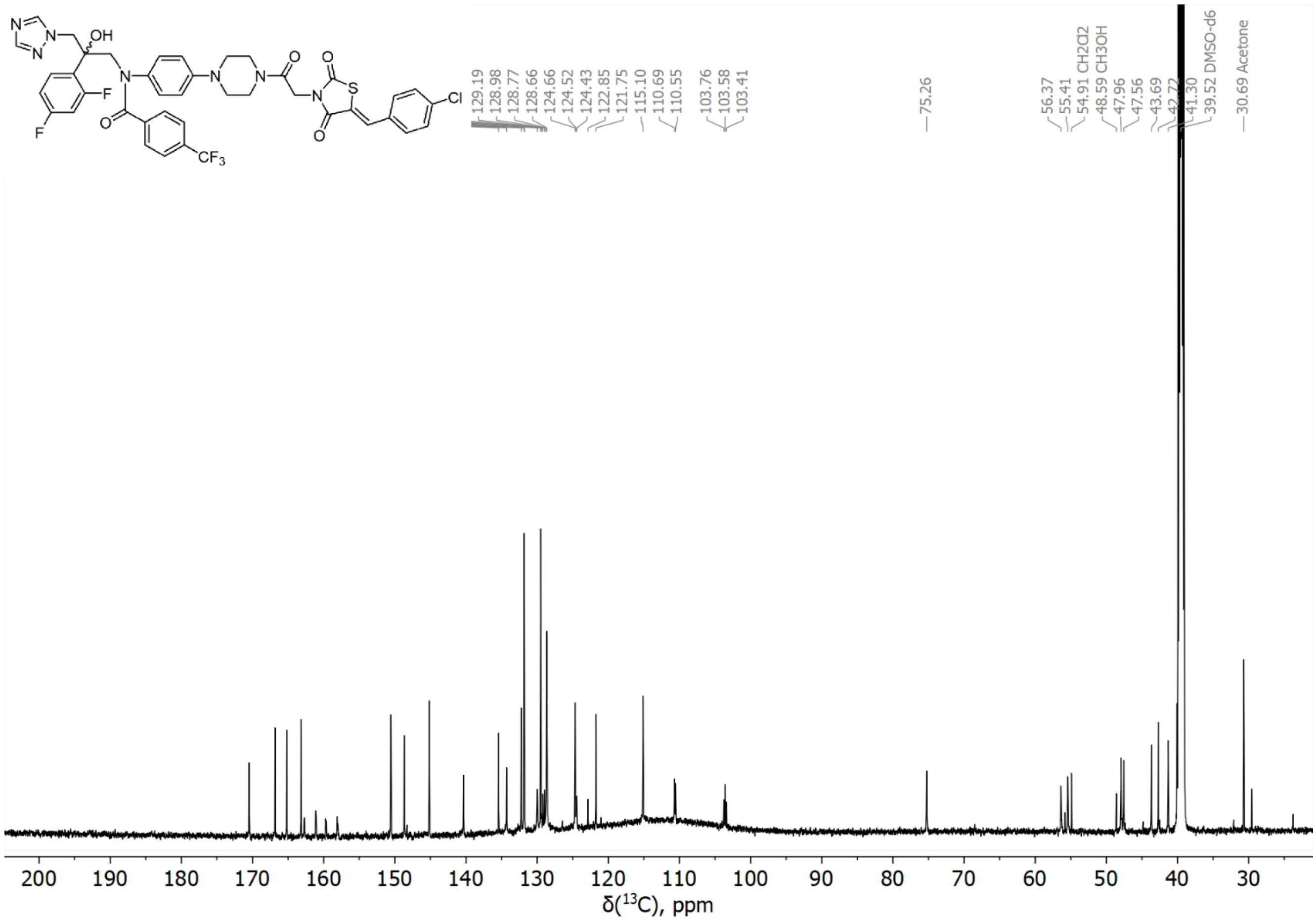

Supplementary Figure S80. ^{13}C NMR spectrum of **19f**

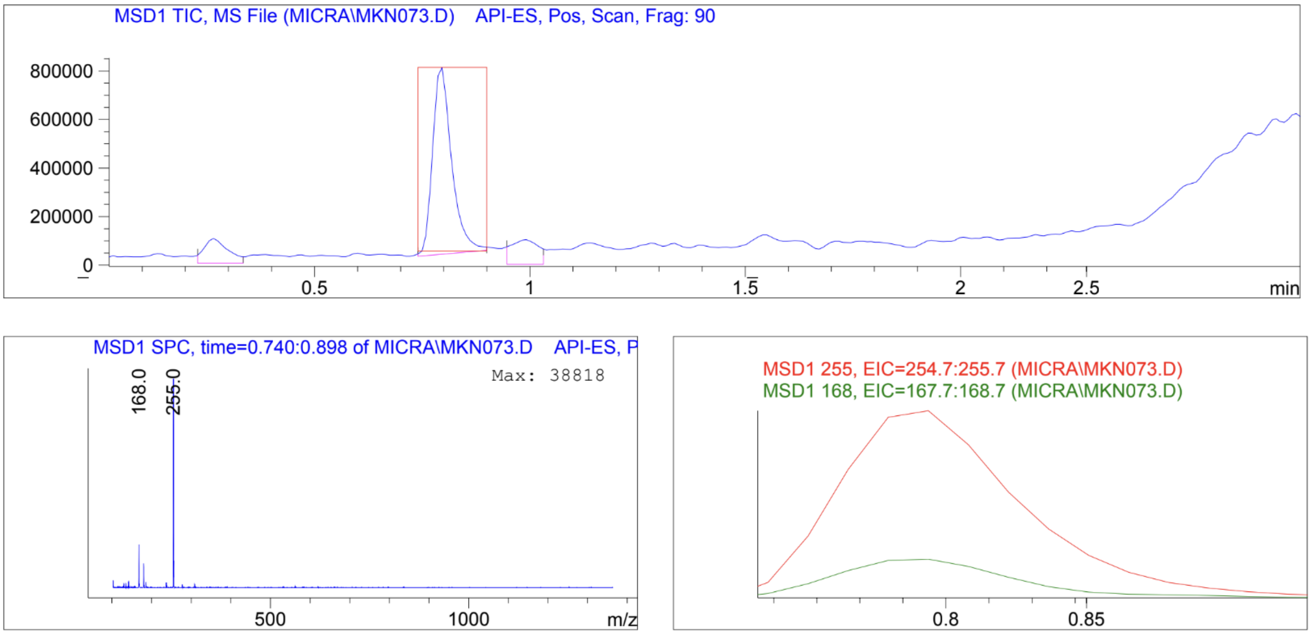

Figure S81. Mass-spectra for **Compound 2a** (positive ions). Found mass for $C_{11}H_{12}F_2N_4O$ = 255.0 $[M+H]^+$

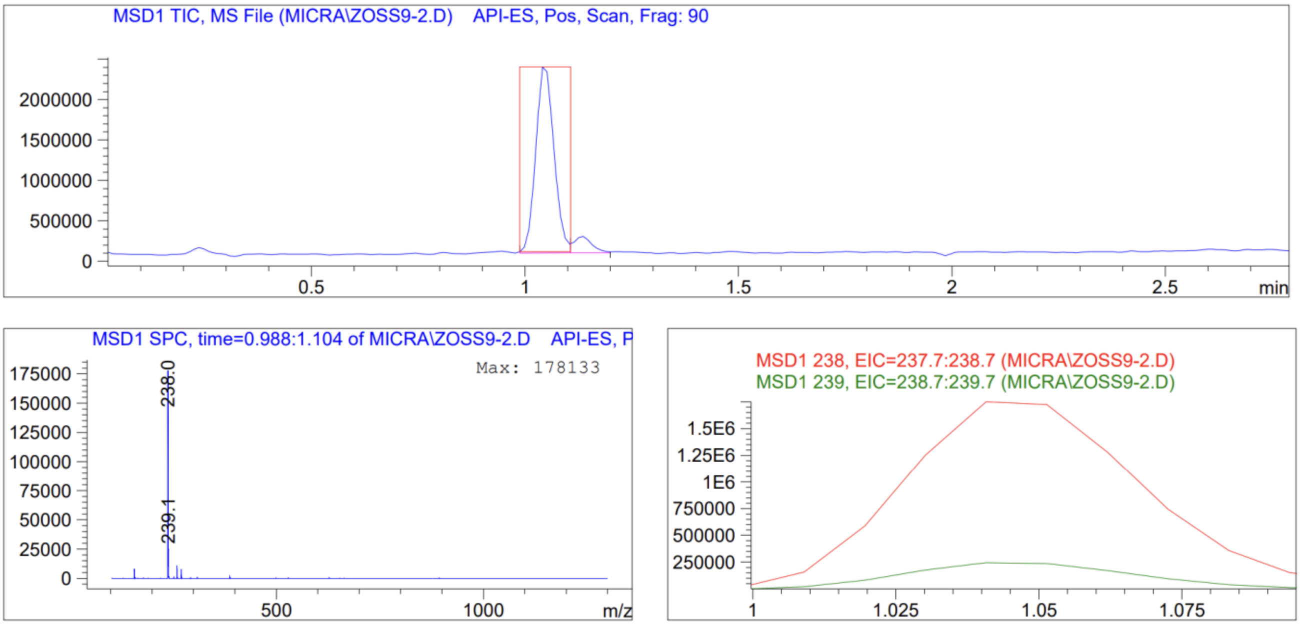

Figure S82. Mass-spectra for **Compound 3** (positive ions). Found mass for $C_{11}H_9F_2N_3O$ = 238.0 $[M+H]^+$

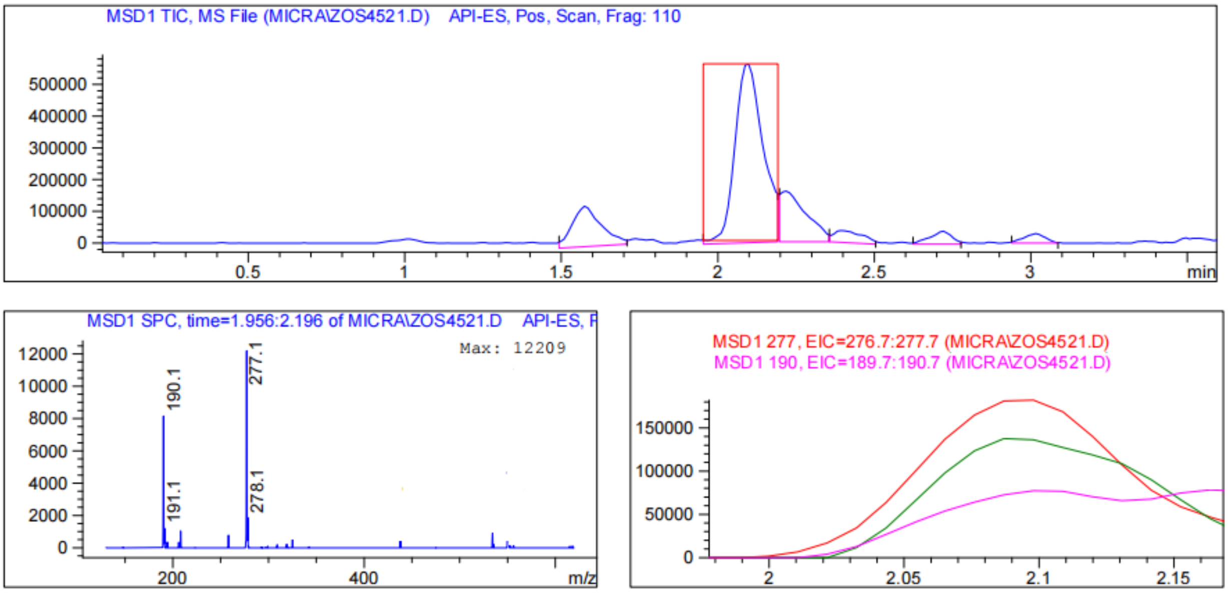

Figure S83. Mass-spectra for **Compound 5** (positive ions). Found mass for $C_{14}H_{17}FN_4O$ = 277.1 $[M+H]^+$

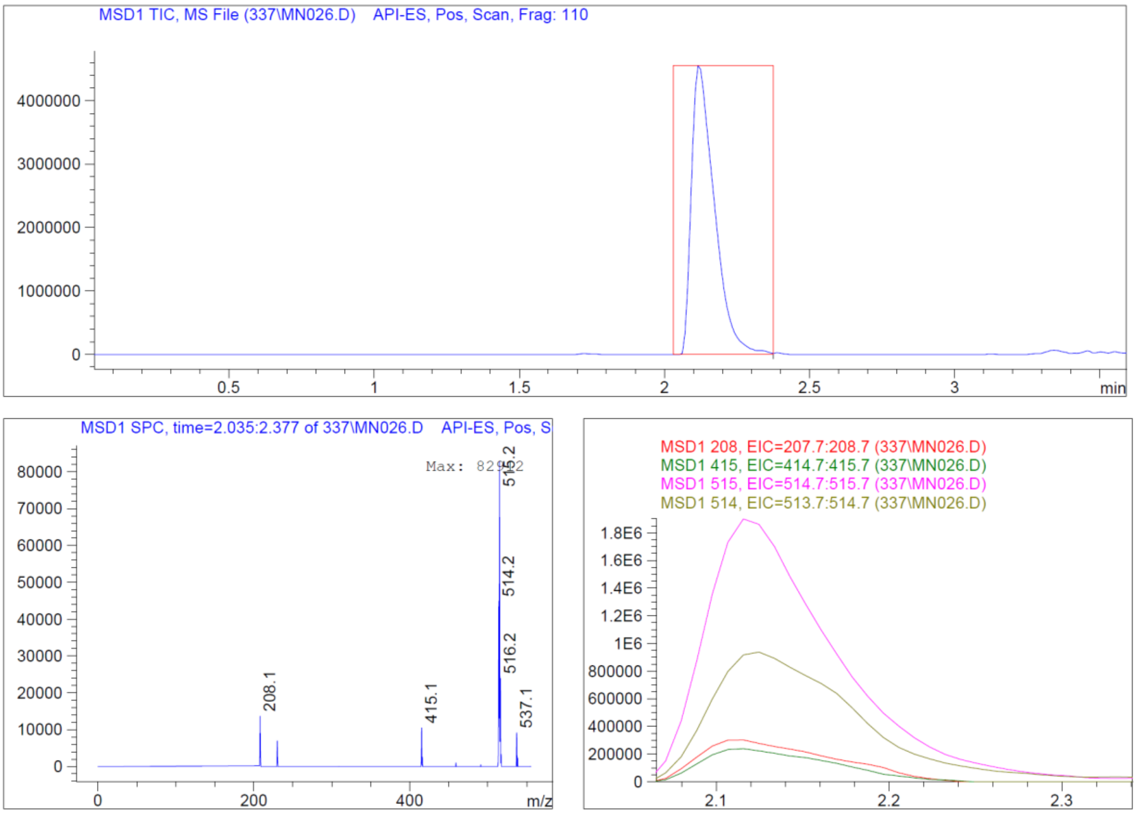

Figure S84. Mass-spectra for **Compound 2f** (positive ions). Found mass for $C_{26}H_{32}F_2N_6O_3$ = 515.2 $[M+H]^+$

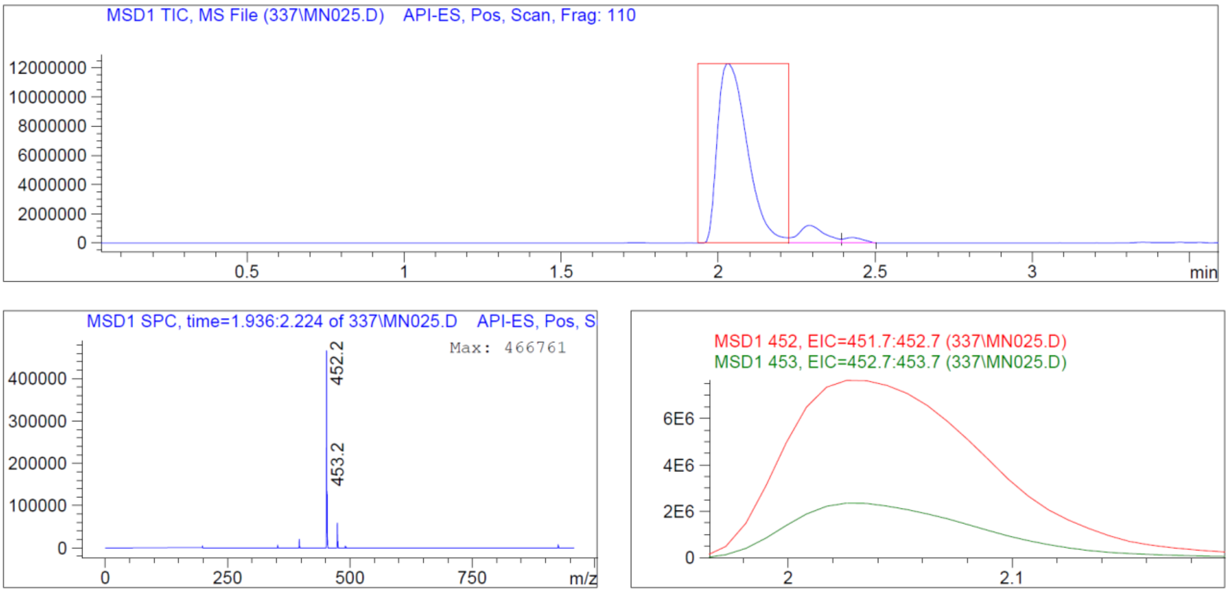

Figure S85. Mass-spectra for **Compound 2g** (positive ions). Found mass for $C_{22}H_{31}F_2N_5O_3$ = 452.2 $[M+H]^+$

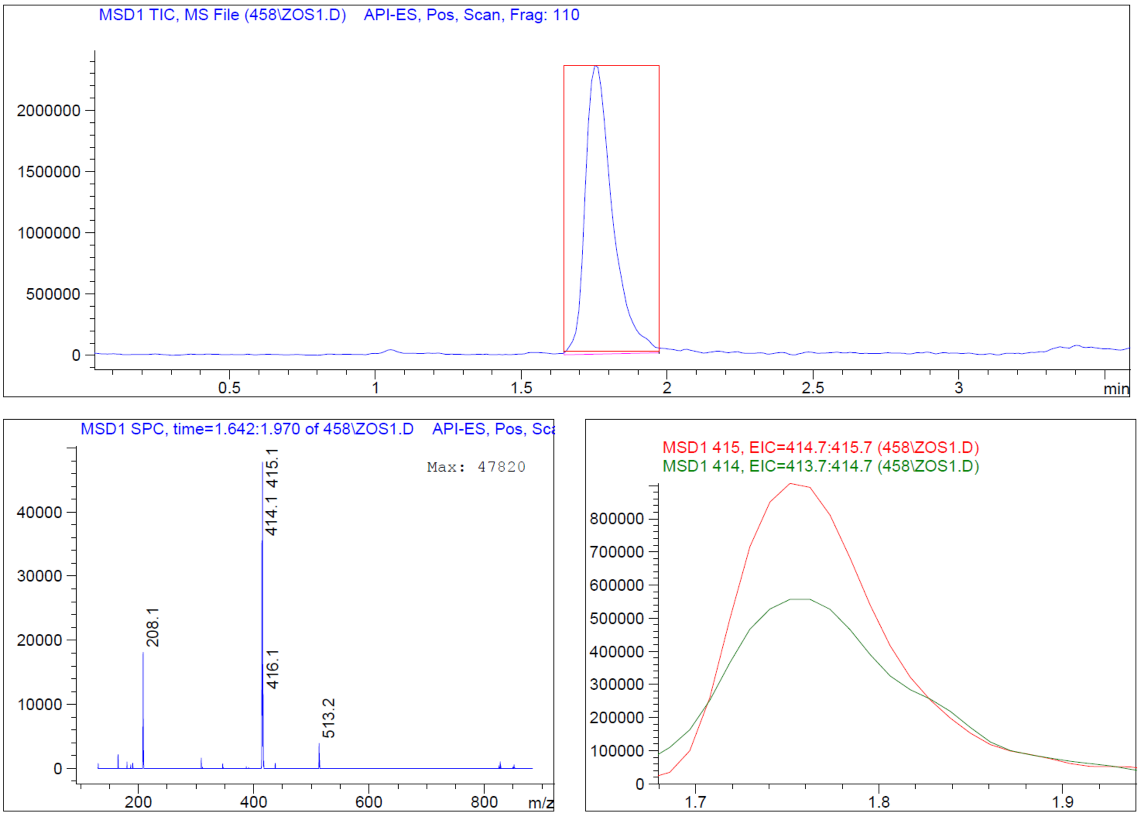

Figure S86. Mass-spectra for **Compound 4f** (positive ions). Found mass for $C_{21}H_{25}F_2N_6O$= 415.1 $[M]^+$

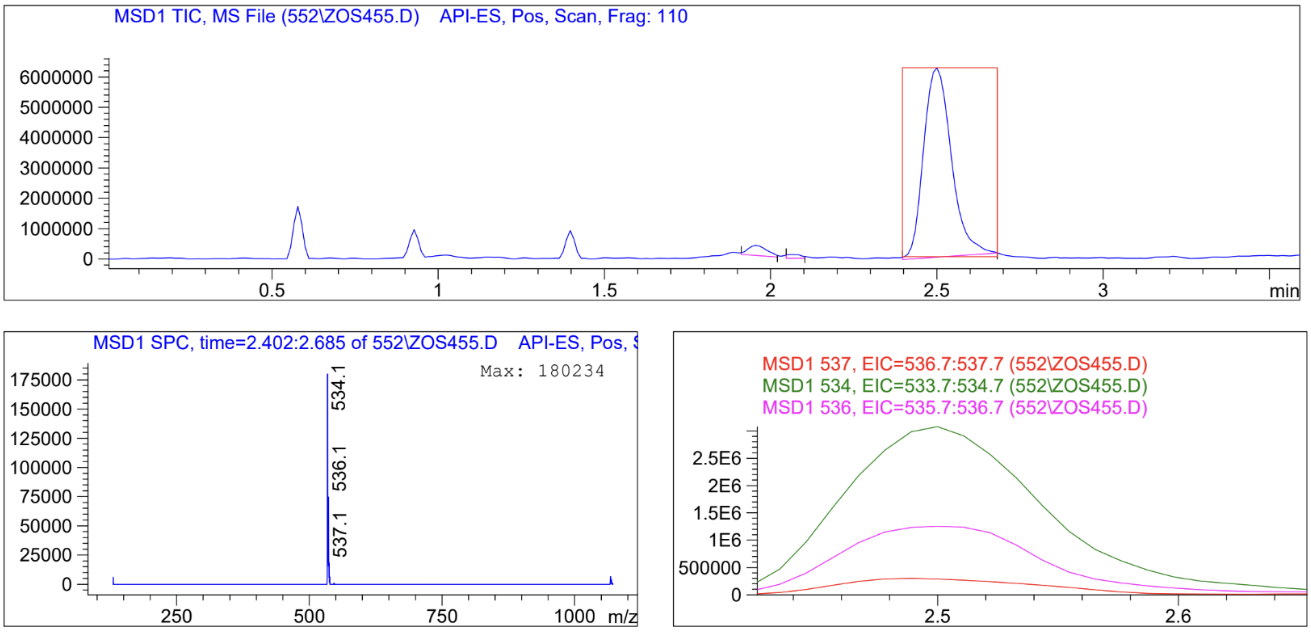

Figure S87. Mass-spectra for **Compound 6a** (positive ions). Found mass for $C_{23}H_{18}ClF_2N_5O_4S$= 534.1 $[M+1]^+$

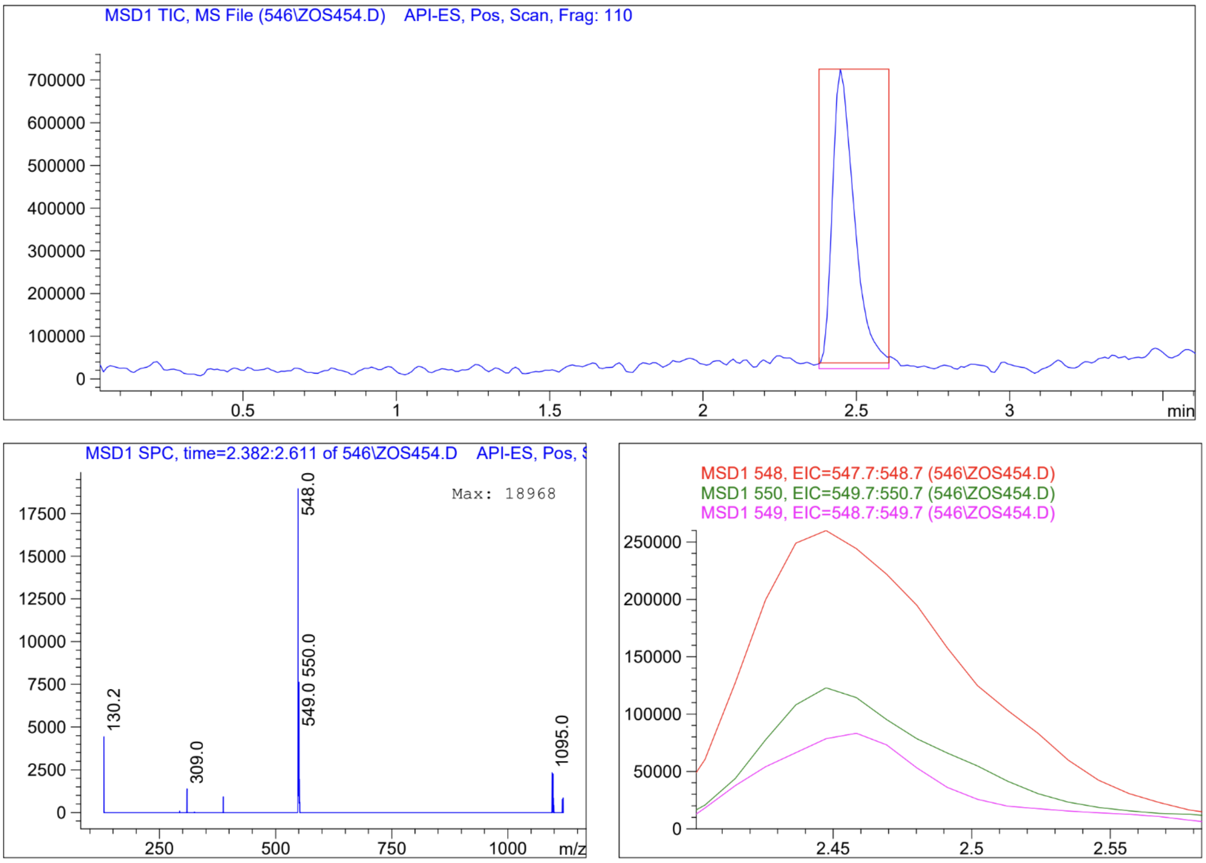

Figure S88. Mass-spectra for **Compound 6b** (positive ions). Found mass for $C_{24}H_{20}ClF_2N_5O_4S$= 548.0 $[M+1]^+$

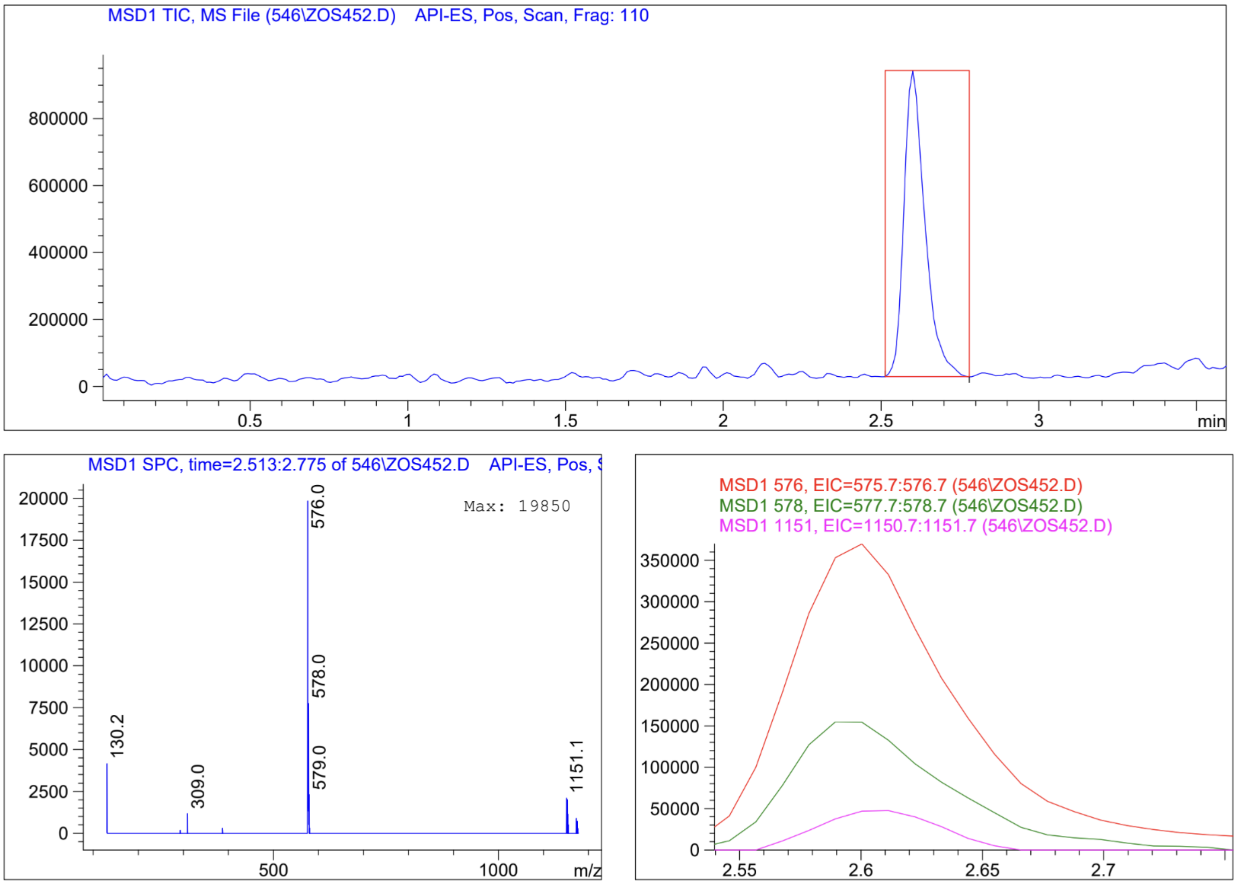

Figure S89. Mass-spectra for **Compound 6b** (positive ions). Found mass for $C_{26}H_{24}ClF_2N_5O_4S$= 576.0 $[M+1]^+$

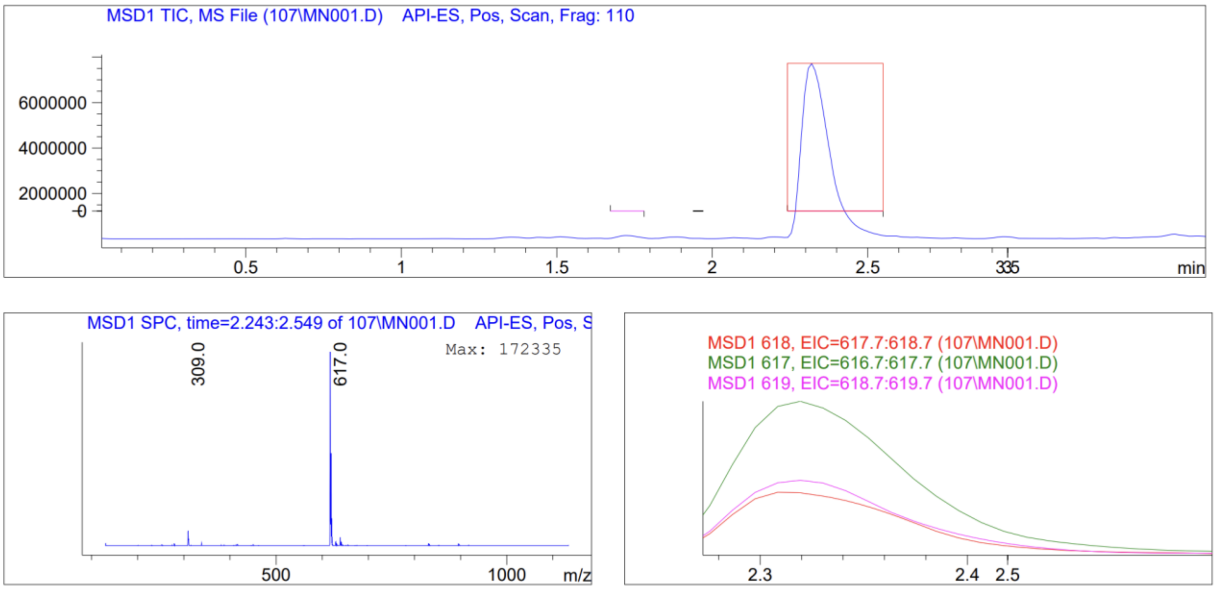

Figure S90. Mass-spectra for **Compound 7e** (positive ions). Found mass for $C_{28}H_{27}ClF_2N_6O_4S$ = 617.0 $[M+H]^+$

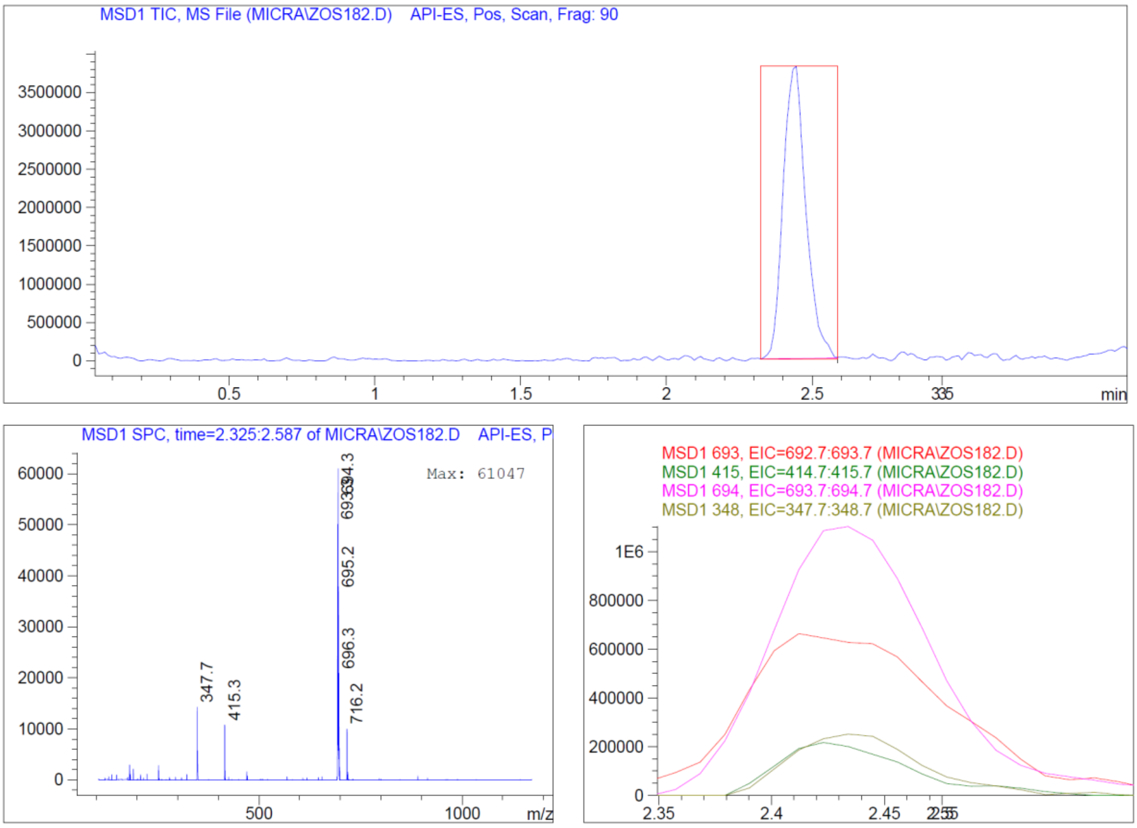

Figure S91. Mass-spectra for **Compound 8f** (positive ions). Found mass for $C_{33}H_{30}ClF_2N_7O_4S$= 693.3 $[M]^+$, 694.3 $[M+H]^+$

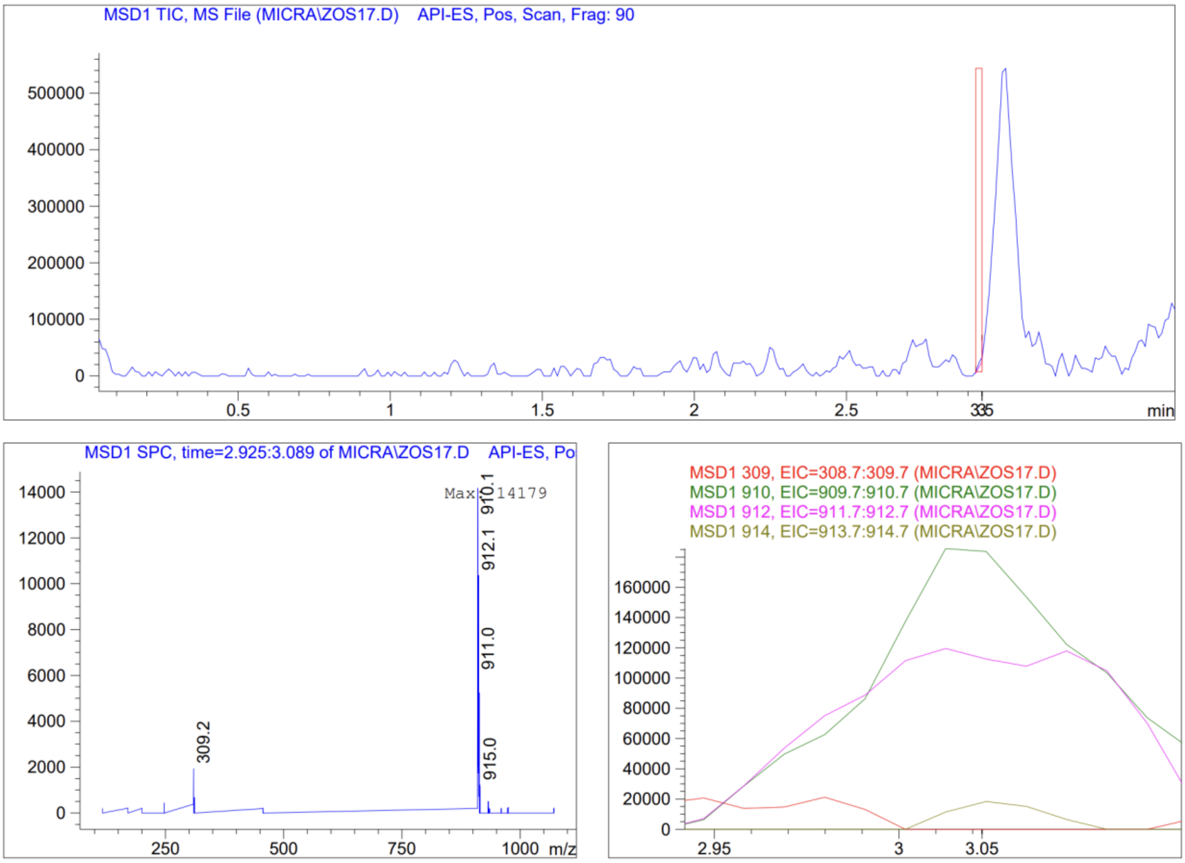

Figure S92. Mass-spectra for **Compound 10** (positive ions). Found mass for $C_{41}H_{35}Cl_2F_2N_7O_7S_2$= 910.1 $[M+1]^+$

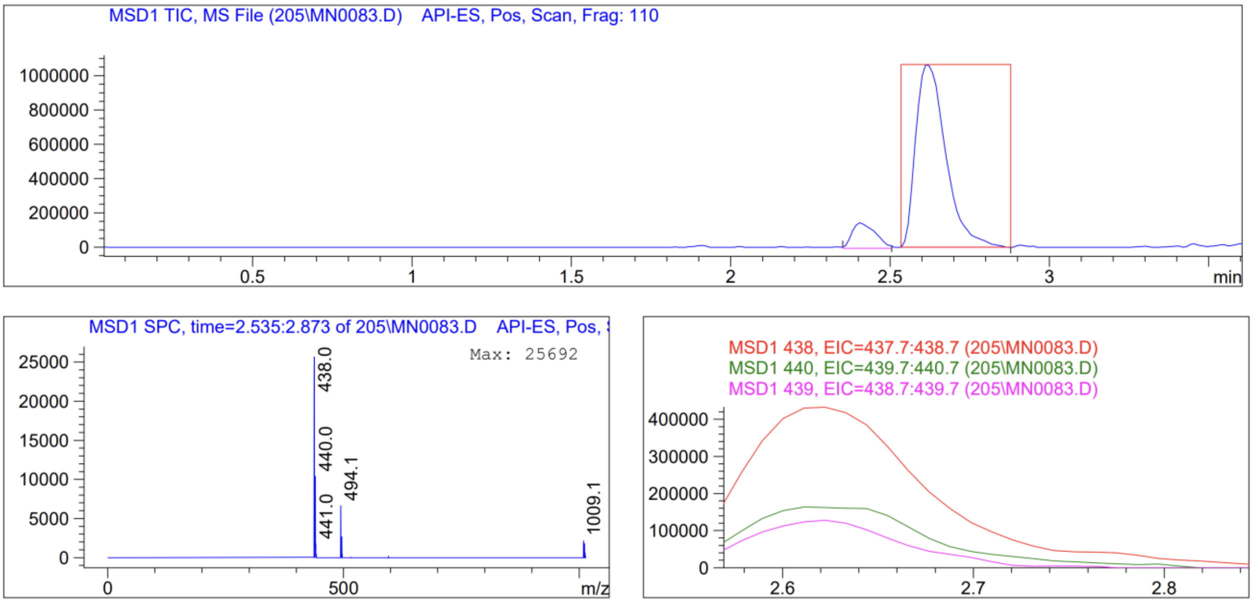

Figure S93. Mass-spectra for **Compound 11** (positive ions). Found mass for $C_{23}H_{28}ClN_3O_5S$= 494.1 $[M+1]^+$

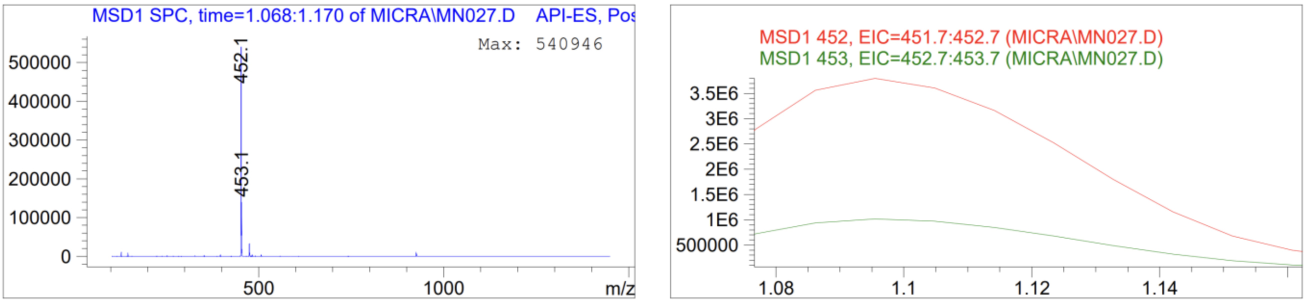

Figure S94. Mass-spectra for **Compound 12e** (positive ions). Found mass for $C_{22}H_{31}ClF_2N_5O_3$= 452.1 $[M+1]^+$

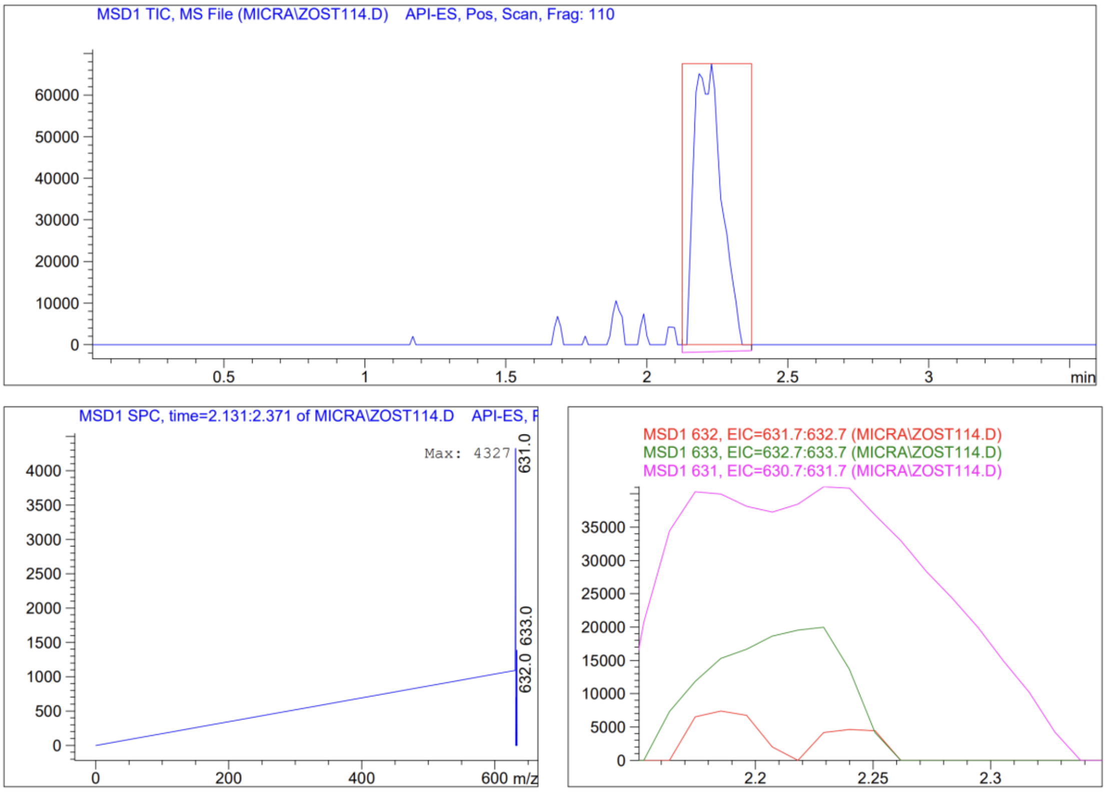

Figure S95. Mass-spectra for **Compound 12f** (positive ions). Found mass for $C_{29}H_{29}ClF_2N_6O_4S$= 631.0 $[M+1]^+$

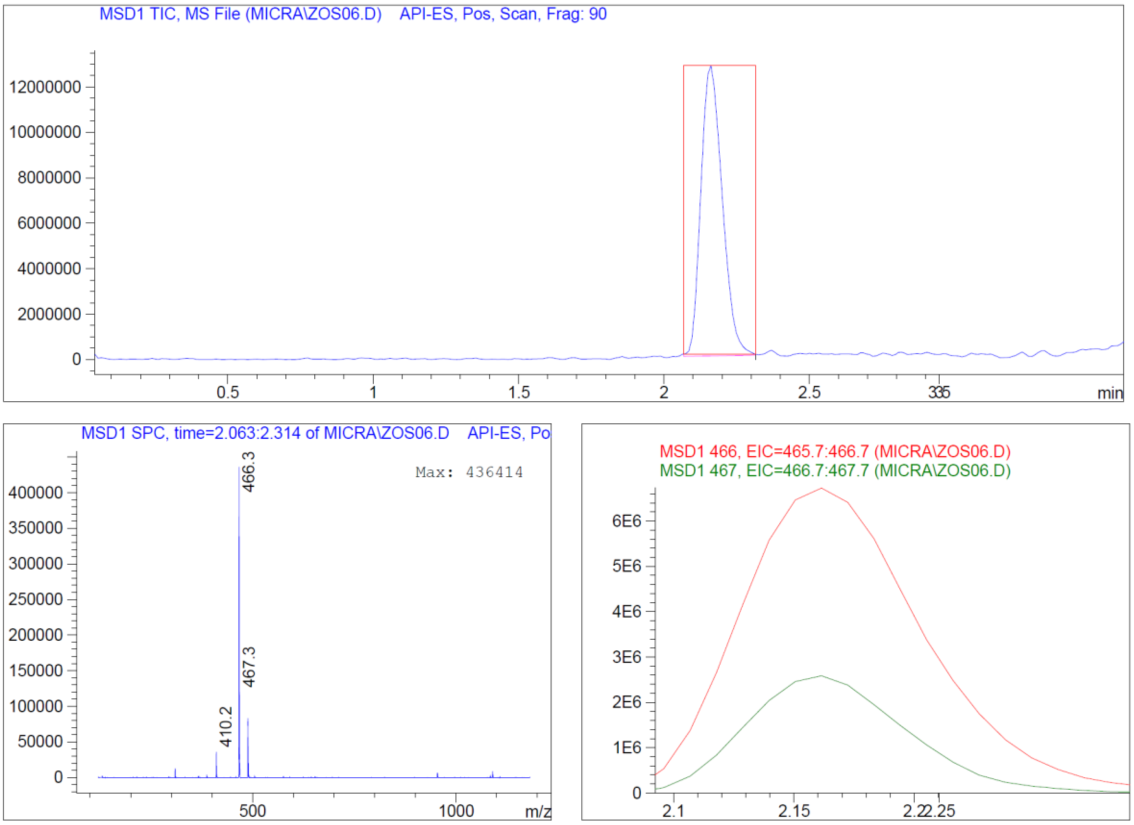

Figure S96. Mass-spectra for **Compound 12g** (positive ions). Found mass for $C_{23}H_{33}F_2N_5O_3$ = 466.3 $[M+H]^+$

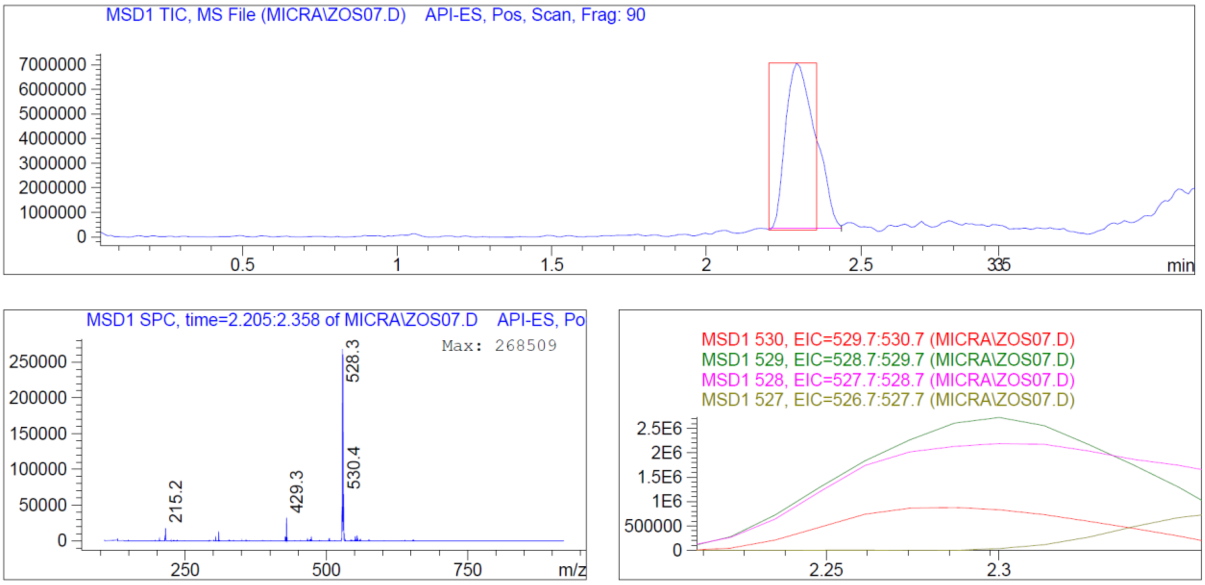

Figure S97. Mass-spectra for **Compound 12f** (positive ions). Found mass for $C_{27}H_{34}F_2N_6O_3$ = 528.3 $[M]^+$

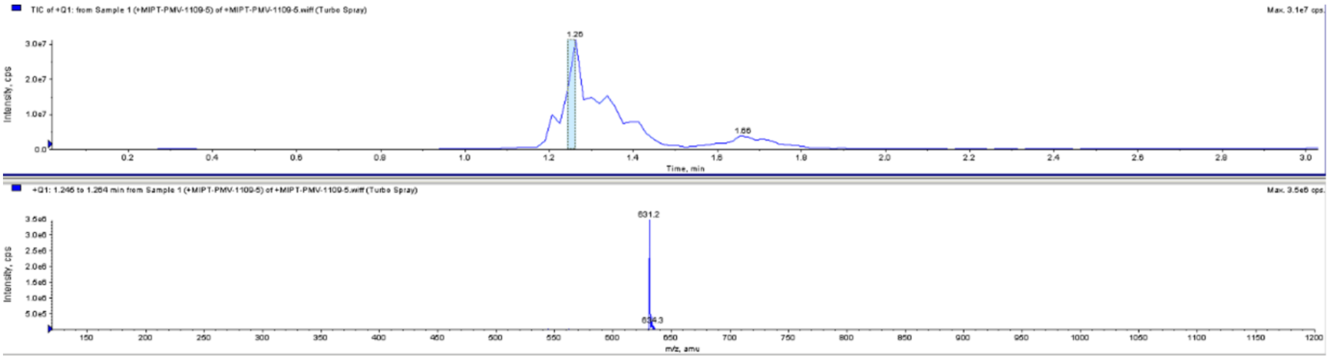

Figure S98. Mass-spectra for **Compound 13e** (positive ions). Found mass for $C_{29}H_{29}ClF_2N_6O_4S$ = 631.2 $[M+H]^+$

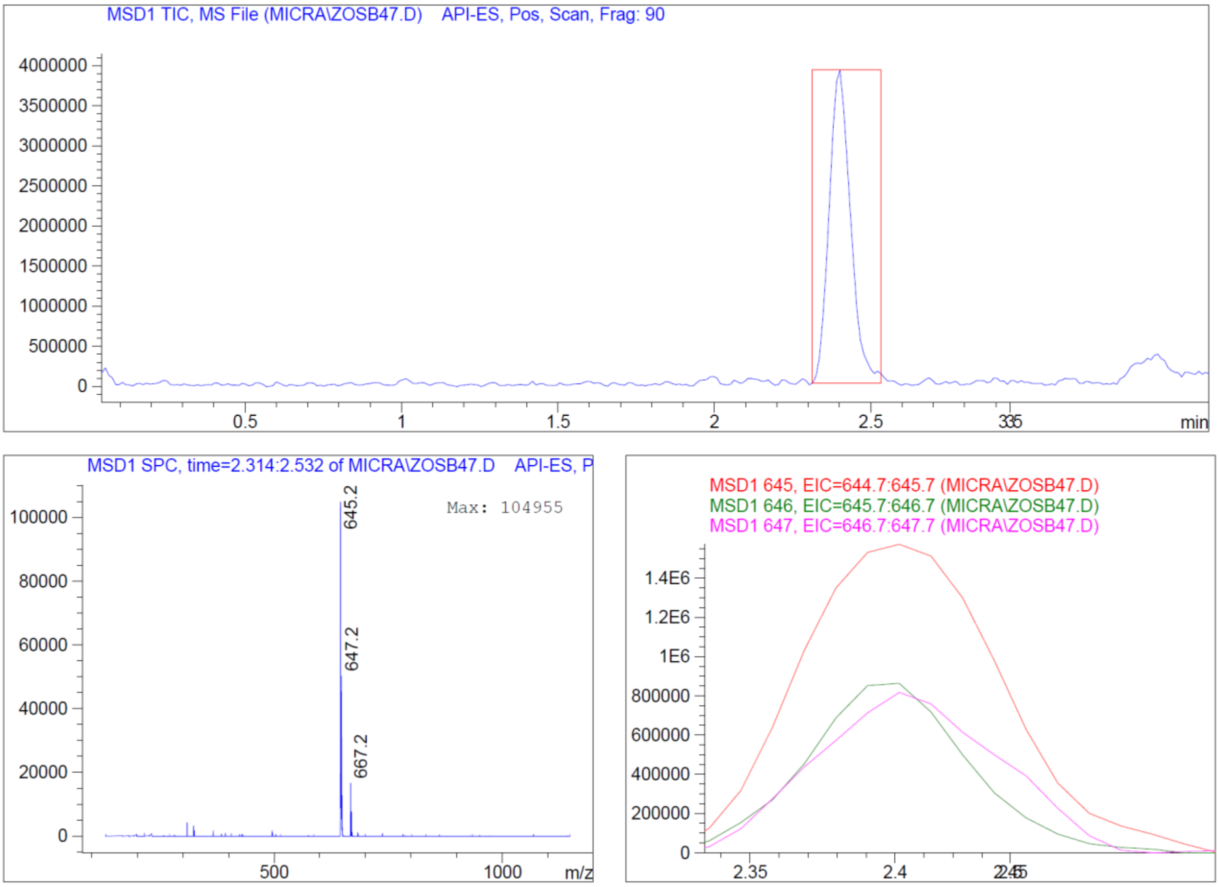

Figure S99. Mass-spectra for **Compound 13f** (positive ions). Found mass for $C_{30}H_{31}ClF_2N_6O_4S$ = 645.2 $[M+H]^+$

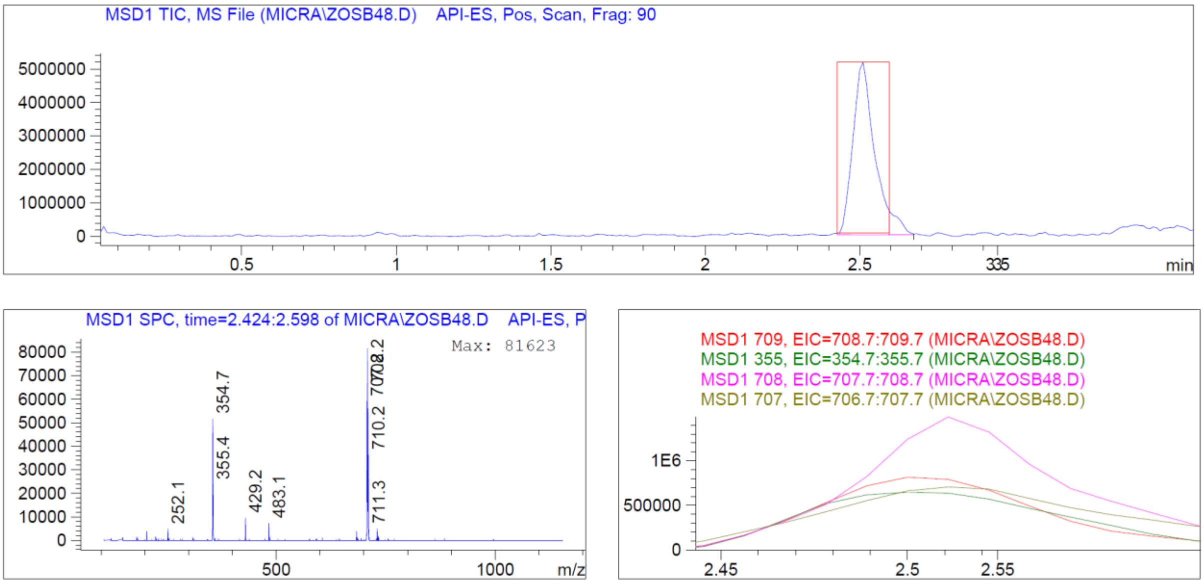

Figure S100. Mass-spectra for **Compound 13g** (positive ions). Found mass for $C_{34}H_{32}ClF_2N_7O_4S$ = 708.2 $[M+H]^+$

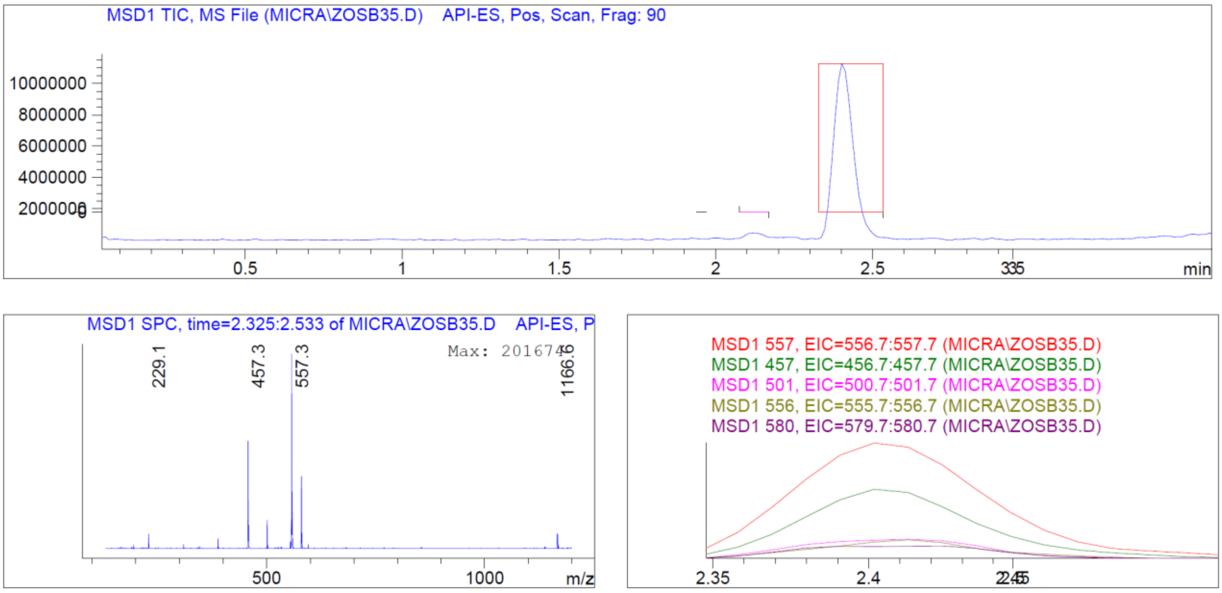

Figure S101. Mass-spectra for **Compound 14f** (positive ions). Found mass for $C_{28}H_{34}F_2N_6O_4$ = 557.3 $[M+H]^+$

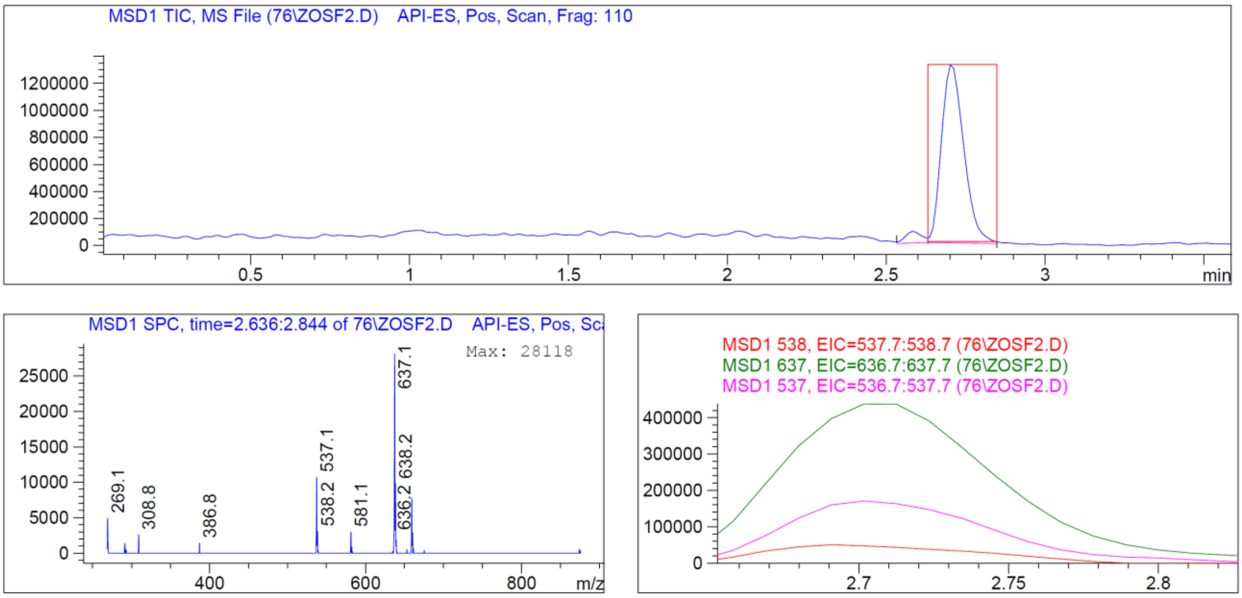

Figure S102. Mass-spectra for **Compound 15f** (positive ions). Found mass for $C_{33}H_{35}F_3N_6O_4$ = 637.1 $[M+H]^+$

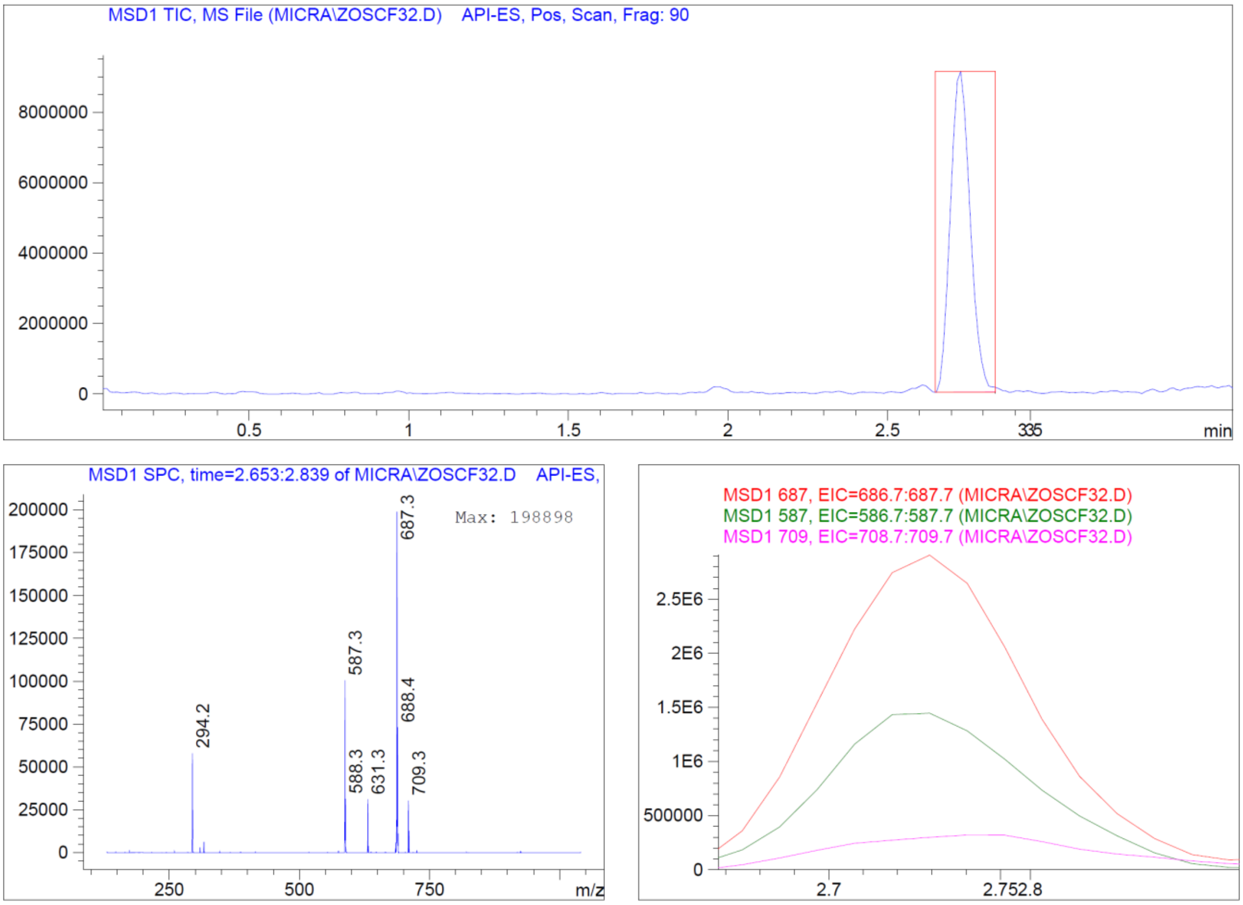

Figure S103. Mass-spectra for **Compound 16f** (positive ions). Found mass for $C_{34}H_{35}F_5N_6O_4$ = 687.3 $[M+H]^+$

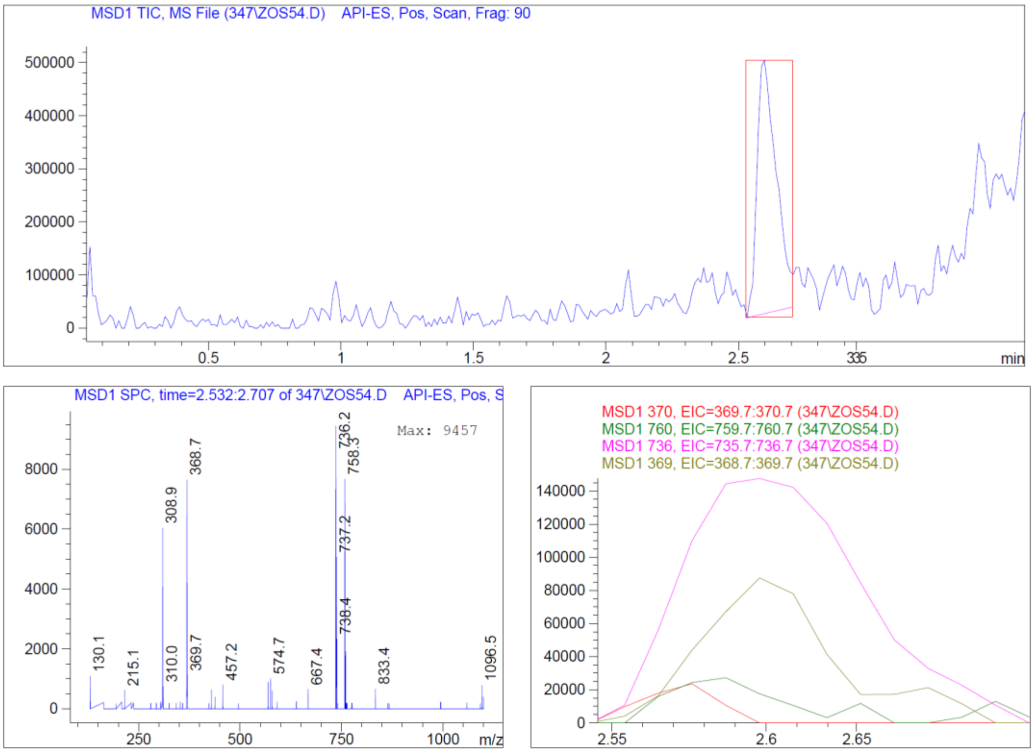

Figure S104. Mass-spectra for **Compound 17f** (positive ions). Found mass for $C_{35}H_{32}ClF_2N_7O_5S$ = 736.2 $[M+H]^+$

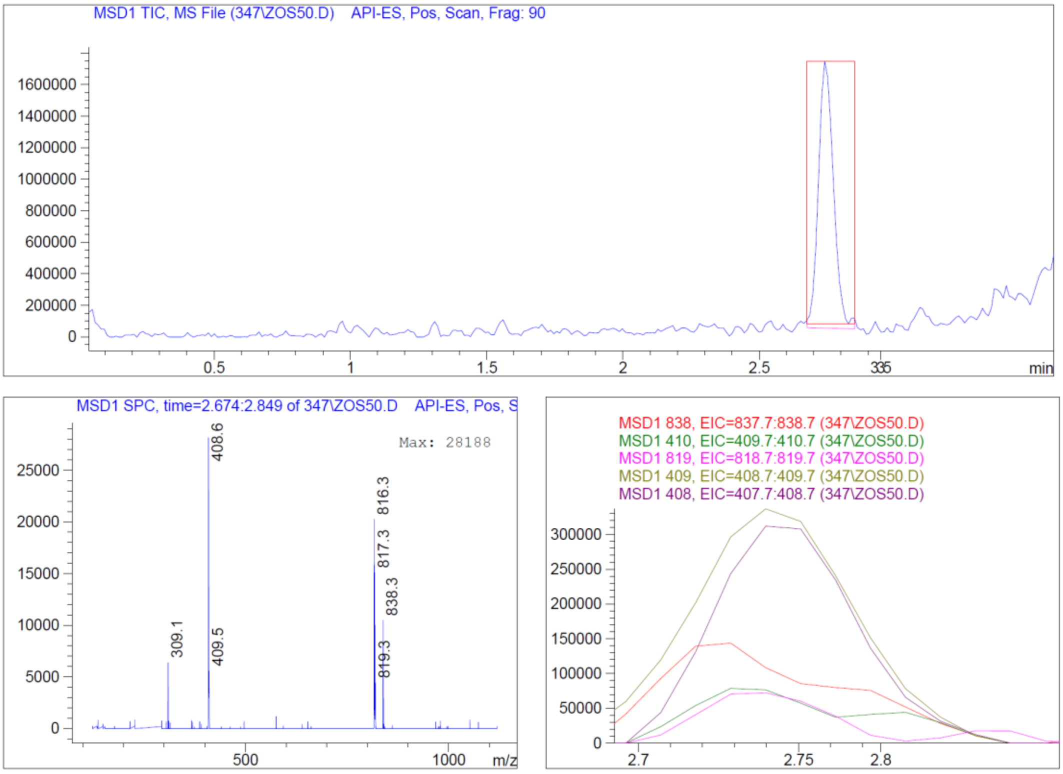

Figure S105. Mass-spectra for **Compound 18f** (positive ions). Found mass for $C_{40}H_{33}ClF_3N_7O_5S$ = 816.3 $[M+H]^+$

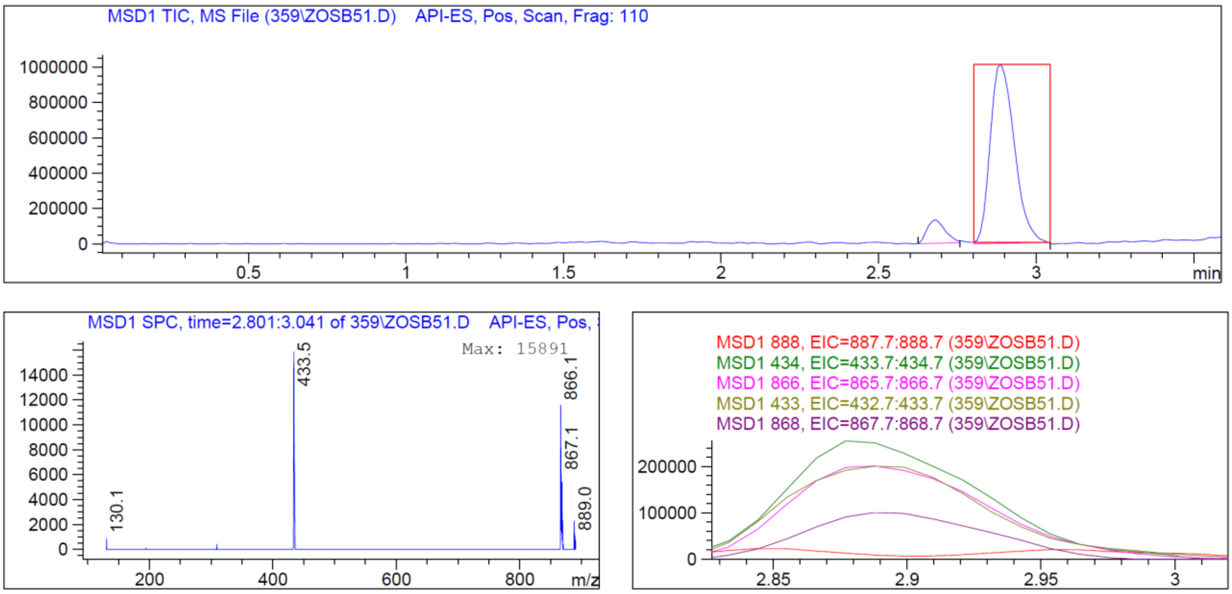

Figure S106. Mass-spectra for **Compound 19f** (positive ions). Found mass for $C_{41}H_{33}ClF_5N_7O_5S$ = 866.1 $[M+H]^+$

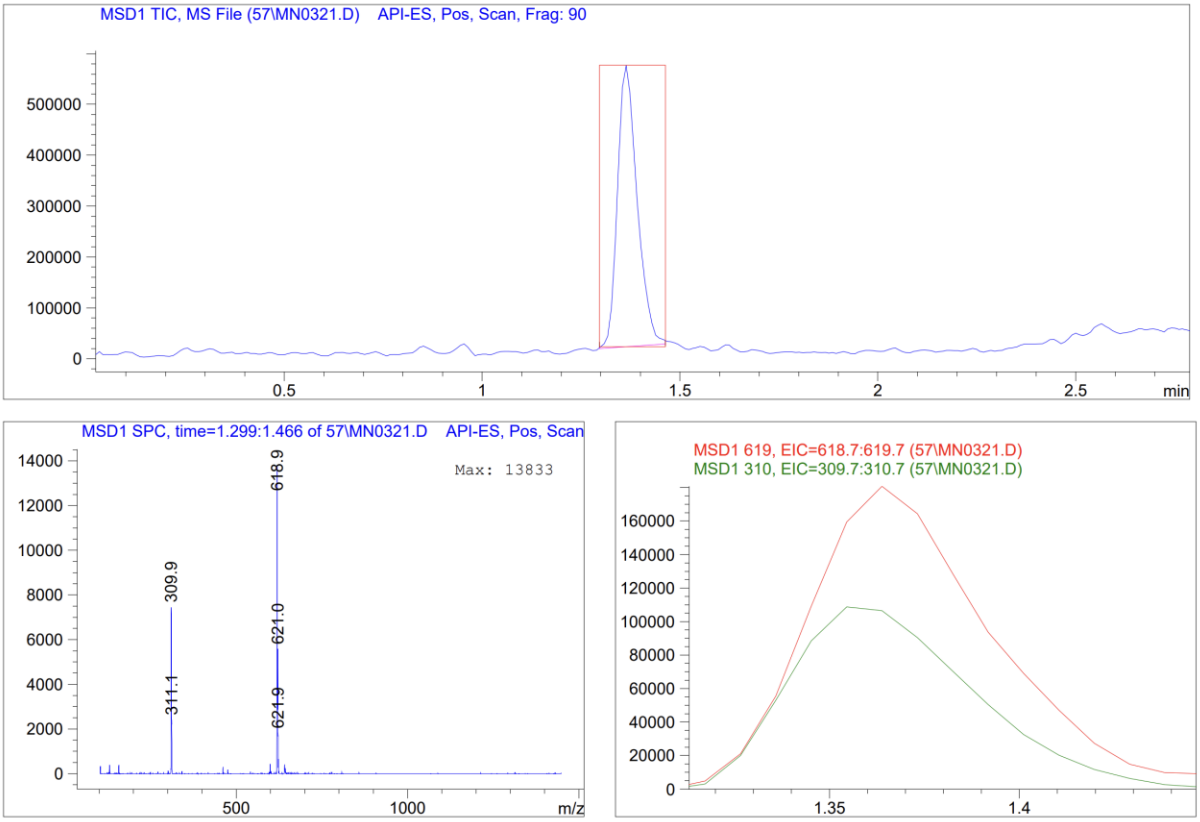

Figure S107. Mass-spectra for **Compound 28**(positive ions). Found mass for $C_{28}H_{29}ClF_2N_6O_4S$ = 618.9 $[M+H]^+$

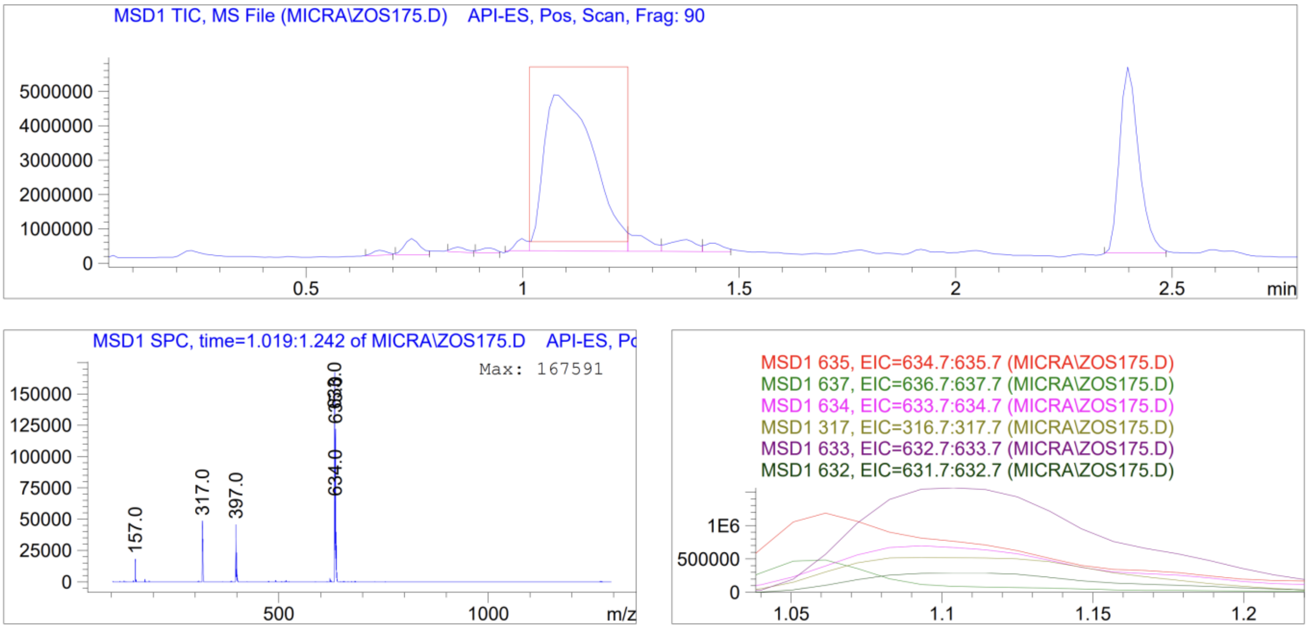

Figure S108. Mass-spectra for **Compound 28**(positive ions). Found mass for $C_{29}H_{31}ClF_2N_6O_4S$ = 633 $[M]^+$

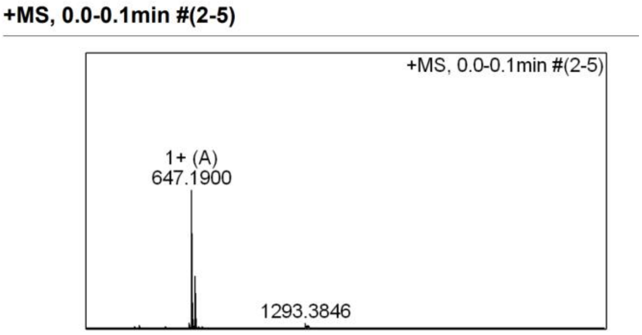

#	m/z	Res.	S/N	I	I %
1	633.1839	11456	233.1	104644	3.6
2	647.1900	28098	6216.2	2937736	100.0
3	648.2123	24902	5401.1	2562172	87.2
4	649.2056	19687	4904.3	2335048	79.5
5	650.2052	19108	2511.4	1200181	40.9
6	651.1977	14856	598.0	286979	9.8
7	665.2149	18718	2181.4	1099616	37.4
8	666.2131	13914	616.3	311865	10.6
9	667.2095	15971	819.2	415940	14.2
10	668.2073	11567	230.3	117497	4.0

Figure S109. Mass-spectra for **Compound 32** (positive ions). Found mass for $C_{30}H_{33}ClF_2N_6O_4S$ = 647.1900 $[M+H]^+$

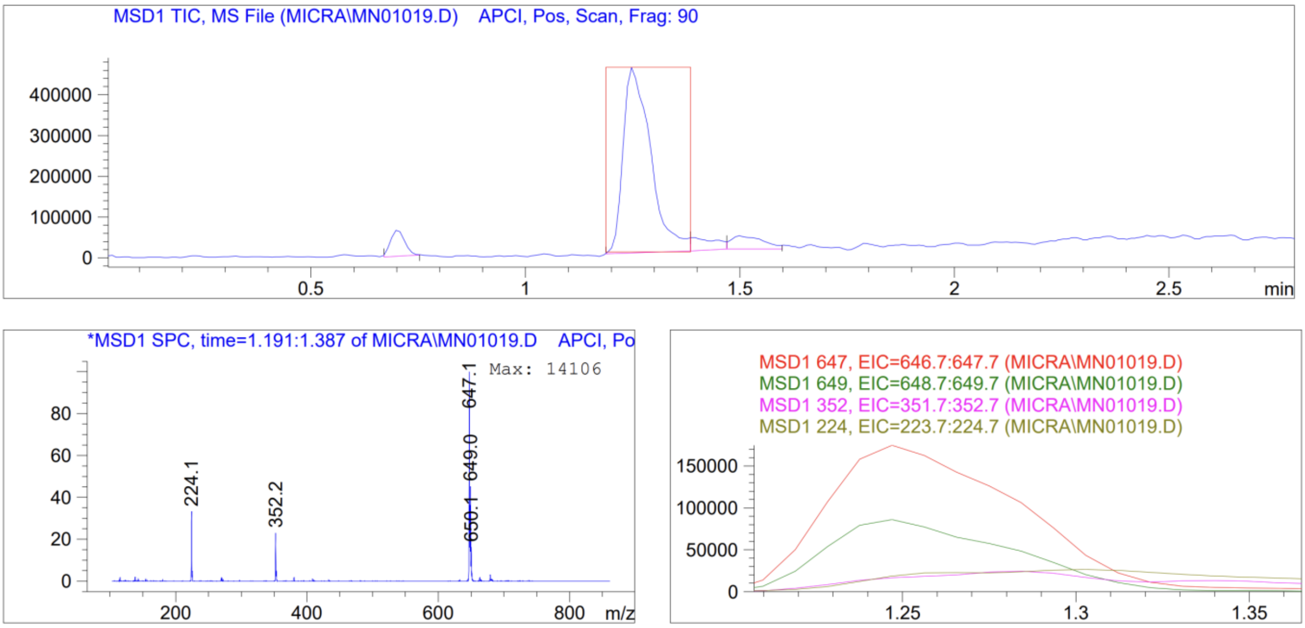

Figure S110. Mass-spectra for **Compound 34** (positive ions). Found mass for $C_{30}H_{33}ClF_2N_6O_4S$ = 647.1 $[M+H]^+$

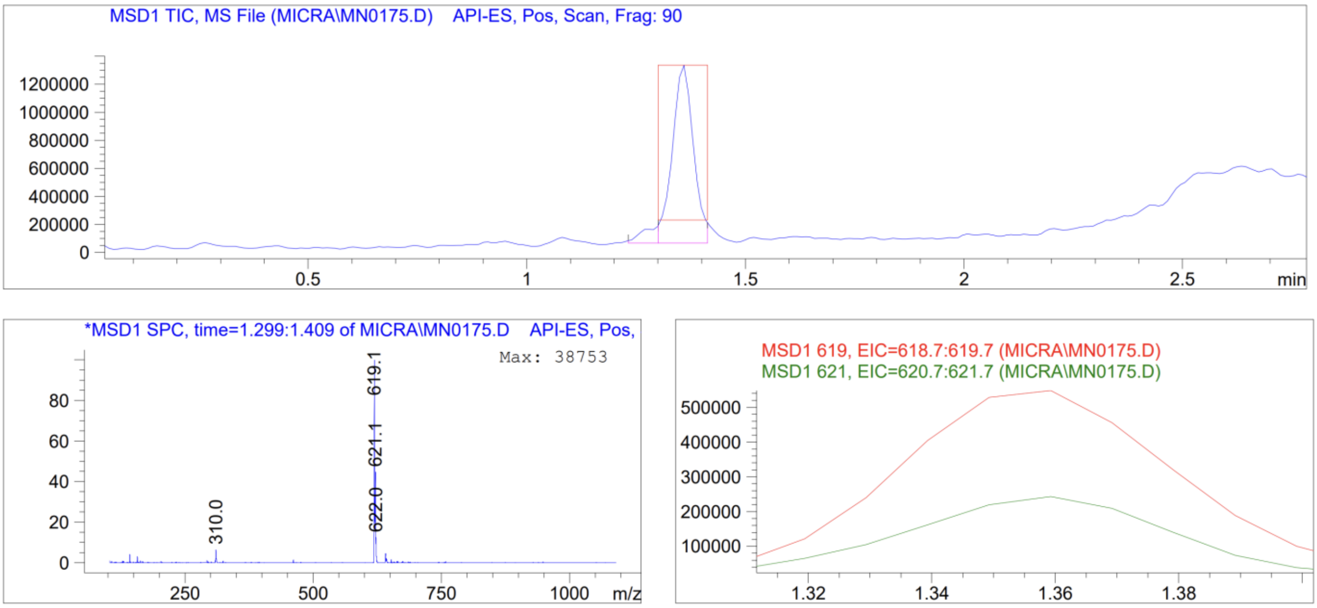

Figure S111. Mass-spectra for **Compound 35** (positive ions). Found mass for $C_{28}H_{29}ClF_2N_6O_4S$ = 619.1 $[M+H]^+$

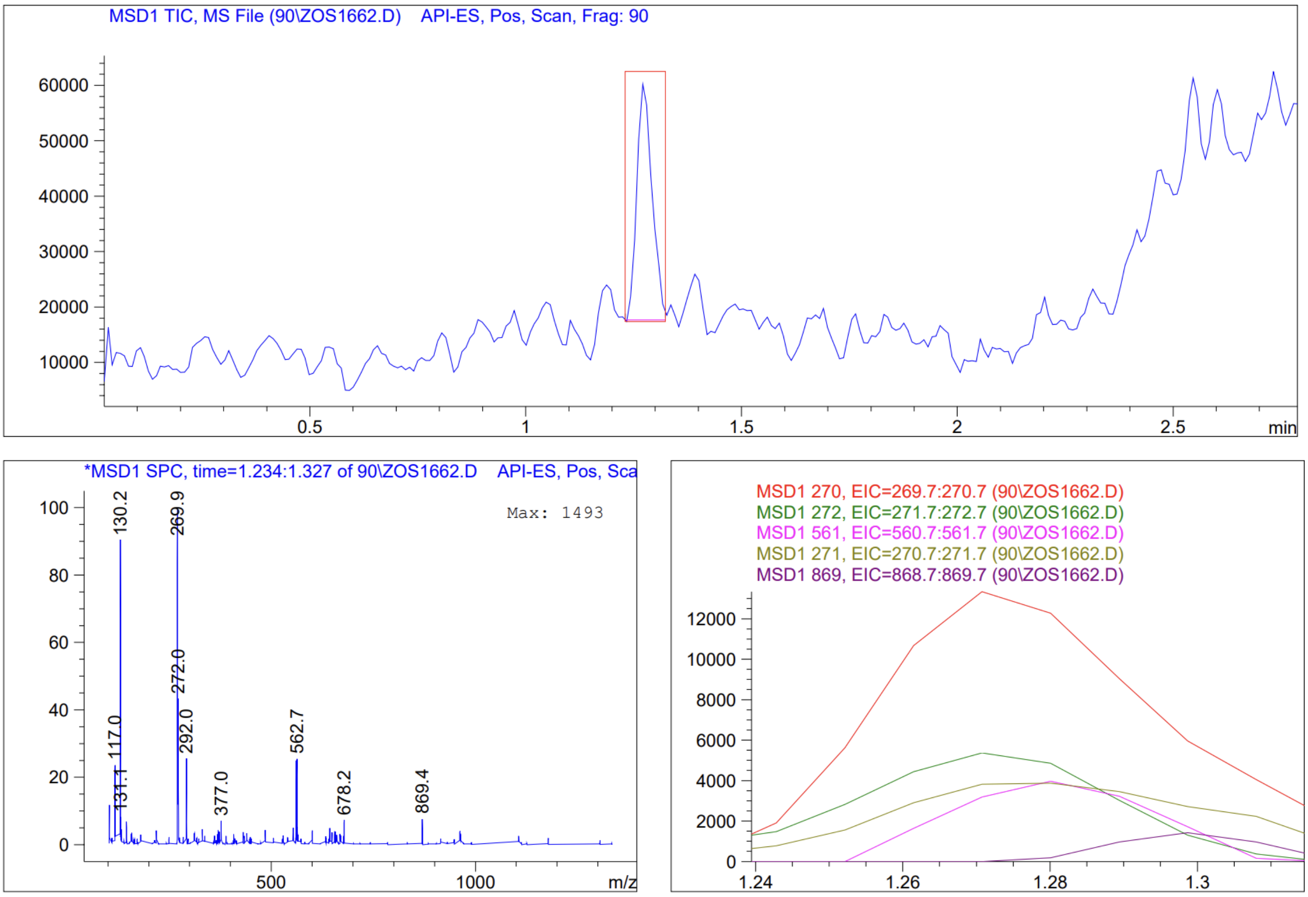

Figure S112. Mass-spectra for **Compound 36** (positive ions). Found mass for $C_{12}H_{12}ClNO_2S$ = 269.9 $[M]^+$

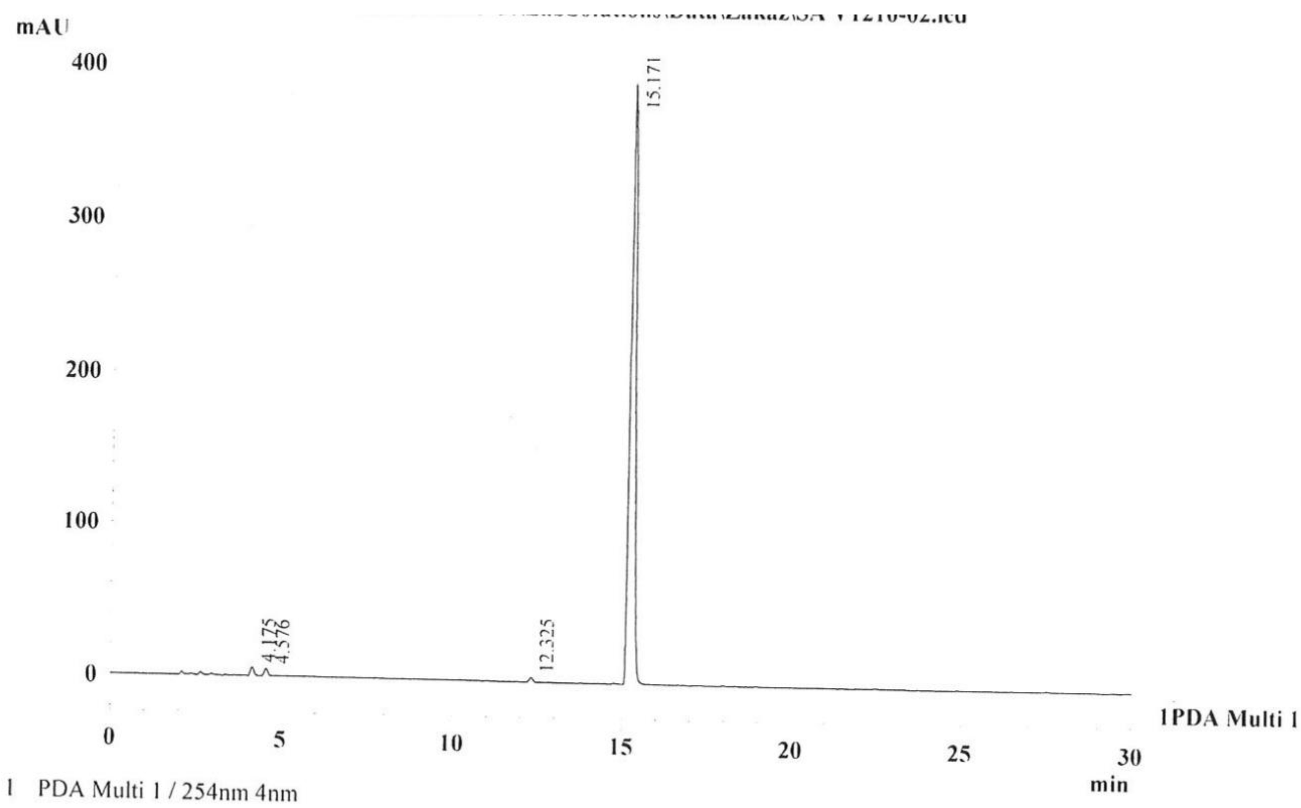

PeakTable

PDA Ch1 254nm 4nm

Peak#	Ret. Time	Area	Height	Area %
1	4.175	40594	5640	0.955
2	4.576	31715	4668	0.746
3	12.325	21246	2947	0.500
4	15.171	4157769	392251	97.799
Total		4251324	405506	100.000

Method

<<LC Program>>

Time	Unit	Command	Value
0.10	Pumps	B.Conc	20
30.00	Pumps	B.Conc	80
33.00	Pumps	B.Conc	20
45.00	Controller	Stop	

Method Filename : FOS B.lcm

Shimadzu LC-20AD; 2-System FOS, Colon Kromasil 100-C18,.size 5mkm, 4,6*250mm, N 86912
Elution: A - H3PO4 0.01M pH 2.6; B - MeCN, fl. 1,0 ml/min, loop 20mkl.

Figure S113. HPLC chromatogram of **Compound 6a**

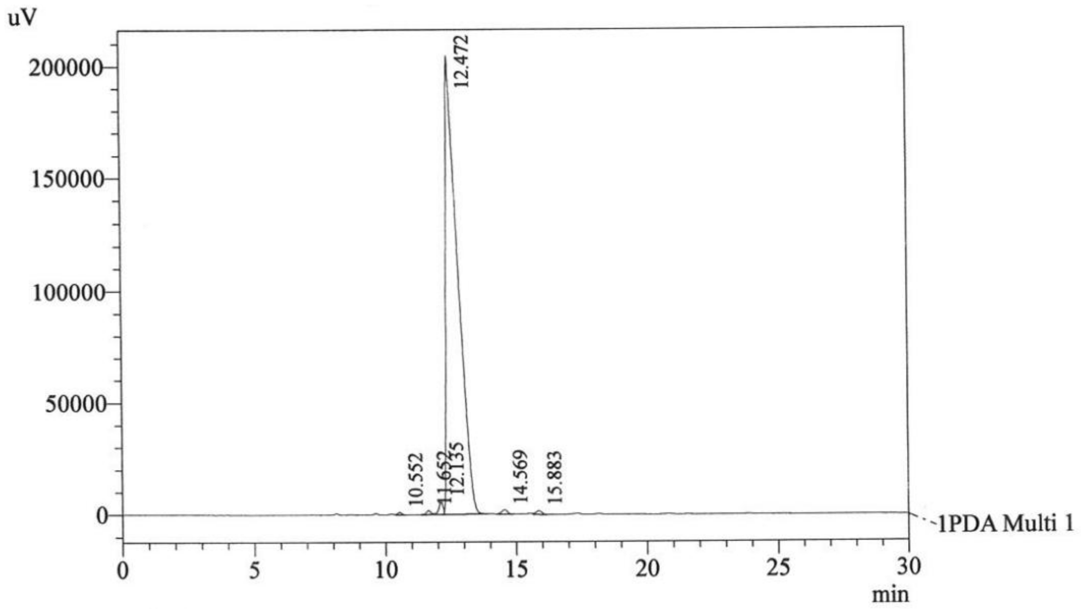

1 PDA Multi 1 / 490nm 4nm

PDA Ch1 490nm 4nm

Peak#	Ret. Time	Area	Height	Area %
1	10.552	10785	1030	0.160
2	11.652	20873	1802	0.310
3	12.135	64663	5670	0.960
4	12.472	6585445	204631	97.775
5	14.569	29757	1962	0.442
6	15.883	23813	1602	0.354
Total		6735335	216697	100.000

Method Filename : FOS C.lcm 27.03.2023 12:53:44

Time	Unit	Command	Valu
0.01	Pumps	B.Conc	50
30.00	Pumps	B.Conc	90
33.00	Pumps	B.Conc	50
45.00	Controller	Stop	

Shimadzu LC-20 AD; System - FOS Colon- Kromasil-100-5mkm. C-18, 4,6x250 mm. N 62511
Elution: A - H3PO4 0,01M pH 2,6; B - MeCN, fl - 1.0 ml/min, loop 20 mkl

Figure S114. HPLC chromatogram of **Compound 6b**

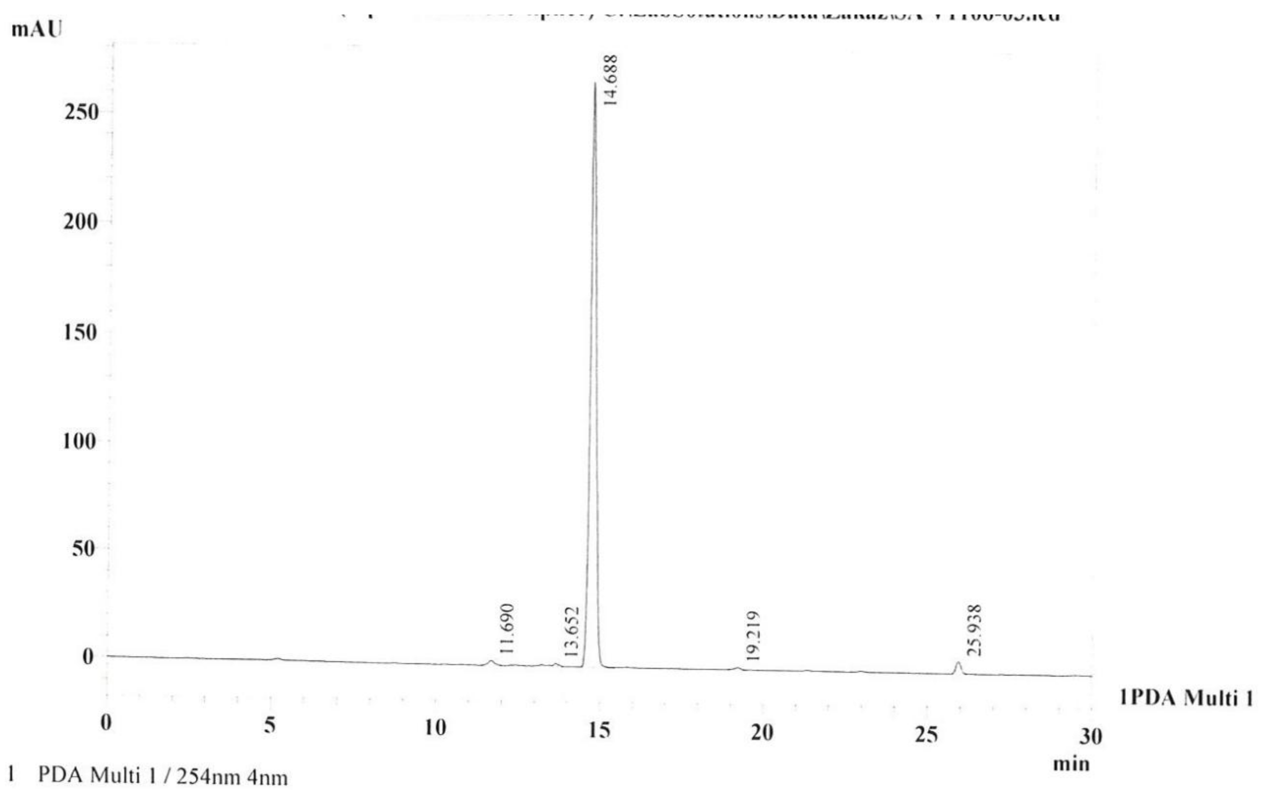

1 PDA Multi 1 / 254nm 4nm

PeakTable

PDA Ch1 254nm 4nm

Peak#	Ret. Time	Area	Height	Area %
1	11.690	31939	2256	0.923
2	13.652	15333	1494	0.443
3	14.688	3338492	268144	96.486
4	19.219	15467	1025	0.447
5	25.938	58842	5795	1.701
Total		3460074	278715	100.000

Method

<<LC Program>>

Time	Unit	Command	Value
0.10	Pumps	B.Conc	20
30.00	Pumps	B.Conc	80
33.00	Pumps	B.Conc	20
45.00	Controller	Stop	

Method Filename : FOS B.lcm

Shimadzu LC-20AD; 2-System FOS, Colon Kromasil 100-C18,.size 5mkm, 4,6*250mm, N 86912
Elution: A - H3PO4 0.01M pH 2.6; B - MeCN, fl. 1,0 ml/min, loop 20mkl.

Figure S115. HPLC chromatogram of **Compound 6c**

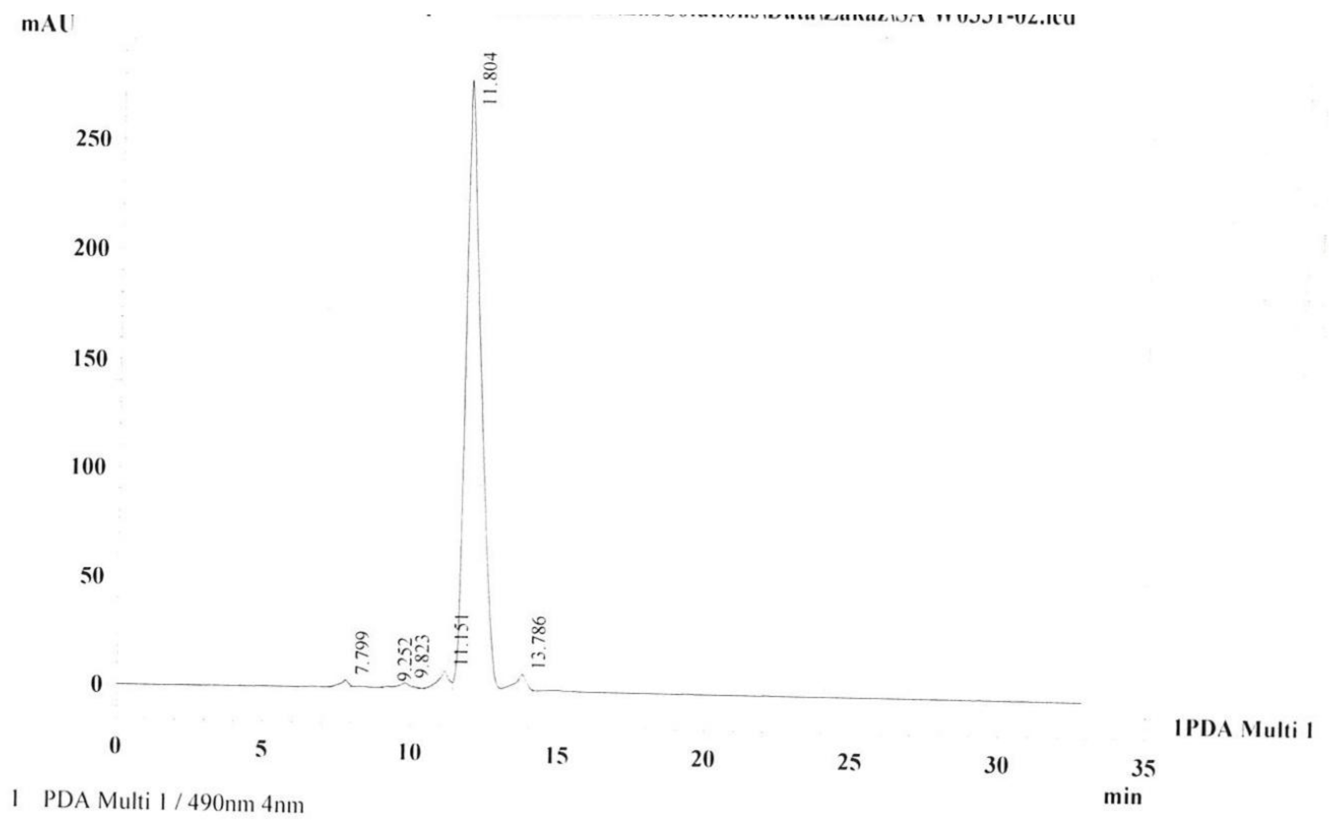

PeakTable

PDA Ch1 490nm 4nm

Peak#	Ret. Time	Area	Height	Area %
1	7.799	58303	3045	0.557
2	9.252	11326	682	0.108
3	9.823	58154	2395	0.555
4	11.151	196478	7980	1.876
5	11.804	9952250	279025	95.036
6	13.786	195595	7193	1.868
Total		10472107	300319	100.000

Method

<<LC Program>>

Time	Unit	Command	Value
0.10	Pumps	B.Conc	50
30.00	Pumps	B.Conc	90
33.00	Pumps	B.Conc	50
45.00	Controller	Stop	

Method Filename : FOS C.lcm

Shimadzu LC-20AD; 2-System FOS, Colon Kromasil 100-C18,.size 5mkm, 4,6*250mm, N 86912
Elution: A - H3PO4 0.01M pH 2.6; B - MeCN, fl. 1,0 ml/min, loop 20mkl.

Figure S116. HPLC chromatogram of **Compound 6d**

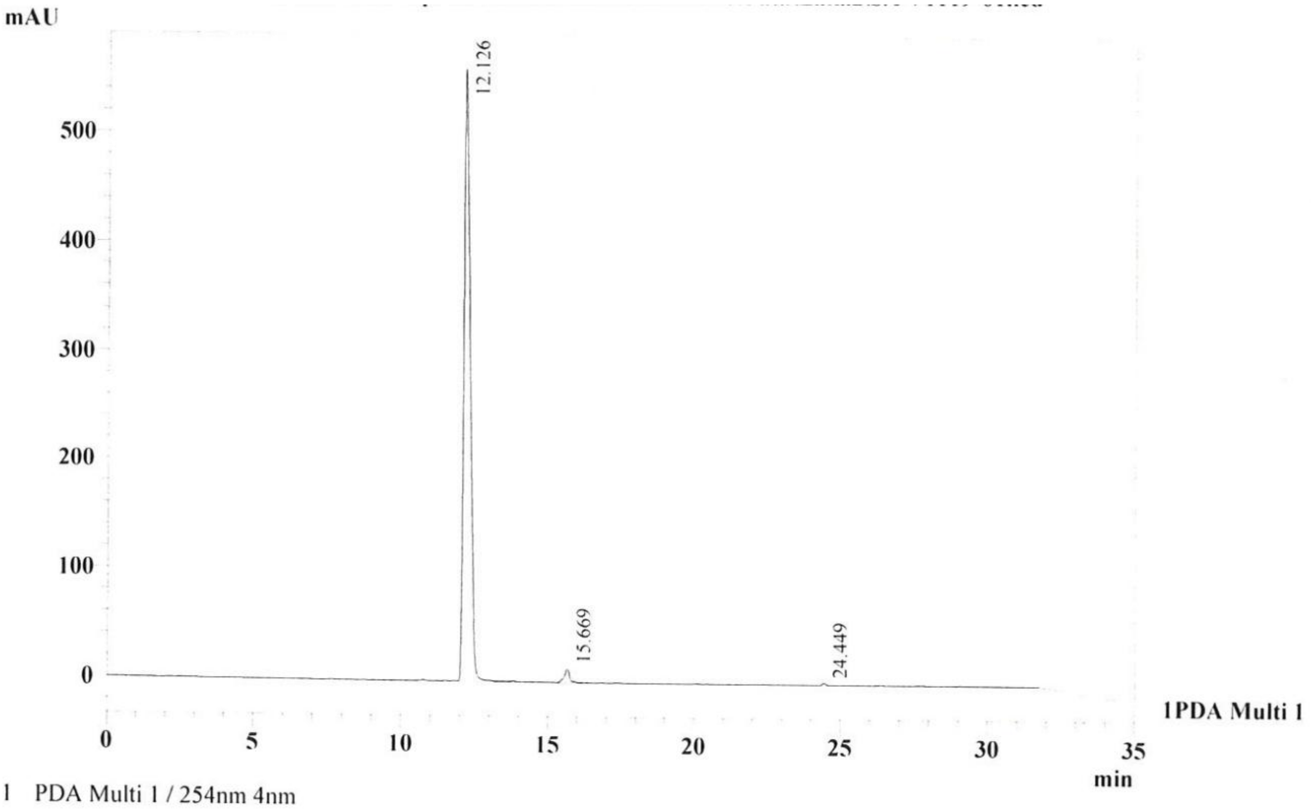

1 PDA Multi 1 / 254nm 4nm

PeakTable

PDA Ch1 254nm 4nm

Peak#	Ret. Time	Area	Height	Area %
1	12.126	8452067	560526	98.050
2	15.669	150447	12673	1.745
3	24.449	17666	2283	0.205
Total		8620180	575482	100.000

Method

<<LC Program>>

Time	Unit	Command	Value
0.10	Pumps	B.Conc	20
30.00	Pumps	B.Conc	80
33.00	Pumps	B.Conc	20
45.00	Controller	Stop	

Method Filename : FOS B.lcm

Shimadzu LC-20AD; 2-System FOS, Colon Kromasil 100-C18,.size 5mkm, 4,6*250mm, N 86912
Elution: A - H3PO4 0.01M pH 2.6; B - MeCN, fl. 1,0 ml/min, loop 20mkl.

Figure S117. HPLC chromatogram of **Compound 7e**

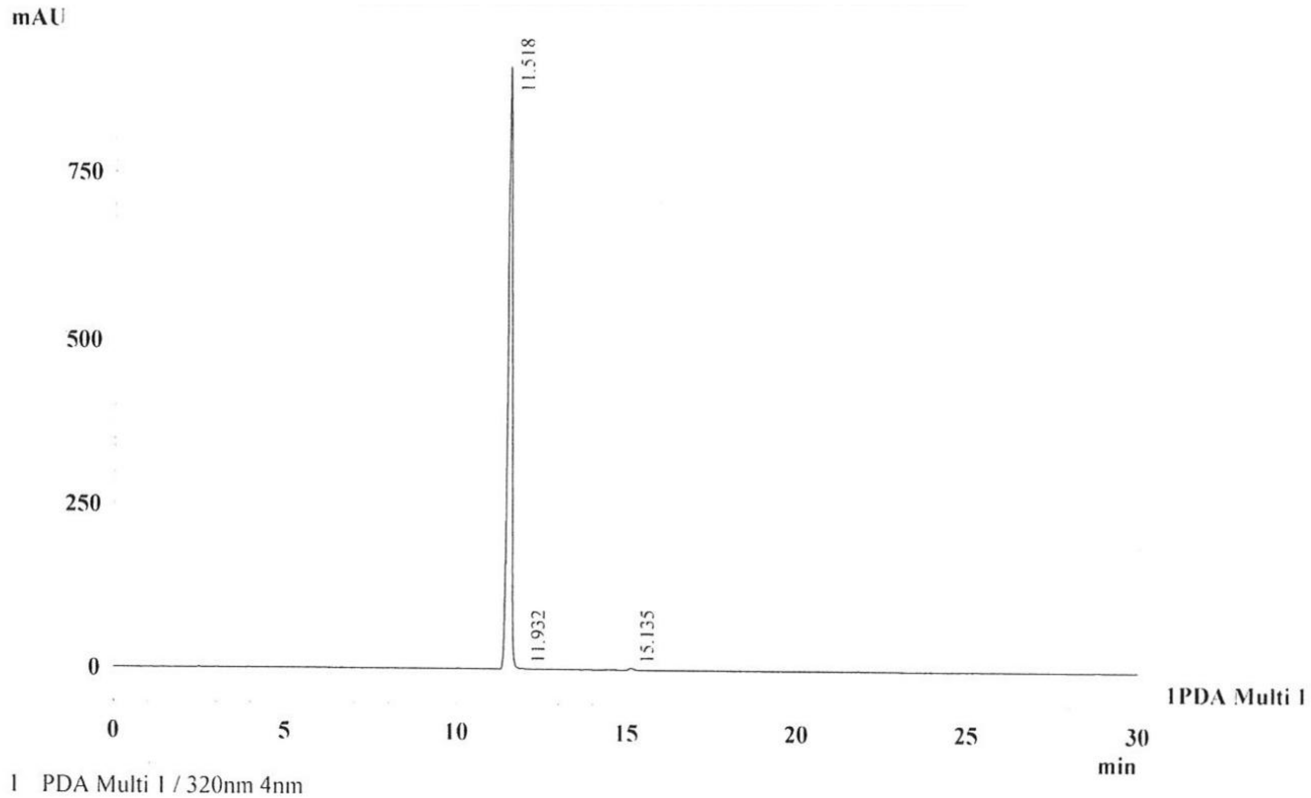

1 PDA Multi 1 / 320nm 4nm

PeakTable

PDA Ch1 320nm 4nm

Peak#	Ret. Time	Area	Height	Area %
1	11.518	7851732	911214	99.398
2	11.932	16195	1394	0.205
3	15.135	31366	3498	0.397
Total		7899294	916105	100.000

Method

<<LC Program>>

Time	Unit	Command	Value
0.10	Pumps	B.Conc	50
30.00	Pumps	B.Conc	95
33.00	Pumps	B.Conc	50
45.00	Controller	Stop	

Method Filename : FOS C.lcm

Shimadzu LC-20AD; 2-System FOS, Colon Kromasil 100-C18,.size 5mkm, 4,6*250mm, N 86912
Elution: A - H3PO4 0.01M pH 2.6; B - MeCN, fl. 1,0 ml/min, loop 20mkl.

Figure S118. HPLC chromatogram of **Compound 8f**

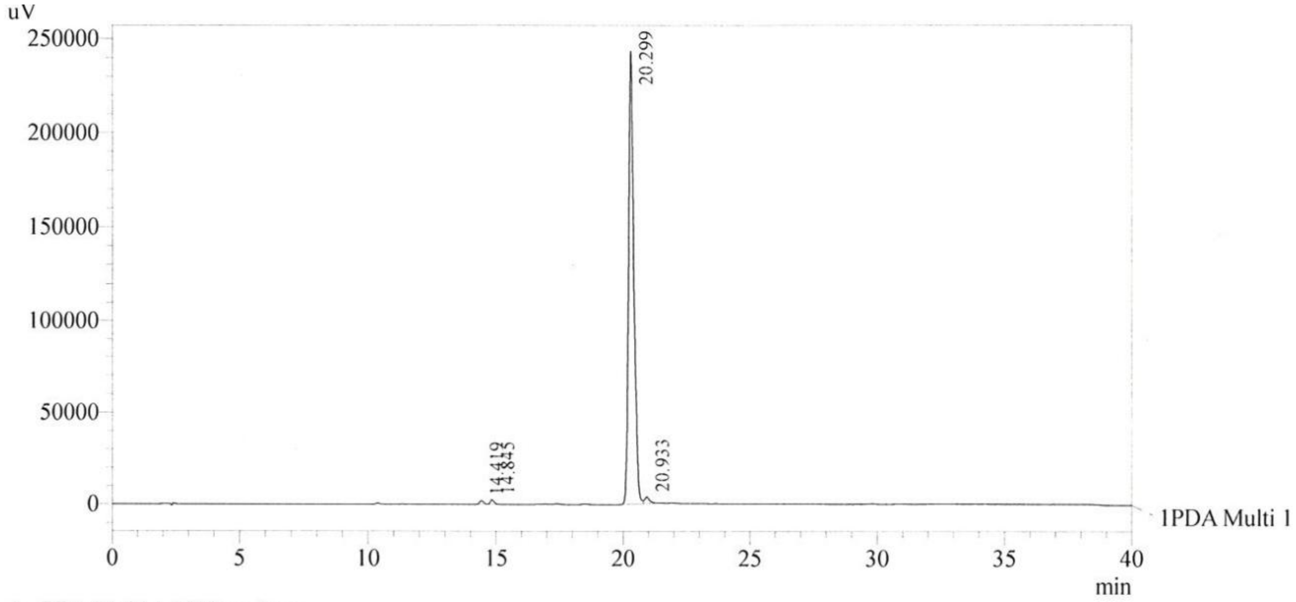

1 PDA Multi 1 / 330nm 4nm

PDA Ch1 330nm 4nm

Peak#	Ret. Time	Area	Height	Area %
1	14.419	22779	2190	0.607
2	14.845	27950	2681	0.744
3	20.299	3649877	243135	97.196
4	20.933	54580	3758	1.453
Total		3755186	251765	100.000

Method Filename : FRA02BvB.lcm 12.01.2026 14:37:11

Time	Unit	Command	Valu
0.01	Pumps	B.Conc	30
30.00	Pumps	B.Conc	80
40.00	Pumps	B.Conc	90
45.00	Pumps	B.Conc	30
55.00	Controller	Stop	

Shimadzu LC-20 AD; System - FRA 02 vA, Colon- Kromasil C-18, 4.6x250 mm. 5 mkm, N 59967
Elution: A - COOHNH4 0.2% pH 4.5; B - MeCN, fl - 1 ml/min, loop 20 mkl

Figure S119. HPLC chromatogram of **Compound 9g**

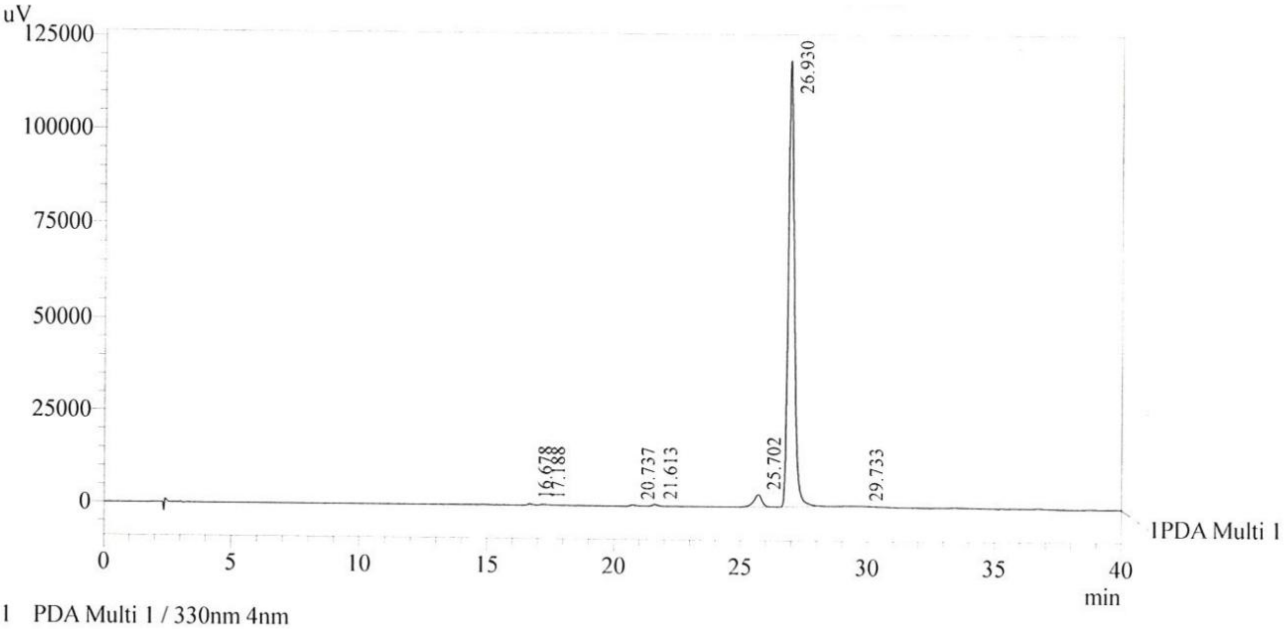

1 PDA Multi 1 / 330nm 4nm

PDA Ch1 330nm 4nm

Peak#	Ret. Time	Area	Height	Area %
1	16.678	4126	367	0.197
2	17.188	4382	275	0.209
3	20.737	6895	377	0.329
4	21.613	7698	493	0.367
5	25.702	75066	3363	3.577
6	26.930	1983924	119379	94.529
7	29.733	16647	240	0.793
Total		2098740	124494	100.000

Method Filename : FRA02BvB.lcm 12.01.2026 16:39:22

Time	Unit	Command	Valu
0.01	Pumps	B.Conc	30
30.00	Pumps	B.Conc	80
40.00	Pumps	B.Conc	90
45.00	Pumps	B.Conc	30
55.00	Controller	Stop	

Shimadzu LC-20 AD; System - FRA 02 vA, Colon- Kromasil C-18, 4.6x250 mm. 5 mkm, N 59967
Elution: A - COOHNH4 0.2% pH 4.5; B - MeCN, fl - 1 ml/min, loop 20 mkl

Figure S120. HPLC chromatogram of **Compound 13e**

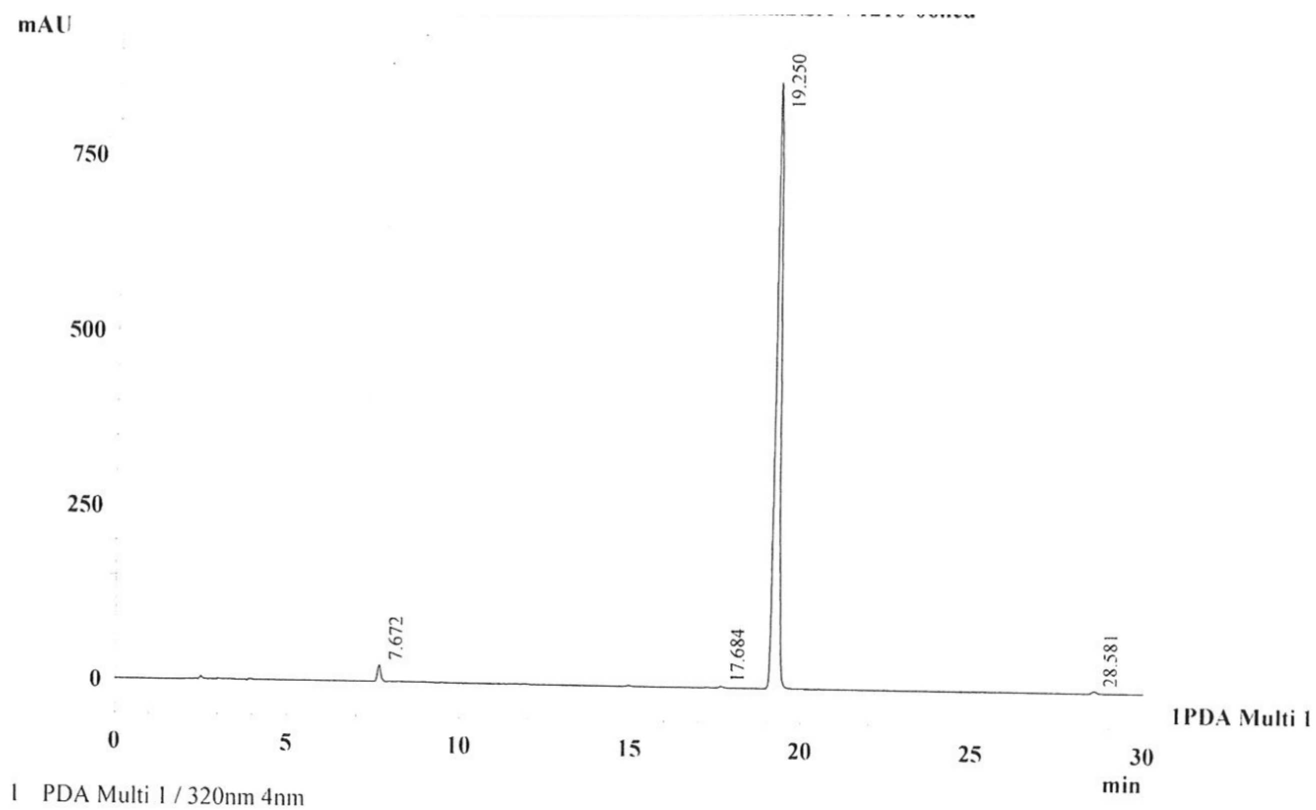

1 PDA Multi 1 / 320nm 4nm

PeakTable

PDA Ch1 320nm 4nm

Peak#	Ret. Time	Area	Height	Area %
1	7.672	161591	24412	1.798
2	17.684	20652	2104	0.230
3	19.250	8764574	864533	97.514
4	28.581	41206	3485	0.458
Total		8988024	894534	100.000

Method

<<LC Program>>

Time	Unit	Command	Value
0.10	Pumps	B.Conc	50
30.00	Pumps	B.Conc	95
33.00	Pumps	B.Conc	50
45.00	Controller	Stop	

Method Filename : FOS C.lcm

Shimadzu LC-20AD; 2-System FOS, Colon Kromasil 100-C18,.size 5mkm, 4,6*250mm, N 86912
Elution: A - H3PO4 0.01M pH 2.6; B - MeCN, fl. 1,0 ml/min, loop 20mkl.

Figure S121. HPLC chromatogram of **Compound 13f**

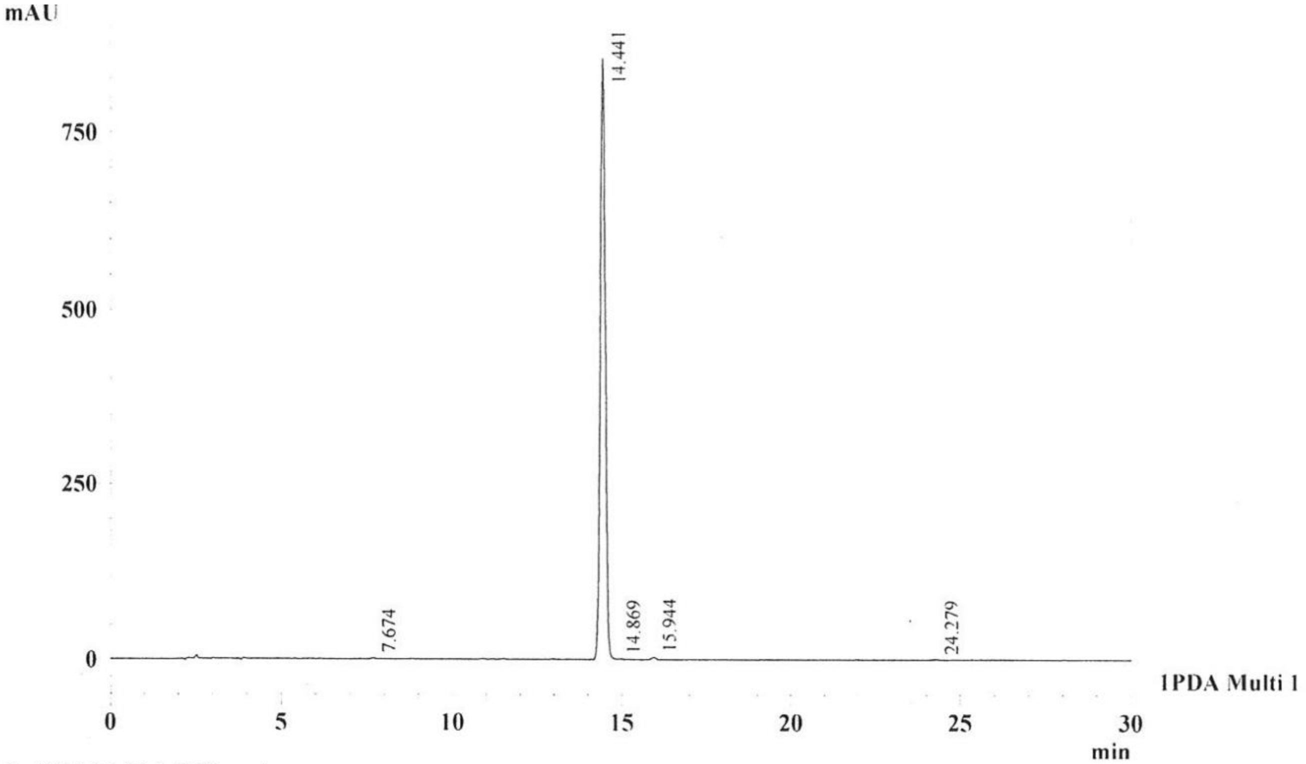

1 PDA Multi 1 / 320nm 4nm

PeakTable

PDA Ch1 320nm 4nm

Peak#	Ret. Time	Area	Height	Area %
1	7.674	10350	1288	0.116
2	14.441	8816887	854457	99.153
3	14.869	18927	868	0.213
4	15.944	29412	3106	0.331
5	24.279	16604	1098	0.187
Total		8892180	860816	100.000

Method

<<LC Program>>

Time	Unit	Command	Value
0.10	Pumps	B.Conc	50
30.00	Pumps	B.Conc	95
33.00	Pumps	B.Conc	50
45.00	Controller	Stop	

Method Filename : FOS C.lcm

Shimadzu LC-20AD; 2-System FOS, Colon Kromasil 100-C18,.size 5mkm, 4,6*250mm, N 86912
Elution: A - H3PO4 0.01M pH 2.6; B - MeCN, fl. 1,0 ml/min, loop 20mkl.

Figure S122. HPLC chromatogram of **Compound 13g**

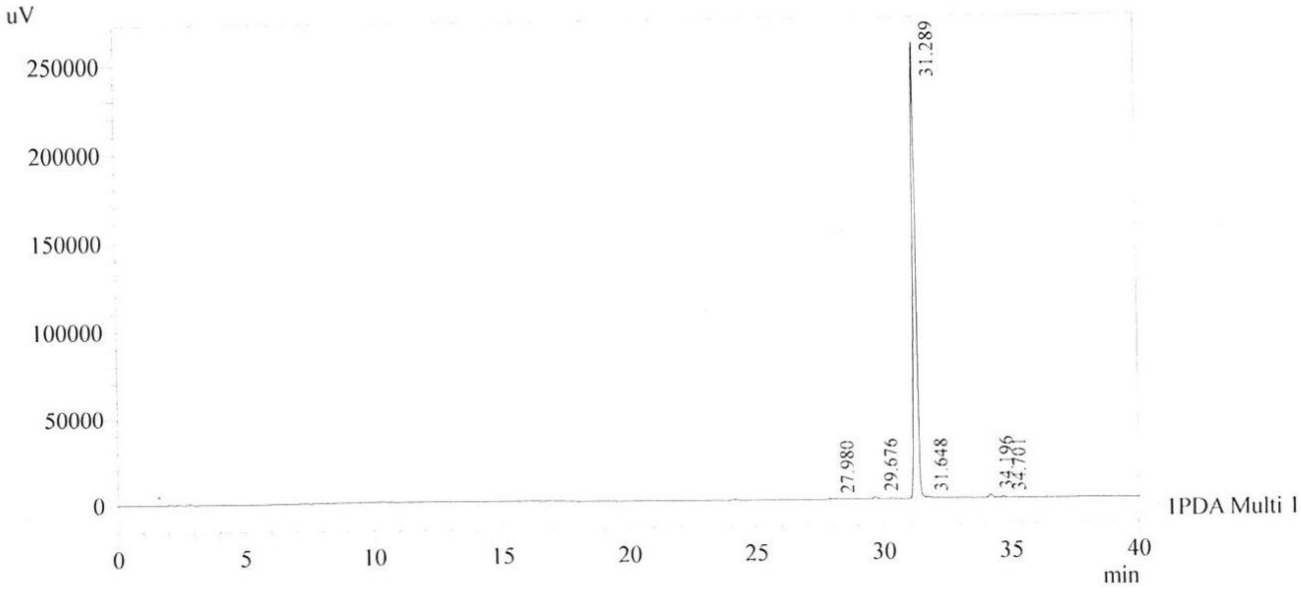

1 PDA Multi 1 / 328nm 4nm

PDA Ch1 328nm 4nm

Peak#	Ret. Time	Area	Height	Area %
1	27.980	7378	378	0.316
2	29.676	10420	1150	0.446
3	31.289	2278231	258369	97.590
4	31.648	12447	825	0.533
5	34.196	18484	1833	0.792
6	34.701	7533	690	0.323
Total		2334493	263244	100.000

Figure S123. HPLC chromatogram of **Compound 17f**

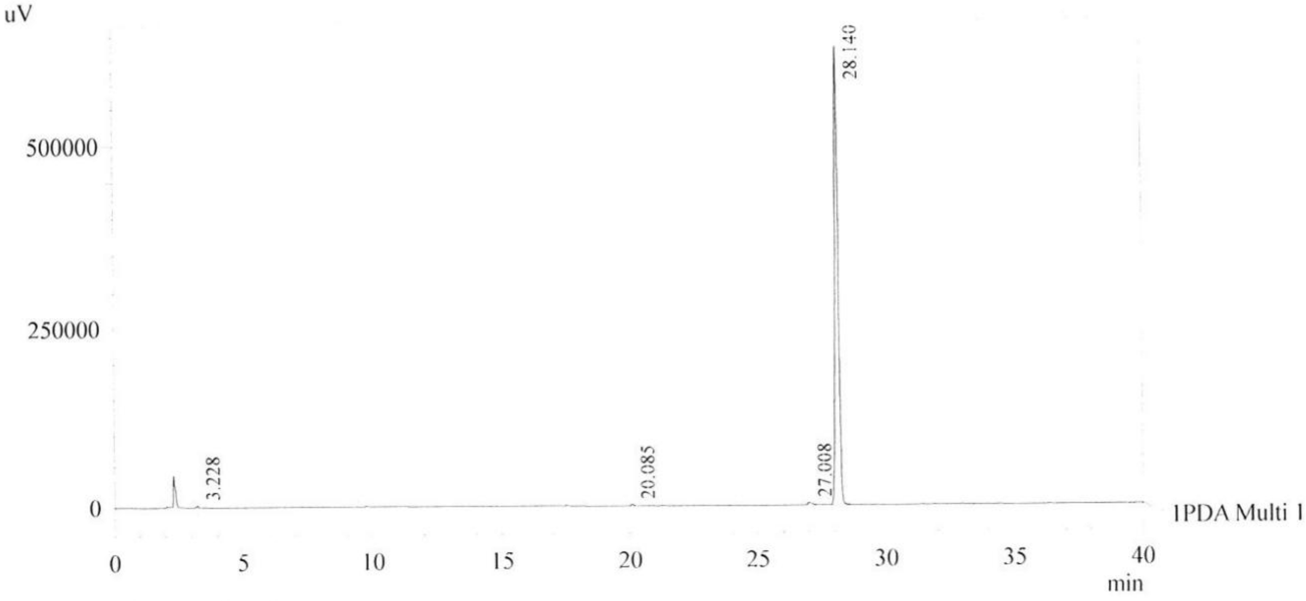

1 PDA Multi 1 / 254nm 4nm

PDA Ch1 254nm 4nm

Peak#	Ret. Time	Area	Height	Area %
1	3.228	16764	3158	0.277
2	20.085	18833	2536	0.311
3	27.008	45763	3693	0.757
4	28.140	5966012	632813	98.655
Total		6047372	642201	100.000

Figure S124. HPLC chromatogram of **Compound 18f**

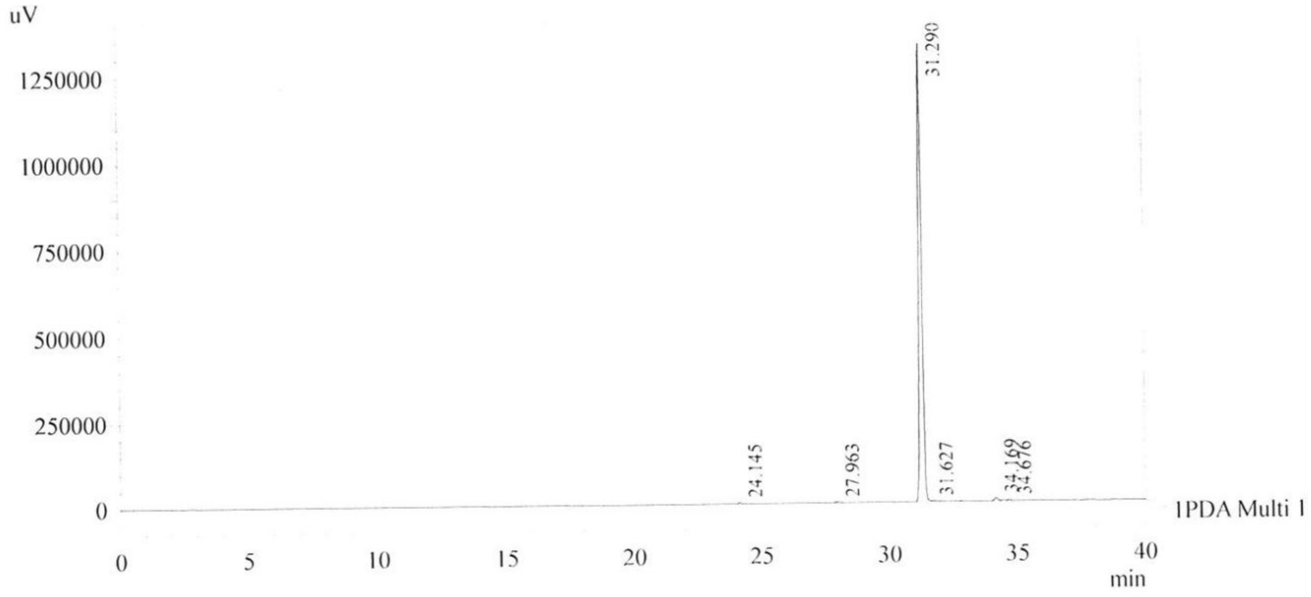

1 PDA Multi 1 / 328nm 4nm

PDA Ch1 328nm 4nm

Peak#	Ret. Time	Area	Height	Area %
1	24.145	29306	3623	0.252
2	27.963	36801	2018	0.317
3	31.290	11351944	1326036	97.790
4	31.627	68714	2780	0.592
5	34.169	89894	8765	0.774
6	34.676	31870	3557	0.275
Total		11608530	1346779	100.000

Figure S125. HPLC chromatogram of **Compound 19f**

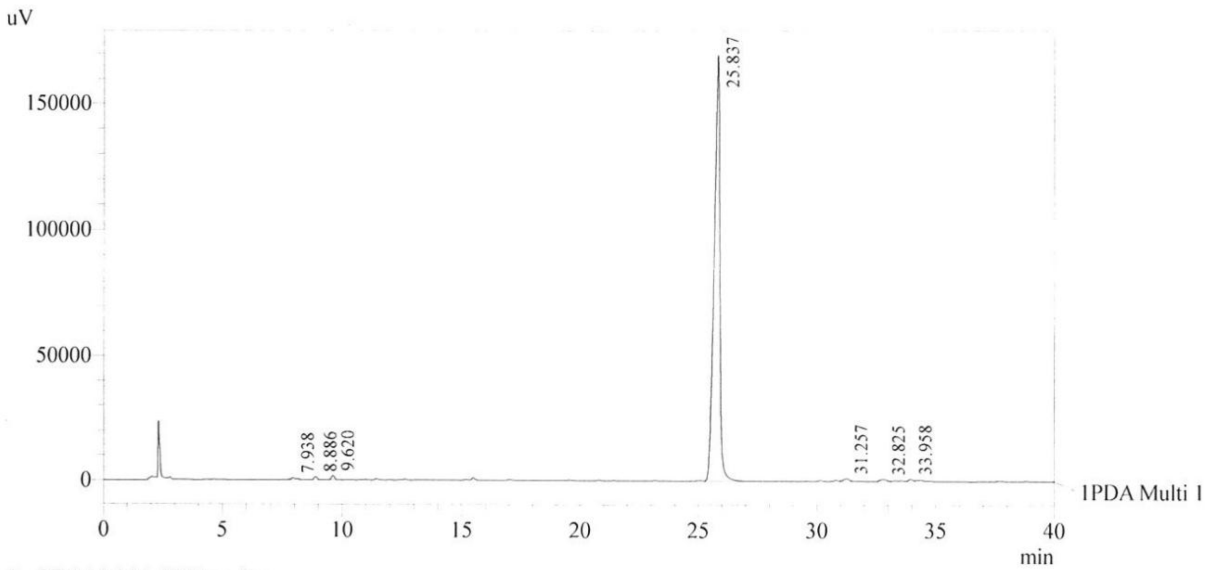

1 PDA Multi 1 / 254nm 4nm

PDA Ch1 254nm 4nm

Peak#	Ret. Time	Area	Height	Area %
1	7.938	10796	749	0.362
2	8.886	12692	1305	0.426
3	9.620	16888	1789	0.567
4	25.837	2891300	169409	97.033
5	31.257	20247	1086	0.680
6	32.825	17117	761	0.574
7	33.958	10673	782	0.358
Total		2979714	175881	100.000

Method Filename : FRA02BvB.lcm 25.11.2025 11:37:15

Time	Unit	Command	Valu
0.01	Pumps	B.Conc	30
30.00	Pumps	B.Conc	80
40.00	Pumps	B.Conc	90
45.00	Pumps	B.Conc	30
55.00	Controller	Stop	

Shimadzu LC-20 AD; System - FRA 02 vA, Colon- Kromasil C-18, 4.6x250 mm. 5 mkm, N 59967
Elution: A - COOHNH4 0.2% pH 4.5; B - MeCN, fl - 1 ml/min, loop 20 mkl

Figure S126. HPLC chromatogram of **Compound 28**

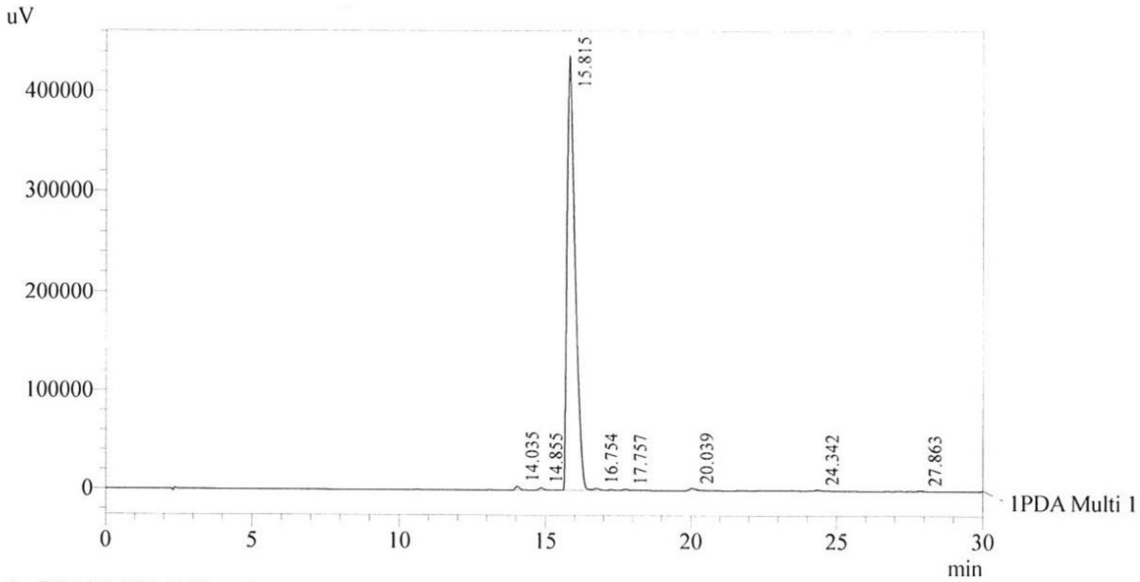

1 PDA Multi 1 / 330nm 4nm

PDA Ch1 330nm 4nm

Peak#	Ret. Time	Area	Height	Area %
1	14.035	44213	3929	0.517
2	14.855	34179	2459	0.400
3	15.815	8313221	437073	97.172
4	16.754	49259	2192	0.576
5	17.757	43136	1420	0.504
6	20.039	42860	2410	0.501
7	24.342	15211	1090	0.178
8	27.863	13076	1068	0.153
Total		8555155	451642	100.000

Method Filename : FOS C.lcm 14.01.2026 13:29:36

Time	Unit	Command	Valu
0.01	Pumps	B.Conc	30
30.00	Pumps	B.Conc	90
33.00	Pumps	B.Conc	30
45.00	Controller	Stop	

Shimadzu LC-20 AD; System - FOS Colon- Kromasil-100-5mkm. C-18, 4,6x250 mm. N 62511
Elution: A - H3PO4 0,01M pH 2,6; B - MeCN, fl - 1.0 ml/min, loop 20 mkl

Figure S127. HPLC chromatogram of **Compound 31**

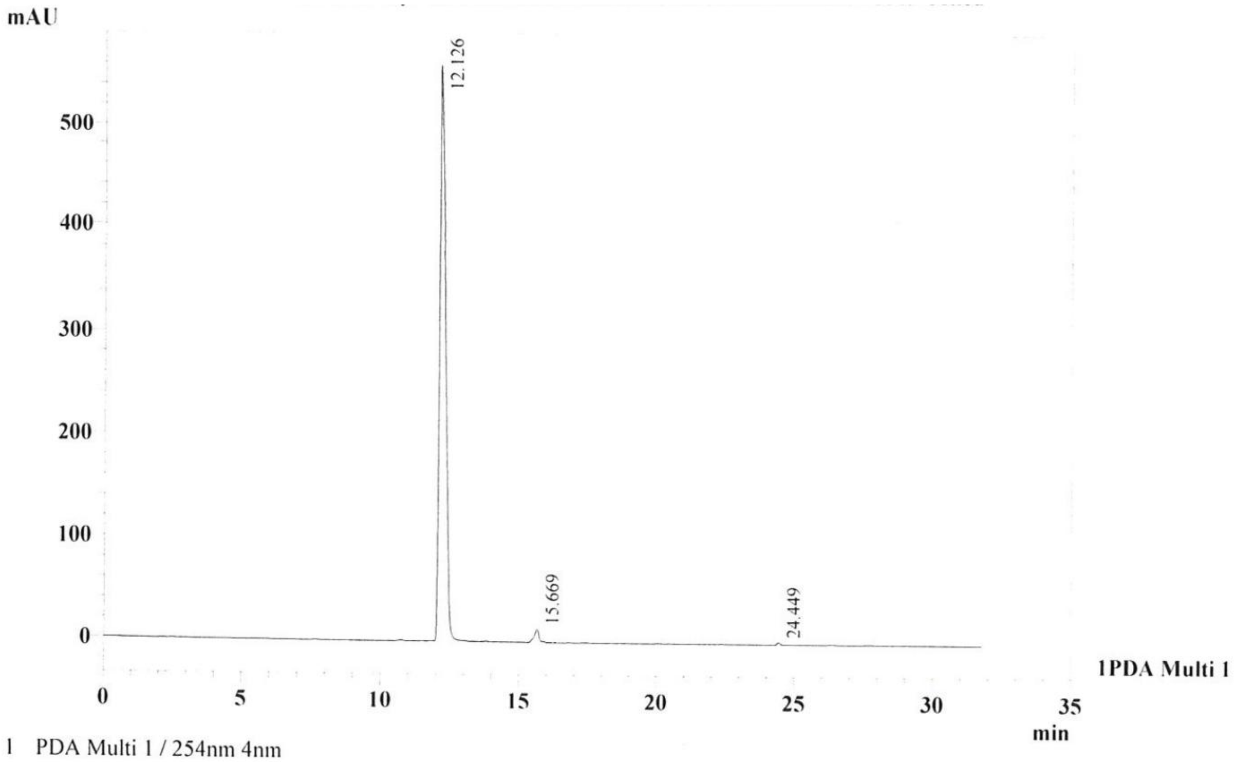

1 PDA Multi 1 / 254nm 4nm

PeakTable

PDA Ch1 254nm 4nm

Peak#	Ret. Time	Area	Height	Area %
1	12.126	8452067	560526	98.050
2	15.669	150447	12673	1.745
3	24.449	17666	2283	0.205
Total		8620180	575482	100.000

Method

<<LC Program>>

Time	Unit	Command	Value
0.10	Pumps	B.Conc	20
30.00	Pumps	B.Conc	80
33.00	Pumps	B.Conc	20
45.00	Controller	Stop	

Method Filename : FOS B.lcm

Shimadzu LC-20AD; 2-System FOS, Colon Kromasil 100-C18,.size 5mkm, 4,6*250mm, N 86912
Elution: A - H3PO4 0.01M pH 2.6; B - MeCN, fl. 1,0 ml/min, loop 20mkl.

Figure S128. HPLC chromatogram of **Compound 32**

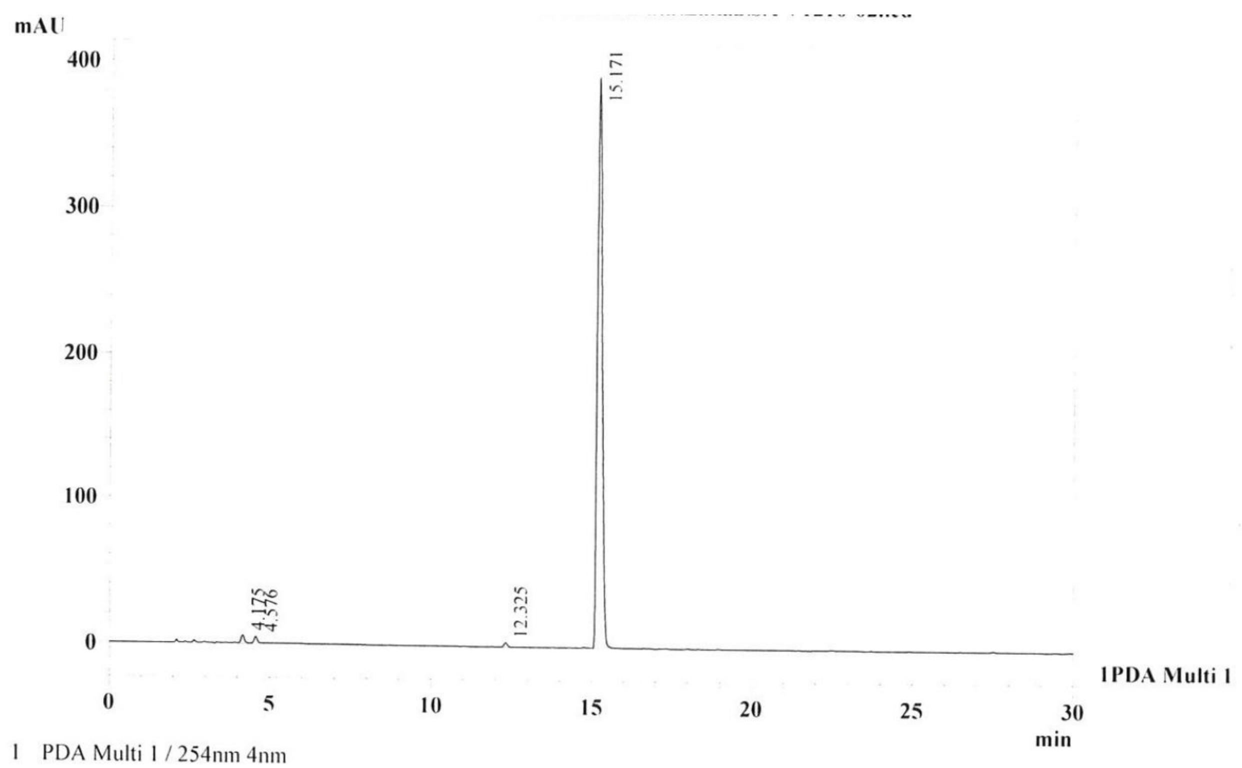

1 PDA Multi 1 / 254nm 4nm

PeakTable

PDA Ch1 254nm 4nm

Peak#	Ret. Time	Area	Height	Area %
1	4.175	40594	5640	0.955
2	4.576	31715	4668	0.746
3	12.325	21246	2947	0.500
4	15.171	4157769	392251	97.799
Total		4251324	405506	100.000

Method

<<LC Program>>

Time	Unit	Command	Value
0.10	Pumps	B.Conc	20
30.00	Pumps	B.Conc	80
33.00	Pumps	B.Conc	20
45.00	Controller	Stop	

Method Filename : FOS B.lcm

Shimadzu LC-20AD; 2-System FOS, Colon Kromasil 100-C18,.size 5mkm, 4,6*250mm, N 86912
Elution: A - H3PO4 0.01M pH 2.6; B - MeCN, fl. 1,0 ml/min, loop 20mkl.

Figure S129. HPLC chromatogram of **Compound 34**

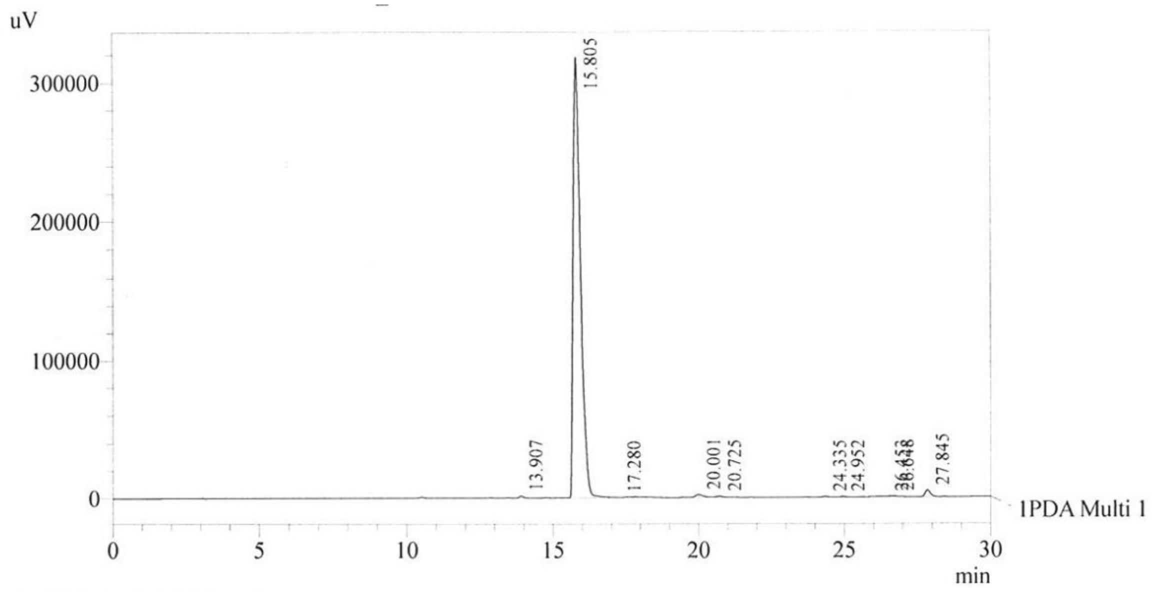

1 PDA Multi 1 / 330nm 4nm

PDA Ch1 330nm 4nm

Peak#	Ret. Time	Area	Height	Area %
1	13.907	18460	1737	0.318
2	15.805	5596686	317800	96.358
3	17.280	10913	269	0.188
4	20.001	45912	2252	0.790
5	20.725	15402	935	0.265
6	24.335	10810	740	0.186
7	24.952	11536	637	0.199
8	26.453	21739	545	0.374
9	26.648	17129	793	0.295
10	27.845	59645	4910	1.027
Total		5808232	330618	100.000

Method Filename : FOS C.lcm 14.01.2026 15:07:18

Time	Unit	Command	Valu
0.01	Pumps	B.Conc	30
30.00	Pumps	B.Conc	90
33.00	Pumps	B.Conc	30
45.00	Controller	Stop	

Shimadzu LC-20 AD; System - FOS Colon- Kromasil-100-5mkm. C-18, 4,6x250 mm. N 62511
Elution: A - H3PO4 0,01M pH 2,6; B - MeCN, fl - 1.0 ml/min, loop 20 mkl

Figure S130. HPLC chromatogram of **Compound 35**

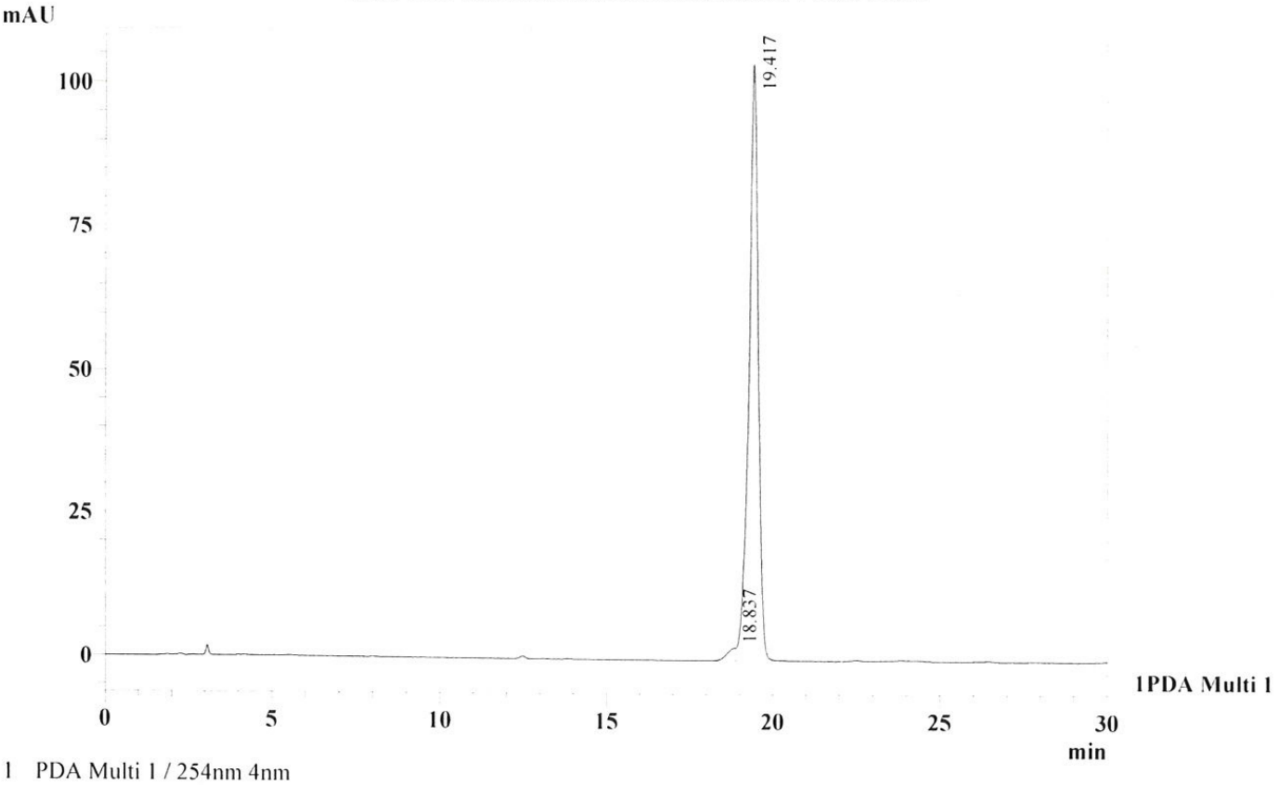

1 PDA Multi 1 / 254nm 4nm

PeakTable

PDA Ch1 254nm 4nm

Peak#	Ret. Time	Area	Height	Area %
1	18.837	40261	2275	1.954
2	19.417	2020078	103728	98.046
Total		2060339	106003	100.000

Method

<<LC Program>>

Time	Unit	Command	Value
0.10	Pumps	B.Conc	50
30.00	Pumps	B.Conc	95
33.00	Pumps	B.Conc	50
45.00	Controller	Stop	

Method Filename : FOS B.lcm

Shimadzu LC-20AD; 2-System FOS, Colon Kromasil 100-C18,.size 5mkm, 4,6*250mm, N 86912
Elution: A - H3PO4 0.01M pH 2.6; B - MeCN, fl. 1,0 ml/min, loop 20mkl.

Figure S131. HPLC chromatogram of **Compound 36**
